# Postmastectomy Breast Reconstruction in Patients with Non-Metastatic Breast Cancer: A Systematic Review

**DOI:** 10.3390/curroncol32040231

**Published:** 2025-04-16

**Authors:** Toni Zhong, Glenn G. Fletcher, Muriel Brackstone, Simon G. Frank, Renee Hanrahan, Vivian Miragias, Christiaan Stevens, Danny Vesprini, Alyssa Vito, Frances C. Wright

**Affiliations:** 1 Plastic and Reconstructive Surgery, University Health Network, Toronto, ON M5G 2C4, Canada; 2 Department of Surgery, University of Toronto, Toronto, ON M5T 1P5, Canada; hanrahanr@rvh.on.ca; 3 Program in Evidence-Based Care, Department of Oncology, McMaster University, Hamilton, ON L8V 5C2, Canada; gfletche@mcmaster.ca; 4 Department of Surgery, London Regional Cancer Program, London, ON N6A 5W9, Canada; muriel.brackstone@lhsc.on.ca; 5 Departments of Surgery and of Oncology, University of Western Ontario, London, ON N6A 5W9, Canada; 6 Department of Surgery, The Ottawa Hospital, Ottawa, ON K1H 8L6, Canada; sfrank@toh.ca; 7 Division of Plastic and Reconstructive Surgery, University of Ottawa, Ottawa, ON K1Y 4E9, Canada; 8 Department of Surgery, Royal Victoria Regional Health Care Centre, Barrie, ON L4M 6M2, Canada; 9 Department of Surgery, McMaster University, Hamilton, ON L8S 1C7, Canada; 10 Patient Representative, Toronto, ON, Canada; 11 Radiation Treatment Program, Royal Victoria Hospital, Barrie, ON L4M 6M2, Canada; stevensc@rvh.on.ca; 12 Departments of Radiation Oncology and of Family and Community Medicine, University of Toronto, Toronto, ON M5T 1P5, Canada; 13 Department of Radiation Oncology, Odette Cancer Centre, Sunnybrook Hospital, Toronto, ON M4N 3M5, Canada; danny.vesprini@sunnybrook.ca; 14 Department of Radiation Oncology, University of Toronto, Toronto, ON M5T 1P5, Canada; 15 Patient Representative, Port Perry, ON, Canada; alyssavito87@gmail.com; 16 Department of Surgery, Sunnybrook Health Sciences Centre, Toronto, ON M4N 3M5, Canada; frances.wright@sunnybrook.ca; 17 Departments of Surgery and of Health Policy, Management and Evaluation, University of Toronto, Toronto, ON M5T 1P5, Canada; 18 Surgical Oncology Program, Ontario Health (Cancer Care Ontario), Toronto, ON M5G 2L3, Canada

**Keywords:** breast reconstruction, breast implants, autologous reconstruction, autologous fat grafting, acellular dermal matrix, prepectoral, subpectoral, immediate reconstruction, delayed reconstruction, nipple-sparing mastectomy

## Abstract

Breast reconstruction after mastectomy improves the quality of life for many patients with breast cancer. There is uncertainty regarding eligibility criteria for reconstruction, timing (immediate or delayed—with or without radiotherapy), outcomes of nipple-sparing compared to skin-sparing mastectomy, selection criteria and surgical factors influencing outcomes of nipple-sparing mastectomy, prepectoral versus subpectoral implants, use of acellular dermal matrix, and use of autologous fat grafting. We conducted a systematic review of these topics to be used as the evidence base for an updated clinical practice guideline on breast reconstruction for Ontario Health (Cancer Care Ontario). The protocol was registered on PROSPERO, CRD42023409083. Medline, Embase, and Cochrane databases were searched until August 2024, and 229 primary studies met the inclusion criteria. Most studies were retrospective non-randomized comparative studies; 5 randomized controlled trials were included. Results suggest nipple-sparing mastectomy is oncologically safe, provided there is no clinical, radiological, or pathological indication of nipple-areolar complex involvement. Surgical factors, including incision location, may affect rates of complications such as necrosis. Both immediate and delayed reconstruction have similar long-term outcomes; however, immediate reconstruction may result in better short to medium-term quality of life. Evidence on whether radiotherapy should modify the timing of initial reconstruction or expander-implant exchange was very limited; studies delayed reconstruction after radiotherapy by at least 3 months and, more commonly, at least 6 months to avoid the period of acute radiation injury. Radiation after immediate reconstruction is a reasonable option. Surgical complications are similar between prepectoral and dual-plane or subpectoral reconstruction; prepectoral placement may give a better quality of life due to lower rates of long-term complications such as pain and animation deformity. Autologous fat grafting was found to be oncologically safe; its use may improve quality of life and aesthetic results.

## 1. Introduction

Mastectomy to treat breast cancer often has psychological sequelae, including alterations in body image, feelings of attractiveness, sexual health, and a continuing visual reminder of cancer. Breast reconstruction using saline or silicone-filled implants and/or autologous tissue restores appearance and, often, quality of life (QoL) [1,2,3,4,5]. Aesthetic results and patient satisfaction vary widely. While techniques such as nipple-sparing mastectomy (NSM) and skin-sparing mastectomy (SSM) [6,7,8] with or without the use of acellular dermal matrix (ADM) have been widely adopted, there is uncertainty regarding technical factors and safety. Use of acellular dermal matrix (ADM) has allowed a rapid switch from primarily submuscular implants to subpectoral/dual-plane and prepectoral implants [9]. The cost of ADM is often a major issue but is sometimes offset by fewer operations. Circumstances where ADM is beneficial and potential adverse effects are not well-defined [10,11,12,13,14]. The timing of breast reconstruction is broadly classified as immediate (commenced during the same operation as mastectomy) or delayed (commenced after mastectomy healing or even years later). The optimal timing, especially with respect to the use of postmastectomy radiation therapy (PMRT), has been unclear [15,16,17,18,19]. While breast reconstruction provides many benefits to the breast cancer patient, the uncertainty around techniques, timing, and use of adjunct materials prompted a new systematic review to be conducted to provide better clinical practice guidelines.

## 2. Materials and Methods

The Surgical Oncology program of Ontario Health (Cancer Care Ontario) identified a need to update the 2016 clinical practice guideline on breast reconstruction [20] and was a sponsor of this work. The Program in Evidence-Based Care (PEBC) at McMaster University assembled a working group to conduct a new systematic review to be used as the evidence base for the revised clinical practice guideline. The group had expertise in general surgery/surgical oncology, plastic surgery, radiation oncology, health research methodology, and patient representatives with personal or family experience with cancer. The working group determined the research questions together with the sponsor and then developed the review protocol; prior to starting the systematic review, it was registered with PROSPERO (an international prospective register of systematic reviews, University of York, UK) with registration number CRD42023409083 [21]. The guideline recommendations and extended version of the systematic review will be available on the Ontario Health (Cancer Care Ontario) website [22]. This review was conducted in accordance with the Preferred Reporting Items for Systematic Reviews and Meta-analyses (PRISMA) guidelines [23].

### 2.1. Research Questions

1. What is the effect of patient factors (smoking status, body mass index, breast size, age), comorbidities (diabetes, hypertension), or oncologic factors (previous breast surgery, previous radiotherapy (RT) to the breast/chest, inflammatory breast cancer, skin involvement) on post-mastectomy breast reconstruction outcomes?
2a. In patients with breast cancer undergoing therapeutic mastectomy, is there a difference in outcomes in immediate versus delayed reconstruction for patients who do not receive RT?
2b. In patients with breast cancer undergoing therapeutic mastectomy, is there a difference in outcomes in immediate versus delayed reconstruction for patients who receive RT?
3a. In patients with breast cancer who are candidates for SSM/NSM and reconstruction, is there a difference in outcomes between NSM and SSM?
3b. In patients with breast cancer, do oncologic outcomes for NSM vary according to the criteria used in selecting patients for NSM (e.g., tumour to nipple distance) or how nipple/areolar involvement is assessed (e.g., clinical examination, mammography, MRI, other imaging, biopsy of areola/nipple/nipple core, frozen/intraoperative or permanent section)?
3c. In patients with breast cancer and NSM, what surgical factors have been reported that influence the rates of nipple viability or necrosis and retention of sensation after NSM?
4. Does the use of prepectoral implants for postmastectomy breast construction result in differences in outcomes than subpectoral implants?
5. After therapeutic mastectomy, do outcomes differ for breast reconstruction using human-derived ADM, synthetic absorbable matrix, or no scaffolding/matrix? Are there differences in outcomes between different human ADMs or different synthetic absorbable matrices?
6. What are the benefits and risks of autologous fat grafting (lipofilling) as an adjunct to breast reconstruction?

### 2.2. Literature Search

A literature search was conducted in February 2023 and updated on 21 August 2024, using OVID databases of Medline, Embase, EBM Reviews–Cochrane Central Controlled Trials, and EBM Reviews–Cochrane Database of Systematic Reviews. The search strategies are reported in Table S1. The search contained terms for breast cancer, breast reconstruction, ADM, and fat grafting. Citations were exported to Endnote X9.3.3 (Clarivate, Philadelphia, PA, USA) for screening.

### 2.3. Study Selection Criteria

#### 2.3.1. Systematic Reviews

Systematic reviews were included if they were current (<5 years old), comprehensive, addressed the research questions, and used similar inclusion criteria as in our protocol. It was determined that no systematic reviews adequately addressed Questions 2 to 6. We included recent systematic reviews for Question 1 that focused on one or more of the patient factors, comorbidities, or oncologic factors listed in the research question, were identified as a systematic review or meta-analysis, used a systematic search, reported databases search and results of the search, listed included studies and data from them, and followed some standard such as the Preferred Reporting Items for Systematic reviews and Meta-Analyses (PRISMA) [23,24]. Reviews meeting these criteria but with a critical risk of bias using the AMSTAR II tool were excluded.

#### 2.3.2. Primary Studies

Studies were included if they addressed one of the research questions, reported one or more of the outcomes of interest (see next section), and were conducted in patients with non-metastatic breast cancer receiving therapeutic mastectomy and breast reconstruction. Studies on breast augmentation, reduction, or mastectomy for other than cancer were excluded. Studies in mixed populations but primarily focused on therapeutic mastectomy were included, provided patients with therapeutic mastectomy were reported separately, or patients without breast cancer (e.g., bilateral risk-reducing prophylactic mastectomy or congenital conditions) did not comprise more than 20% of the patients. For some questions, most patients had a therapeutic mastectomy and contralateral prophylactic mastectomy; while this was noted and considered a potential confounding factor, they were not excluded.

Except for Questions 3b and 3c, studies had to be either randomized controlled trials (RCTs) with ≥30 patients per arm or other comparative studies with ≥50 patients per arm. For subgroup analysis, these minimum patient numbers are applied to the subgroups. For Question 3b (oncologic safety of NSM) and Question 3c (surgical factors in NSM), non-comparative studies with ≥100 patients were included if they reported both criteria for conducting NSM and oncologic outcomes (Question 3b) or reported both surgical details/technique and outcomes of nipple viability/necrosis, sensation, and erection (Question 3c). Abstract-only publications were only included for RCTs. Letters, comments, commentaries, viewpoints, editorials, notes, and news articles were excluded.

Non-randomized comparative studies were included only if there was an indication that groups were similar or equivalent in key characteristics. Matching (e.g., propensity score matching), stratification, or multivariable analysis were preferred. While multivariable and multivariate are distinct types of analysis, these terms are often used interchangeably in the medical or public health literature [25,26], and articles using either term were included if their statistical analysis attempted to determine the effect of the intervention of interest while adjusting for confounding by other independent variables [27]. Variables should include those with plausible or known influence on the outcome, as well as those found in initial univariate analysis to be significant at an arbitrary level, such as *p* = 0.2 or *p* = 0.05 [28,29,30]. While *p* = 0.05 is often used, this may exclude variables that, when combined, have an important effect and was considered suboptimal. Studies with multivariable analysis but which failed to include variables generally considered to affect the outcome reported were excluded. Variables varied among questions and outcomes and generally included stage or other indications of disease severity such as use of RT or chemotherapy, patient characteristics (e.g., BMI), comorbidities (e.g., diabetes), or habits such as smoking. In order to have reliable results, there needed to be both a sufficient number of patients and a sufficient number of events. There is a rule of thumb that 10 events are required for each variable being adjusted for; while this is often debated as being too lax or too strict, it gives a starting point in the decision process as to whether the analysis is adequate [31,32,33,34,35,36,37]. It was decided to exclude studies with <25 events per outcome (or, in the case of multiple outcomes of interest, to only extract data for outcomes that had ≥ 25 events). This often meant that for complications, only the outcome of total complications or major complications had sufficient events, while individual outcomes did not. Studies specifying multiple or multivariate linear regression, multiple or multivariate logistic regression, or Cox proportional hazards analysis were included if they met the above requirements. Studies that only mentioned univariate analysis or tests that were generally considered univariate (*t*-test, Chi2, Fisher’s exact, Mann-Whitney, Wilcoxon rank sum) were generally excluded.

Equivalence could also be assumed by the presence of narrow inclusion criteria such that groups were equivalent by nature of the selection criteria or broader criteria, with the authors indicating that groups were similar/not statistically different and supported by a table comparing patient and disease characteristics for both (all) groups. A judgement was then made as to whether differences were likely to affect results. For example, for oncologic outcomes of recurrence and survival, groups had to be of similar stage, tumour size, lymph node status, and distribution of cancer types (e.g., in situ, invasive). For most other outcomes, the type of reconstruction and indication for reconstruction had to be similar.

#### 2.3.3. Outcomes of Interest

A wide range of outcomes were identified as of interest, acknowledging these would vary for different questions. The full list follows and applies to Questions 1, 2, and 4 (except donor site problems). Question 5 was concerned with surgical complications, aesthetics, and patient-reported outcomes (PROs). Question 6 included outcomes specific to fat grafting (surgical outcomes of fat necrosis, infection, wound complications, reoperations, hematoma, seroma, capsular contraction, oil cysts, skin necrosis; aesthetics; PROs; oncologic outcomes of recurrence or survival; and non-comparative outcomes only in the fat grafting arm such as donor site numbness, pain, contour defects, bruising, ecchymosis, hematoma, swelling, and infection). For Question 3, outcomes were limited to oncologic outcomes (3a and 3b), surgical outcomes and aesthetics (3a), nipple viability and necrosis (3a, 3b, 3c), and nipple sensation/erection (3c).

(a) Surgical complications:
  - Short-term (<30 days): seroma, hematoma (bleeding), infection, flap necrosis, nipple necrosis, wound complications, reoperations, pulmonary embolus or deep vein thrombosis
  - Long-term: flap failure, loss of implant, fat necrosis, reoperation, capsular contracture (implants only), implant malfunction (leaking, rupture, shift), chronic breast pain, hypertrophic scarring
(b) Donor site problems: hernia, wound complications, abdominal weakness
(c) Functional: restricted mobility, decreased strength, pectoralis tightness, animation deformity, lymphedema. This may also be phrased as being able/unable to perform work and leisure-related activities and assessed using a validated instrument
(d) Aesthetics (acceptable cosmetic outcome: volume, shape, symmetry, scarring, skin quality) assessed by surgeons and/or patients (see also PROs)
(e) PROs: patient satisfaction with breasts, satisfaction with the overall outcome, psychosocial well-being, physical well-being, sexual well-being, health-related QoL, body image, sexual functioning
(f) Oncologic outcomes: delay in adjuvant therapy (radiation or chemotherapy), recurrence, disease-free survival (DFS), overall survival (OS)

### 2.4. Data Extraction and Assessment of Evidence Quality/Certainty

All included primary studies underwent data extraction by GGF, with all extracted data and information audited subsequently by an independent auditor. Ratios, including hazard ratios or odds ratios, were expressed with a ratio of <1.0, indicating improvement in survival or reduction in recurrence or other adverse events for the experiment group (or first group) compared with the control group (or second group).

A decision was made not to complete data screening and extraction for Question 1 when other systematic reviews on portions of this topic were identified; these were assessed using the AMSTAR II tool [38] (see Table A2).

The risk of bias for randomized studies was assessed by GGF using methods outlined in the Cochrane Handbook for Systematic Reviews of Interventions [39,40,41]. The Cochrane risk-of-bias (RoB) tool (revised version RoB2) for RCTs and Risk of Bias In Non-randomized Studies of Interventions (ROBINS-I) for non-RCTs are described in this handbook and other publications [42,43,44]. The risk of bias was not assessed for non-comparative studies. As described in the PEBC Handbook [45] and PEBC Methods Handbook [46], the quality of studies was evaluated considering quality-related features of the studies, including threats to validity. Studies with particular factors affecting bias or quality are noted in the data tables and/or narrative results. The AGREE II framework [47] was used. In evaluating the overall quality of the evidence, we considered the risk of bias, precision, and consistency of results, as well as how well the evidence answered the questions. Publication or reporting bias was not considered due to the predominance of retrospective studies from medical records, the small number of similar studies per comparison, and because meta-analysis was not conducted for most questions.

### 2.5. Synthesizing the Evidence

When clinically homogeneous results for several studies were available, a meta-analysis was conducted using Review Manager 5.4 software (RevMan) provided by the Cochrane Collaboration [48]. This was only conducted for Question 6 (fat grafting). The generic inverse variance model with random effects was used. While there is some debate as to whether odds ratio (OR) or relative risk is more meaningful and easier to interpret for clinical studies, multiple logistic regression calculates adjusted ORs [49,50,51,52]. ORs and confidence intervals (CIs) were, therefore, the preferred statistics for meta-analysis. For retrospective studies with multivariate analysis to adjust for confounding, outcomes with adjusted ORs were reported. Forest plots include all studies, with data in subgroups according to subtype of recurrence or survival outcome. Summary statistics (range, mean, median) were calculated for Questions 3b and 3c.

## 3. Results

### 3.1. Overview of the Literature Search Results

**Figure 1:**
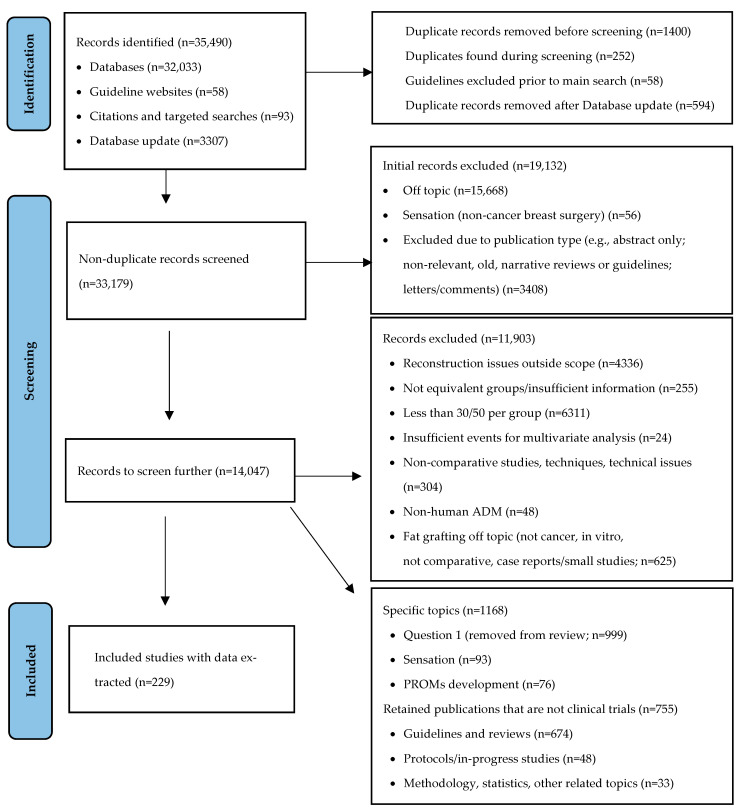
PRISMA Flow Diagram. Format adapted from Page et al. [23].

After the removal of duplicates in OVID, 32,033 citations were exported to Endnote. Repeated searching for duplicates in Endnote identified an additional 1400 citations that were excluded as duplicates, leaving 30,633 records to screen. The updated search identified an additional 3307 citations; after the removal of duplicates from the new search and with publications already included, there were 2734 additional publications. A review of the titles, as well as abstracts and full text if required, was conducted by one reviewer (GGF). A PRISMA diagram showing the search results is provided in Figure 1. There were 229 primary studies that met the inclusion criteria.

RCTs were rare, and completed RCTs were only found for Question 5 (3 trials in 6 publications) and Question 6 (2 trials in 3 publications). Most other studies were of retrospective design; they often had prospective charting or entering into the database but retrospective study design and data analysis. A small proportion of studies are described as prospective; however, it is suspected that some of the analyses were retrospectively designed. Further breakdown of study type is provided in the subsections for each research question.

### 3.2. Risk of Bias and Quality of Evidence

#### 3.2.1. Randomized Controlled Trials

Of the four RCTs for Question 5 on ADM use, all had a high risk of bias (see Appendix A) as assessed by the Cochrane RoB2 tool and evaluated as providing low certainty of evidence. For the two RCTs on fat grafting for Question 6, Gentilucci et al. [53] was rated as low risk of bias, and the Breast Trial [54,55,56,57] was rated as having some concerns due to deviations from intended interventions but otherwise low risk of bias. These studies provide high-quality evidence on narrow topics.

#### 3.2.2. Other Studies

Most publications were of non-randomized comparative studies. The following applies to all questions; more details for specific studies are included in the extended version of the systematic review [22]. Based on the ROBINS-I tool, there was potential for confounding (Domain 1) that was generally controlled for but to varying degrees in different studies. As only studies with equivalent baseline characteristics, matching, or multivariable/multivariate analysis were included in this systematic review, studies were generally of low to moderate risk of bias. Comments on this are included in the data tables for each question. In general, the included comparative studies were assessed as having a low risk of bias for Domain 2 on selection bias, Domain 3 on classification bias, and Domain 4 on departure from interventions. Missing data (Domain 5) are of potential concern for most non-randomized studies as there were a wide range of possible outcomes, and hospital records likely did not capture them all; this is apparent by a lack of consistency in reported complications in different studies but is not expected to vary between arms within a particular study. For studies reporting results from the same surgeon or group of surgeons at the same institution, this is considered to be minor (low risk of bias); however, they contribute to inconsistency between studies. For large database studies representing results from many institutions, these factors contribute to a moderate risk of bias. Domain 6 (bias in outcome measurements) is considered low risk of bias, except for complications such as degree of necrosis that are not well defined. This is more of an issue when comparing studies than within studies. Domain 7 (bias in the selection of reported results) is of low risk of bias. Overall, the risk of bias for non-randomized comparative studies is low to moderate.

Studies using national or other large multi-institutional databases included thousands of patients and therefore provided some data not available elsewhere, but many important details, including surgical and pathological ones, were not available, therefore limiting the usefulness even when the study itself was well-designed. General conclusions, such as whether reconstruction could be used in patients with specific characteristics, were of high certainty, whereas they provided low-quality or no evidence on more specific issues, such as how to assess patients as being suitable for a specific type of reconstruction or surgical details to minimize complications or recurrence. Some of these limitations are noted in Section 3 for each research question.

Studies (often from a single institution or single surgeon) with well-conducted matching or multivariate analysis with sufficient numbers of patients and events and appropriate selection of variables were considered to have a low risk of bias and provide moderate to high-quality evidence. For non-RCT comparative studies, our threshold of 50 patients per group and requirement for either similar baseline factors or adjustment for this (matching or multivariable/multivariate analysis) eliminated many of the studies of the lowest strength of evidence. Due to a low number of individual complications, often only composite or total complications had sufficient numbers of events. Studies with matching (propensity score matching) were generally rated as moderate quality, as were larger studies that used multivariate/multivariable analysis.

When evaluating the evidence, consistency between outcomes was important. Study designs and patient populations were considered to be too diverse to conduct a meta-analysis for most questions except the effect of fat grafting on cancer recurrence (see Question 6). Summary statistics were also reported for oncologic outcomes and necrosis after NSM (see Question 3). When small but statistically significant differences were reported for complications, and these differed widely among studies, it was considered insufficient evidence to conclude the effect could be extrapolated to other studies. Complications were often reduced by subsequent modifications by the investigators and were not inherent to the topic of study.

In some areas with insufficient comparative evidence or with a need for further details), we have identified other publications considered to be informative and described them, clearly stating they are not part of the studies meeting the inclusion criteria or included in the data tables. We consider this appropriate for the field of surgery in which a baseline of prior results by the same surgeon is used, modifications are made, and the effect is noted. The skill and expertise of surgeons vary greatly, and therefore, such results may be useful. The formal system of evaluation of the quality of evidence used for comparative studies is not appropriate. Recentness, consistency, quality of reporting, importance of outcomes, and other publications by the authors were considered. These do not address the primary question of whether to do something but rather technical details on how to do it.

#### 3.2.3. Mastectomy Reconstruction Outcomes Consortium Study

The MROC study addresses several different questions, and therefore, a broad description is given here instead of repeating the analysis for each question. MROC was a prospective multicentre observational cohort study funded by the National Cancer Institute (USA) [58,59]. It was conducted at nine institutions in the United States and two in Canada [60], although embedded studies did not always include all 11 institutions. The MROC study was designed to evaluate and compare various options for breast reconstruction. It included 4464 women undergoing immediate or delayed breast reconstruction after mastectomy using tissue expander/implant or autologous flaps [59,61]. The primary outcome was the change from baseline in health-related quality of life (HRQoL), with other outcomes including complications. It accepted patients with either therapeutic or prophylactic mastectomy but excluded patients undergoing reconstruction due to complications of breast augmentation, mastopexy, or breast reduction.

Thirty publications of the MROC study reporting on different aspects of the trial were identified in the literature search. The full set of patients was not used in any of these reports. Most were secondary analyses with their own inclusion and exclusion criteria and only used subsets of patients with specific characteristics or treatments. It is suspected that many of the analyses were retrospectively designed using the prospectively collected data. PRO or HRQoL data were collected using the BREAST-Q questionnaire; however, data were not available for all patients at all time points, and 1360 patients did not complete the baseline survey. Sample size calculations to ensure statistically significant results were not reported in most papers.

For PROs, baseline refers to before reconstruction [62], and therefore, the baseline data were before mastectomy only for the immediate group; for the delayed group, the baseline was after mastectomy (and PMRT when used). This accounts for the much poorer baseline PRO data for the delayed reconstruction group [63]. The PRO model adjusted for baseline prior to reconstruction; there was no baseline prior to mastectomy for the delayed group, and therefore, the baseline does not measure the same state. Adjustments in the model may be inappropriate in determining the effect of the timing of reconstruction. Based on an analysis of data from this trial, it has been proposed that a 4-point difference on a BREAST-Q scale is a clinically useful minimal important difference score (MID) [64].

Of the publications of MROC found, five were included in our systematic review for Question 2 (three comparing immediate vs. delayed, not considering the effect of RT [63,65,66], one on timing of RT with respect to autologous reconstruction [67], and one on timing of RT with expanders and implants [68]), two for Question 5 (use of ADM [69,70]), and one for Question 6 (fat grafting [58]). These all appear to be secondary analyses with relatively small and unequal patient numbers in some arms. For example, the publication of immediate versus delayed reconstruction [63] had 1806 immediate and only 151 delayed reconstructions; for further subgroups by reconstruction type, there were only 17 patients with delayed implants (and therefore did not meet our inclusion criteria). The large imbalance means there is an extremely small number of outcome events in the smaller group. Other characteristics of the groups are non-equivalent; multivariate analysis may be inadequate. Despite the large amount of information in the MROC study, it is insufficient to give definitive answers to many of the questions explored in its publications.

### 3.3. Question 1: Patient Factors

Question 1 is based primarily on existing systematic reviews. Seventeen systematic reviews [15,18,71,72,73,74,75,76,77,78,79,80,81,82,83,84,85] were found that cover several of the frequently studied factors, and these tend to be those with stronger associations with increased risk of complications. These reviews are summarized in Table S2. It was determined that no published systematic review sufficiently addressed the question of age, and therefore, primary studies were reviewed and are summarized in Table S3 [86,87,88,89,90,91].

#### 3.3.1. Age

A systematic review of studies that compared older and younger age groups was undertaken (see Table S3). One prospective study (MROC, [88]), three single-institutional retrospective studies [86,87,89], and two retrospective multi-institution database studies [90,91] were included. The MROC study subdivided patients into three age groups (>60, 45–60, and <45 years old). Odds ratios for total and major complications for > 60 compared to < 45 were OR = 1.46, 95% CI = 0.99 to 2.15, *p* = 0.059 and OR = 1.43, 95% CI = 0.93 to 2.18, *p* = 0.101. Older patients (>60 years) compared to younger (<45 years) had less satisfaction with breasts with implants but no difference for autologous construction. There was improved sexual well-being for both implants and autologous reconstruction in older patients.

Some of the single institution studies used a higher cut-off for the oldest age group (age 70 or 75 years), although the number of patients aged > 70 years was considered sufficient in only two [86,87]. The effect of this limitation is reduced by the use of age as a continuous variable in both these studies and one additional one [89]. It was indicated that this is preferred over the use of age categories, which can lead to loss of power and incomplete correction of confounders, especially in studies limited by sample size [87].

Honig et al. [87] included 4185 free flaps from 2598 patients to evaluate the safety of their use in patients of advanced age. They used cubic spline curves to model age as a continuous variable and then reported estimates at 5-year intervals from ages 55 to 74 years; *p* values are assumed to be based on the trend over all ages. Comparing age 70 to 74 years versus age < 55 years, there were small increases in rates of delayed healing (35.7% vs. 29.2%, *p* = 0.036), skin necrosis (15.3% vs. 10.7%, *p* = 0.039), and hematoma (9.3% vs. 5.3%, *p* = 0.005). Other surgical complications, including seroma, surgical site infection, and flap loss, and medical complications, such as venous thromboembolism (VTE), did not increase with age. The authors concluded that an age cutoff was not warranted.

Kim et al. [86] used age as a continuous predictor in a set of 4379 patients and found complications (mastectomy skin flap/nipple necrosis, infection, seroma) increased by 1 to 2% per year of age. Using the BREAST-Q, Satisfaction with Breasts was lower in older patients, while Psychosocial Well-Being was higher, and there was no difference in Physical Well-Being-Chest or Sexual Well-Being. Chang et al. [89] included 818 patients and found age was not predictive of surgical complications when used as either a continuous variable or by age groups (60 to 69, 50 to 59, or <50 years old).

Two studies using the American College of Surgeons National Surgery Quality Improvement Program (ACS-NSQIP) database met our inclusion criteria [90,91]. This database has the limitation of only including short-term complications (within 30 days of operation). Cuccolo et al. looked at all pedicled flaps and also reported locations such as breast separately [90]. Multivariable analysis appears to use age as a continuous variable. They found a small increase in overall complications (OR = 1.010, 95% CI = 1.004 to 1.006, *p* = 0.002), severe complications (OR = 1.043, 95% CI = 1.019 to 1.068, *p* < 0.001), and wound complications (OR = 1.009, 95% CI = 1.000 to 1.018, *p* = 0.053), but concluded the effect of age does not have strong predictive power and may not be clinically relevant on its own. The other ACS-NSQIP study compared outcomes in patients ≥ 65 years versus < 65 years and found no significant differences. Adjusted OR (aOR) for any complication for implants was 1.16 (95% CI = 0.85 to 1.57, *p* = 0.346) and for autologous reconstruction was 1.16 (95% CI = 0.71 to 1.88, *p* = 0.555). As they were comparing two groups instead of using age as a continuous variable, this study had less power than that of Cuccolo et al. to detect a difference.

#### 3.3.2. Diabetes

Two recent systematic reviews of moderate quality on the effects of diabetes on reconstructive outcomes were found. Liu et al. [74] found higher overall complications (OR = 2.04, 95% CI = 1.86 to 2.25, *p* < 0.0001), surgical complications (OR = 2.23, 95% CI = 1.98 to 2.50. *p* < 0.0001), implant loss/flap failure (OR = 1.68, 95% CI = 1.27 to 2.23, *p* < 0.0001), infection (OR = 3.88, 95% CI = 2.32 to 6.51, *p* < 0.01), skin necrosis (OR = 2.82, 95% CI = 1.51 to 5.29, *p* = 0.001), and longer hospital stay (41.0% vs. 34.7% over 5 days long; OR = 1.31, 95% CI = 1.12 to 1.54, *p* < 0.01). Mortada et al. [75] found a higher rate of wound dehiscence but no significant difference in wound infection, total flap complications, or total flap loss. Other complications were similar but not included in the meta-analysis. While the reviews included a large number of studies (38 and 43), Mortada et al. only included a subset of five studies in the meta-analysis. A systematic review by Mrad et al. [77] on complications after breast reconstruction found that diabetes increased rates of major/re-operative complications (OR = 4.54, 95% CI = 1.63 to 12.64), and 90-day complications (OR = 1.59, 95% CI = 1.30 to 1.95, *p* < 0.00001). Results were also reported for any complication (OR = 1.18, 95% CI = 0.96 to 1.45, *p* = 0.13) and infection (OR = 1.44, 95% CI = 0.84 to 2.48, *p* = 0.19).

#### 3.3.3. Smoking

A systematic review on the role of smoking in elective plastic surgery [76] found ever smoking was a risk factor in breast reconstruction for postoperative complications (OR = 1.91; 95% CI = 1.69 to 2.17), donor site complications (OR = 1.59. 95% CI = 1.27 to 1.99, *p* < 0.001), infection (OR = 1.66, 95% CI = 1.05 to 2.63, *p* = 0.03), and fat necrosis (OR = 1.62, 95% CI = 1.06 to 2.48, *p* = 0.024). There were no significant differences in flap necrosis, reoperation, hematoma, or seroma. The review was rated as low quality due to the non-reporting of the risk of bias in individual studies, but it was strengthened due to the study size (26 studies and > 20,000 patients) and the consistency of results between studies. A systematic review by Mrad et al. [77] on complications after breast reconstruction found that smoking history increased the risk of any complication (OR = 2.43, 95% CI = 1.54 to 3.82, *p* = 0.0001), major/re-operative complications (OR = 1.46, 95% CI = 1.08 to 1.97), 90-day readmissions (OR = 2.13, 95% CI = 1.05 to 4.34, *p* = 0.04), and infection (OR = 1.52, 95% CI = 0.98 to 2.36, *p* = 0.06).

#### 3.3.4. BMI

A moderate-quality systematic review of DIEP flap reconstruction by Tan et al. [72] found that patients with BMI ≤ 25 kg/m^2^ (mean of 22.9 kg/m^2^) had no difference in complete or partial flap loss, fat necrosis, all complications, abdominal wound healing, infections, or seroma compared with patients with BMI > 25 to < 30 kg/m^2^ (mean BMI 27.9 kg/m^2^).

A moderate-quality systematic review by Panayi et al. [71] looked at the effect of obesity (BMI > 30 kg/m^2^) and found higher rates of surgical complications (RR = 2.29, 95% CI = 2.19 to 2.39, *p* < 0.00001), fat necrosis (RR = 1.65, 95% CI = 1.31 to 2.07, *p* < 0.0001), seroma (RR = 1.96, 95% CI = 1.57–2.45, *p* < 0.00001), partial flap failure (RR = 1.60, 95% CI = 1.06 to 2.41, *p* = 0.03), total flap failure (RR = 1.97, 95% CI = 1.34–2.91, *p* = 0.0006), wound dehiscence (RR = 2.51, 95% CI = 1.80–3.52, *p* < 0.00001), wound infection (RR = 2.34, 95% CI = 2.03 to 2.69, *p* < 0.00001), hernia (RR = 1.67, 95% CI = 1.15 to 2.43, *p* = 0.007), medical complications (RR = 2.89, 95% CI = 2.50 to 3.35, *p* < 0.00001), and return to operating room (RR = 1.91, 95% CI = 1.75–2.07, *p* < 0.00001). Surgical complications (based on a subset of 4 studies) increased with class of obesity: class I (30 to 34.9 kg/m^2^) RR = 1.32; class II (35 to 39.9 kg/m^2^) RR = 1.84; and class III (>40 kg/m^2^) RR = 1.66. A moderate-quality systematic review by ElAbd et al. [73] compared autologous versus implant reconstruction in patients with obesity and found that autologous reconstruction resulted in lower infection, hematoma, seroma, and reconstructive failure; there was no difference in skin necrosis or wound dehiscence but increased rates of deep vein thrombosis and pulmonary embolism. BREAST Q QoL was slightly better in the autologous patients (but not statistically significant, *p* > 0.05).

#### 3.3.5. Hypertension

A systematic review by Mrad et al. [77] on complications after breast reconstruction found that hypertension increased the risk of any complication (OR = 1.59, 95% CI = 1.23 to 2.05, *p* = 0.0004), major/re-operative complications (OR = 1.29, 95% CI = 1.03 to 1.62), 90-day readmissions (OR = 1.65, 95% CI = 1.06 to 2.57, *p* = 0.03), and infection (OR = 3.71, 95% CI = 1.14 to 12.07, *p* = 0.03). The review is rated low due to the non-reporting of risk of bias of individual studies but strengthened due to a large number of studies and patients (33 studies with over 100,000 patients, of which 7 studies reported any/total complications by hypertension status). While we did not conduct a systematic review on this topic, the studies included for other questions suggest the effect of hypertension to be low and, therefore, often not statistically significant in small studies. Most studies did not define this variable and may have used different cutoffs.

#### 3.3.6. Previous Surgery

A moderate-quality systematic review on the effect of prior abdominal surgery on abdominally based flap reconstruction [79] found a small increase in donor-site delayed wound healing (RR = 1.27, 95% CI = 1.00 to 1.61) and no statistically significant difference in other complications (flap complications of total and partial flap loss, fat necrosis, infection, reoperation; and donor site complications of seroma, hematoma, infection, and abdominal wall morbidity).

Two systematic reviews of low quality were also relevant. One on the effect of pre-existing abdominal scars on reconstruction using abdominal flaps [78] found no significant difference in flap complications or flap loss. There were higher donor-site complications than in control patients without abdominal scars (19.5% vs. 14.5%, RR = 1.35, 95% CI = 1.13 to 1.62, *p* = 0.001). The authors suggest that the constraints of the scar may be overcome by technical modifications and the use of computed tomography angiography if there is uncertainty. A systematic review of complications in patients with prior breast augmentation compared with those without [80] found early complications of 36.7% versus 24.8% (OR = 1.57, 95% CI = 0.94 to 2.64, *p* = 0.09), hematoma of 3.39% versus 2.15% (OR = 2.68, 95% CI = 1.00 to 7.16, *p* = 0.05), and no difference in seroma, infection, skin flap necrosis, or prosthesis loss. Late complications were 10.1% versus 19.9% (OR = 0.53, 95% CI = 0.06 to 4.89, *p* = 0.57), and overall complications 36.5% versus 31.2% (OR = 1.23, 95% CI = 0.76 to 2.00, *p* = 0.40).

#### 3.3.7. Neoadjuvant Chemotherapy

A high-quality systematic review [81] compared reconstruction in patients with and without neoadjuvant chemotherapy (NACT) and did not find a statistically significant difference in overall complications (RR = 0.91, 95% CI = 0.74–1.11, *p* = 0.34), flap loss (RR = 0.94, 95% CI = 0.46 to 1.94, *p* = 0.87), hematoma (RR = 0.99, *p* = 0.97), or wound complications (RR = 1.15, *p* = 0.22). There was an increase in implant/expander loss (17.4% vs. 11.3%, RR = 1.54, 95% CI = 1.04 to 2.29, *p* = 0.03). Most studies did not report adjusted risk estimates, and patients with NACT often had more advanced diseases.

#### 3.3.8. Radiotherapy

Four systematic reviews (2 moderate and 2 low quality) on the effect of PMRT on implant-based breast reconstruction [15,83,84,85] found increased rates of complications with PMRT (see Table S2). There was an increase in early complications, including surgical site infection, mastectomy skin flap necrosis, and implant extrusion/exposure, and late complications, including reconstructive failure, capsular contracture, and the need for revisional surgery. Adjuvant RT also resulted in lower patient satisfaction and aesthetic results.

A high-quality systematic review of PMRT in conjunction with autologous flap reconstruction [18] found higher rates of fat necrosis (RR = 1.91, 95% CI = 1.45 to 2.52, *p* < 0.00001), secondary surgery (RR = 1.62, 95% CI = 1.06 to 2.48, *p* = 0.03), and volume loss (RR = 8.16, 95% CI = 4.26 to 15.63, *p* < 0.00001). No difference was found for infection (RR = 1.14, *p* = 0.60), healing complications (RR = 1.23, *p* = 0.17, hematoma (RR = 1.14, *p* = 0.81), seroma (RR = 1.19, *p* = 0.67), total flap loss (RR = 0.80, *p* = 0.81), or partial flap loss/necrosis (RR = 0.34, *p* = 0.15). Cosmetic results (observer-reported) were lower with PMRT in four of five studies. It was noted that there are higher risks with PMRT, but these are not necessarily clinically significant.

### 3.4. Question 2: Immediate Versus Delayed Reconstruction

Question 2 deals with the timing of reconstruction and the timing of RT in relation to mastectomy and reconstruction. Results of the literature search are provided in Table S4 and include 16 non-randomized comparative studies in 20 publications (plus two references for additional details) [16,17,19,62,63,64,65,66,67,68,92,93,94,95,96,97,98,99,100,101,102,103]. Complications can arise from either the mastectomy or reconstruction processes. Immediate reconstruction occurs (or is initiated) at the same time as mastectomy, and total complications at this point and during follow-up could have components from either procedure. Expander-implant exchange or revision surgeries may also be required, and complications need to be assessed as part of the overall evaluation. Publications did not distinguish between mastectomy-related and reconstruction-related complications. Delayed reconstruction is conducted months or years after mastectomy, and at a minimum after surgical healing has occurred, and usually after adjuvant treatment is completed. Studies on delayed reconstruction usually did not specify the timing of adverse events measured; rather, they generally appear only to capture those at the time of reconstruction or later. Most studies did not report sufficient details of when the assessment was performed and whether they excluded any types of complications. Immediate reconstruction entails a longer initial operation, and operating time and some types of surgical complications are correlated. In contrast, delayed reconstruction requires additional surgery and risks associated with additional surgery and anesthesia. In the absence of studies that assess all outcomes from the time of mastectomy until years after the reconstruction process is complete, interpretation of some outcomes is difficult. PROs such as anxiety may improve after mastectomy and cancer treatment, while body image would be expected to be highest prior to mastectomy and lowest between mastectomy and delayed reconstruction, with recovery after reconstruction is complete. Studies often did not use equivalent baselines, with some comparisons being before and after reconstruction without considering when the mastectomy occurred.

#### 3.4.1. Question 2a: Studies Without RT Comparing Delayed to Immediate Reconstruction

Three studies, namely a national audit of mastectomy and breast reconstruction in England [92,93], the MROC study [63,65,66], and a study by Knoedler et al. [94] using the American College of Surgeons National Surgical Quality Improvement Program (ACS-NSQIP) database included patients with a mix of implants or autologous reconstruction. RT was used in some patients but not reported separately in relation to the timing of reconstruction. These studies are confounded by the timing of assessments of complications and HRQoL.

Knoedler et al. [94] reported complications in subgroups of patients with implants and patients with autologous flaps, as well as combined data. Any complications, general complications (30-day mortality, reoperation, readmission, unplanned readmission) and surgical compilations (superficial incisional infection, deep incisional infection, organ space infection, dehiscence, and bleeding) were higher in the immediate group. There was no significant difference in medical complications. Seroma and hematoma were not reported, and only complications in the first 30 days were recorded, so capsular contraction, aesthetics, sensation, and oncologic outcomes were not available. Delayed reconstruction requires two operations, and it is unclear whether the complications of each were added together. For any/general/surgical/medical complications, all were higher in the immediate group; this may reflect a combination of mastectomy plus reconstruction complications being captured but only reconstruction complications in the delayed group.

In the audit [92,93], the BREAST-Q was administered 18 months after surgery, but it is not mentioned whether it was after the mastectomy surgery or reconstructive surgery. There is no baseline data (prior to mastectomy), and there is no information on the duration of delay between mastectomy and reconstruction. While it is reported to be a prospective design, due to the nature of the audit, patients who had mastectomy prior to the start of the study but delayed reconstruction during the study were included. The timing of measurement of complications is unclear but may not include complications of mastectomy in the delayed reconstruction group. This study was an audit of hospital performance, and variation between institutions is often greater than between patient groups. The risk of complications was not associated with reconstructive timing. PRO differences were generally less than a clinically useful minimal important difference score. An exception is for Sexual Well-Being, for which the delayed group had better scores; however, the authors note that the response rate was much lower for this question and varied across hospital organizations.

In MROC [63,65,66], the baseline BREAST-Q was administered prior to reconstruction (prior to mastectomy in the immediate group but much after mastectomy in the delayed group), and therefore, patients had lower values in the delayed group. At 2 years after reconstruction, they found no significant differences in adjusted PROs. Complications were determined by reviewing medical records 1 and 2 years after reconstruction; it is unclear whether they went back to the time of mastectomy in both groups. Patient characteristics were not equivalent for immediate and delayed groups, and about 93% of reconstructions were immediate. For implants, 98% of patients had immediate reconstruction (58.5% bilateral). Delayed reconstruction with implants was rare (only 27 and 34 patients in two publications), and therefore most delayed cases were autologous. A comparison of immediate versus delayed implants did not meet our inclusion criteria due to the low number of patients in the delayed group. For autologous reconstruction, 82% of patients were immediate (30.2% bilateral). In Yoon et al. [63], 70.6% of immediate reconstructions used implants, while 70.2% of delayed reconstructions were autologous reconstructions. The large imbalance of immediate and delayed cases by implant type raises concerns about the ability to sufficiently correct for differences in the mixed effects logistic regression model used for major complications (those requiring re-hospitalization or re-operation) and any/total complications. The immediate group had significantly more total and major complications (aOR = 2.63, *p* < 0.001 and aOR = 1.92, *p* = 0.016). The report on this study by Wilkins et al. [66] reported similar results for any complications (OR = 1.82, *p* = 0.017) but no difference in major complications (OR = 1.17, 95% CI = 0.68 to 2.00, *p* = 0.566). This difference points out the uncertainty of these results, which may be due to the inability to correct for all risk factors, the small number of delayed cases, and the strong association between timing and type of surgery.

Eight retrospective studies compared immediate versus delayed autologous reconstruction [17,19,95,96,97,98,99,100,101]. The study by Joosen et al. [97] and Beugels et al. [19] is the only one that reported oncologic outcomes, as well as complications. The delayed group had more systemic therapy and RT, history of expanders/implants, longer follow-up, and less history of lumpectomy. These suggest that the delayed group had different characteristics and more advanced disease. Six patients were excluded from the delayed group due to recurrence prior to reconstruction. This high rate of cancer recurrences also suggests a greater risk in the delayed group because of more advanced disease that is not related to reconstruction. Local and regional recurrence rates were all low (1.5% versus 1.7%; 3.7% versus 2.4%) and insufficient events to meet our criteria of multivariate analysis, although it needs to be mentioned that the apparent direction of benefit switched after multivariable regression. Distant metastasis was 2.8% versus 6.9%, HR = 1.351, *p* = 0.085; after adjustment HR = 5.244, *p* < 0.001. The large changes in all endpoints from before to after multivariable regression suggest that groups may have been too disparate for this analysis, and residual confounders may still be present. Results are, therefore, considered unreliable. This study reported complications in the subgroups with breast cancer and found higher rates of hematoma and seroma in the immediate group and fewer wound problems; major complications were similar in each group.

Kroll et al. [95] found better aesthetic scores with immediate reconstruction (mean aesthetic score 3.25 vs. 2.82, adjusted *p* = 0.0001) and suggested it may be due to the associated use of SSM. Bilateral reconstruction and nipple reconstruction both resulted in higher scores, while prior irradiation resulted in a lower score. Prantl et al. [17] studied DIEP flap reconstruction and found similar complication rates, including flap loss, between immediate and delayed reconstruction. This was the largest study (897 patients immediate and 3016 patients delayed); while multivariate analysis was not used, groups had similar comorbidities.

Shammas et al. [98] used claims codes to study immediate, delayed, and staged (delayed-immediate with expander at the time of mastectomy) free-flap reconstruction and associated complications within 90 days of either operation. The immediate group received less chemotherapy and RT but was older and had more bilateral mastectomies. In unadjusted data, surgical, systemic, and any complications were lowest in the immediate group and highest in the staged group. After adjustment, the relative risk of at least one complication was lower for immediate versus delayed (RR = 0.78, 95% CI = 0.68 to 0.88, *p* < 0.001), immediate versus staged (RR = 0.60, 95% CI = 0.53 to 0.67, *p* < 0.001), and delayed versus staged (RR = 0.77, 95% CI = 0.67 to 0.88, *p* < 0.001). Results were similar in sensitivity analysis in patients without RT. When looking at the results tables, part of the increased complications in delayed and staged groups was due to additive complications as a result of two operations (mastectomy and later reconstruction) compared with one operation in the immediate setting.

Huang et al. [99] found similar complications in immediate and delayed groups with DIEP flap reconstruction, except for higher breast skin necrosis in the immediate group. Authors suggest lower rates in the delayed group may be due to additional time to revascularize and heal after mastectomy.

Marquez et al. [100] and Kalmar et al. [101] both used the ACS NSQIP database and, therefore, have the same limitations as Knoedler et al. [94]. Marquez et al. confirmed that for immediate reconstruction, “it is difficult to distinguish between complications related to the mastectomy versus those related to reconstructive efforts”. They found increased odds of surgical complication in the immediate group compared with delayed or delayed-immediate, while there was no significant difference between delayed-immediate and delayed (both of which require a second operation). Kalmar et al. [101] looked only at flap failure and found no difference between immediate and delayed reconstruction but noted that the flap failure rate decreased over time (2.7% in 2015 and 1.2% in 2020).

#### 3.4.2. Question 2b: Studies Comparing Delayed Versus Immediate Reconstruction and Timing of Radiotherapy

Five studies [16,67,68,102,103] examined the timing of RT in relation to the timing of reconstruction. Complications differ between implants (2 studies) and autologous reconstruction (3 studies), and therefore, the use of RT needs to be analyzed separately for these two methods. The MROC study has separate analyses in different publications for implants (Yoon et al. [68]) and autologous reconstruction (Billing et al. [67]).

Ulrikh et al. [102] compared implants in patients receiving PMRT (single stage then PMRT, 2-stage with PMRT to the expander, or PMRT then delayed implants) and found similar rates of grade I/II infection and seroma but 9.5% versus 17.6% versus 20.4% grade III complications and 10.8% versus 19.1% versus 17.3% reconstructive failure. Immediate reconstruction with a single-stage implant had fewer complications, except for capsular contracture, which was higher (15.2% vs. 2.9% vs. 2.0%).

In the MROC study [68], 80 patients who received PMRT after the expander/implant exchange were compared with 237 patients who received PMRT to the expander (prior to the exchange). There was no significant difference in overall or major complications or failure. Anxiety, depression, and fatigue at 2 years were reported to be statistically better in the group with PMRT to the implant, though differences were <MID of the Patient-Reported Outcomes Measurement Information System (PROMIS) scales. There were no differences in BREAST-Q, European Organisation for Research and Treatment of Cancer (EORTC), and other PROMIS scales.

A report of the MROC study compared mastectomy and immediate autologous reconstruction followed by PMRT versus mastectomy, then PMRT, then delayed reconstruction [67]. This study is only partially prospective, as the delayed group was recruited subsequent to mastectomy, and the delay between mastectomy and reconstruction was not reported. There were only 108 immediate and 67 delayed reconstructions. Due to the small number of patients, we considered there to be insufficient events for logistic regression of individual complications; the difference between immediate and delayed groups for any breast complication was not significant (OR = 0.64, *p* = 0.442 at 1 year; OR = 1.14, *p* = 0.848 at 2 years). Prior to reconstruction (before mastectomy in immediate group but after mastectomy for delayed group), the immediate group had better Satisfaction with Breasts (59.5 vs. 36.3, *p* < 0.001), Psychosocial Well-being (66.1 vs. 50.0, *p* < 0.001), and Sexual Well-being (52.1 vs. 29.8, *p* < 0.001) and differences are likely due to presence/absence of breasts; Physical Well-Being (abdominal) was 87.3 vs. 82.9 (*p* = 0.058). The large differences in baseline PROs are likely not related to reconstruction (but rather the absence of reconstruction in the delayed group). After reconstruction, there were no significant differences in PROs at 1 year and only differences in Physical Well-Being (chest and upper body) at 2 years. In this comparison, *p* values at 1 and 2 years were adjusted for baseline (but not pre-mastectomy PROs) and covariates; mean values but not preoperative values were adjusted for covariates.

A variation of immediate reconstruction, termed staged or delayed-immediate or immediate-delayed, uses an expander or temporary implant after SSM or NSM to maintain the skin flap and provides a breast mound instead of a flat chest while awaiting reconstruction. This allows time for recovery prior to reconstruction or while awaiting final pathology or other information that determines the need for PMRT. It has also been used on the assumption that PMRT to an expander is preferable to irradiation of the final implant or autologous flap.

A propensity-matched study (age, BMI, comorbidities) by Christopher et al. [16] used staged/delayed-immediate reconstruction (tissue expander → PMRT → autologous reconstruction) versus immediate reconstruction followed by PMRT. It found fewer complications (fat necrosis, skin necrosis, additional office visits and outpatient surgeries, revision surgery) due to reconstruction in the staged group but additional complications due to the expander and RT steps (24/28 complications including infection, wound dehiscence, expander exposure, and failure to expand occurred during or after RT but before reconstruction). Revision surgery (primarily for asymmetry) was higher in the immediate group (42.4% vs. 12.1%, *p* < 0.001).

Hassan et al. [103] compared staged (skin-sparing; immediate expander/spacer, then reconstruction) to delayed microvascular (autologous) reconstruction. PMRT, when used, was to the expander in the staged group and prior to reconstruction in the delayed group. Expander had either prepectoral or subpectoral placement, and results do not make a distinction. While the publication implies the spacer/expander was placed pending pathology results and would be replaced as soon as possible if PMRT was not needed, the results indicate a median of 8 months (interquartile range 3 to 16 months) in the staged group and 15 months (9 to 30 months) in the delayed group. In patients with delayed reconstruction, those with PMRT had sooner reconstruction than those without (median 12 months vs. 15 months), suggesting there may be differences in baseline characteristics. The publication does not indicate whether complications were measured after reconstruction, mastectomy or combined, but these were generally similar except for seroma (higher in the delayed group without PMRT) and infection (higher in a delayed group with PMRT). Cosmetic outcomes were not assessed. Differences in PROs were not statistically significant; however, PROs were only measured at the end of the study and not a specific time after surgery and, therefore, are generally a long-term assessment. Factors used in the multivariate model were not reported, and the discussion suggests that baseline differences may confound the complication rates.

#### 3.4.3. Interval Between Radiotherapy and Further Reconstruction

During the systematic review process, an ancillary question arose regarding how long after PMRT the surgeon should wait before performing delayed autologous reconstruction or expander-implant exchange. While this was not a specific question in the review protocol, relevant studies were noted. Studies included for other questions offer guidance as to current practice. They were consistent in waiting at least 3 months to avoid the period of acute radiation injury for both delayed autologous reconstruction [16,96,99] and for expander-implant exchange after PMRT to the expander [104,105,106,107]. While 3 months is considered an absolute minimum, and 3 to 6 months is sometimes acceptable [16,104], a minimum of 6 months was more commonly used [99,106,107,108]. A review by Koesters et al. [109] suggests that instead of an arbitrary time, surgeons may use clinical assessment of the skin quality and laxity to avoid operating on tissue that is acutely tight, inflamed and predisposed to complications.

### 3.5. Question 3: Nipple-Sparing Mastectomy Issues

#### 3.5.1. Question 3a: NSM and SSM

Nine studies (10 publications) comparing NSM and SSM are summarized in Table S5 [110,111,112,113,114,115,116,117,118,119]. Two of these [110,111,112] identified patients for NSM, and then those with involved or close margins on frozen section analysis had the nipple-areolar complex (NAC) removed and, therefore, considered as having SSM. Groups are expected to be similar as all patients met the same criteria until the time of mastectomy. There were no significant differences in complications or cancer-related outcomes. Cho et al. [118] removed the nipple if positive retroareolar margins were found in the frozen-section biopsy of retroareolar tissue and excluded these patients from the NSM group. They used both propensity-score matching and Kaplan-Meier and Cox proportional hazard regression to compare NSM and total/SSM and found no differences in 5-year DFS, OS, or local recurrence-free survival. When they applied their model to the SEER dataset, they found NSM had better OS, likely due to younger patients with less advanced cancer in the SEER set.

A fourth study [113] offered NSM to 187 patients with stage IIIa cancer with a clinically normal nipple and no skin involvement; the use of preoperative imaging was not mentioned. Thirty-five patients were found to have cancer involvement on subareolar frozen section biopsy and were converted to SSM, while 333 patients were preoperatively assigned to SSM. Preoperative criteria for NSM and SSM were the same, so SSM may have been by patient choice, but this is not stated explicitly. After adjustment for patient and tumour characteristics, there was no difference in DFS (HR = 1.004, *p* = 0.991) or OS (HR = 0.866, *p* = 0.813). These four studies suggest that both NSM and SSM have similar oncologic results when pathologic analysis of retroareolar/excised tissue does not show tumour involvement. This is explored more in 3b and 3c.

The other five studies [114,115,116,117,119] did not report on surgical methods or whether pathologic analysis of resected tissue was conducted. Kelly et al. [115] reported unadjusted data that indicated NSM required less aesthetic revision operations (13.8% vs. 32.6%, *p* < 0.001) and resulted in better BREAST-Q scores on Psychosocial Well-Being and Sexual Well-Being and some components of Satisfaction with Breasts. They included only the outcome of Dissatisfaction with Breasts in multivariate analysis and found no difference between groups. Racz et al. [116] also found better scores on Sexual Well-Being for the NSM group, and this was significant on multivariable analysis, whereas there were no differences in Satisfaction with Breasts and Psychosocial Well-Being scales. They did find that nipple reconstruction and/or tattooing modified the Sexual Well-Being scores; NSM versus SSM without these was better (*p* = 0.008), but NSM and SSM were similar if nipple reconstruction/tattooing was used (*p* = 0.182). Wang et al. [114] reported that the rate of any complication (initial or adjusted data) was higher in the NSM group, but they included nipple ischemia/eschar/necrosis, which occurred in 16.5% of NSM patients and made up a large portion complications but by definition of SSM could not occur in SSM. Most other complications (unadjusted values) were higher in the SSM group. They did not subdivide by major or total complications but noted that only 2 of 36 patients with nipple complications required nipple removal. Reoperation rates within 90 days were not significantly different in multivariate analysis (*p* = 0.695).

Two studies were conducted in Japan. Ogiya et al. [117] found 7-year local recurrence (LR) of 2.6% NSM versus 3.8% SSM in patients with non-invasive cancer and 6.6% versus 4.8% for invasive cancer. Multivariate analysis only compared results to total mastectomy. Of NSM with recurrence, 13.7% involved the nipple, which is high compared with most other studies. Sasada et al. [119] looked at the impact of RT in patients with involved surgical margins. They found involved surgical margins in 8.6% of NSM, including 1.8% of cases with involved nipple margins, while 6.4% of SSM cases had involved margins. In patients with involved margins and PMRT, LR was compared with patients with total mastectomy; in NSM, they reported HR = 1.26 (*p* = 0.696) and in SSM, HR = 0.65 (*p* = 0.515).

#### 3.5.2. Questions 3b and 3c: Patient Selection and Surgical Factors

Patient selection and surgical factors in NSM were often dealt with in the same studies, and therefore, these were combined during data extraction. Table S6 summarizes 79 studies in 85 publications [6,7,8,12,106,112,113,120,121,122,123,124,125,126,127,128,129,130,131,132,133,134,135,136,137,138,139,140,141,142,143,144,145,146,147,148,149,150,151,152,153,154,155,156,157,158,159,160,161,162,163,164,165,166,167,168,169,170,171,172,173,174,175,176,177,178,179,180,181,182,183,184,185,186,187,188,189,190,191,192,193,194,195,196,197]. These include six studies [6,120,121,122,123,124] comparing tumour-to-nipple distance (TND), while most of the others are non-comparative. Two studies from Question 3a [112,113] are of relevance.

No RCTs were found. Studies were conducted mostly in the USA (20 studies), Italy (19 studies), Korea (15 studies), and Japan (6 studies), with 11 other countries contributing 20 studies. Most were non-comparative studies that met the criteria of having at least 100 patients and had details on patient selection or surgical methods.

#### 3.5.3. Effect of Tumour-to-Nipple Distance

Six studies compared the effect of TND on outcomes [6,120,121,122,123,124]. One study [6] calculated TND using magnetic resonance imaging (MRI), ultrasound, or mammography, while another used these three imaging techniques but did not specify how TND was calculated [124]. The other studies used MRI to measure TND. Cutoffs of 1 cm, 2 cm, or 5 cm were compared. Studies generally excluded patients with preoperative evidence of NAC involvement and conducted intraoperative/frozen section pathology analysis of the nipple base; patients with nipple involvement had the NAC or nipple removed. Differences in oncologic outcomes for TND < 2 cm versus > 2 cm did not appear to favor either group; these were small and were not statistically significant [120,121,122,124]. Similarly, there were no differences when comparing TND ≤ 2 cm, 2 to 5 cm, or >5 cm [123]. Wu et al. [6] used propensity-score matching to adjust for age at diagnosis, T stage, N stage, molecular subtype, and RT status in the comparison of TND ≤ 1 cm versus >1 cm. This study was larger than the other and had 495 patients per group. There were no significant differences in local or regional recurrence or distant metastasis at 5 years, nor in 10-year local recurrence-free survival or DFS. These studies suggest that for patients without preoperative evidence of NAC involvement, as long as frozen section and permanent pathology are conducted and are negative (or NAC excised in positive cases), TND < 2 cm or <1 cm does not predict worse recurrence or survival. MRI may give information about the probability of NAC involvement as this increases with decreased TND, raise suspicion if frozen section pathology is negative, and influence surgical strategy [198]. As noted in subsequent sections, frozen section analysis is not used at some institutions or is used selectively, and permanent/final pathology alone may be used.

#### 3.5.4. Recurrence, Survival, and Necrosis

A summary of recurrence, survival, and necrosis rates is provided in Table 1. These studies report on approximately 30,500 patients, although there may be some overlap in multiple publications from the same institutions.

Oncologic outcomes reported in the studies varied, though LR and LR in the NAC were most common and reported in 40 and 39 of the studies, respectively. As expected, recurrence was higher in subgroups with higher-stage cancer. Direct comparison between studies is difficult due to differences in follow-up times, cancer stage and other disease and patient characteristics, adjuvant treatment, and whether data are reported on a per-patient or per-breast reconstruction basis.

Two studies compared outcomes in patients with and without NACT (which is correlated to higher stage); LR was 2.8% and 2.0% in patients without NACT and 9.8% and 7.0% in patients with NACT [141,153]. Two additional studies were conducted in patients receiving NACT [158,159] and reported higher rates of distant metastasis (DM; 11.9% and 19.6%) than most studies (median 4.3%). A study in patients with locally advanced breast cancer (77% received NACT) [168] reported higher rates of recurrence and lower survival (LR 7.2%, 5-year DFS 70%) than most studies. One other study, by Benediktsson et al. [127], had much worse recurrence (locoregional recurrence [LRR] 16.2%) and survival (5-year DFS 68%, 10-year DFS 60%) results than most of the others. Inclusion criteria were patients with multifocal disease or tumours > 3 cm in size, suggesting that patients may have been at higher risk; the authors also mentioned that many patients for whom RT would be standard practice by the end of the study did not receive RT. In the subgroup with RT, LRR was 8.5%, while it was 28.4% in patients without RT. Median follow-up was 13 years (much longer than most) and may contribute to higher rates of LRR. Results excluding the two studies with only NACT and the other two studies mentioned above are summarized in Table S7. Median values were 3.4% LR, 3.6% DM, 96.7% 5-year OS, and 92.0% 5-year DFS. Of particular relevance is that the rate of LR in the NAC is very low (median 0.5%, average 1.2%).

Of the 42 studies that reported recurrence rates in the NAC, 17 reported none (8 from the USA, 6 from Italy, 1 from Brazil, 1 from Germany, 1 from Canada) and an additional 8 reported < 1% recurrence in the NAC. Only one study from China, two studies from Japan, and five from Korea (4 from the same group) reported > 2% recurrence in the NAC. Wu et al. [155,156,157] reported that when LRR occurred, 5-year post-recurrence DFS was higher for NAC recurrence (89.1%) than when recurrence was in the skin/chest wall (73%) or regional (59.4%).

#### 3.5.5. Total Skin-Sparing Mastectomy

Five studies from the USA (four from the same group at the University of California [106,166,167,168] and one from the University of Arkansas for Medical Sciences [128]) reported that no NAC recurrence occurred when using total skin-sparing mastectomy (TSSM). Boneti et al. [128] conducted complete removal of the glandular NAC while preserving the skin and refer to this as TSSM; they suggest the term NSM is confusing, and it is actually nipple-skin-sparing mastectomy. Sbitany et al. of the University of California group indicate that TSSM uses more aggressive surgical dissection relative to NSM and greater reduction in vascularity of the NAC [199]. Reduction in nipple perfusion after TSSM is greater than after NSM, and single-stage reconstruction should be avoided to minimize the risk of NAC necrosis. This aggressive surgery may account for the absence of recurrence in the NAC in these studies. Other studies with NSM may use similar or less aggressive surgery (leaving more NAC tissue), and differences may contribute to the range of both NAC recurrence and NAC necrosis observed in this review.

#### 3.5.6. Incision Location and Other Risk Factors

Five studies focused on incision type [169,171,181,182,186], while four others focused on factors affecting necrosis [177,184,187,197]. Park et al. reported complete nipple necrosis of 21.3% periareolar, 11.3% radial, and 3.4% inframammary fold (IMF) incision [181]. Multivariate analysis found risk factors were periareolar incision (vs. IMF; OR = 3.628, 95% CI = 1.596 to 8.250, *p* = 0.002); TND (OR = 0.712, 95% CI = 0.546 to 0.927, *p* = 0.012); breast weight (OR = 1.002, 95% CI = 1.000 to 1.004, *p* = 0.014). Seki et al. also found higher rates of complete nipple necrosis with periareolar incisions than for IMF incisions (3.3% vs. 0) [182]. Salibian et al. found higher rates of major ischemic complications (including major mastectomy flap necrosis and/or full NAC necrosis requiring debridement) with IMF incisions (25%) and inverted-T incisions (36.1%) than vertical (5.8%) or lateral radial (7.8%) incisions [171]. In multivariate analysis, inframammary (OR = 4.382) and inverted T incisions (OR = 3.952) were independently associated with an increased risk of major ischemic complication. Cavalcante et al. [186] compared IMF and periareolar incisions and reported complications in 15.3% versus 35% (*p* = 0.0002, OR = 0.33, 95% CI = 0.14 to 0.79). NAC necrosis was a large portion of complications and occurred in 8.5% versus 22.4% of patients (aOR = 0.34, 95% CI = 0.13 to 0.88, *p* = 0.025). Moo et al. [187] reported necrosis by incision type as follows: 12.1% lateral IMF (OR = 0.41, aOR = 0.35); 19.0% central IMF (OR = 0.64, aOR = 0.54); 29.8% lateral radial (OR = 1.0 as reference); 37.3% inferior periareolar/lateral extension (OR = 1.25, aOR = 1.24); and 41.2% superior periareolar/lateral extension (OR = 1.38, aOR = 1.59). They also found necrosis was higher with specimen (mastectomy tissue) weight > 400 g, fill volume > 200 mL, a subpectoral expander, and surgeons with less experience.

Braun et al. [197] found rates of necrosis varied with incision type: 12.4% IMF incision versus 21.2% radial (*p* = 0.1). Larger mastectomy weight, implant weight, and implant volume were also risk factors. NAC necrosis rates were higher in the retrospective set of patients (15.1%) than in the prospective set (6.7%); the latter used more IMF incisions, prepectoral implants, and SPY (a fluorescence imaging platform using indocyanine green [ICG] angiography).

Lai et al. [184] found that periareolar incisions had a higher risk of NAC ischemia or necrosis compared with upper outer radial incisions (OR = 5.33, *p* < 0.001) [184]. In multivariate analysis, Ahn et al. [177] found periareolar incision was a predictor of NAC necrosis (OR = 4.281, *p* < 0.0015) and indicated that periareolar incision is known to decrease blood flow to the NAC. Holland et al. [169] studied patients with prepectoral reconstruction and reported NAC complications in 25% with superior periareolar incision and 7.4% with IMF incision (*p* = 0.033); in multivariable regression, a periareolar incision was associated with increased odds of nipple necrosis (OR = 3.6, *p* = 0.018).

Lin et al. [191] reported on lessons learned in 3035 NSM with immediate implants. Relative rates of nipple necrosis by incision type were inferolateral IMF (reference, 1.0), horizontal radial OR = 3.823 (95% CI = 1.081 to 13.515, *p* = 0.037); vertical inferior OR = 2.124 (95% CI = 0.453 to 9.944, *p* = 0.339); periareolar OR = 14.235, (95% CI = 6.248 to 32.435, *p* < 0.001); and extension of prior incision OR = 2.98 (95% CI = 0.657 to 13.511, *p* = 0.157). Preoperative RT and smoking were also risk factors for nipple necrosis. Complications, including nipple necrosis (1.9% vs. 0.88%), infection (4.2% vs. 2.8%), and explantation (5.1% vs. 3.5%) were higher with expanders than direct-to-implant reconstruction.

#### 3.5.7. Other Factors Affecting Necrosis Rates

While there was no direct comparison, several authors commented on the effect of skin tension on necrosis. The viability of the NAC depends on the preservation of the blood supply to the nipple, ducts, and surrounding skin [132]. Immediate fixed-volume implants may put more tension on the skin flaps than tissue expanders that are gradually inflated. A systematic review by Robertson et al. [200] suggests that skin tension should be minimized to reduce necrosis.

#### 3.5.8. Studies at the University of California

Surgeons at the University of California began performing TSSM in 2001, and it became their procedure of choice [166]. Five studies [106,166,167,168,169] reported serial improvements to reduce ischemic complications, including nipple necrosis, while still ensuring low rates of recurrence. The initial revision was to eliminate the use of periareolar incisions; in most patients, IMF incisions or superior areolar/mastopexy incisions with less than one-third of NAC circumference were used. For implants, they switched to the use of two-stage reconstruction (expander-implant) and used minimal (50 to 100 mL) intraoperative expander fill. With these changes, nipple necrosis decreased from 13% to 1.8%. They also extended the patient criteria to allow those with tumours under the NAC if not directly involving the nipple itself, tumours with skin involvement that respond well to NACT, patients with previous (>6 months) circumareolar incisions for breast reduction or augmentation, and (using a superior areolar/mastopexy incision) patients with moderate ptosis.

For oncologic control, they evolved to allow TSSM even if tumours were close (<1 cm) to the NAC provided that MRI (used only for tumours close to the nipple by clinical examination or mammography) demonstrated no clear involvement of the NAC. They examine serial sections of the removed tissue at final pathologic analysis. If final pathology detected a tumour in the nipple tissue specimen, they conducted repeat excision or NAC removal at the time of expander-implant exchange or autologous flap revision, or RT alone without further surgery. Tumours in or near the nipple skin are treated in the same way as other positive skin margins using repeat excision, resection of the involved skin, or PMRT. Repeat excision is used to ensure the removal of residual ductal tissue or residual tumour, although all these specimens had only fibrous tissue and scar without residual ductal tissue or cancer. In cases of invasive cancer in the nipple specimen, NAC excision is usual, but they will attempt NAC preservation using repeat excision in patients with a strong desire to retain the NAC. In the 2012 publication [166], there were 20 nipple specimens containing tumours; 16 had re-excision or NAC removal, and all were negative for residual tumours.

Their next publication further discusses the evolution of the technique and provides more outcome results [106]. The interval between PMRT and expander-implant exchange was increased from 3 months to 6 months. The study overlaps with the previous publication but contains 32 cases of cancer on final pathology (15 in situ and 17 invasive). Of the in situ cases, 2 had nipple skin resection, 3 PMRT and 10 no additional treatment. Of the invasive cases, 10 had resection of the nipple skin, 5 PMRT alone, and 2 had no treatment because margins were not involved. Of the cases without resection, there was no LRR in the NAC skin, although follow-up was only a median of 22 months. No mention is made regarding the pathological status of resected tissue. Yearly data are provided, and there is wide variation in treatment characteristics and outcomes from year to year such that trends are not apparent. A subsequent publication by Amara et al. added an additional year of patient accrual [167]. While there were the same number of cases with nipple involvement, the treatment was not the same as reported previously; Amara et al. indicated 11 repeat excisions, 8 NAC removals, 5 PMRT, and 8 with no further treatment. This is further broken down by DCIS and invasive cancers: for DCIS, there were 4 re-excisions, 3 NAC removals, 2 PMRT, and 5 no treatment; for invasive cancer, there were 7 re-excisions, 5 NAC removal, 3 PMRT, and 3 with no treatment. All cases with no treatment had a tumour in the nipple without extension to the margins. There were no recurrences in the DCIS cases. In the invasive cases, there was 1 LR (not involving the NAC) in the re-excision group, 3 DM in the PMRT group, 1 LR/DM in the no-treatment group (not involving the NAC), and no recurrence in the NAC removal group. In patients with nipple involvement and PMRT, the PMRT was administered for other primary indications (e.g., tumour size or lymph node involvement). The use of complete NAC excision decreased over time. For nine margin excisions, five had a scar or fibrous tissue, and four had benign breast tissue; two additional cases are pending. This publication mentions that frozen section analysis is not performed due to potential false-negative results and a low rate of tumour involvement. At the time of writing (2015), the only indication for NAC removal was extensive tumour involvement of the subareolar margin and nipple specimen in final pathology. In the absence of PMRT treatment and pathology specimens showing definitive involvement of the anterior aspect of the excised nipple tissue, they performed a re-excision of the margin. In these cases, none of the re-excisions have shown invasive or in situ cancer. No patients have shown LR in the NAC.

The most recent publication included is by Holland et al. [169], studying a more recent patient population undergoing prepectoral reconstruction. They compared superior periareolar incisions (about 50% of circumference based on the photos, and thus exceeding the guideline of less than one-third in previous publications) to IMF incisions and found IMF incisions resulted in fewer NAC complications and nipple necrosis requiring debridement. As a result, they have moved away from superior periareolar incisions if the patient is a candidate for IMF incisions; for patients (such as with grade II ptosis) who require a superior periareolar approach, they have added a lateral extension.

#### 3.5.9. Nipple Sensation and Erection

For question 3c, nipple sensation and erection were among the outcomes of interest. These were not reported in most studies. Fortunato et al. [129] noted that 8% of patients had some NAC sensation in the first month after surgery and 17% at 6 months; this was generally described as minimal or partial. Rossi et al. [132] found nipple sensitivity and erectile capacity were insufficient in most patients. Petit et al. [160] compared patient feeling in the areola when touched to that in the contralateral areola without surgery. Using a scale of 0 to 10 and groupings of 0 to 3 as poor, 5 to 6 as good, and 7 to 10 as excellent, they found the average value for sensitivity was 2. At least partial sensitivity of the NAC was recovered in only 15% of patients 1 year after the operation. In the study by Stanec et al. [131], NAC sensation was evaluated at every postoperative physical examination and again before the end of the study. While most had > 1 year follow-up, the median follow-up was 63 months. The influence of the length of follow-up on sensation recovery is not reported. The patients scored the degree of sensation to light touching compared with the non-operated breast on a scale of 0 to 4 (0 being no sensation, 1 for more decrease, 2 for less decrease, 4 for normal sensation). At the final assessment, they found that 16% had no sensation, 27% more decrease (large decrease), 35% less decrease, and 22% had normal sensation. While all these studies indicate NAC sensation is low after NSM, this was not the focus of the studies; they did not explore contributing factors or ways to improve this, and measurement/evaluation was either not reported or suboptimal.

A prospective study by Black et al., specifically on breast sensation after NSM, was conducted at Weill Cornell Medical College in New York City, New York [189]. They included patients with immediate expander-implant reconstruction (64 prepectoral and 22 subpectoral) or autologous reconstruction with neurotized DIEP flaps (106 patients). The autologous group had more hypertension, diabetes, and higher BMI (mean 26.9 vs. 23.8 kg/m^2^) than the implant group. Sensation was evaluated using a pressure-specified sensory device and measured at nine regions per breast (NAC, and outer and inner superior, medial, inferior, and lateral regions) at predefined timepoints along the clinical course, both before and after mastectomy/reconstruction. The mean preoperative sensation threshold was 14.1 g/mm^2^ in the NAC and 16.5 to 20.0 g/mm^2^ in other locations. Short-term (<1 year) and long-term (≥4 years) post-operation, the autologous group had sensation thresholds of 67.4 and 38.5 g/mm^2^ at the NAC, 62.2 to 73.2 and 37.5 to 40.8 g/mm^2^ in the inner quadrants and outer inferior quadrant, and 42.5 to 56.2 and 25.9 to 32.0 g/mm^2^ in the other outer quadrants. The sensation threshold was worse in the patients with implant reconstruction: at <1 and ≥4 years post-operation, sensation thresholds were 79.2 and 45.2 g/mm^2^ at the NAC, 72.3 to 79.1 and 50.9 to 66.2 g/mm^2^ in the inner quadrants and outer inferior quadrant, and 48.9 to 61.6 and 35.2 to 48.0 g/mm^2^ in the other outer quadrants. This study did not report surgical details such as incision type, although an earlier study by this group used inframammary incisions for NSM [201]. Authors suggest that while neurotization of the flap likely contributes to better sensation compared with implants, better sensation is also expected (based on other studies) with non-neurotized flaps and that randomized studies are needed.

#### 3.5.10. Treatment of Patients with Tumour Detected in Resection Specimens

Patients with subareolar resection specimens with tumours detected were most commonly treated with NAC resection. When subareolar samples were involved by tumour on interoperation/frozen-section pathology, the most common treatment was NAC resection during the same operation. Some more recent studies removed the nipple but retained the areola (e.g., Fregatti et al. [123]; Smith et al. [139]; Tang et al. [138]; Shanno et al. [193]), or excised only the portion of the areolar with involved margins. Tang et al. [138] reported full NAC excision decreased over time, with only the nipple removed in 38% of cases from 2007 to 2011 and 62% of cases from 2012 to 2014. Shanno et al. [193] also reported 50% only nipple removal in 2009 to 2015 and 89% nipple-only excision in 2016 to 2019. Three of these studies were conducted at the same institution. Some studies considered LCIS or atypical hyperplasia to be negative results and did not result in NAC excision. Shanno et al. [193] included 134 patients with atypia without a nipple or NAC excision, and none developed recurrence in the NAC or periareolar skin. In some cases, further dissection, either as confirmation or in an attempt to obtain clear margins, was conducted. When NAC involvement is suspected based on the final pathology of subareolar resection specimens, resection of the nipple or NAC is usual practice; however, RT or no treatment is sometimes used. Of studies where nipple or NAC was excised and underwent pathologic analysis, rates of involvement were variable (0 to 100%): Fregatti et al. [123] 63.6%; Fortunato et al. [129] 58%; Rossi et al. [132] 100% (17% invasive, 72% in situ, 11% LIN III); Santoro et al. [133] 52%; Agresti et al. [141] 41%; Cont et al. [142] 45%; Warren Peled et al. [166] 0%; Amara et al. [167] 0%; Shanno et al. [193] 24%; Spoor et al. [196] 59.5%; and Zhu et al. 9.1% [7]. Possible factors are patient selection and imaging used, the extent of excision of breast tissue, how margins and orientation of samples were marked, and the use of re-excision prior to eventual NAC removal.

In patients with tumours on pathology but no nipple excision, Spoor et al. [196] found no recurrence in 20/20 patients. Petit et al. [160] used ELIOT (intraoperative RT) or delayed RT in patients with NSM. Intraoperative frozen section analysis was performed, and the margin was further revised if a tumour was present in the initial sample. NAC was excised in patients with both samples containing tumours or with poor blood supply and high risk of necrosis, which contraindicated RT. Of the patients with NSM and RT, 86 cases had tumours on final pathology, and in 79 cases, only the first but not second frozen section sample was involved; no recurrence in the preserved NAC was found. It is noted that the median follow-up was only 20 months. A study by the same group (Galimberti et al. [163]) with at least 5 years follow-up (median 94 months) and likely involving some of the same patients reported 1.8% NAC recurrence, and no statistically significant difference between patients with ELIOT/IORT to the NAC and those without (although the group without RT is small). They did not report whether cases with tumours on final pathology were included.

Warren Peled et al. [166] used only permanent pathology analysis and repeated excision for tumours near or in the nipple skin (all results were negative) or used PMRT. Cases with invasive cancer in the nipple had repeat excision if patients were highly motivated to preserve the NAC (7 cases) or NAC removal (9 cases) or PMRT (4 cases); all 16 patients with re-excision or NAC removal had no residual tumour. There were no NAC recurrences with a median follow-up of 28 months. Similar results by the same group were reported by Wang et al. [106]. Of 32 cases with a tumour on final pathology, 12 had nipple skin resection, 8 had PMRT, and 12 had no treatment; there were no recurrences in the NAC skin. Another paper from this group by Amara et al. [167] had 32 breasts with positive margin or nipple tissue, of which 11 had repeat incision, 5 had PMRT, 8 had NAC removal, and 8 had no further treatment; they also reported no recurrence in the preserved NAC skin.

Shanno et al. [193] found no significant difference in periareolar recurrence for patients with nipple excision compared with those with NAC excision. For patients with tumour in the nipple margin (excluding 4 patients without <1 year of follow-up), 64 had nipple excision, 34 had NAC excision, 15 had no excision (9 clear margins, 2 planned RT, 2 small amounts of cancer, 2 only lymphovascular invasion). There were 2 recurrences as subareolar nodules, both in patients with initial nipple-only excision.

#### 3.5.11. Intraoperative Frozen Section Versus Definitive/Final Pathology

While some of the above studies compared the assessment of resected subareolar tissue by frozen section or permanent/final pathology, it was not the focus of any of the studies. Most studies that gave details of sampling and pathology analysis used intraoperative/frozen-section analysis and based decisions to excise the NAC on these results. However, some major institutions have implemented policies to only use final/definitive pathology.

Fregatti et al. [123] used a definitive pathological examination of the retroareolar margin of the excision or nipple duct bundle to determine whether to excise the nipple. They note that permanent section analysis is more accurate and can distinguish between benign atypia and intraductal carcinoma that would be false positive in frozen section, leading to unnecessary NAC excision, is more able to detect small foci of cancer that could be false negative on frozen sections, and allows time for consultation with the patient. Serio et al. [188] indicated that San Giovanni-Addolorata Hospital, Rome, Italy, has used only definitive pathology since 2016 and that intraoperative pathology may have freezing artifacts that interfere with diagnosis and false-positive and false-negative cases.

Massachusetts General Hospital (affiliated with Harvard Medical School; Tang et al. [138], Smith et al. [139], Webster et al. [151], Shanno et al. [193]) and the University of California (Warren Peled et al. [166,168], Wang et al. [106], Amara et al. [167], and Holland et al. [169]) appear to be leading centres in the field of breast reconstruction with many publications describing advancement of technique and clinical studies. They do not use frozen section analysis but instead do intensive ductal excision and base decisions on final pathology. The Massachusetts General Hospital group notes that practice is based in part on their earlier studies on techniques to ensure complete excision of duct tissue, such as those by Rusby et al. [202]. Both groups have many other relevant studies, although they did not meet the inclusion criteria.

Studies on this topic that do not meet our inclusion criteria may provide further information [203,204,205,206,207,208,209,210,211,212]. In general, frozen section analysis is specific but less sensitive. In one of the largest studies Alperovich et al. compared results for 307 breasts [209] and found the sensitivity of frozen section analysis to be 0.58 and the specificity to be 1. The high specificity may be in part because the pathologists were cautious about reporting equivocal frozen section analysis as positive due to the possibility of unneeded NAC resection. Of 20 positive subareolar biopsies, only 6 resected specimens (NAC) had evidence of diseases.

Hogan et al. [204] also found that of nine cases of NAC excision due to intraoperative findings, six were benign, and DCIS was found in three. A study by D’Alonzo et al. [206] evaluated the accuracy of sub-areolar/nipple tissue analysis in predicting NAC involvement. Compared with final pathology, 6% were false negatives (3 cases of DCIS), and 11.5% were false positives (3 cases of low-grade DCIS). They found that of 25 cases in which the frozen section was positive and the NAC was removed, only 10 had tumours in the NAC at conclusive pathology. The sensitivity of subareolar examination at predicting NAC involvement was 57.7%. The status of the margin facing the NAC, as well as the size and number of tumour foci on the subareolar tissue sample, were associated with NAC status in multivariate analysis. With no ink on tumour, the risk of NAC involvement was < 10%. Some studies use two biopsies, with the second being just beneath the retained NAC [213].

#### 3.5.12. Sensation

Sensation after mastectomy and reconstruction was identified as an important issue but not addressed sufficiently in the included primary studies. A brief narrative review of this topic is included in the extended version of the systematic review [22]. Careful identification of the nerves, particularly the fourth intercostal nerve, and avoiding damage during surgery is important in maintaining or restoring sensation to the NAC. Selection of the incision site is important in both the preservation of blood supply and minimizing nerve damage. A group of plastic surgeons from various institutions in the USA published a resource in 2024 to guide breast and plastic surgeons in mastectomy techniques that preserve nerves, including applied anatomy of breast innervation and steps to incorporate nerve-sparing mastectomy and breast neurotization [214].

### 3.6. Question 4: Implant Location

Results for Question 4 are summarized in Tables S8–S10. Table S8 contains 26 studies with prepectoral reconstruction compared with subpectoral or submuscular reconstruction, with the use of ADM similar in each arm [9,104,107,215,216,217,218,219,220,221,222,223,224,225,226,227,228,229,230,231,232,233,234,235,236,237]. All except four compare prepectoral versus dual-plane reconstruction. Table S9 consists of five studies where patients initially had subpectoral implants and, due to complications (in particular, animation deformity or pain), had conversion to prepectoral implants [238,239,240,241,242]. Table S10 reports five studies where the use of ADM is different in each arm, such as prepectoral with ADM versus subpectoral without ADM [108,232,243,244,245]. It is difficult to interpret whether differences in outcomes are due to the ADM or implant location. Results should be interpreted together with those of Question 5, which deals with ADM.

Of the 26 studies in Table S8, 3 were conducted in Italy, 2 in Korea, 2 in France, and the rest in the United States. There was a high proportion of contralateral prophylactic mastectomies in many of the studies. In the various studies, implants could be prepectoral (directly under the skin, with or without ADM), subpectoral (under the pectoralis major muscle) without specifying whether the lower pole is covered, submuscular (total coverage of implant beneath the pectoralis major and serratus anterior), dual-plane (upper portion under the pectoral muscle but with the lower portion only under the skin (ADM or other mesh used in most patients), or subpectoral and subfascial (under pectoralis major muscle and fascia of serratus anterior muscle). While we included only comparative studies, we included studies that compared any combination of these. As the terms submuscular and subpectoral are often used interchangeably, as well as subpectoral and dual-plane, we used the study methods (where available) to classify studies and only relied on these terms in the absence of surgical/reconstructive details. Initial breast size, implant size, skin flap circulation, patient and surgeon preference, and other patient factors often determined the location of the implant. Patients with poor perfusion or thin skin flaps, as assessed during surgery, were often placed in the non-prepectoral group. In some studies, patients with risk factors such as smoking or diabetes were also not considered for prepectoral implants. Multivariate or multivariable analysis was used in 10 studies, although often for only a subset of outcomes.

#### 3.6.1. Prepectoral Versus Submuscular

Two studies in Italy by members of the same group compared prepectoral foam-coated implants versus submuscular textured implants [215,216]. The final decision on the type of reconstruction was based on flap thickness and perfusion. ADM was not used. Complications and recurrence were similar, but the operating time was longer in the submuscular group. Franceschini et al. [215] found that the prepectoral groups had better aesthetics (excellent 65.6% vs. 11.3%), less chronic pain in the pectoral region (none or very mild 79.7% vs. 21.0), less shoulder dysfunction (4.7% vs. 40.3%), less contralateral operations for symmetry (3.6% vs. 100%), and more skin sensibility (48.4% vs. 29.0%). The study by Scardina et al. [216] was in patients who had received NACT; symmetrization procedures were required in 28% versus 82% of patients with unilateral mastectomy.

#### 3.6.2. Prepectoral Versus Subpectoral (Either Submuscular or Dual-Plane)

Two studies [217,218] compared prepectoral versus subpectoral (both submuscular and dual-plane used in the study). Darrach et al. [217] found that opioid use in the first 24 h was lower in the prepectoral group. Kraenzlin et al. [218] found higher rates of mastectomy flap and NAC necrosis, resulting in a higher rate of return to the operating room in the prepectoral group. Infection was lower in the prepectoral group but not significantly after adjusting for mastectomy weight. Hematoma was found in 2.0% versus 4.9% (*p* = 0.07) and cellulitis in 7.8% versus 12.5% (*p* = 0.09).

#### 3.6.3. Prepectoral Versus Dual-Plane

The most frequent comparison was prepectoral versus dual-plane and was made in 22 studies [9,104,107,219,220,221,222,223,224,225,226,227,228,229,230,231,232,233,234,235,236,237,245]. In dual-plane placement, ADM or other mesh was generally used to cover and support the lower half (lateral pole) of the expander or implant (the portion not under the pectoralis major muscle) and sutured to the lower border of the pectoralis muscle and in the area of the IMF. ADM was usually used with prepectoral implants to provide support for the lower pole and/or to provide an additional layer between the skin envelope and the implant. The expander or implant could be wrapped entirely with ADM (most common), the pocket after SSM/NSM lined entirely with ADM, or ADM used only on the anterior surface and posterior lower pole. There is an assumption that the development and use of ADM have made prepectoral (and, to a lesser extent, dual-plane) feasible. Most used ADM or other mesh in all patients, although Plachinski et al. [228] decided on prepectoral use depending on the quality of tissue coverage and surgeon preference and thus used it in 69.9% of prepectoral implants and 90.3% of subpectoral (lower pole coverage). Most also used ADM or mesh in all dual-plane implants, although Houvenaeghel et al. [232,245] used TIGR^®^ Matrix (Novus Scientific, Uppsala, Sweden) resorbable synthetic mesh in 54.3% of prepectoral patients and 1.7% of subpectoral patients. It is included here because they conducted regression analysis with ADM use as one of the factors. Some of the more recent studies found in the updated search used ADM in most prepectoral cases but only a portion (65% to 74%) of dual-plane cases [9,233,234,235].

Banuelos et al. [222] reported complications in obese patients (BMI ≥ 30 kg/m^2^) and used ADM in 93.7% of prepectoral cases and 93.2% of subpectoral cases. They found that while obesity increases complications and failure rates, these were similar in both groups and, therefore, is not a contraindication for consideration of prepectoral implants; additional care may be needed with BMI > 35 kg/m^2^. While methods indicated multivariate analysis was to be used, adjusted results were not reported. Asad et al. also studied the effect of obesity (BMI ≥ 30 kg/m^2^) and used ADM in 95.1% of prepectoral and 70% of subpectoral reconstructions [233]. Overall complications, explanations, and infections were lower in the prepectoral group.

Most studies found little or no significant differences in complications. Copeland-Halperin et al. [220] found lower postoperative pain, as measured by opioid use and refills in the prepectoral group. Bozzuto et al. [226] also measured postoperative pain in matched groups and using multivariate analysis and found significantly lower patient-reported pain and opioid use until discharge. Holland et al. [231] also found lower opioid use in the prepectoral group (*p* < 0.001, *p* = 0.048 multivariable analysis) and maximum patient-reported pain (*p* < 0.004, *p* = 0.001 multivariable analysis).

Avila et al. [221] found lower rates of ischemic complications of nipple necrosis, mastectomy flap necrosis, and nipple loss due to necrosis in the prepectoral group and no differences in infection, hematoma, seroma, implant loss/exchange in univariate analysis. A composite outcome of overall complications found rates of 5.91% versus 9.41% (*p* = 0.1842), with similar results after multivariate analysis (OR = 0.61, *p* = 0.190). Gabriel et al. [104] found in univariate analysis that the prepectoral group had lower rates of capsular contraction, infection, and seroma. Multivariate logistic regression was conducted on the outcome of any complication and found that the prepectoral group was better (14.7% vs. 25.8%, *p* = 0.030; *p* = 0.013). In contrast, Plachinski et al. [228] found no difference in major complications but higher rates of minor complications (21.7% vs. 7.8%) in the prepectoral group, mostly due to differences in seroma (20.5% vs. 4.9%). The prepectoral group had shorter hospital stays, fewer expansion visits, less animation deformity, and fewer prescriptions for muscle relaxants. Ribuffo et al. [229] found lower rates of any complication, seroma, or hematoma in the prepectoral group. They did not perform multivariate analysis, but patient characteristics were well-balanced. They also found lower animation deformity (0 vs. 68.7%) and better aesthetic results in the prepectoral group. Houvenaeghel et al. [245] found higher patient satisfaction and shorter duration of surgery for prepectoral implants.

The largest study was by Hung et al. [9]. They found higher rates of early complications with prepectoral reconstruction in the first (expander) stage in bivariate but not multivariate analysis and higher rates of late infection after 30 days (HR = 2.4, *p* = 0.01). There were no differences in second stage (implant) early complications but higher late infection rates (HR = 5.3, *p* = 0.03) with prepectoral implants. Houvenaeghel et al. [232] found higher rates of overall complications within 90 days for the prepectoral group, but after multivariate regression, there were no differences in overall or grade 2 to 3 complications. Asaad et al. [234] found no differences in complications; only 33.7% completed PROs, and therefore, the sample size was too small for PROs. Hassan et al. [235] found no significant differences. Min et al. [237] found that prepectoral reconstruction had less seroma and implant migration than dual-plane reconstruction.

#### 3.6.4. Conversion from Subpectoral to Prepectoral

Conversion to prepectoral placement in patients with previous subpectoral or dual-plane implants suffering from animation deformity or other implant-related issues was reported in five publications [238,239,240,241,242], summarized in Table S9. In these cases, patients would serve as their own controls for outcomes related to the presenting complaints. ADM was used for all of the implant revisions in three of the studies and 81.3% in the study by Holland et al. [241]. In the latest study by Salgarello et al. [242], ADM was only used in the first seven patients, while the remaining patients had polyurethane foam-coated implants. Publications by Sigalove et al. and by Gabriel et al. [238] include several of the same authors, and the patient population may overlap. In these two studies, surgical complications were 3.9% and 3.2%. In the study by Sigalove et al. [239], most patients had animation deformity (99.2%), pain (99.2%), and asymmetry (96%), sometimes accompanied by implant malposition (68.5%), capsular contracture (16.9%) or rippling (1.6%). All presenting complaints were resolved and did not recur. In Gabriel et al. [238], 94.1% of patients had animation deformity, and 89.2% had pain; after revision to prepectoral placement, all animation deformity was resolved; pain was not measured, but no patients complained. Complaints were animation deformity, implant distortion, and tightness in the study by Jones et al. [240]. After revision surgery, all animation deformity was resolved, and most patients had an improved range of shoulder movement; there was also an improvement in overall breast aesthetics, including better cleavage appearance. Minor contour deformity and rippling were treated with fat grafting in 18.3% of patients. Revision surgery complications included 4.2% infection, 2.1% seroma, and 0.7% hematoma, dehiscence, partial necrosis, and explanation. No capsular contraction occurred within the 44 months of follow-up, and authors attributed this to the use of ADM and a biofilm reduction protocol. Holland et al. [241] included patients with animation deformity, and this was resolved in all patients. This study had higher rates of complications, with 2.5% requiring reoperation and 13.8% treated for infections but no reconstructive failures. Asymmetry occurred in 21.25% of patients and was lower when ADM was used (15.4% vs. 47.0%). Similarly, capsular contraction occurred in 6.25% of patients (1.5% with ADM and 26.7% without). Preoperative fat grafting was used in patients with <1 cm of subcutaneous tissue before the prepectoral conversion operation (52.5% of patients). These patients had less asymmetry, capsular contraction, and cosmetic revision surgery. Salgarello et al. [242] conducted a cosmetic evaluation and reported high scores (2/2) in most patients. Mild capsular contracture occurred in most patients (47.1% Baker grade 1a, 42.5% grade 1b, 10.4% grade 2), and pain resolved in all patients. All measures on the BREAST Q also improved (values of 23 to 40 prior to replacement increased to 83 to 96).

#### 3.6.5. Studies Where Plane of Implant and ADM Use Are Both Varied

Five studies compared prepectoral to submuscular or subpectoral (dual-plane) placement of implants in which ADM use was different in each arm [108,232,243,244,245]. These studies are summarized in Table S10. Except for the second Houvengaeghel publication [232], mesh or ADM use was 0% versus 100% and, therefore, cannot be adjusted for in matching or multivariate analysis. Comparison, therefore, is only to a system of reconstruction such as prepectoral with ADM versus subpectoral without ADM; the relative importance of ADM compared with the plane of implant cannot be determined. These studies could also be considered as part of Question 5 on ADM use, with the same limitations.

Bettinger, 2017 [108] found complications to be 13.33% prepectoral with ADM versus 6.49% submuscular without ADM. Adjusted relative risk is given compared with a third arm, but it appears that the difference is not significant. Talwar, 2024 [243] compared prepectoral (96.6% had ADM) versus submuscular (4.8% had ADM) and found the prepectoral group had more surgical site infection, seroma, expander loss, and less mastectomy flap necrosis and NAC necrosis. They suggested that staging reconstruction to allow complete eradication of infection and proper wound healing, and vigilant sterile technique may be beneficial. The higher rate of gram-negative organisms may be related to prolonged drain use in the prepectoral group. Whether the higher rates of complications are due to implant plane or use of ADM is unknown; ADM used were non-sterile forms.

Houvenaeghel et al. [245] used TIGR Matrix in 86.6% of prepectoral and 0.9% of subpectoral patients. There were no significant differences in complications, and patient satisfaction was better in the prepectoral group. The duration of surgery was less in the prepectoral group. An additional analysis of the same study [232] with more patients indicated use of prepectoral reconstruction increased sharply over time. Use of mesh peaked at 92% for prepectoral implants in 2021 and decreased to 6.7% in 2023 due to reports of a negative impact on complication rate. Overall, mesh was used in 54.3% of prepectoral and 1.7% of subpectoral implants. Using multivariate regression, there were no differences in any complications, grade 2 to 3 complications, or patient satisfaction. Smoking, larger breasts (cup size > C), higher American Society of Anesthesiologists (ASA) status, mesh use, and areolar or inverted T incision increased complications while smoking, mesh use, greater mastectomy weight (>300 g), and diabetes increased grade II-III complications.

Chen et al. [244] took the opposite approach to most studies and used ADM or poly-4-hydroxybutyrate mesh (P4HB) in dual-plane implants but no ADM/mesh in prepectoral implants. A Cox proportional hazards model was used only for capsular contracture. Capsular contraction was higher in the P4HB group (HR = 1.60, *p* = 0.01 compared with no mesh), while dual-plane implants with ADM and prepectoral implants without ADM/mesh were similar (HR = 0.85, *p* = 0.38). Prepectoral and dual-plane with ADM had similar rates of necrosis, infection, and revision surgery, while necrosis and infection were lower with the P4HB mesh. Multivariate analysis was not used for these outcomes.

#### 3.6.6. Plane of Implants Summary

Overall, studies found that placing implants under muscle required slightly longer operations and resulted in higher levels of postoperative pain but similar surgical complications. Animation deformity is a persistent adverse effect that may occur with implants placed under muscle. Revision surgery to change implants from submuscular/subpectoral to prepectoral eliminated animation deformity and greatly reduced pain while improving cosmetic results and PROs. Surgical complications may depend more on surgical technique than the plane of the implant. Avila et al. [221] noted that the evolution of technique to preserve subdermal vascular supply, use of gentle retraction, and careful dissection in the supra-areolar region have improved results. As noted in Question 3, the size of the expander/implant and the rate of expansion may influence rates of complications. Subpectoral placement was often used if patients were judged not suitable for prepectoral implants, and therefore, it is difficult to assign outcomes specifically to the location of implants. Several studies have suggested that ADM use contributes to infection, and the next question should be looked at regarding this topic.

### 3.7. Question 5: Acellular Dermal Matrix

#### 3.7.1. Background

A list of some of the different ADMs mentioned in publications is provided in Table S11. Animal-derived products are generally used in Europe, while both human-derived and animal-derived products are used in the USA. Animal-derived products and non-absorbable synthetic mesh are not included in this review. AlloDerm^TM^ (LifeCell Corporation, Branchburg, NJ, USA) appears to be the most widely investigated and used human-based ADM in Canada and the USA; all studies included in this review in which the ADM was specified used AlloDerm in at least one arm of the study.

Until 2010, AlloDerm was provided as an aseptic freeze-dried form that needed to be rehydrated for 30 min prior to use [246]. FlexHD^®^ (Musculoskeletal Transplant Foundation [MTF], Edison, NJ, USA) is another aseptic product that is stored in alcohol instead of being freeze-dried [247,248]. AlloDerm RTU (ready to use) became available in 2010 and is terminally sterilized to a Sterility Assurance Level (SAL) of 10^−3^ and requires soaking for 2 min [249,250]. More recently, it has been renamed as AlloDerm SELECT^TM^ Regenerative Tissue Matrix (AlloDerm SELECT RTM or AlloDerm RTM) and is sold by Allergan Aesthetics, an AbbVie company [249,250].

Many studies do not specify the type of AlloDerm, and it can be assumed based on the year of patient enrollment. DermACELL^®^ (LifeNet Health, Virginia Beach, VA, USA), AlloMax^TM^ (Bard, Warwick, RI, USA), DermaMatrix^®^ (Synthes/Musculoskeletal Transplant Foundation, West Chester, PA, USA) and NeoForm^®^ (Mentor, now marked as AlloMax) are sterile products with a SAL of 10^−6^ [248,251]. ADM may be modified during manufacturing or by the end user. The regular or confluent form is unmodified. It may be perforated [252] by punching holes by the surgeon or more uniformly during production. ADM may be meshed by cutting slits that increase expansion and surface area [253,254,255,256,257] and improve fluid egress and presumably reduce seroma formation. Fenestration generally refers to cutting slits (especially staggered to allow expansion) but sometimes refers to perforating. Especially for prepectoral use where the entire expander or implant is wrapped, modifications allow better and neater fitting and sometimes allow smaller pieces of ADM, thus reducing cost. Products may be shaped by the user during surgery to fit the implant, such as the butterfly wrap [258], the wonton or ravioli technique [259,260], or Kirigami cutting [261].

#### 3.7.2. Overview of Results

Comparisons of human-derived ADM or synthetic mesh are reported in 59 publications summarized in Tables S12–S15 [10,11,13,14,69,70,105,191,192,193,232,244,245,246,262,263,264,265,266,267,268,269,270,271,272,273,274,275,276,277,278,279,280,281,282,283,284,285,286,287,288,289,290,291,292,293,294,295,296,297,298,299,300,301,302,303,304,305,306,307,308].

Two studies were conducted in Korea, one in Canada, the MROC study in the USA and Canada, and the rest in the USA. Additional publications with prepectoral implants in one arm were summarized in Question 4. Of the included publications, 30 (26 studies) in Table S12 addressed the use of ADM compared with no ADM [10,11,13,69,70,105,191,192,193,262,263,264,265,266,267,268,269,270,271,272,273,274,275,276,277,278,279,280,281,282,283]. Only two used the same surgery/implant position with and without ADM [105,274]. Twenty (15 studies) in Table S13 compared different ADMs [14,191,192,193,284,285,286,287,288,289,290,291,292,293,294,295,296,297,298,299,300], 8 in Table S14 investigated different treatments or forms of ADM [246,283,301,302,303,304,305,306], and 4 in Table S15 compared synthetic mesh to either ADM or none [232,244,245,307,308]. There were three RCTs (plus one terminated early), while the others were non-randomized comparative studies (4 prospective, 1 unclear, and the rest retrospective).

#### 3.7.3. ADM Versus No ADM—Studies with AlloDerm

AlloDerm use was compared with no ADM in 11 studies conducted in the USA [105,269,274,275,276,277,278,279,280,281,282] (see Table S12). The only RCT was terminated early due to slow accrual [280] with 70 patients; while the primary outcome was postoperative pain, the baseline pain levels were not the same. ADM was usually used with either subpectoral or dual-plane implants. Most non-ADM groups had either complete muscle coverage (submuscular) or dual-plane under the pectoralis major muscle plus the lower pole under the skin/subcutaneous tissue or serratus anterior muscle fascia.

Only two used the same surgery/implant position with and without ADM [105,274]. Nahabedian et al. [274] used dual-plane implants with or without ADM. Infection rate was 5.85% with ADM and 5.0% without. Warren-Peled et al. [105] used ADM along the inferolateral border of the pectoralis major muscle, with the non-ADM group having the same implant placement but without ADM. The inferior aspect of the pectoralis major was left intact at the inferior origin superior to the IMF. Technical refinements to reduce NAC complications to <5% had been made prior to this prospective study, and then three cohorts of consecutive patients were studied (no ADM 2006 to 2007, with ADM 2007 to 2008, selective ADM for patients with thinner skin flaps 2008 to 2010). Groups were not equivalent, and the difference in PMRT use was statistically significant. Multivariate analysis was not used except to compare groups. Infection was 15.8% with selective ADM use, 20% in the ADM cohort, and 27.8% without ADM (*p* = 0.04); expander-implant loss was 5% with selective ADM, 7% in the ADM cohort, 17.8% in the cohort without ADM (*p* = 0.001); unplanned return to the operating room was 10% with selective ADM, 11% in the ADM cohort, and 23.3% without ADM (*p* = 0.004); and skin flap necrosis 6.2% with selective ADM, 6% in the ADM cohort, and 11.1% without ADM (*p* = 0.26). After adjusting for age and BMI, they found that the use of PMRT increased the risk of complications between two-fold and six-fold. Given unequal use of PMRT between groups, as well as higher rates of therapeutic mastectomy in the no ADM group, these alone could account for differences in complications observed. While the study may be too small for multivariate analysis, its omission makes conclusions of low value. This placement varies from the usual dual-plane or submuscular placement, and with these modifications, ADM use may only be required in select cases.

The other studies used a dual-plane approach with ADM covering the inferior pole and attached to the pectoralis major muscle and IMF area. Four studies compared dual-plane implants with ADM to submuscular implants without ADM [275,279,281,282]. Differences in outcome could be due to patient selection, use of ADM, or differences in the plane of implants; multivariate analysis cannot control for the latter two variables. Therefore, these studies can only be used to compare dual-plane plus ADM to submuscular without ADM but do not address the role of ADM. Vardanian et al. [279] found that the dual-plane plus ADM group had lower rates of capsular contracture, IMF problems, and mechanical shift in multivariate analysis and also fewer overall complications in univariate analysis. Aesthetic outcomes were rated as significantly better in the dual-plane group. Parks et al. [281] used logistic regression for expander loss and found no difference; multivariate analysis was not used for other outcomes, although they reported seroma was higher in the dual-plane plus ADM group. Weichman et al. [282] did not report the multivariate results; unadjusted data showed higher rates of major complications, infections, flap necrosis, and explantation in the ADM group and no difference in seroma or hematoma. The study by Sbitany et al. [275] found similar rates of complications but did not adjust for the fact that the ADM group had a greater expander size and intraoperative fill volume.

Three publications from Brigham and Women’s Hospital in Boston [276,277,278] used dual-plane with ADM compared with either total (submuscular) coverage or dual-plane position for the group without ADM but did not subdivide results according to implant position. The publications, therefore, have the same limitations as those with submuscular implants. Due to high seroma rates with ADM in the first study (14.1% ADM vs. 2.7% without, OR = 4.24, *p* = 0.018), as well as higher rates of necrosis and infection [276], the authors made modifications to their procedure [277] and found the rate of seroma decreased (4.7% vs. 1.4%, *p* = 0.2277), although skin flap necrosis was still very high (28.3% vs. 5.5%, *p* = 0.0003). This may have been partially due to the much higher intraoperative fill volume in the ADM group (298.1 mL vs. 96.5 mL, *p* < 0.001), as well as higher mastectomy specimen weight and BMI; these are all risk factors for necrosis but were not adjusted for. A third study at the same institution but by different investigators reported surgical complications of 19.5% versus 12.3% (OR = 1.76, *p* = 0.036) and infection rates of 6.8% versus 2.5% (OR = 3.25, *p* = 0.097). Other outcomes were not analyzed with multivariate analysis; major skin necrosis was 11.7% versus 8.3% (*p* = 0.282), and seroma was 7.1% versus 3.9%, *p* = 0.136. Mastectomy weight, initial expander or implant volume, and final implant volume were higher in the ADM group, but only BMI and ADM use were included in the multivariate analysis. They did not take into account that some patients in the non-ADM group had submuscular implants.

Two studies at Northwestern Memorial Hospital in Chicago [13,262] used either AlloDerm or FlexHD with dual-plane implants versus none for submuscular implants. Differences in complications were not statistically significant. Two studies were conducted in Korea [10,263]. The first used human ADM (non-fenestrated) either dual-plane or to fill the gap in almost submuscular insertion versus submuscular without ADM [10]. In matched analysis, there were no differences in skin flap complications, infection, overall complications, major complications, and reconstructive failure; seroma rates were 4.0% versus 8.5% (*p* = 0.065). The second did not specify the type of ADM, but previous reports by the same authors used mainly AlloDerm or CGDerm/CGCryoDerm [263,264,265]. They compared ADM to cover inferolateral aspects of the tissue expander versus no ADM using serratus anterior muscle fascia and found no difference in hematoma.

#### 3.7.4. ADM Versus No ADM—Studies with AlloDerm RTU

Weichman et al. [283] compared Alloderm RTU with dual-plane implants to no ADM with submuscular implants in a prospective cohort study. Flap necrosis, major flap necrosis, infection, and cellulitis requiring intravenous (IV) antibiotics were higher with AlloDerm RTU, but the differences were not statistically significant. As baseline differences existed and multivariate analysis was not used, the interpretation of the results is uncertain.

#### 3.7.5. ADM Versus No ADM—Studies with Type of ADM Not Specified

The MROC study [69,70] reported on the use of ADM in immediate reconstruction with expanders. While prospective, it did not attempt to differentiate between ADM type and did not report the expander/implant or ADM location. The use of ADM was highly surgeon-specific, with either used in most patients (49.6% of surgeons) or rarely used (25.8% of surgeons). ADM and BMI had significant interaction on outcomes of any complication or major complications. There was no significant difference in PROs using the BREAST-Q instrument.

Lin et al. [191] compared the use of ADM (AlloDerm [most common], FlexHD, Vicryl, Vicryl/ADM hybrid, SurgiMend) versus none in immediate implant-based reconstruction. There was no difference in rates of overall complications or nipple necrosis.

Studies using the ASPS-TOPS database [267] and the ACS-NSQIP database [270,271,272,273] used CPT (Current Procedural Terminology Codes, USA) and/or ICD (International Statistical Classification of Diseases and Related Health Problems; formerly International Classification of Diseases) codes to identify expander/implant-based breast reconstruction with ADM. These codes do not give information about the specific ADM used nor the location of the ADM and implant for the ADM group. While such studies contain large numbers of patients, they are limited in the amount of information on patient and disease characteristics, surgical techniques, and outcomes. Panucci et al. [267], using ASPS-TOPS, found tissue expander loss of 2.58% with ADM versus 1.88% without (OR = 1.42, *p* = 0.026) but noted statistically significant outcomes may be clinically trivial, and ADM may improve the ability to perform breast reconstruction. Using the ACS-NSQIP database, Luo et al. [272] found higher rates of surgical site infection (3.9% vs. 3.4%, RR = 1.10, *p* = 0.03) and reoperation (7.4% vs. 6.0%, RR = 1.15, *p* < 0.001) and no difference in dehiscence (0.7% vs. 0.7%, RR = 1.02, *p* = 0.86). Graziano et al. [273] reported OR = 0.997 (*p* = 0.017) for superficial wound infection; while statistically significant due to the large database, this difference is not clinically meaningful.

Kilmer et al. [268] used the PearlDiver insurance-based database, also without information on specific ADM use or location, for patients with immediate implant or expander (plane not specified). They only conducted multivariate analysis for risk factors for implant removal, and ADM had OR = 1.22 (*p* < 0.001).

Other studies [10,13,262,263,264,265,266] used human ADM versus none but listed more than one type of ADM or did not specify the type at all. Seth et al. [13] found slightly higher rates of various complications with ADM (OR = 1.17 to OR = 1.64), but these were not statistically significant. Jordan et al. [262], Woo et al. [10], and Lee et al. [263] found no significant difference in complications. Pires et al. [266] also found no significant difference in complications within three months. Plotsker et al. [269] compared prepectoral reconstruction with or without ADM and found no statistically significant differences in expander loss, although the study was underpowered. Multivariate analysis was not used for other complications; in univariate analysis, there were no significant differences.

#### 3.7.6. Comparison of Different ADM

Various ADM products are compared in 19 publications of 15 trials summarized in Table S13 [14,191,192,284,285,286,287,288,289,290,291,292,293,294,295,296,297,298,299,300]. The BREASTrial [14,290,291,292] was an RCT comparing AlloDerm to DermaMatrix used as an inferolateral sling. Arms were also not balanced, with the DermaMatrix arm having more advanced disease (stage III or IV 18.7% vs. 37.6%) and correspondingly higher use of chemotherapy and PMRT and more smokers (0% vs. 9.4%). With only 64 patients per group, the study was underpowered to find differences in complication rates and had insufficient events for multivariate analysis. The REaCT investigators [284,285] conducted an RCT comparing non-fenestrated AlloDerm RTU versus DermACELL for dual-plane implants. Groups had 31 patients each; due to the small size, there were imbalances in mastectomy weight, smoking status, location of incisions, heart disease, breast size, and ptosis grade. There were no significant differences in complications. The AlloDerm group had better PROs of Satisfaction with Breasts and Overall Satisfaction at 3 months but no difference at 12 months. A third RCT by Broyles et al. [298] included 117 patients with AlloDerm RTU and 113 with Flex HD pliable. While no differences were found, the study was underpowered due to lower-than-anticipated complication rates; only 22 events occurred.

The other studies were non-randomized retrospective studies. Three compared AlloDerm RTU to DermACELL. Johnson et al. [288] found higher rates of seroma with the AlloDerm and no differences in surgical site infection. Zenn et al. [289] found no seroma in either group and very low rates of hematoma (0 vs. 0.8%) and infection (0.8% vs. 1.7%). Berger et al. [287] found similar 90-day complication rates.

AlloDerm was compared with FlexHD in six studies, plus the RCT by Broyles, which has already been mentioned. Palaia et al. [293] compared these with additional comparisons with or without fenestration. In the comparison of AlloDerm versus FlexHD, there were no differences in seroma, infection, or explantation; extrusion was higher in the AlloDerm group. The cosmetic score was higher in the AlloDerm group (8.7 ± 1.5 AlloDerm vs. 8.4 ± 1.7 FlexHD, *p* = 0.0717; multivariate *p* = 0.0466), but it is unclear whether this is a meaningful difference. In a comparison of fenestrated versus non-fenestrated ADM, seroma was lower in the fenestrated group; there were no differences in infection, extrusion, explantation, or cosmetic scores. Seth et al. [294] found no differences in total complications, flap necrosis, expander migration, hematoma, seroma, or exposure/dehiscence; infection was 10.3% versus 5.2% (multivariate OR = 2.11, *p* = 0.09). Liu et al. [295] reported surgical site infection of 8.5% versus 14.4% (*p* = 0.15; multivariate OR = 0.44, *p* = 0.053) and no difference in delayed healing; events were too low to analyze other complications. Ranganathan et al. [296] found no difference in seroma, delayed wound healing, return to the operating room, or implant exposure. Major infections were lower in the AlloDerm group (8.1% vs. 17.7%, OR = 0.50, *p* = 0.049 on a per-patient basis; 5.3% vs. 12.7%, OR = 0.35, *p* = 0.004 on a per-breast basis). The study by Sobti et al. [297] was more recent and used AlloDerm (60% RTU and 31% freeze-dried) or FlexHD (primarily pliable perforated); they found no differences in complications. Chu et al. [286] found no difference in expander loss (OR = 1.37, *p* = 0.658); other complications were not included in the multivariate analysis.

Keifer et al. [299] compared AlloDerm to Cortiva^®^ (RTI Surgical, Alachua, FL, USA) and found no difference in overall complications, seroma/hematoma, or infection. Mastectomy flap necrosis was 0.6% versus 4.8% (*p* = 0.022; *p* = 0.059 by logistic regression but based on only 8 events). The authors concluded complications were equivalent. Hadad et al. compared AlloDerm (aseptic) using more (traditional dual-plane) or less (patching lateral area of reconstruction; pectoralis not released) and found more seroma (3% vs. 0%, *p* = 0.01) and infection (9% vs. 1%, *p* < 0.05) with the dual-plane procedure. It is not possible to attribute differences to ADM use as surgery was also different. Lin et al. [191] compared AlloDerm, FlexHD and Vicryl (or Vicryl hybrid). AlloDerm had numerically lower unadjusted rates of overall complications, skin flap necrosis, explantation, and reconstructive failure, while FlexHD and Vicryl/Vicryl Hybrid were equivalent. Rates were similar for nipple necrosis, hematoma, and seroma. The type of ADM/mesh was not included in the multivariate analysis.

#### 3.7.7. Different ADM Preparations/Treatments

Eight studies compared various forms of ADM [246,283,301,302,303,304,305,306] and are summarized in Table S14. Five compared AlloDerm (aseptic/freeze-dried) to AlloDerm RTU (sterile) [246,301,302,303,304], and one made the same comparison and included an additional arm of contoured + fenestrated ADM (presumably RTU/sterile based on years used) [301]. One compared DermACELL or MegaDerm with fenestration [305]. The same group [306] compared sterile (irradiated) DermACELL or MegaDerm to nonsterile CGCryoderm (all fenestrated).

Hanson et al. [304] used propensity-score matching of AlloDerm aseptic versus sterile/RTU resulting in 384 matched pairs and found higher early complications (37.5% vs. 28.9%, *p* = 0.011) in the aseptic group. Surgical site complications were similar in the first stage (expander) (21.4% vs. 16.7%, *p* = 0.103) and lower with aseptic AlloDerm in the second stage (0.3% vs. 3.9%, *p* < 0.001); the latter result may be due to relatively low complications rates in both groups. Infection rates were similar (9.6% vs. 7.8%, *p* = 0.354), while failure was higher with aseptic AlloDerm (7.8% vs. 4.4%, *p* = 0.050).

Parikh et al. [246] compared AlloDerm aseptic versus sterile/RTU. In multivariate regression of primary outcomes, they found no difference in any complication or in infections requiring IV antibiotics, but explantation was 18.0% versus 12.0% (OR = 1.570, *p* = 0.0161). Other complications were low, and differences were not statistically significant; multivariate analysis was not conducted.

Widmyer et al. [303] used aseptic AlloDerm until 2011 and then sterile/RTU AlloDerm thereafter, with the last 123/227 RTU patients receiving the perforated contoured form (results not reported separately). They found higher rates of infection, implant loss, and unplanned reoperation in the aseptic group and no significant differences in seroma, hematoma, or necrosis. Multivariate analysis was not conducted; however, due to the consecutive nature of assignments, patient characteristics were similar between groups, other than that the aseptic group was younger, had lower BMI, and used more expanders (87% vs. 36%). These factors are expected to lower the risk of complications and, therefore, do not detract from the results.

Frey et al. [301] compared AlloDerm aseptic versus RTU versus contoured and fenestrated. The groups were not equivalent, with a switch to more NSM and direct-to-implant reconstruction over time and a corresponding switch to AlloDerm RTU and then contoured plus fenestrated ADM. This could contribute to the increasing rates of minor mastectomy flap necrosis over time, as well as higher rates of major mastectomy flap necrosis in the contoured arm than the RTU arm. Multivariate analysis was not conducted. Infection rates were highest with aseptic AlloDerm, intermediate with sterile RTU AlloDerm, and lowest with contour fenestrated AlloDerm (major infection 11.0% vs. 4.3% vs. 1.7%; minor infection 7.7% vs. 3.0% vs. 0%). The study by Weichman et al. [283] likely overlaps with the study by Frey et al. [301] and found no difference in flap necrosis between aseptic and RTU/sterile AlloDerm; infection was 20.0% aseptic versus 8.5% RTU (*p* = 0.0088) and cellulitis (deep infection requiring IV antibiotics) was 12.2% versus 4.7% (*p* = 0.069).

Yuen et al. [302] used a multivariable model only for the outcome of cellulitis and found this higher in the sterile/RTU group (12.5% vs. 21.0%, *p* = 0.129; aOR = 0.269, *p* = 0.011); however, they noted that cellulitis was significantly higher in patients with BMI ≥ 30 kg/m^2^ and for patients with lower BMI the type of ADM had no effect on outcome. The model included only obesity, NACT, and type of ADM. Due to differences in BMI between groups, small numbers of patients with BMI < 30 kg/m^2^, and the large effect of BMI on outcomes, these results may be less reliable than other studies that found the opposite results.

The study of Han et al. [305] comparing prepectoral wrap-around or anterior ADM coverage using DermACELL or MegaDerm (both fenestrated during operation) found similar rates of complications and noted the wrap-around could make the breast more ptotic in shape. Multivariate analysis was not used. The other study by this group compared sterilized (DermACELL or MegaDerm) versus non-sterilized ADM (CGCryoderm) with all fenestrations during operation. Surgical complications were similar, but implant failure was 3.4% versus 0%. As multivariate analysis was not used, it is unclear whether differences were due to sterilization and not baseline differences in patients.

#### 3.7.8. ADM Plus Synthetic Mesh Versus ADM Alone

Four studies had comparisons of synthetic mesh [232,244,245,307,308] and are summarized in Table S15. The study by Sigalove et al. [307] explored using a bioabsorbable synthetic mesh (GalaFLEX) to replace part of the ADM (AlloDerm) that would otherwise be used as a means of minimizing cost. AlloDerm alone was therefore compared with AlloDerm + GalaFLEX. When using both materials, AlloDerm was used to cover the lower third of the expander, and the rest of the expander was covered with GalaFLEX. Any complication rates were 7.6% with AlloDerm alone and 6.4% in the combined group (*p* = 0.590). Any skin necrosis was higher in the AlloDerm group (5.2% vs. 1.2%, *p* = 0.011); this could be due to differences in patient or disease characteristics or transition in operating methods. There were no significant differences in infection, major skin necrosis, seroma, capsular contracture, prosthesis exposure, or prosthesis loss. The study authors noted that GalaFLEX is stiffer and gives a more stable pocket, but AlloDerm is preferred for the lower pole to allow for expansion.

Levy et al. [308] compared AlloMax (fenestrated) versus P4HB. Major complications and infection were higher with AlloMax, but differences were not significant after univariate analysis. Chen et al. [244] compared prepectoral (no ADM) to dual-plane with either ADM or P4HB. Necrosis and infection were lower with P4HB (no multivariate analysis), but capsular contraction was higher in univariate and multivariate analysis. Houvenaegh et al. [232,245] compared TIGR Matrix to none in mostly prepectoral implants. In regression analysis, mesh use was associated with a higher rate of complications.

### 3.8. Question 6: Autologous Fat Grafting

**Figure 2:**
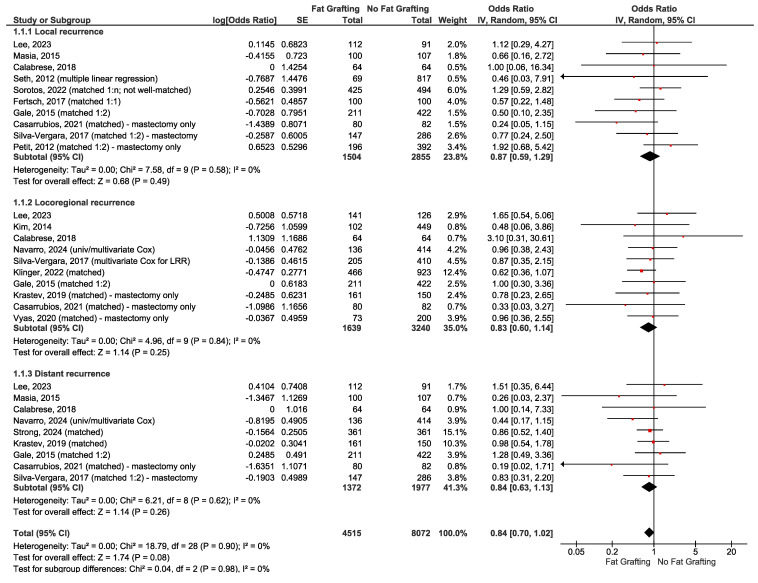
Effect of Fat Grafting on Cancer Recurrence.

**Figure 3:**
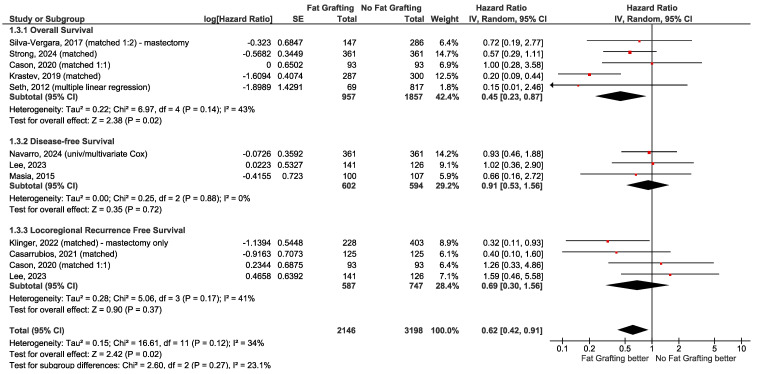
Effect of Fat Grafting on Survival Outcomes.

Twenty-nine publications of 24 studies on autologous fat grafting are summarized in Table S16 [53,54,55,57,58,309,310,311,312,313,314,315,316,317,318,319,320,321,322,323,324,325,326,327,328,329,330,331,332]. There were two RCTs and two prospective non-randomized studies; the rest were retrospective non-randomized comparative studies. One of the major concerns with fat grafting is whether there is increased cancer recurrence or lower survival. To address this, data that report on these outcomes have been summarized in forest plots in Figure 2 and Figure 3. These figures indicate that there is no significant difference in cancer recurrence with fat grafting compared with no fat grafting. Results for survival outcomes are based on fewer studies and events but suggest fat grafting also does not affect survival.

The BREAST Trial [54,55,57,309] randomized patients to reconstruction with fat grafting alone or to reconstruction with expander-implants. A negative pressure external device was used before and after fat grafting sessions. The power calculation required 86 patients per group, while only 64 and 68 patients completed follow-up. Patients with BMI > 30 kg/m^2^ or with breast size more than a C cup (unless contralateral reduction was desired) were excluded. Using the Breast-Q, they found at the 12-month assessment that the fat grafting group had higher Satisfaction with Breasts (70.3 vs. 60.4, *p* = 0.002), Physical Well-Being Chest (79.9 vs. 72.3, *p* = 0.007), and Satisfaction with Outcome (73.9 vs. 66.3, *p* = 0.04). These differences were also clinically relevant. Sexual Well-Being was different at baseline (54.4 vs. 61.1); this improved over time in the fat grafting group (61.5 at 12 months) but decreased in the implant group (58.6 at 12 months). Because of differences at baseline, the difference in Sexual Well-Being between groups at 12 months was not statistically significant. The fat grafting group had fewer non-oncologic serious adverse events, while oncologic events were similar. Long-term results are not yet available. For non-serious adverse events, the fat grafting group had higher rates of seroma and hematoma. Most other adverse events were specific to the technique used: the fat grafting arm had irritation/itch/pain or blisters due to BRAVA external expander, fat necrosis, or donor site pain, while the implant group had implant or expander rupture or migration and capsular contracture. An additional exploratory study suggested better sensibility with fat grafting [54].

The other RCT, by Gentilucci et al. [53], included 30 patients with fat injections and 30 without who had expander-implant reconstruction and PMRT. The fat injections were used after PMRT and prior to expander-implant exchange. The group with fat injections had fewer complications (*p* = 0.07), including delayed wound healing, seroma, implant extrusion and dermal fibrosis; less disability measured with the LENT-SOMA scale; and better aesthetic evaluation. Capsular contraction was similar but of lower grade in the fat injection group. The fat injection group had a soft adipose tissue thickness of 0.36 at the first session and 1.78 at the time of expander removal, while the group without fat grafting had a thickness of 0.42 at the time of expander removal (*p* < 0.001).

The MROC study [58] included 165 patients with contour irregularities or volume deficits and fat grafting between years 1 and 2. Patients with fat grafting had lower QoL than controls before fat grafting, and QoL was similar to controls afterward, suggesting that fat grafting is beneficial in patients with contour irregularities or other defects.

The other prospective study used stromal vascular fraction (SVF)-enriched fat transfer compared with fat transfer or none [319]. There were no differences in LR, LRR, or DFS; distant metastasis was 7.3% versus 3.1% versus 3.1%. Adjusted odds ratios indicated no significant differences for any recurrence events (aOR = 1.92, *p* = 0.477 for enriched vs. control; aOR = 1.26, *p* = 0.778 for normal fat transfer vs. control). DFS from the time of NSM was 37, 34, and 38 months. The SVF group had only 41 patients; it did not meet our criteria of 50 patients for this group, and the results were less reliable than for the other two comparisons. Many of the other studies matched patients with and without lipofilling and looked for oncologic events, as summarized in Figure 2 and Figure 3.

As indicated above, most studies that reported oncologic outcomes found no significant effect of fat grafting. An exception is the study by Lee et al. [329] that reported lower survival rates with fat grafting; however, there are several concerns with data analysis. Methods state that patients were categorized by timing of fat graft in relation to primary operation (mastectomy), as fat grafting ≤ 12 months or >12 months after mastectomy. It is implied but not stated explicitly that fat grafting was a one-time event that took place during the same operation as the expander-implant exchange. It is assumed that corresponding controls were patients with expander-implant exchange ≤ 12 months or >12 months after mastectomy. Results indicate the median time between mastectomy and expander-implant exchange was 12.0 months, and therefore, one-half of patients should have had the second operation after 12 months. This is contradicted by the statement that 203/267 patients had the second-stage operation within 1 year and 64/267 at >12 months. Of patients with expander-implant exchange within 12 months, 112 of 203 had fat grafting, and 91 did not. While the authors state that groups had similar baseline characteristics, data indicate that the fat graft group had more axillary dissection and positive lymph nodes, higher-stage disease, more lymphovascular invasion, and received more adjuvant chemotherapy and hormone therapy. Follow-up after the second operation was longer for the fat grafting group. These factors suggest that patients with fat grafting had a higher baseline risk of recurrence. There were ten recurrences with fat grafting and three without, and three versus zero breast cancer-related deaths. The 5-year DFS was 93.4% versus 98.7%, and the 5-year locoregional recurrence-free survival (LRRFS) was 95% versus 100%. Survival curves showed no differences until approximately 55 months. Median follow-up was 65 months, suggesting that almost half of patients were censored and did not contribute to 5-year DFS and 5-year LRRFS. The univariable analysis found ADM use had the largest effect (HR = 35.8) on LRRFS, but it was not included in multivariate analysis; tumour stage, lymphovascular invasion, BMI, and fat grafting all contributed to increased rates of LRRFS, and the contribution of fat grafting alone is unknown. Many of these factors were imbalanced in the groups. While multivariate analysis was conducted, we consider that the number of events is insufficient to conduct reliable multivariable analysis. The authors state there were no differences in outcomes with or without fat grafting when fat grafting and expander-implant exchange occurred after 12 months; however, the data, when looked at in isolation, suggest a protective effect of fat grafting (6.9% vs. 14.3% recurrence; 95.2% vs. 89.0% 5-year LRRFS; 95.2% vs. 82.1% 5-year DFS). This is based on small numbers of patients (29 with fat grafting and 35 without). The combined recurrences in both analyses were 12 versus 8 events, and this could be due to unequal baseline characteristics. Combined data have been included in the forest plots. The reversal in outcome depending on the timing of expander-implant exchange could be an artifact, and the authors of the current review do not consider it to be a reliable conclusion.

Kim et al. [313] found that complications increased with fat graft volume, with a mean volume of 45.2 mL without complications and 67.5 mL with complications. Complications tend to be minor, such as fat necrosis and cyst formation. Calabrese et al. [321] reported lower rates of capsular contracture (7.14% vs. 21.53%) with fat grafting, as well as lower rates of hematoma, implant displacement/rotation, pain, and revision surgery within 3 years. Using the BREAST-Q, patients with fat grafting reported significantly better for several scales of Satisfaction (softness, natural appearance and feel to touch, natural part of body) and of Physical Well-Being (pain in chest muscles; breast tightness, pulling, nagging feeling, sharp pains, aching feeling, throbbing feeling). There were no differences in Psychosocial and Sexual Well-Being questions.

Cason et al. [323] reported on imaging and biopsies after fat grafting compared to patients without fat grafting. They found more palpable masses (38.0% vs. 18.3%). These were mostly normal or benign on imagining, and biopsies were required in only 11.8% of patients versus 7.5% of patients. Fat necrosis was the most frequent radiologic interpretation and is generally identifiable on imaging.

Palve et al. [327] studied the use of implants and lipofilling in LD reconstruction and suggested that fat grafting can replace implant use and allow less aggressive subcutaneous tissue harvesting in the LD site. Escandon et al. [331] compared LD with or without immediate fat transfer and found no differences in breast site complications. There were higher rates of secondary fat grafting in patients with initial fat grafting, and the authors suggested this could be due to patient factors influencing the choice of initial fat grafting. The group with immediate fat transfer had less wound disruption at the donor site, possibly due to less aggressive flap harvesting in patients also having fat grafting.

## 4. Discussion

Breast reconstruction is an area of rapid growth in research and knowledge. In the literature search, there were approximately 1100 publications per year in 2014, increasing to about 1500 by 2018 and around 2000 in the latest years. Many of these reported on the experience of various surgeons in case reports or other retrospective series, as well as basic research on various topics. Most of the studies included in this systematic review were retrospective studies using hospital records to compare different treatments or factors, with randomized trials being rare. As expected with this study design, patient characteristics were often not reported in detail, and there were differences between groups being compared because treatment was designed based on patient and disease characteristics instead of by random allocation or another method to ensure equivalence. Most studies had too few patients and too few events to allow meaningful multivariate/multivariable analysis. Surgical information was often not reported in sufficient detail to allow the assignment of outcomes to differences in technique. However, we found 229 studies that met the inclusion criteria. Through this process, it became clear that studies clearly evaluating and documenting surgical experience and the effectiveness of systematic and well-documented changes in procedures would also be valuable in addressing some of the questions. These studies illustrate what has been achieved by surgeons and groups that have established expertise in various aspects of NSM and breast reconstruction and may provide some guidance to other oncologic and reconstructive surgeons. For this reason, the observations from some of these studies supplement the results of the systematic review.

### 4.1. Question 1: Patient and Disease Factors

Several patients and disease factors are often reported as potentially influencing outcomes. Studies found small or no increase in complications with increasing age after controlling for comorbidities and other factors. Reconstruction had positive benefits on QoL. Kim et al. used age as a continuous predictor in a set of 4379 patients and found complications (mastectomy skin flap/nipple necrosis, infection, seroma) increased by 1 to 2% per year of age [86]. Comparing free flap reconstruction in patients aged 70 to 74 years versus age < 55 years, Honig et al. [87] found there were small increases in rates of delayed healing (35.7% vs. 29.2%, *p* = 0.036), skin necrosis (15.3% vs. 10.7%, *p* = 0.039), and hematoma (9.3% vs. 5.3%, *p* = 0.005); they concluded an age cutoff was not warranted. A study using the ACS-NSQIP database for patients with pedicled flaps found a small increase in overall complications (OR = 1.010, 95% CI = 1.004 to 1.006, *p* = 0.002), severe complications (OR = 1.043, 95% CI = 1.019 to 1.068, *p* < 0.001), and wound complications (OR = 1.009, 95% CI = 1.000 to 1.018, *p* = 0.053), but concluded the effect of age does not have strong predictive power and may not be clinically relevant on its own. [90].

Patient factors such as smoking and diabetes, which are well known to affect circulation and healing, may increase the risk of certain complications but, on their own, are not absolute contraindications to reconstruction. Aesthetic outcomes may be worse for patients with obesity. NSM may not be feasible without repositioning the nipple in patients with a higher degree of obesity or ptosis. Obesity is also a risk factor for surgical complications, and the risk increases with grade/degree of obesity. Effects of various factors may be additive and, together, make reconstruction (or certain types of reconstruction) inadvisable. As risk profiles of different reconstructions vary (outside the scope of this review), some patients may be considered poor candidates for specific reconstructions. Several studies reviewed for other questions indicated that large breast size (or mastectomy weight) and large implant size were risk factors for complications as these are associated with poorer skin perfusion. Risk can be reduced by ensuring expanders or implants do not put pressure on the skin or tension on the closure, which may mean lower initial expansion or smaller implants.

### 4.2. Question 2: Timing of Reconstruction

The evidence on timing of reconstruction was very limited. For an individual patient, risks for one option, such as immediate reconstruction, may be considered too high and delayed reconstruction is recommended by the surgeon. Alternatively, when both immediate and delayed reconstruction are considered to be of acceptable risk, immediate reconstruction may be preferred due to better short- to medium-term psychological outcomes. There is consistent evidence for this, although limited in this review.

As a general principle, immediate reconstruction will require a longer and more complex operation than mastectomy alone, but with mastectomy alone, the reconstruction will require an additional operation with its own set of complications. It is, therefore, difficult to compare the total level of complications for both mastectomy and reconstruction or alternatively to measure complications only due to reconstruction but not mastectomy. Very few studies mentioned or accounted for this. In general, mastectomy alone requires the shortest operative duration, followed by mastectomy plus implants; mastectomy plus autologous reconstruction requires the longest operating time. Some patients, due to comorbidities, may not be good candidates for a longer operation, and for this reason, delayed reconstruction may be advised. Immediate reconstruction may not be advised following SSM or NSM in cases when the skin shows poor perfusion. In these cases, a short delay in reconstruction may occur (e.g., 2 weeks), and a temporary expander is sometimes used. This may be preplanned or decided during the mastectomy operation. Patient preference is a major factor, with many preferring immediate reconstruction in order not to have to wake up from the operation (and live like that for months or years) without a breast. Others may be undecided about reconstructive options, and a delay gives them additional time. BREAST-Q scores tend to be lowest between mastectomy and reconstruction and then improve gradually following reconstruction, illustrating a negative effect of delayed reconstruction. Some other guidelines suggest that immediate reconstruction should be the norm. The NICE recommendation is to “offer immediate breast reconstruction to women who have been advised to have a mastectomy, including those who may need RT, unless they have comorbidities that rule out reconstructive surgery” [333].

RT is known to cause an increase in complications, including capsular contracture when expanders or implants are used, delayed healing, and worse aesthetic results. The increased complications exist, regardless of the timing of reconstruction, but may have a different profile. For two-stage implants, irradiation of expanders is common, thus avoiding irradiation after the final implant, and this has been found to decrease the rate of capsular contracture compared with irradiation of the implant [102]. Results were similar to those of PRMT followed by delayed implant-based reconstruction. However, grade III complications and reconstructive failure were lowest in the immediate group. Staged or delayed-immediate or immediate-delayed with an expander or temporary implant after SSM or NSM has been used to maintain the skin flap and provides a breast mound while awaiting reconstruction. This allows time for recovery prior to reconstruction or while awaiting final pathology or other information that determines the need for PMRT. It has also been used on the assumption that PMRT to an expander is preferable to irradiation of the final implant or autologous flap. Several small studies (not meeting our sample size criteria) have used this with good results. Christopher et al. [16] found less fat necrosis and skin necrosis in the delayed group, but infection and wound dehiscence during the expander-delay stage increased the overall complication rate in the delayed group. Overall, there is limited evidence regarding the optimal timing of reconstruction, and the decision may come down to patient preference, individual risks for specific complications, and the ability of surgeons to minimize or treat complications.

### 4.3. Question 3: Nipple-Sparing Mastectomy

Studies found that NSM and SSM have equivalent oncologic outcomes and that risks of LR in patients who had NSM are low, provided an adequate assessment of NAC involvement is conducted. For patients considered suitable for SSM, NSM may also be considered. A small (<1 cm or <2 cm) TND was not an absolute contraindication for attempting NSM. Most studies considered inflammatory breast cancer, Paget disease, nipple involvement and nipple retraction as contraindications and patients were excluded. The lowest recurrence rates were found in studies that specified that nipple coring or sampling of ducts in the core occurred. It is not possible to determine whether duct sampling or nipple coring is essential or is an indication of better-quality studies and surgeon expertise. Studies with the highest rates of LR or recurrence in the NAC were conducted in Asia and did not mention duct sampling/coring. Worse outcomes could be due to patient factors or surgical procedures.

The studies indicate wide variability of complications and other outcomes depending on patient and disease factors, as well as surgeon expertise and techniques used. Surgeon factors include type of incision used, degree of excision of breast tissue, tension on skin (which may be reduced by lower filling of expanders, smaller implants, or surgical delay before reconstruction), methods to assess vascularity/blood supply, dissection methods, use and duration of surgical drains, antibiotic use, and whether attempts were made to identify and preserve, or reconnect nerves.

### 4.4. Question 4: Plane of Implants

Animation deformity, pain, and asymmetry are well-known complications of submuscular and, to a lesser extent, dual-plane implants and may be of serious concern for some patients. Removal of implants and replacement with prepectoral implants resolved the presenting complaints and improved BREAST-Q scores. Prepectoral implants avoid these complications and generally have shorter operations. The use of ADM and synthetic mesh allowed a rapid increase in prepectoral and dual-plane reconstruction. The ADM or mesh allowed implant placements that previously were not feasible. However, there is concern about increased infection and other complications with ADM (see Question 5). A few studies have adapted the process to allow prepectoral or dual-plane reconstruction without ADM, and this appears to be a promising approach for some patients.

Most studies on prepectoral reconstruction used ADM for full or anterior coverage, and several studies used ADM in dual-plane reconstruction to create a sling supporting the lower pole of the expander or implant. Short-term postoperative pain was lower with prepectoral reconstruction. There was generally little difference in complications between these two approaches, and the profile varied among studies. Overall, the prepectoral location appears to have slightly fewer complications. It should be noted that patients deemed not suitable for prepectoral reconstruction were often converted to subpectoral reconstruction, which may influence the reported results.

Details of reconstruction, including the type and location of implants or autologous tissue, should be documented in the clinical records and available to radiologists when conducting follow-up imaging.

### 4.5. Question 5: Acellular Dermal Matrix Use

Increased rates of infection and seroma have been reported with the use of ADM. Both of these appear modifiable by selection and treatment of ADM prior to use. AlloDerm is the most frequently studied material in the included studies and was aseptically processed but not sterilized until 2010, after which it was replaced by AlloDerm RTU terminally sterilized to a SAL of 10^−3^. Some other products have SAL < 10^−6^. In earlier studies, meshing was performed at the clinic prior to use; pre-meshed or perforated forms are now commercially available, although at additional cost. Some current studies report lower infection rates, and this may be due to changes in processing as well as reduced handling and better infection control in the clinic. Meshing allows better conformation to the implant, reduced size of ADM and, therefore, cost, better exchange of fluid (thought to reduce seroma), and better integration into the tissue. Surgical factors may be important, as illustrated by a decrease in seroma rate from 14.1% to 4.7% in the ADM groups and 2.7% to 1.4% without ADM after modification of procedures at Brigham and Women’s Hospital in Boston [276,277,278].

As noted in Question 4, comparisons are often confounded by changes in both plane and ADM use. Generally, differences in complications were none or slightly increased with ADM but not statistically significant. Many studies reported no significant difference between the use of ADM or no ADM. Studies with the ASPS-TOPS and ACS-NSQIP databases found a small increase in surgical site infection, expander loss, and reoperation, but differences may not be clinically significant. Studies comparing AlloDerm to other ADMs found no consistent differences. Studies comparing AlloDerm aseptic to AlloDerm RTU found more complications, including infection with the aseptic form, although this is of mostly historical importance in comparing older studies. Studies of synthetic absorbable mesh were too limited to comment on the relative value of ADM versus synthetic mesh.

### 4.6. Question 6: Autologous Fat Grafting

Studies indicated there were no significant differences in cancer recurrence with or without fat grafting. Fat grafting was found to have several benefits. Fat grafting was most commonly used to treat contour irregularities or volume deficits and improved aesthetic results, QoL, and outcomes of the BREAST-Q. This appears to be a well-accepted and effective use. A small RCT [53] also reported benefit after PMRT and prior to expander-implant exchange. The group with fat injections had fewer complications, including delayed wound healing, seroma, implant extrusion and dermal fibrosis; less disability measured with the LENT-SOMA scale; and better aesthetic evaluation. Capsular contraction was similar but of lower grade in the fat injection group. This and other studies suggest fat grafting may help in the healing of tissue after RT damage and improve the quality of the radiated skin [334]. One study [327] found fat grafting may replace implants used in conjunction with LD flap reconstruction. Another application of fat grafting is for total breast reconstruction without the use of implants or autologous flaps. This may be performed with pre-expansion, such as in the randomized BREAST Trial [54,55,57,309]. Good results have been obtained; however, patients need to be very committed to this process. Non-comparative studies [335,336] have reported good results without pre-expansion.

Palpable masses as a result of fat necrosis may occur in patients who have received fat transfer. Fat necrosis was the most frequent radiologic interpretation and is generally identifiable on imaging without biopsy in most cases. Details of reconstruction including location and type of implants or autologous reconstruction, and use of fat grafting should be reported in the clinical history and made available to radiologists to aid in interpretation and avoid false positive diagnoses. A recent book (2023) on the topic of fat transfer in plastic surgery covers much of the background and technical details [337]. Several chapters are relevant to breast reconstruction. The UK guideline is a reference document on lipomodelling of the breast that may also be useful [338].

## 5. Conclusions

For many questions, we were unable to conclude that one treatment was better than the other and found that both (all) had acceptable outcomes, often with different profiles of complications that could mean appropriateness for some patients but not others. Patient characteristics (such as comorbidities affecting wound healing and risks of infection and tissue necrosis), breast size and implant size, ptosis, and receipt of RT interact with other factors, and therefore, decisions tend to be quite individualized.

Our overall conclusion is that all the variations of reconstruction are oncologically safe, and there is a role for each. There is moderate certainty of evidence to support the use of NSM, immediate and delayed reconstruction, prepectoral and subpectoral planes, the use of ADM, and fat grafting. The best approach for each patient varies depending on specific patient factors, including individual values and judgement on the competing risks and benefits.

This systematic review is the basis for recommendations in a clinical practice guideline [22]. This guideline will be available in a subsequent issue of this journal and should be referred to for more detailed conclusions.

## Figures and Tables

**Table 1:** Summary Statistics for Patients with NSM.

Measure	LR	LR in NAC	RR	LRR	CBC	DM	Death	5-y OS	5-y DFS	Total Nipple Necrosis	Partial NAC Necrosis	Flap Necrosis
# Studies	45	42	13	24	7	39	14	14	12	45	36	27
Min (%)	0.0	0.0	0.7	0.9	0.9	0.0	0.4	83.5	68.0	0.0	0.9	0.4
Max (%)	10.9	6.0	9.6	16.2	3.9	20.4	12.2	99.1	98.3	19.5	23.7	23.3
Avg (%)	4.0	1.2	2.9	5.1	2.0	5.6	3.6	94.6	87.4	2.8	7.9	7.4
Median (%)	3.7	0.5	1.8	3.9	2.0	4.0	2.6	95.8	90.5	1.7	6.9	6.3

Abbreviations: CBC, contralateral breast cancer; DFS, disease-free survival; DM, distant metastasis; LR, local recurrence; LRR, locoregional recurrence; NAC, nipple-areolar complex; OS, overall survival; RR, regional recurrence.

## Data Availability

This review is based on data from publicly available and referenced sources. Data sharing is not applicable to this article.

## Supplementary Materials

The following supporting information can be downloaded at: https://www.mdpi.com/article/10.3390/curroncol32040231/s1, Table S1: The literature Search Strategy; Tables S2–S16: Evidence Tables.

## Appendix A

**Table A1:** Assessment of Bias in RCTs using the Cochrane Risk of Bias 2 (RoB2) Tool.

Study Analysis	Study ID	Experimental	Comparator	Outcome	D1	D2	D3	D4	D5	Overall
Intention-to-treat	NCT00639106 McCarthy, 2012 [280]	ADM	None	Postoperative pain; pain in the expansion phase						
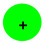
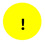
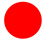
Intention-to-treat	BREASTrial, stage I NCT00872859 Mendenhall, 2015 [290]	AlloDerm	DermaMatrix	Overall complications; other complication						
Intention-to-treat	BREASTrial, stage II NCT00872859 Mendenhall, 2017 [291]	AlloDerm	DermaMatrix	Overall complications; other complication						
Intention-to-treat	BREASTrial, stage III NCT00872859 Mendenhall, 2023 [292]	AlloDerm	DermaMatrix	Overall complications						
Intention-to-treat	NCT03145337 Broyles, 2021 [298]	AlloDerm RTU	FLEX HD pliable	Overall complications; other complication						
Per Protocol	REaCT investigators NCT03064893 Arnaout, 2021 [284]	Non-fenestrated AlloDerm RTU	DermACELL	Complications; QoL						
Intention-to-treat	Gentilucci, 2020 [53]	Expander, PMRT, fat grafting, expander-implant exchange	Expander, PMRT, expander-implant exchange (no fat grafting)	Soft adipose tissue thickness; complications; disability; aesthetics						
Per Protocol	BREAST Trial NCT02339779 Piatkowski, 2023 [54,55,56,57]	Fat grafting alone	Expander-implants	Breast-Q						
	Low risk of bias			Some concerns		High risk of bias				

D1, Randomisation process; D2, Deviations from the intended interventions; D3, Missing outcome data; D4, Measurement of the outcome; D5, Selection of the reported result.

## Appendix B

**Table A2:** Quality Assessment by AMSTAR II of Systematic Reviews in Table S2 *.

Citation	1. PICO Components Included	2. Protocol Prior to Review	3. Study Design Inclusion Explanation	4. Comprehensive Search Strategy	5. Duplicate Study Selection	6. Duplicate Data Extraction	7. Details of Excluded Studies	8. Description of Included Studies	9a. RoB Assessment (RCTs)	9b. RoB Assessment (NRSIs)	10. Funding Sources	14. Heterogeneity Absent or Discussed	15. Publication bias Assessed	16. Reports COI	Overall Rating of Quality
Panayi, 2018 [71]	Y	Y	N	PY	N	N	Y	PY	N/A	Y	N	Y	N	Y	Moderate
Tan, 2022 [72]	Y	N	N	Y	N	N	N	Y	N/A	Y	N	Y	N	Y	Moderate
ElAbd, 2022 [73]	Y	N	N	PY	N	N	N	Y	N/A	Y	N	Y	N	Y	Moderate
Liu, 2022 [74]	Y	Y	N	PY	Y	N	N	Y	N/A	Y	N	Y	Y	Y	Moderate
Mortada, 2023 [75]	Y	Y	N	PY	N	N	N	Y	N/A	Y	N	Y	Y	Y	Moderate
Theocharidis, 2018 [76]	Y	PY	N	Y	Y	N	N	Y	N/A	N	N	Y	N	Y	Low
Mrad, 2022 [77]	Y	PY	N	Y	Y	N	N	Y	N/A	N	N	Y	N	PY	Low
Chung, 2021 [78]	Y	N	N	PY	N	N	N	Y	N/A	N	N	Y	Y	Y	Low
Bond, 2021 [79]	Y	N	N	Y	Y	N	Y	Y	N/A	Y	N	Y	Y	Y	Moderate
Chicco, 2021 [80]	Y	N	N	Y	N	N	Y	Y	N/A	N	N	Y	Y	Y	Low
Varghese, 2021 [81]	Y	Y	N	Y	Y	Y	N	Y	N/A	Y	N	Y	Y	Y	High
Spera, 2020 [82]	Y	N	PY	PY	Y	PY	N	Y	N/A	Y	N	Y	Y	Y	Moderate
Hong, 2021 [83]	Y	N	N	Y	Y	Y	N	Y	N/A	Y	N	Y	Y	Y	Moderate
Zugasti, 2021 [84]	Y	PY	N	PY	N	N	Y	Y	N/A	Y	N	Y	Y	PY	Moderate
Pu, 2018 [15]	Y	PY	N	Y	Y	N	N	Y	N/A	PY	N	Y	Y	Y	Low
Magill, 2017 [85]	Y	Y	N	Y	N	N	N	Y	N/A	N	N	Y	N	Y	Low
Liew, 2021 [18]	Y	Y	N	Y	Y	Y	N	Y	N/A	Y	N	Y	N	Y	High

* AMSTAR II [38]. Abbreviations: COI, conflict of interest; NRSI, non-randomized study of intervention; PICO, population, intervention, comparison, and outcome; PY, probably yes (yellow background); N, no (red background); RCT, randomized controlled trial; RoB, risk of bias; Y, Yes (green background); N/A, not applicable (grey background).
