# Supplementary material for: Postmastectomy Breast Reconstruction in Patients with Non-Metastatic Breast Cancer: A Systematic Review
Source: Curr Oncol. 2025 Apr 16;32(4):231. doi: 10.3390/curroncol32040231 (PMC12025830; doi:10.3390/curroncol32040231)
Supplement: Supplementary file 1 [file curroncol-32-00231-s001.zip › curroncol-3536959-supplementary.pdf]

Postmastectomy Breast Reconstruction in Patients with Non-Metastatic Breast Cancer

Toni Zhong, Glenn G Fletcher, Muriel Brackstone, Simon Frank, Renee Hanrahan, Vivian Miragias, Christiaan Stevens, Danny Vesprini, Alyssa Vito, Frances C Wright

Supplementary Materials

Table S1. Literature Search Strategy 2

Table S2. Systematic Reviews and Meta-analyses 5

Table S3. Effect of Age on Reconstructive Outcomes 13

Table S4. Question 2: Immediate versus Delayed 16

Table S5. Question 3a: NSM versus SSM 32

Table S6. Question 3b: Oncologic and nipple outcomes according to criteria for NSM patient selection & Question 3c: Surgical factors influencing nipple outcomes 37

Table S7. Question 3b/c: Summary Statistics for Patients with NSM, Excluding Four Studies* 100

Table S8. Question 4: Comparisons of Prepectoral, Subpectoral, and/or Dual-plane Reconstruction. A. Prepectoral versus Other, ADM use Similar 101

Table S9. Question 4: Comparisons of Prepectoral, Subpectoral, and/or Dual-plane Reconstruction. B. Conversion of subpectoral to prepectoral 123

Table S10. Question 4: Comparisons of Prepectoral, Subpectoral, and/or Dual-plane Reconstruction. C. Prepectoral, Subpectoral, and/or Dual-plane Reconstruction; ADM different in each arm 129

Table S11. Types of Mesh 134

Table S12. Question 5: Acellular Dermal Matrix. A. ADM versus none 135

Table S13. Question 5: Acellular Dermal Matrix. B. Comparison of ADMs 153

Table S14. Question 5: Acellular Dermal Matrix. C. ADM Treatments 165

Table S15. Question 5: Acellular Dermal Matrix. D. Comparison of synthetic mesh 171

Table S16. Question 6: Autologous Fat Grafting 175

Table S1. Literature Search Strategy

Table S1(a). Medline, Embase, EBM Reviews - Cochrane Central Controlled Trials*

Line	Query	Number of citations
1	exp breast neoplasms/ or exp breast cancer/ or paget's disease, mammary/ or exp Paget nipple disease/ or (ductal carcinoma or lobular carcinoma or ductolubular carcinoma or DCIS or LCIS).ti,kw. or ((breast: or mammar: or nipple:) adj3 (cancer: or tumour: or tumor: or neoplasm: or carcinoma: or adenocarcinoma: or Paget: disease)).ti,kw. or exp mastectomy/ or (mastectom: or PMRT or postmastectom: or post-mastectom: or post mastectom:).mp. [Breast Cancer terms]	1,108,147
2	1 and (exp mammaplasty/ or exp breast reconstruction/ or exp breast implants/ or breast endoprosthesis/ or exp breast prosthesis/ or implants, artificial/ or prosthesis/ or silicones/ or ((implant or implants or reconstruct: or autologous) adj4 (breast or mammar:)).ti,ab,kw.) [Breast Cancer and (Reconstruction or Implants)]	32,469
3	breast reconstruction.ti,kw,ab. or exp nipple-sparing mastectomy/ or ((prepectoral or pre-pectoral or subpectoral or sub-pectoral or premuscular or pre-muscular or submuscular or sub-muscular or submammary or sub-mammary or subglandular or sub-glandular or retroglandular or above muscle or subfascial or retropectroral or under muscle or dual-plane) and (breast: or mammar:)).mp. or Nipples/bs, dg, ir, re, su, tr or ((nipple: or skin:) adj4 (sparing or mastectomy)).mp. or nipple areola.mp. or nipple-areola.mp. or (tumo*r: adj3 nipple).mp. [Breast Reconstruction Terms]	35,701
4	(exp Surgical Flaps/ or exp deep inferior epigastric perforator flap/ or exp tissue flap/ or exp epigastric arteries/ or exp Transplantation, Autologous/ or exp Autografts/ or (diep or deep inferior epigastric or TRAM or transverse rectus abdomin: or pedicle or flap or flaps or autologous tissue or transverse rectus abdomin: or SIEA or superficial inferior epigastric artery or latissimus dorsi or thoracodorsal artery perforator or TDAP or lumbar artery perforator or LAP flap or LAP free flap or gluteal free flap or gluteal artery perforator or GAP flap or superior gluteal artery perforator or SGAP or inferior gluteal artery perforator or IGAP or upper gracilis or TUG or VUP or DUG or profunda artery perforator or PAP flap or lateral thigh perforator or LTP).mp.) and (breast: or mammary:).mp. [Autologous Breast Reconstruction Terms]	27,715
5	exp bioabsorbable mesh/ or exp surgical mesh/ or exp tissue scaffold/ or (bioabsorbable mesh or surgical mesh or surgical scaffold: or tissue scaffold:).mp. or exp acellular dermal matrix/ or exp Acellular Dermis/ or exp skin allograft/ or exp biodegradable implant/ or exp Tissue Scaffolds/ or exp Absorbable Implants/ or (((acellular or decellular: or dermal or biologic: or synthetic or absorbable) adj2 (matrix or matrices or matrixes or scaffold: or mesh)) or (acellular adj2 derm:) or ADM or HADM).mp. or (AlloDerm or Allomax or Cortiva or DermACell or DermaMatrix or DermaPure or FlexHD or Flex HD or Graftjacket or hMatrix or Neoform or Repriza or BellaDerm or SimpliDerm or MODA or "matrice omologa dermica acellulata" or Epiflex or Megaderm).mp. or (GalaFlex: or GalaSHAPE: or GalaForm: or Phasix or Seri Surgical Scaffold or Seri or TIGR).mp. [ADM or synthetic matrix]	161,344
6	exp Transplantation, Autologous/ or exp autograft/ or exp autotransplantation/ or exp Adipose Tissue/tr or exp adipose tissue/su or ((autologous adj2 graft:) or (autologous adj2 fat:) or (fat: adj2 graft:) or (adipose adj2 derived) or lipofill: or lipomodel: or lipoinject: or fat transfer: or fat injection or BRAVA or Renuva).mp. [Autologous Fat Grafting]	163,641
7	(1 or 2 or 3 or 4) and (5 or 6) [(breast cancer or reconstruction) and (ADM or fat grafting)]	10,223
8	(2 or 3 or 4) not 7 [reconstruction other than ADM or fat grafting]	50,085
9	exp phase 3 clinical trial/ or exp "phase 3 clinical trial (topic)"/ or exp clinical trial, phase iii/ or exp clinical trials, phase iii as topic/ or exp phase 4 clinical trial/ or exp "phase 4 clinical trial (topic)"/ or exp clinical trial, phase iv/ or exp clinical trials, phase iv as topic/ or exp randomized controlled trial/ or exp "randomized controlled trial (topic)"/ or exp randomized controlled trials as topic/ or exp controlled clinical trial/ or "controlled clinical trial (topic)"/ or controlled clinical trials as topic/ or exp	5,317,659

	randomization/ or exp random allocation/ or exp double-blind method/ or exp single-blind method/ or exp double blind procedure/ or exp single blind procedure/ or exp triple blind procedure/ or exp placebos/ or exp placebo/ or ((exp phase 2 clinical trial/ or exp "phase 2 clinical trial (topic)"/ or exp clinical trial, phase ii/ or exp clinical trials, phase ii as topic/ or exp clinical trial/ or exp prospective study/) and random$.tw.) or (((phase II or phase 2 or clinic$) adj3 trial$) and random$).tw. or ((singl$ or double$ or treble$ or tripl$) adj3 (blind$ or mask$ or dummy)).tw. or placebo?.tw. or (allocat: adj2 random:).tw. or (rct or phase III or phase IV or phase 3 or phase 4 or randomi$: or randomly).tw. or (random$ adj3 trial$).mp. or "clinicaltrials.gov".mp. [RCT search filter]	
10	(7 or 8) and 9 [RCTs]	4418
11	(7 or 8) and (systematic review: or meta-analys: or metaanalys: or meta analy:).ti,pt.	1312
12	(7 or 8) and (*practice guideline/ or practice guideline:.ti.)	136
13	(7 or 8) not (10 or 11 or 12) not (abstract or letter or comment or editorial or news or note).pt.	43,747
14	remove duplicates from 10	2789
15	remove duplicates from 11	821
16	remove duplicates from 12	123
17	limit 13 to yr="2022 -Current"	3352
18	limit 13 to yr="2020 - 2021"	5513
19	limit 13 to yr="2018 - 2019"	4290
20	limit 13 to yr="2015 - 2017"	5583
21	limit 13 to yr="2012 - 2014"	4735
22	limit 13 to yr="2008 - 2011"	4761
23	limit 13 to yr="2001 - 2007"	5341
24	limit 13 to yr="1990 - 2000"	5716
25	13 not (17 or 18 or 19 or 20 or 21 or 22 or 23 or 24)	4456
26	remove duplicates from 17	2115
27	remove duplicates from 18	3510
28	remove duplicates from 19	2735
29	remove duplicates from 20	3534
30	remove duplicates from 21	2883
31	remove duplicates from 22	2911
32	remove duplicates from 23	3323
33	remove duplicates from 24	3794
34	remove duplicates from 25	3180
35	or/26-34	27,985
36	26 or 27 or 28	8360
37	or/29-31	9328
38	32 or 33	7117
39	34	3180

* Embase <1974 to 2023 February 23>; EBM Reviews - Cochrane Central Register of Controlled Trials <January 2023> Ovid MEDLINE(R) and Epub Ahead of Print, In-Process, In-Data-Review & Other Non-Indexed Citations, Daily and Versions <1946 to February 23, 2023>

Feb 28, 2023: Added truncation mark : to nipple areola and nipple-areola terms to capture areolar in line 3 (nipple areola: or nipple-areola:).mp. After deduplication was 334 results

Table S1(b). EBM Reviews - Cochrane Database of Systematic Reviews*

Line	Query	Number of citations
1	(ductal carcinoma or lobular carcinoma or ductolubular carcinoma or DCIS or LCIS or ((breast: or mammar: or nipple:) adj3 (cancer: or tumour: or tumor: or neoplasm: or carcinoma: or adenocarcinoma: or Paget: disease)) or (mastectom: or PMRT or postmastectom: or post-mastectom: or post mastectom:)).mp. [Breast Cancer terms]	557
2	1 and ((implant or implants or reconstruct: or autologous) adj4 (breast or mammar:)).mp. [Breast Cancer and (Reconstruction or Implants)]	23
3	(breast reconstruction or ((prepectoral or pre-pectoral or subpectoral or sub-pectoral or premuscular or pre-muscular or submuscular or sub-muscular or submammary or sub-mammary or subglandular or sub-glandular or retroglandular or above muscle or subfascial or retropectroral or under muscle or dual-plane) and (breast: or mammar:)) or ((nipple: or skin:) adj4 (sparing or mastectomy)) or nipple areola or nipple-areola or (tumo*r: adj3 nipple)).mp. [Breast Reconstruction Terms]	24
4	((diep or deep inferior epigastric or TRAM or transverse rectus abdomin: or pedicle or flap or flaps or autologous tissue or transverse rectus abdomin: or SIEA or superficial inferior epigastric artery or latissimus dorsi or thoracodorsal artery perforator or TDAP or lumbar artery perforator or LAP flap or LAP free flap or gluteal free flap or gluteal artery perforator or GAP flap or superior gluteal artery perforator or SGAP or inferior gluteal artery perforator or IGAP or upper gracilis or TUG or VUP or DUG or profunda artery perforator or PAP flap or lateral thigh perforator or LTP) and (breast: or mammary:)).mp. [Autologous Breast Reconstruction Terms]	32
5	(bioabsorbable mesh or surgical mesh or surgical scaffold: or tissue scaffold: or (((acellular or decellular: or dermal or biologic: or synthetic or absorbable) adj2 (matrix or matrices or matrixes or scaffold: or mesh)) or (acellular adj2 derm:) or ADM or HADM) or (AlloDerm or Allomax or Cortiva or DermACell or DermaMatrix or DermaPure or FlexHD or Flex HD or Graftjacket or hMatrix or Neoform or Repriza or BellaDerm or SimpliDerm or MODA or "matrice omologa dermica acellulata" or Epiflex or Megaderm) or (GalaFlex: or GalaSHAPE: or GalaForm: or Phasix or Seri Surgical Scaffold or Seri or TIGR)).mp. [ADM or synthetic matrix]	106
6	((autologous adj2 graft:) or (autologous adj2 fat:) or (fat: adj2 graft:) or (adipose adj2 derived) or lipofill: or lipomodel: or lipoinject: or fat transfer: or fat injection or BRAVA or Renuva).mp. [Autologous Fat Grafting]	64
7	(1 or 2 or 3 or 4) and (5 or 6) [(breast cancer or reconstruction) and (ADM or fat grafting)]	12
8	(2 or 3 or 4) not 7 [reconstruction other than ADM or fat grafting]	37
9	7 or 8	49

*EBM Reviews - Cochrane Database of Systematic Reviews <2005 to February 22, 2023>

Back to Start of Tables

Table S2. Systematic Reviews and Meta-analyses

Citation	Title	Topic	Search details	Review details	Number of included studies; number of patients	Results or conclusions
Comorbidities/patient factors						
BMI or obesity						
Panayi, 2018 [71]	Impact of obesity on outcomes in breast reconstruction: A systematic review and meta-analysis	Effect of obesity (BMI >30 kg/m^2)	Cochrane, PUBMED, and EMBASE from inception to June 1, 2016	Conducted in line with Cochrane Handbook; published protocol; GRADE to assess methodological quality; used Quality of Reporting of Meta-analyses guidelines; reported in line with PRISMA criteria	33 studies (29 with enough data for meta-analysis; 71,368 pts, 20,061 obese)<br>Only non-RCTs were found	Complications, obese vs. other: surgical RR=2.29 (fat necrosis RR-1.65, seroma RR=1.96, partial flap failure RR=1.60, total flap failure RR=1.97, wound dehiscence RR=2.51, wound infection RR=2.34, hernia RR=1.67); medical RR=2.89, return to operating room RR=1.91<br>Subgroups of surgical complications (obese vs. non-obese): implants RR=2.64, autologous RR=2.59<br>With just comparative studies, RR=2.36 for surgical complications<br>Surgical complications by class of obesity: class I (30-34.9 kg/m^2) RR=1.32; class II (35-39.9 kg/m^2) RR=1.84; class III (>40 kg/m^2) RR=1.66
Tan, 2022 [72]	Deep inferior epigastric perforator (DIEP) flap safety profile in slim versus non-slim BMI patients: A systematic review and meta-analysis	DIEP in slim vs. non-slim pts; autologous DIEP	Cochrane, EMBASE, OVID Medline, PubMed, and Web of Sciences; searched Feb 1, 2021; looked at reference lists of retrieved articles	Followed PRISMA guidelines; quality assessed using MINORS	7 studies, 574 pts slim (low BMI; mean 22.9 kg/m^2) and 901 pts non-slim (mean BMI 27.9 kg/m^2)	No difference in complete or partial flap loss, fat necrosis, all complications, abdominal wound healing, infections, seroma
ElAbd, 2022 [73]	Autologous versus alloplastic reconstruction for patients with obesity: A systematic review and meta-analysis	Autologous vs. implants in pts with obesity<br>Definition of obesity not reported; data tables suggest cutoff ≥30	PubMed, Cochrane, Google Scholar, Embase from inception to Dec 31, 2020; cross-bibliography review of included studies	Followed PRISMA protocol; included controlled studies; quality assessment using MINORS	12 studies (7 in meta-analysis); 11,895 pts (3845 autologous, 8050 implants), mean BMI 33.8±1.9 kg/m^2	Autologous compared with implants: lower infection, hematoma, seroma, reconstructive failure; no difference in skin necrosis, wound dehiscence; worse deep vein thrombosis, pulmonary embolism, better BREAST-Q (but p>0.05)

Citation	Title	Topic	Search details	Review details	Number of included studies; number of patients	Results or conclusions
Diabetes						
Liu, 2022 [74]	Impact of diabetes on outcomes in breast reconstruction: A systematic review and meta-analysis	Complications in pts with diabetes	PubMed, Embase, and MEDLINE from inception to Nov 1, 2020<br>Included comparative studies only (prospective observational or retrospective cohort studies and case-control studies)	Conducted in accordance with PRISMA, registered on PROSPERO	38 studies in meta-analysis<br>151,585 pts, including 9299 with diabetes	• Overall complications 11.6% vs. 5.6%, OR=2.04, p<0.0001 [35 studies]; in subset with quality score >9: 10.2% vs. 3.5%<br>• Surgical complications 7.7% vs. 3.3%, OR=2.23, p<0.0001 [15 studies]; in subset with quality score >9: 7.8% vs. 2.5%<br>• Implant loss/flap failure 2.5% vs. 1.6%, OR=1.68, p=0.0003 [10 studies]<br>• Infection 6.8% vs. 2.5%, OR=3.88, p<0.0001 [4 studies]<br>• Skin necrosis 23.8% vs. 6.5%, OR=2.82, p=0.001 [4 studies]<br>• Length of hospital stay >5 days: 41.0% vs. 34.7%, OR=1.31, p<0.01 [3 studies]
Mortada, 2023 [75]	The impact of diabetes mellitus on breast reconstruction outcomes and complications: A systematic literature review and meta-analysis	Complications in pts with diabetes	PubMed, MEDLINE, Cochrane until Jan 2022<br>Included RCTs, prospective or retrospective cohort/comparative, case-control, or case series	Followed PRISMA, Cochrane review methods, registered in PROSPERO	43 studies in qualitative synthesis 19,731 diabetes, 197,812 without<br>Subset of 5 studies in meta-analysis; 13,293 diabetic and 114,845 non-diabetic	No difference in risk of total flap loss (RR=1.04, p=0.892), wound infection (RR=1.25, p=0.579), or total flap complications (RR=1.17, p=0.417); higher wound dehiscence (RR=2.18, p<0.0001)<br>Other complications only reported in 1 or 2 studies and therefore not included in meta-analysis:<br>• Donor site hernia/abdominal bulge 3.8% vs. 4.1%<br>• Abdominal flap necrosis at donor site 3.8% vs. 2.0%<br>• Mastectomy flap necrosis 5.6% vs. 5.5%<br>• Seroma 9.1% vs. 9.8%<br>• Flap hematoma 2.1% vs. 4.4%<br>• Flap thrombosis 1.2% vs. 0.9%<br>• Partial flap loss 0.4% vs. 0.4%<br>There is correlation between diabetes and impaired wound healing; data support using caution and clinical reasoning, but that diabetes is not a contraindication to reconstruction
Smoking						
Theocharidis, 2018 [76]	Current evidence on the role of smoking in plastic surgery elective procedures: A systematic review and meta-analysis	Role of smoking in facelift, abdominoplasty, breast reduction, breast reconstruction	PubMed and Cochrane January 1950 to October 2016	Conducted according to PRISMA	26 for breast reconstruction<br>21,639 pts divided as 1841 smokers and 19,798 non-smokers	Pts with breast reconstruction, ever smokers vs. non-smokers:<br>• Postoperative complications OR=1.91, 95% CI=1.69-2.17;<br>• Donor site complications OR=1.59, 95% CI=1.27-1.99, p<0.001 Infection OR=1.66, 95% CI=1.05-2.63, p=0.03<br>• Fat necrosis OR=1.62, 95% CI=1.06-2.48, p=0.024<br>• No significant difference in hematoma, seroma, mastectomy flap necrosis, reoperation

Citation	Title	Topic	Search details	Review details	Number of included studies; number of patients	Results or conclusions
					22,647 pts when divided as 2578 ever smokers and 20,069 non-smokers	
Various Predictors						
Mrad, 2022 [77]	Predictors of complications after breast reconstruction surgery: A systematic review and meta-analysis	Effect of age, diabetes, hypertension, obesity/BMI, smoking, RT, COPD	MEDLINE and Cochrane CENTRAL from inception to March 2022<br>RCTs and observational studies	Conducted in accordance with PRISMA	33 studies (18 reported on multiple complications), over 100,000 pts	Any complication<br>• Age OR=1.01, 95% CI=0.98-1.04, p=0.47<br>• Diabetes OR=1.18, 95% CI=0.96-1.45, p=0.13<br>• Hypertension OR=1.59, 95% CI=1.23-2.05, p=0.0004<br>• Obesity OR=1.66, 95% CI=1.37-2.01, p<0.00001<br>• RT OR=2.10, 95% CI=1.33-3.31, p=0.001<br>• Smoking OR=2.43, 95% CI=1.54-3.82, p=0.0001<br>Major/re-operative complications [14 studies]<br>• Age OR=1.01, 95% CI=1.00-1.02<br>• Diabetes OR=4.54, 95% CI=1.63-12.64<br>• Hypertension OR=1.29, 95% CI=1.03-1.62<br>• Obesity OR=1.08, 95% CI=1.03-1.13<br>• RT OR=1.82, 95% CI=1.15-2.88<br>• Smoking OR=1.46, 95% CI=1.08-1.97<br>0 to 90 day readmission [2 studies]<br>• Age OR=0.99, 95% CI=0.97-1.01, p=0.33<br>• COPD OR=1.30, 95% CI=0.97-1.72, p=0.07<br>• Diabetes OR=1.59, 95% CI=1.30-1.95, p<0.00001<br>• Hypertension OR=1.65, 95% CI=1.06-2.57, p=0.03<br>• Obesity OR=2.19, 95% CI=1.65-2.91, p<0.00001<br>• RT OR=1.72, 95% CI=0.89-3.32, p=0.11<br>• Smoking OR=2.13, 95% CI=1.05-4.34, p=0.04<br>Seroma [1 study]<br>• Diabetes OR=1.51, 95% CI=1.02-2.24, p=0.04<br>Infection [4 studies]<br>• Age OR=1.03, 95% CI=0.91-1.16, p=0.67<br>• COPD OR=1.05, 95% CI=0.76-1.45, p=0.77<br>• Diabetes OR=1.44, 95% CI=0.84-2.48, p=0.19

Citation	Title	Topic	Search details	Review details	Number of included studies; number of patients	Results or conclusions
						• Hypertension OR=3.71, 95% CI=1.14-12.07,p=0.03<br>• Obesity OR=1.83, 95% CI=1.36-2.47, p<0.0001<br>• RT OR=2.67, 95% CI=0.48-14.82, p=0.26<br>• Smoking OR=1.52, 95% CI=0.98-2.36, p=0.06<br>Overall: age has no effect; diabetes worse for major complications, readmissions, seroma; hypertension worse for any complication, major complication, readmission, infection; COPD very limited data; obesity worse for any complication major complication, readmission, infection; smoking worse for any complication, major complication, readmission, RT worse for any complication, major complication, infection
Prior Surgery						
Abdominal scars						
Chung, 2021 [78]	Effects of pre-existing abdominal scar on postoperative complications after autologous breast reconstruction using abdominal flaps: A systematic review and meta-analysis	Existing abdominal scars vs. control without	Medline and Cochrane database until March 2020<br>Exclude case reports and case series <10 pts	Conducted according to PRISMA	11 studies, 2109 pts and 2792 flap transfers (1094 scars and 1698 without)	Flap complications (complete flap loss, partial flap loss, fat necrosis) 19% vs. 18%, RR=1.12, 95% CI=0.95-1.32<br>• Complete flap loss 1.6% vs. 0.9%, RR=1.36, 95% CI=0.70-2.65, p=0.36<br>Donor-site complications (seroma, infection, wound dehiscence or delayed wound healing, abdominal bulge or hernia) 19.5% vs. 14.5%, RR=1.35, 95% CI=1.13-1.62, p=0.001<br>• Delayed wound healing or dehiscence 10.3% vs. 6.0%, RR=1.83, 95% CI=1.35-2.46, p<0.0001<br>• Abdominal weakness or hernia 4.6% vs. 4.5%, RR=1.19, 95% CI=0.78-1.81, p=0.42<br>• Donor-site wound problems RR=1.83, 95% CI=1.35-2.46<br>Technical modifications may be used to overcome constraints of the previous scar<br>Careful preoperative planning based on CTA in case of uncertainty or specific concerns should be used
Abdominal surgery						
Bond, 2021 [79]	The impact of prior abdominal surgery on complications of abdominally based autologous breast	Previous abdominal surgery, excluding liposuction	PubMed, Scopus, and Web of Science until April 2020	Used PRISMA	16 articles, 4718 pts and 5723 flaps; this includes 1656 pts	Donor-site delayed wound healing RR=1.27, 95% CI=1.00-1.61<br>Flap complications of total and partial flap loss, fat necrosis, infection, reoperation; and donor site complications of seroma,

Citation	Title	Topic	Search details	Review details	Number of included studies; number of patients	Results or conclusions
	reconstruction: A systematic review and meta-analysis		Excluded case series of <10 pts		(2236 flaps) with prior surgery<br>14 retrospective cohort, 2 retrospective case-control studies	hematoma, infection, and abdominal wall morbidity had no statistically significant differences<br>Number of events for many outcomes is small; rates varied widely between studies
Augmentation surgery						
Chicco, 2021 [80]	Systematic review and meta-analysis of complications following mastectomy and prosthetic reconstruction in patients with and without prior breast augmentation	Previous breast augmentation followed by cancer diagnosis and NSM or SSM plus implants	PubMed/MEDLINE, Embase, Scopus Jan 1966-Feb 2020<br>Comparative studies only	Followed PRISMA	6 studies, 241 breasts with prior augmentation and 1441 without	Early complications: 36.7% vs. 24.8%, OR=1.57, 95% CI=0.94-2.64, p=0.09<br>Hematoma: 3.39% vs. 2.15%, OR=2.68, 95% CI=1.00-7.16, p=0.05<br>No difference in seroma, infection, skin flap necrosis, prosthesis loss<br>Late complications: 10.1% vs. 19.9%, OR=0.53, 95% CI=0.06-4.89, p=0.57<br>Overall complications 36.5% vs. 31.2%, OR=1.23, 95% CI=0.76-2.00, p=0.40
Oncologic factors						
Neoadjuvant chemotherapy						
Varghese, 2021 [81]	A systematic review and meta-analysis on the effect of neoadjuvant hemotherapy on complications following immediate breast reconstruction	Neoadjuvant chemotherapy; immediate implant or autologous reconstruction	PubMed, Embase, Cochrane Library, 1995 to Sept 2, 2020	According to PRISMA guidelines<br>Registered in PROSPERO<br>Comparative studies (all observational); studies used appropriate matching of patients in both arms	17 studies with 3249 pts; including 575 NACT and 2674 without NACT	Overall complications: RR=0.91, 95% CI=0.74-1.11, p=0.34<br>Flap loss: RR=0.94, 95% CI=0.46-1.94, p=0.87<br>Implant/expander loss: RR=1.54, 95% CI=1.04-2.29, p=0.03<br>Hematoma (RR=0.99, p=0.97), wound complications (RR=1.15, p=0.22) no significant difference<br>Delay in adjuvant therapy RR=1.59, 95% CI=0.66-3.87, p=0.30

Citation	Title	Topic	Search details	Review details	Number of included studies; number of patients	Results or conclusions
Antiestrogen therapy						
Spera, 2020 [82]	Perioperative use of antiestrogen therapies in breast reconstruction: A systematic review and treatment recommendations	Current use of antiestrogens	MEDLINE, PubMed, and EBSCO Host from Dec 1977 to May 2018<br>Only observational studies because no RCTs were found	Reported in agreement with PRISMA; review is compliant with Cochrane Handbook for Systematic Reviews of Interventions, evaluated studies using MINORS	7 studies with 3248 pts and 4086 flaps<br>3074 without hormone modulators, 676 with SERM, 367 with AIs	SERMs at the time of reconstruction vs. none:<br>• Flap loss 9.8% vs. 5.3%, RR=1.78, 95% CI=1.31-2.43, p=0.0003<br>• Donor site complications 7.4% vs. 3.1%, RR=2.33, 95% CI=1.36-4.00, p=0.0021<br>• Flap wound complications 15% vs. 21.6%, RR=1.06, 95% CI=1.01-1.10, p=0.02<br>• Venous thromboembolic events 1% vs. 0.3%, RR=0.993, 95% CI=0.983-1.00, p=0.089<br>Concurrent AI use:<br>• Flap loss 1.3% vs. 5.3%, R=0.2469, 95% CI=0.08-0.77, p=0.01<br>• Donor site complications 11.2% vs. 3.1%, RR=3.40, 95% CI=2.05-5.64, p<0.0001<br>• Flap wound complications 30.3% vs. 21.6%, RR=1.31, 95% CI=0.94-1.82, p=0.11<br>• Venous thromboembolic events 0% vs. 0.3%
Adjuvant radiotherapy (PMRT)						
Hong, 2021 [83]	The effect of previous irradiation for patients with prosthetic breast reconstruction: A meta-analysis [note: despite title, they used PMRT not previous RT]	Immediate reconstruction with implants; PMRT vs. no PMRT<br>Does not mention whether RT to expander or implant; included 1- and 2-stage implants	PubMed, Embase, Cochrane Library until March 2020	Guided by PRISMA; assessed studies using Newcastle-Ottawa Scale (NOS)	19 studies with 6757 pts<br>4 prospective, 15 retrospective	Reconstructive failure OR=2.57, 95% CI=1.55-4.26, p<0.001 [14 studies]<br>Capsular contracture OR=5.99, 95% CI=3.12-11.47, p<0.001 [11 studies]<br>Overall complications OR=2.52, 95% CI=1.68-3.79, p<0.001 [12 studies]<br>Patient satisfaction lower OR=0.29, 95% CI=0.16-0.52, p<0.001 [3 studies]<br>Worse aesthetic results OR=0.25, 95% CI=0.12-0.52, p<0.001 [3 studies]
Zugasti, 2021 [84]	The impact of adjuvant radiotherapy on immediate implant-based breast reconstruction surgical and satisfaction outcomes: A systematic review and meta-analysis	Adjuvant RT: immediate expander/implant based<br>PMRT to either expander or	PubMed until April 2021 and published in Q1-Q2 medical journals<br>Only comparative studies	Cochrane Handbook for Systematic Reviews of Interventions; MOOSE guidelines: PRISMA	14 studies with 11,958 reconstructions: 2311 PMRT and 9647 control	Early complications<br>• Surgical site infection RR=2.44, 95% CI=1.97-3.01, p<0.00001 [9 studies]<br>• Mastectomy skin flap necrosis RR=1.62, 95% CI=1.27-2.08, p=0.0001 [8 studies]<br>• Seroma and hematoma RR=1.10, 95% CI=0.85-1.43, p=0.47 [9 studies]

Citation	Title	Topic	Search details	Review details	Number of included studies; number of patients	Results or conclusions
		implant vs. no PMRT		Studies assessed with Newcastle-Ottawa Quality Assessment Form for Observational Studies		• Implant extrusion or exposure RR=3.44, 95% CI=2.18-5.43, p<0.00001 [5 studies]<br>Late complications<br>• Capsular contracture (III-IV) RR=1.64, 95% CI=1.17-2.31, p=0.004 [7 studies]<br>• Revision surgery RR=1.64, 95% CI=1.17-2.31, p=0.004 [3 studies]<br>• Reconstruction failure RR=3.32, 95% CI=2.82-3.91, p<0.00001 [12 studies]<br>Aesthetics and Satisfaction (BREAST-Q) [2 studies]<br>• Satisfaction with Breast: mean difference -11.41 (95% CI is -13.88 to -8.95), p<0.00001 [2 studies]<br>• Satisfaction with Outcome: mean difference -6.91 (95% CI is -9.47 to -4.35), p<0.00001
Pu, 2018 [15]	The role of PMRT in patients with immediate prosthetic breast reconstruction: A meta-analysis	Immediate implant<br>PMRT (to expander or implant) vs. none	PubMed, Embase, the Cochrane Library databases, Web of Science, Chinese Biomedical Database, Chinese Scientific Journals until 2016	Well-controlled cohort comparative studies (all used multivariate analysis)<br>Quality assessed by Jadad scoring system; included PRISMA flowchart	15 trials with 5314 pts: 1069 PMRT and 4245 no PMRT	Overall complications OR=3.45, 95% CI=2.62-4.54, p<0.00001 [9 studies]<br>Reconstruction failure OR=2.59, 95% CI=1.46-4.62, p=0.001 [10 studies]<br>Capsular contracture OR=5.26, 95% CI=2.73-10.13, p<0.00001 [11 studies]<br>Worse patient satisfaction with PMRT: OR=0.28, 95% CI=0.19-0.42, p<0.00001 [3 studies]
Magill, 2017 [85]	Determining the outcomes of post-mastectomy radiation therapy delivered to the definitive implant in patients undergoing one- and two-stage implant-based breast reconstruction: A systematic review and meta-analysis	Adjuvant RT to definitive implant (not expander)	MEDLINE, Embase until October 2016	PRISMA, registered with PROSPERO	7 studies with 2921 pts (520 PMRT, 2401 control)	Capsular contracture (Grade III or IV) OR=10.21, 95% CI=3.74-27.89, p<0.00001 [7 studies]<br>Revisional surgery OR=2.18, 95% CI=1.33-3.57, p=0.002 [7 studies]<br>Reconstructive failure (removal or replacement of implant) OR=2.52, 95% CI=1.48-4.29, p=0.0007 [6 studies]<br>Patient satisfaction: lower with PMRT OR=0.29, 95% CI=0.15-0.57, p=0.0003 [4 studies]<br>Cosmetic outcome: OR=0.287, 95% CI=0.11-0.67, p=0.005 [4 studies]

Citation	Title	Topic	Search details	Review details	Number of included studies; number of patients	Results or conclusions
Liew, 2021 [18]	Does post-mastectomy radiation therapy worsen outcomes in immediate autologous breast flap reconstruction? A systematic review and meta-analysis	Immediate autologous with vs. without PMRT<br>(or immediate vs. delayed with PMRT)	MEDLINE, Embase, Cochrane CENTRAL to November 2020<br>Excluded case reports and case series or <10 pts	Registered on PROSPERO; conduced in accordance with PRISMA; evaluated studies with ROBINS-I Tool	21 studies, 3817 pts: 939 immediate + PMRT, 2462 immediate without PMRT, 416 PMRT and delayed<br>5 prospective, 16 retrospective, no RCTs	Immediate with vs. without PMRT [16 studies]:<br>Fat necrosis RR=1.91, 95% CI=1.45-2.52, p<0.00001 [12 studies]<br>Secondary surgery RR=1.62, 95% CI=1.06-2.48, p=0.03 [4 studies]<br>Volume loss RR=8.16, 95% CI=4.26-15.63, p<0.00001 [4 studies]<br>Revisional operations RR=0.95, 95% CI=0.80-1.13, p=0.54<br>Infection RR=1.14, p=0.60 [8 studies]<br>Healing complications RR=1.23, p=0.17<br>Hematoma RR=1.14, p=0.81 [3 studies]<br>Seroma formation RR=1.19, p=0.67 [4 studies]<br>Total flap loss RR=0.80, p=0.81 [7 studies]<br>Partial flap loss/necrosis RR=0.34, p=0.15 [4 studies]<br>Skin contracture and hyperpigmentation higher with PMRT [2 studies]<br>Cosmetic results (observer-reported) better in 4/5 studies without PMRT, and no difference in 1/5<br>Higher risks but not necessarily clinically significant, immediate reconstruction with PMRT is still viable option

Abbreviations:

ACS/NSQIP, American College of Surgeons/National Surgical Quality Improvement Program; AIs, aromatase inhibitors (eg, anastrozole, exemestane, letrozole); BMI, body mass index; BRA, Breast Reconstruction Risk Assessment score; CI, confidence interval; COPD, chronic obstructive pulmonary disease; CTA, computed tomography angiography; DIEP, deep inferior epigastric perforator flap; MINORS, methodological index for non-randomized studies; MOOSE, Meta-analysis Of Observational Studies in Epidemiology; NACT, neoadjuvant chemotherapy; NOS, Newcastle-Ottawa Scale; NSM, nipple-sparing mastectomy; OR, odds ratio; PMRT, postmastectomy radiotherapy; PRISMA, Preferred Reporting Items for Systematic Reviews and Meta-Analyses; PROSPERO, International Prospective Register of Systematic Reviews, University of York, UK; pts, patients; RCT, randomized controlled trial; ROBINS-I, Risk of Bias In Non-randomized Studies of Interventions; RR, risk ratio; RT, radiotherapy; SERMs, selective estrogen receptor modulators (e.g., tamoxifen, raloxifene); SSM, skin-sparing mastectomy

Back to Start of Tables

Table S3. Effect of Age on Reconstructive Outcomes

Study	Study name or database and location	Years of study	Topic or comparison	Age, # pts	Pt or study details	Statistical design/comparison	Outcomes
Prospective Study							
Santosa, 2016 [88]	MROC; USA (9 centres) and Canada (2 centres)	2012-2014	Effect of age on outcomes	>60, n=234<br>45-60, n=803<br><45, n=494	Prospective<br>Age ≥18 y; implant or autologous, ≥2 y follow-up after reconstruction	Mixed-effects regression models: independent variables with statistically significant effects in the univariate analysis (p <0.05) or known predictors of postoperative complications (BMI, laterality, and smoking status) included	Complications, > 60 y and 45-60 y compared to <45 y as reference group<br>Any complication OR=1.46, 95% CI=0.99-2.15, p=0.059; OR=1.23, 95% CI=0.93-1.62, p=0.140<br>Major complication OR=1.43, 95% CI=0.93-2.18, p=0.101; OR=1.16, 95% CI=0.85-1.57, p=0.349<br>PROs (excluded pts with reconstructive failure) at 2 y post-reconstruction, age >60 compared to younger pts (<45 y):<br>Implants<br>• Satisfaction with Breasts -5.05, p=0.025<br>• Psychosocial Well-Being 3<br>• Physical Well-Being -2<br>• Sexual Well-Being 4.25, p=0.04<br>Autologous<br>• Satisfaction with Breasts 3<br>• Psychosocial Well-Being 8.21, p<0.01<br>• Physical Well-Being 6.07, p<0.01<br>• Sexual Well-Being 10.39, p<0.01
Single-institution retrospective studies							
Kim, 2024 [86]	Memorial Sloan Kettering Cancer Center, New York, NY	2017-2022	Effect of age on outcome after PMRT	4370 pts<br>>70, n=168<br>60-69, n=701<br>50-59, n=1413<br>40-49, n=1681<br><40, n=676	Retrospective<br>Age ≥18 y; autologous or 2-stage implant	Multivariable generalized estimating equation (GEE) models for longitudinal data for BREAST-Q domains<br>Multivariable logistic regression models for complications that were significantly different across age groups univariably.<br>Age was a continuous predictor. Covariates were selected based on a hypothesized relationship with the outcome	Complications: mastectomy skin flap/nipple necrosis, infection, seroma higher in older pts. With age as continuous variable, OR per year of age increase:<br>• Mastectomy skin flap/nipple necrosis OR=1.02, 95% CI=1.01-1.03, p<0.001<br>• Infection OR=1.01, 95% CI=1.00-1.03, p=0.038<br>• Seroma OR=1.02, 95% CI=1.00-1.03, p=0.024<br>• Hematoma OR=1.01, 95% CI=1.00-1.03, p=0.14<br>PROs by BREAST-Q using multivariable generalized estimating equations (GEE) models for repeated measures with age at surgery as continuous variable

Study	Study name or database and location	Years of study	Topic or comparison	Age, # pts	Pt or study details	Statistical design/comparison	Outcomes
							• Satisfaction with breasts lower in older pts (ß= −0.06, 95% CI= −0.12 to −0.01, p=0.033)<br>• Psychosocial Well-Being higher in older pts (ß= 0.14, 95% CI=0.09 to 0.20, p<0.001)<br>• No difference in Physical Well-Being of Chest (ß= −0.03, 95% CI= -0.08 to 0.02, p=0.2)<br>• No difference in Sexual Well-Being (ß= −0.04, 95% CI= −0.11 to 0.02, p=0.2)
Honig, 2024 [87]	University of Pennsylvania, Philadelphia, Pennsylvania	2005-2018	Free flap reconstruction and risk at greater age	2598 pts; median age 51y<br>>75, n=19<br>70-74, n=68<br>65-69, n=164<br>60-64, n=327<br>55-59, n=394<br><55, n=1625	Retrospective	Risk-adjusted logistic regression models controlling for demographics (race, body mass index, smoking history) and comorbidities (diabetes, pulmonary disease, hypertension, and vascular disease) to determine association of age and outcomes.<br>Analysis modelled age over a continuous spectrum instead of discrete categories; outcomes reported at 5-y intervals from median age (51 y) of cohort	• After multivariate analysis, age in 5-year categories, age 70-74 vs age <55; p values appear to be based on trend over full age range<br>• Delayed healing 35.7% vs 29.2%, p=0.036<br>• Skin necrosis 15.3% vs 10.7%, p=0.039<br>• Hematoma 9.3% vs 5.3%, p=0.005<br>• Venous thromboembolism 2.1% vs 1.4%, p=0.381<br>• Flap loss 2.2% vs 2.5%, p=0.574<br>• Seroma 9.1% vs 7.6%, p=0.548<br>• Infection 6.9% vs 6.8%, p=0.588<br>• Multiple surgical complications 10.3% vs 8.0%, p=0.364<br>Risk of hematoma, delayed healing, and skin necrosis increase ≈ 0.2% per year. No increase in VTE or free flap loss. Authors concluded that age cutoff does not appear warranted
Chang, 2011 [89]	UCLA Medical Center	2002-2009	Microvascular reconstruction in pts with advanced age	650 pts, 818 reconstructions:<br>≥60 n=122<br>60-69, n=103<br>70+, n=19<br><50, n=411<br>50-59, n=285	Retrospective<br>Microvascular free-flap reconstruction	X^2 analysis with a trend test for comorbidities and outcomes; multivariate analysis using logistic regression to evaluate age, BMI, previous surgeries	Surgical complications:<br>26.2% age 60-69<br>31.6% age 50-59<br>29.0% age <50<br>Age was not predictive of surgical complications when used as a continuous variable
Multi-institution Database Studies							
Cuccolo, 2020 [90]	ACS-NSQIP	2005-2016	Age or frailty and pedicled flaps, all types of surgery (not just breast)	Breast:<br>≥80, n=31<br>70-79, n=377<br>60-69, n=1834	Retrospective<br>Complications only recorded for 30 days post-operation	Variables with p<0.05 on univariate analysis included in multivariable binary logistic regression<br>Age appears to be a continuous variable in multivariable	For breast, effect of age on complications (30 days postoperatively); rates per each additional year of age<br>All-cause complications<br>Authors concluded that the effect of age does not have strong predictive power and may not have clinical relevance on its own

Study	Study name or database and location	Years of study	Topic or comparison	Age, # pts	Pt or study details	Statistical design/comparison	Outcomes
				50-59, n=3084<br>18-49, n=3199		analysis, as discussion indicates OR gives increase in risk per one additional year of age	Frailty index had much stronger predictive capacity than age
Butz, 2015 [91]	ACS-NSQIP	2005-2012	30-day complications after reconstruction with advanced age	≥65, n=1624<br><65, n=10,140	Retrospective<br>Complications limited to those within 30 days follow-up (common medical and surgical perioperative complications)	Multivariable linear and logistic regression to control for demographics and comorbidities	Complications, compared to age <65: overall, unadjusted: 6.8% vs 5.2%<br>With multivariate adjustment, by reconstruction type<br>Implants:<br>• Any complication 6.1% vs 4.2%, aOR=1.16, 95% CI=0.85-1.57, p=0.346<br>• Surgical-site infection 3.5% vs 2.5%, aOR=0.98, 95% CI=0.66-1.47, p=0.929<br>• Return to operating room 7.0% vs 6.8%, aOR=0.90, 95% CI=0.68-1.18, p=0.440<br>• Other complications no significant difference though too few to meet our criteria for multivariate analysis<br>Autologous:<br>• Any complication 10.1% vs 8.9%, aOR=1.16, 95% CI=0.71-1.88, p=0.555<br>• Individual complications too few for multivariate analysis, although venous thromboembolism appears elevated (2.2% vs 0.8%, aOR=3.67, 95% CI=1.20-11.22, p=0.023)

Abbreviations:

ACS-NSQIP, American College of Surgeons-National Surgical Quality Improvement Program; BMI, body-mass index; CI, confidence interval; DIEP, deep inferior epigastric perforator flap; MROC, Mastectomy Reconstruction Outcomes Consortium; NSQIP, National Surgical Quality Improvement Program; OR, odds ratio; PMRT, post-mastectomy radiation therapy; PRO, patient-reported outcome; pts, patients

Back to Start of Tables

Table S4. Question 2: Immediate versus Delayed

Citation	Study name and location	Years of study	Topic or comparison	Number of patients	Patient characteristics	Design	Results	Other
Immediate vs. Delayed								
Jeevan, 2014 [92]; Jeevan, 2011 [93]	England, Wales, Scotland	2008-2009	Audit of provision of mastectomy and reconstruction (immediate, delayed) and associated complications and QoL<br>Patients with mastectomy + immediate reconstruction or with delayed reconstruction (mastectomy was previously) from Jan 2008 to March 2009	Operative/clinical data for 3,389 (21%) immediate; 1,731 delayed (after previous mastectomy); 13,096 no reconstruction<br>6,882 responses to 3-month questionnaire (1,553 immediate, 692 delayed, 4,637 none)<br>7,110 responses to 18-month questionnaires<br>3,304 immediate: 1207 (36.5%) expander/implant, 722 (21.9%) pedicle flap + implant, 920 (27.8%) pedicle flap, 455 (13.8%) free flap<br>1699 delayed: 280 (16.5%)	Breast cancer or DCIS, unilateral mastectomy, or primary reconstruction in 2008-2009<br>Pts without reconstruction were older and more frail, higher BMI and other comorbidities<br>Most immediate reconstruction was an implant (with or without a flap), while majority of delayed reconstruction used only a flap<br>In delayed reconstruction, 14% implant-only pts and 45% with flaps had RT; RT use in immediate reconstruction not reported<br>Proportion invasive and DCIS not equivalent: invasive 2311 vs. 1347; DCIS 927 vs. 231<br>Immediate group had less positive lymph nodes	Prospective cohort; national audit; data from charts and questionnaires (subset of BREAST-Q) sent to pts (3 months and 18 months after surgery)<br>Multiple logistic regression to calculate risk-adjusted procedure rates by Cancer Networks and risk-adjusted outcomes by type of surgery and considered age, smoking status, BMI, co-morbidities, ASA grade and ECOG score, and previous treatments (e.g., adjuvant RT or chemotherapy)<br>QoL adjusted for age, deprivation index, performance status, smoking status, postoperative chemotherapy or RT<br>QoL not adjusted for baseline scores	Percent of pts offered immediate reconstruction varied from 24% to 75% in different Networks (regions)<br>Authors state (data not reported) that inpatient mastectomy site complications, adjusted for pt characteristics, did not vary between immediate and no reconstruction; delayed reconstruction had less mastectomy site complications [this may be due to mastectomy being completed previously and associated complications not accounted for in this study].<br>Adjusted risks of implant-related complications were not statistically associated with type of reconstruction or timing (immediate vs. delayed)<br>The risk of flap-related complications varied with flap type but was not associated with timing of procedure<br>18-month adjusted QoL scales [Satisfaction with Breast Appearance, Emotional/Psychosocial Well-Being, Physical Well-Being, Sexual Well-Being], immediate vs. delayed scores<br>• Implant only: breast appearance 54 vs. 56; emotional 65 vs. 66; physical 74 vs. 76; sexual 44 vs. 49<br>• Pedicle with implant: breast appearance 63 vs. 68; emotional 72 vs. 76; physical 75 vs. 76; sexual 50 vs. 58	Patient-reported complications in 3 months after discharge do not appear to be adjusted and therefore data not extracted<br>Two different questionnaires were used (mastectomy alone; immediate or delayed reconstruction)<br>Size of clinically significant differences in Breast Q scales were not reported; Voineskos et al. proposed that 4 points is a clinically useful minimal important difference score [64]<br>Authors indicate that results "should not be interpreted as indicating the relative effectiveness of the various procedures. Each procedure is associated with distinct and different treatment pathways. The choice of operation is likely to reflect women's views, values and expectations" (Jeevan, 2011, page 8 [93]). There may also be a response shift if values/views change such

Citation	Study name and location	Years of study	Topic or comparison	Number of patients	Patient characteristics	Design	Results	Other
				expander/implant, 432 (25.4%) pedicle flap + implant, 433 (25.5%) pedicle flap, 554 (32.6%) free flap			• Autologous pedicle flap: breast appearance 64 vs. 69; emotional 70 vs. 74; physical 73 vs. 77; sexual 49 vs. 56<br>• Free flap: breast appearance 63 vs. 73; emotional 70 vs. 78; physical 75 vs. 80; sexual 49 vs. 63	as during wait for delayed reconstruction
Yoon, 2018 [63]<br>See also Wilkins, 2018 [66]; Kulkarni, 2017 [65]	The Mastectomy Reconstruction Outcomes Consortium (MROC) Study, 11 institutions (9 USA and 2 Canada)<br>NCT01723423	2011-2017	Complications and PROs of immediate vs. delayed postmastectomy reconstruction	4436 pts in full study; this publication 1806 immediate vs. 151 delayed (complete data for 1639 and 147 pts)<br>Implants: 1275 immediate vs. 27 delayed<br>Latissimus Dorsi: 45 immediate vs. 18 delayed<br>Autologous: 486 immediate vs. 106 delayed	Breast cancer or prophylaxis; primary (first-time) reconstruction; 1 or 2-stage implant or autologous<br>For expander-implant pts they had to be at least 3 months after expander-implant exchange<br>Exclude previous augmentation, reduction, or reconstruction<br>Exclude from PRO analysis if reconstruction failure<br>2-y complication and PRO data available	Prospective, multicentre cohort design; followed STROBE guidelines for cohort studies<br>Each subcategory of complication was separately modelled using a mixed-effects logistic regression model adjusted for age, race, BMI, procedure type, diabetes, laterality, radiation, chemotherapy, and lymph node management<br>BREAST-Q, PROMIS, EORTC QLA-BR23 surveys before and 1 and 2 years postoperatively; 1 year data not included in publication<br>Medical records reviewed 1 and 2 years after reconstruction; major complications defined as those requiring re-	Significant baseline differences<br>Comparison of immediate vs. delayed after controlling for clinical covariates: any complications OR=2.63 ($p<0.001$), major complications OR=1.92 ($p=0.016$)<br>Preoperative (before reconstruction) PROs: Delayed patients scored significantly lower on most subscales (except depression)<br>2 years postoperatively: no statistically significant differences in adjusted PROs for immediate vs. delayed<br>BREAST-Q for Patient Satisfaction, Psychosocial, Sexual, Physical Well-Being; PROMIS for physical function, anxiety, depression, fatigue, sleep disturbance, satisfaction in social roles, pain interference; EORTC for body image and sexual functioning	Primary outcome: change from baseline in HRQoL using PROs

Citation	Study name and location	Years of study	Topic or comparison	Number of patients	Patient characteristics	Design	Results	Other
						hospitalization or re-operation; failure defined as complications requiring implant or flap removal		
Wilkins, 2018 [66]	The Mastectomy Reconstruction Outcomes Consortium (MROC) Study, USA (9 institutions) and Canada (2 institutions);<br>NCT01723423	2011-2017	Complications and PROs of postmastectomy reconstruction	4436 pts in full study; 2234 for complications analysis: 2076 immediate vs. 158 delayed	Breast cancer or prophylaxis; primary reconstruction; implant or autologous	Prospective, multicentre cohort design; mixed effects logistic regression	Immediate vs. delayed<br>Any complications OR=1.82, 95% CI=1.11-2.99, p=0.017<br>Major complications OR=1.17. 95% CI=0.68-2.00, p=0.566 [note difference compared with results in Yoon, 2018]	
Kulkarni, 2017 [65]	The Mastectomy Reconstruction Outcomes Consortium (MROC) Study, USA (19institutions) and Canada (2 institutions);<br>NCT01723423	2011-2017	Pain after postmastectomy reconstruction and patient-specific factors	2667 pts; 2487 immediate and 180 delayed	Breast cancer or prophylaxis; primary reconstruction; implant or autologous	Prospective, multicentre cohort design; mixed effects logistic regression	BREAST-Q Physical Well-Being Chest and Upper Body scale: immediate reconstruction was related to lower Physical Well-Being at 1 week post operation (p<0.0001)<br>No significant difference for immediate vs. delayed for postoperative MPQ-affective pain (p=1.00); postoperative MPQ-sensory pain (p=0.46); postoperative NPRS pain score p=0.17	
Knoedler, 2024 [94]	ACS-NSQIP database (risk-adjusted, case-mix adjusted, outcomes-based registry)	2008-2021	Immediate vs. delayed for implant (one stage) or autologous reconstruction<br>Patients with breast cancer	21,560 pts<br>11,237 implant (9791 immediate, 1446 delayed)<br>10,323 autologous (8378	Breast cancer, by ICD codes; implant-based or autologous reconstruction<br>Excluded immediate-delayed (use of expander) or 2-stage expander-implant	30-day outcomes were in database<br>For broad categories (general, surgical, medical, or any complication) compared immediate vs. delayed by 2 methods: (a) with PSW estimated by logistic	Complications, immediate vs. delayed, implants<br>• Any (PSW) OR=2.41, 95% CI=1.69-3.44, p<0.001<br>• Any (MV) OR=2.56, 95% CI=1.84-3.65, p<0.0001<br>• General (PSW) OR=2.48, 95% CI=1.68-3.68, p<0.0001<br>• General (MV) OR=2.68 95% CI=1.86-3.98, p<0.0001	Complications<br>General=30-day mortality, reoperation, readmission, unplanned readmission<br>Surgical =superficial incisional infection, deep incisional infection, organ space infection, dehiscence, bleeding

Citation	Study name and location	Years of study	Topic or comparison	Number of patients	Patient characteristics	Design	Results	Other
			according to CPT codes	immediate, 1945 delayed)	Pt characteristics similar for immediate vs. delayed except as follows:<br>A) for implants: ethnicity because immediate has 21% unknown; tumour type (22% vs. 4.1% in situ and 1.7% vs. 38% unknown); ASA class (22% vs. 28% severe); disseminated cancer 22% vs. 1.7%<br>B) for flaps: tumour type (23% vs. 2.7% in situ and 2.6 % vs. 28% unknown); ASA class (30 vs. 39% severe)<br>Larger differences between implant and flap groups for obesity (26% vs. 40%), ASA group (noted above), disseminated cancer (1.9% in flap groups)<br>SSLNB and ALND were rare in delayed cases (<2%) but high in immediate (52% and 18% implants; 47% and 16% flaps)<br>Type of surgeon (general or plastic) for implants was 30% plastic surgeon for immediate and 97% for delayed; for flaps was 50% plastic surgeon for immediate and 93% for delayed	regression and adjusted for confounders to calculate casual ORs for overlap population (pts eligible for both immediate and delayed); (b) univariable and MV regression adjusting for confounders<br>Authors indicate that "adjusted ORs for other risk factors were obtained from the same analysis" but provide no details	• Surgical (PSW) OR=3.66, 95% CI=2.11-6.36, p<0.0001<br>• Surgical (MV) OR=3.40, 95% CI=2.10-5.75, p<0.0001<br>• Medical (PSW) OR=1.26, 95% CI=0.39-4.01, p=0.70<br>• Medical (MV) OR=1.43, 95% CI=0.49-4.97, p=0.53<br><br>Complications, immediate vs. delayed, flaps<br>• Any (PSW) OR=1.28, 95% CI=1.09-1.50, p=0.003<br>• Any (MV) OR=1.29, 95% CI=1.10-1.52, p=0.002<br>• General (PSW) OR=1.37, 95% CI=1.12-1.67, p=0.002<br>• General (MV) OR=1.38 95% CI=1.14-1.69, p=0.001<br>• Surgical (PSW) OR=1.13, 95% CI=0.93-1.36, p=0.21<br>• Surgical (MV) OR=1.13, 95% CI=0.94-1.37, p=0.19<br>• Medical (PSW) OR=1.15, 95% CI=0.77-1.71, p=0.49<br>• Medical (MV) OR=1.08, 95% CI=0.74-1.61, p=0.70<br><br>Complications, immediate vs. delayed (any type)<br>• Any (PSW) OR=1.33, 95% CI=1.16-1.52, p<0.0001<br>• Any (MV) OR=1.39, 95% CI=1.20-1.60, p<0.0001<br>• General (PSW) OR=1.52, 95% CI=1.28-1.80, p<0.0001<br>• General (MV) OR=1.56 95% CI=1.32-1.86, p<0.0001	Medical =pneumonia, reintubation, pulmonary embolism, ventilator >48h, renal insufficiency, urinary tract infection, cerebral vascular accident/stroke, myocardial infarction, deep vein thrombosis, sepsis, septic shock<br>Notes:<br>• Database did not have data on hematoma or seroma<br>• Only has complication up to 30 days and therefore no information on capsular contraction, aesthetics, sensation, oncologic outcomes<br>• While immediate reconstruction has higher complications this may not all be clinically relevant, or may be offset by better aesthetics and body image in short term (and less anxiety and depression)<br>• Database does not have information on mastectomy and reconstruction technique/approach, or use of neoadjuvant and adjuvant chemotherapy or RT<br>• Delayed reconstruction requires 2 operations,

Citation	Study name and location	Years of study	Topic or comparison	Number of patients	Patient characteristics	Design	Results	Other
					Impatient setting for implants was 49% for immediate 6.2% for delayed; for flaps was 95% for immediate and 91% for delayed		• Surgical (PSW) OR=1.19, 95% CI=1.01-1.41, p=0.04<br>• Surgical (MV) OR=1.23, 95% CI=1.04-1.46, p=0.02<br>• Medical (PSW) OR=1.05, 95% CI=0.72-1.51, p=0.82<br>• Medical (MV) OR=1.02, 95% CI=0.71-1.49, p=0.92<br><br>While not compared statistically and not adjusted for confounders, implant-based reconstruction had lower rates of complications than autologous reconstruction; this may be due to higher rates of obesity and more complex operations for autologous reconstruction and factors such as breast size that were not recorded	and it is unclear whether complications of each were added together; for any/general/surgical/medical complications, all were higher in immediate group; this may reflect a combination of mastectomy + reconstruction complications being captured
Autologous Reconstruction								
Kroll, 1995 [95]	The University of Texas M.D. Anderson Cancer Center, Houston, Texas<br><br>One surgeon	1985-1993	Aesthetic results for immediate vs. delayed	237 pts (267 breasts); 104 immediate vs. 133 delayed	Breast cancer with TRAM flap reconstruction	Aesthetic outcomes based on postoperative photographs; scored by 9 judges (no plastic surgeons) from 1 (poor) to 4 (excellent); multiple regression analysis	Aesthetic scores: 3.25 immediate (mostly SSM) vs. 2.82 delayed (p=0.0001); after multiple regression analysis p=0.0001	Immediate reconstruction may have better results because of associated use of SSM
Fosnot, 2011 [96]	University of Pennsylvania School of Medicine, Philadelphia, PA<br><br>3 reconstructive surgeons	2005-2009	Effect of RT on vascular complications in autologous free flap, prior RT vs. no RT	226 prior RT (109 delayed), 799 no prior RT (95 delayed)	Free flap breast reconstruction<br><br>Preoperative RT include RT to chest wall and axilla on operative side for previous BCS or Hodgkin disease<br><br>RT group had more dyslipidemia, neoadjuvant	Retrospective<br><br>Multiple binary logistic regression	Final logistic regression included hypertension, dyslipidemia, preoperative chemotherapy, preoperative XRT, relayed reconstruction, initial target (IMA)<br><br>Previous RT group had more vascular problems 17.3% vs. 9.6%, p=0.001; in final logistic regression model OR=1.68, 95% CI=1.04-2.70, p=0.04<br><br>Delayed reconstruction had more vascular complications, 15.2% vs. 10.4%,	

Citation	Study name and location	Years of study	Topic or comparison	Number of patients	Patient characteristics	Design	Results	Other
					chemotherapy, delayed reconstruction		p=0.05; in final regression model OR=1.14, 95% CI=0.69-1.91, p=0.61	
Prantl, 2020 [17]	22 German cancer centres<br><br>Data from German Society of Plastic, Reconstructive and Aesthetic Surgeons registry on free flap breast reconstruction	2011-2019	Immediate vs. delayed autologous DIEP free flap	• 3926 pts with 4577 reconstructions<br>• 897 pts (1136 flaps) immediate<br>• 3016 pts (3441 flaps) delayed	Breast cancer; initial or salvage DIEP flaps<br><br>No significant differences regarding perioperative risk factors such as BMI, comorbidities (diabetes mellitus, coagulopathy), or smoking<br><br>Immediate group had more family history and genetic disposition (and more bilateral reconstruction) and less chemotherapy and neoadjuvant RT<br><br>Delayed group had 29.4% with complications after other reconstruction such as implants (1.1% in immediate group)<br><br>Transcutaneous doppler probe used for flap monitoring in 19.3% vs. 53.1% of pts	Retrospective study of pts in prospective German database containing follow-up data for 3 months<br><br>No multivariate analysis but similar risk factors	Medical complications 6.6% vs. 6.4%, p=0.777<br><br>Partial free-flap loss 1.0% vs. 1.2%, p=0.706<br><br>Total free-flap loss 2.3% vs. 1.9%, p=0.516<br><br>Revision surgery 7.7% vs. 9.8%, p=0.039<br><br>Day 1 postoperative mobilization 82.1% vs. 68.7%, p<0.001<br><br>Mean length of hospital stay 7.3 days vs. 8.9 days, p<0.001	
Beugels, 2018 [19]<br><br>Overlaps with Joosen, 2021 [97] [recurrence outcomes]	Maastricht University Medical Center in the Netherlands and 2 community hospitals (VieCuri Medical Center, Venlo, and	2010-2017	Complications after immediate vs. delayed DIEP flap reconstruction	737 pts, 910 DIEP flaps; 397 immediate and 513 delayed<br><br>Immediate 61.2% oncologic reasons; delayed 88.3%	All pts with DIEP flap reconstruction<br><br>Excluded stacked flaps and mixed bilateral (immediate and delayed) reconstruction<br><br>Pts with preoperative or high chance of PMRT were advised to have delayed;	Median follow-up 9 vs. 10 months<br><br>ORs were corrected for unilateral vs. bilateral, university vs. community, BMI, smoking, RT, chemotherapy, endocrine therapy in multivariable models	Primary outcome was major (total or partial flap loss, venous congestion)and minor (infection, hematoma, seroma, fat necrosis, wound problems including dehiscence and superficial skin necrosis related to reconstruction but not mastectomy flap) recipient-site complications<br><br>Patients with breast cancer, adjusted data:	Immediate group had more seroma and hematoma, and less wound problems

Citation	Study name and location	Years of study	Topic or comparison	Number of patients	Patient characteristics	Design	Results	Other
	Zuyderland Medical Center, Sittard-Geleen)			oncologic reasons<br><br>Subset with breast cancer: 243 immediate, 453 delayed	rest were eligible for immediate reconstruction<br><br>SSM used with immediate reconstruction<br><br>Immediate pts more likely to have genetic predisposition, lumpectomy in medical history and more likely to have prophylactic mastectomy<br><br>Delayed pts more likely to have breast cancer and therefore RT, chemotherapy, endocrine therapy	Location of deep inferior epigastric artery perforators with hand-held Doppler device; preoperative imaging with magnetic resonance angiography of the abdomen for patients treated at university hospital; used internal mammary vessels in all cases; flaps monitored after reconstruction with Doppler signals, colour, capillary refill	Major complications 7.8% vs. 9.9%, aOR=0.75, p=0.347<br>• total flap loss 0.8% vs. 2.4%, aOR=0.30, p=0.139<br>• partial flap loss 3.3% vs. 4.9%, aOR=0.61, p=0.311<br>• venous congestion 4.1% vs. 3.8%, aOR=1.11, p=0.804<br><br>Minor complications 25.5% vs. 24.7%, aOR=1.18, p=0.391<br>• Infection 7.4% vs. 7.7%, aOR=1.12, p=0.739<br>• Hematoma 10.7% vs. 3.1%, aOR=3.68, p<0.001<br>• Seroma 2.9% vs. 0.7%, aOR=8.32, p=0.003<br>• Fat necrosis 11.1% vs. 12.8%, aOR=0.95, p=0.831<br>• Wound problems 7.8% vs. 16.3%, aOR=0.52, p=0.020	
Joosen, 2021 [97]<br><br>Overlaps with Beugels, 2018 [19] [complications]	Maastricht University Medical Center and two community hospitals (VieCuri Medical Center Venlo and Zuyderland Medical Center Sittard), The Netherlands<br><br>10 plastic surgeons	2010-2018	Recurrence after immediate vs. delayed DIEP flap reconstruction	862 pts, 919 DIEP flaps; 347 immediate and 572 delayed	Diagnosis of breast cancer;<br><br>Excluded prophylactic mastectomy, metastasis at time of mastectomy, recurrence prior to reconstructive surgery<br><br>Immediate group had greater history of lumpectomy (30.5% vs. 15.4%), less history of expander/implant (1.2% vs. 29.0%), less oncological treatment (RT 32.3% vs. 47.0%; chemotherapy 49.1% vs. 68.8%; endocrine therapy 41.7% vs. 54.9%), lower	Retrospective cohort study<br><br>Analyzed on per-patient basis, Cox proportional hazards (multivariable) regression analysis<br><br>Median follow-up from mastectomy 46 vs. 86 months	LR 1.5% vs. 1.7%, HR=0.804, p=0.400; adjusted HR=2.890 (95% CI=1.536-5.437, p=0.001)<br><br>Regional recurrence 3.7% vs. 2.4%, HR=0.306, p<0.001; adjusted HR=0.912 (95% CI=0.627-1.327), p=0.631<br><br>DM 2.8% vs. 6.9%, HR=1.351, p=0.085; adjusted HR=5.244, 95% CI=3.395-8.102, p<0.001	Local and regional recurrence results switch in direction from HR to adjusted HR<br><br>Note: there were <25 events for LR and 25 for regional recurrence and therefore these outcomes do not meet our criteria for conducting multivariate analysis

Citation	Study name and location	Years of study	Topic or comparison	Number of patients	Patient characteristics	Design	Results	Other
					stage disease (stage II-IV 26.0% vs. 45.0%)			
Shammas, 2023 [98]	Duke University, Durham, NC	2014-2018	Free flaps, immediate vs. delayed vs. staged	3310 pts, 2310 (69.8%) immediate, 388 (11.7%) delayed, 612 (18.5%) staged (delayed-immediate using tissue expander at time of mastectomy)	Breast cancer diagnosis, mastectomy, eventual free-flap reconstruction (based on ICD-9, ICD-10, or Current Procedural Terminology codes)<br>Delayed indications include uncertain need for RT, medical comorbidities, tenuous mastectomy skin flaps, pending final decision on type of breast reconstruction<br>Excluded distant metastasis, implants, expander prior to mastectomy or after time of initial mastectomy<br>Group differences: bilateral mastectomy 37.0% vs. 19.6% vs. 28.9%; Lymph node surgery 61.0% vs. 51.8% vs. 70.3%; chemotherapy 33.2% vs. 55.8% vs. 57.4%; RT 5.7% vs. 49% vs. 40%; ADM 8.5% vs. 9.5% vs. 73.5%	Retrospective using IBM MarketScan commercial claims and encounters and Medicare supplemental databases<br>Complications 90 days after mastectomy and 90 days after free-flap procedure<br>Modified Poisson regression adjusting for several factors (adjusting for age, comorbidity index, urban/rural ZIP code, geographic region, employment status, laterality of mastectomy, lymph node surgery at the time of mastectomy, chemotherapy before or after mastectomy, radiation before or after mastectomy, other cancer diagnoses, use of ADM)	Surgical complications 32.0% vs. 42.3% vs. 57.8%<br>Systemic complications 10.4% vs. 11.9% vs. 17.5%<br>Any complication 38.4% vs. 46.9% vs. 64.2%<br>Adjusted RR for at least one complication: immediate vs. delayed RR=0.78, 95% CI=0.68-0.88, p<0.001; immediate vs. staged RR=0.60, 95% CI=0.53-0.67, p<0.001; delayed vs. staged RR=0.77, 95% CI=0.67-0.88, p<0.001<br>Results in subgroup without RT: immediate vs. delayed RR=0.78, 95% CI=0.66-0.90, p=0.001; immediate vs. staged RR=0.62, 95% CI=0.55-0.70, p<0.001; delayed vs. staged RR=0.80, 95% CI=0.67-0.96, p=0.020	
Huang, 2022 [99]	New York-Presbyterian/ Weill Cornell Medical Center, New York, New York	2011-2020	Autologous DIEP: complications with immediate vs. delayed-immediate (immediate expander/implant later	248 pts (443 breasts); 193 pts (344 breasts) immediate; 55 pts (99 breasts) delayed-immediate	2 cohorts were comparable in age, body mass index, and comorbidities (p>0.05); active smokers 7.3% vs. 1.8%<br>Differences in preoperative	Retrospective cohort study	Major complications: breast hematoma 3.2% vs. 2.0%, p=0.541; anastomotic failure 1.2% vs. 1.0%, p=1; partial or complete flap loss 0 vs. 0; venous thromboembolism 1.0% vs. 1.8%	Authors suggest lower rate of skin necrosis in delayed group may be due to additional time to revascularize and heal after mastectomy<br>Based on their experience, should wait minimum 6

Citation	Study name and location	Years of study	Topic or comparison	Number of patients	Patient characteristics	Design	Results	Other
	2 plastic surgeons and several breast surgeons; initial mastectomy often performed elsewhere		exchanged for flap)		(reconstructive) therapies (chemotherapy 16.6% vs. 67.3%, RT 15.0% vs. 54.5%, hormone therapy 10.9% vs. 72.7%, previous breast surgery 39.4% vs. 100%)<br><br>Exclude if no immediate reconstruction, device removal due to complications prior to flap reconstruction		Other complications similar except breast skin necrosis 16.0% vs. 2.0%, p<0.001	months after completing PMRT before expander-implant exchange
Marquez, 2023 [100]	ACS NSQIP data<br><br>See also Knoedler, 2024 [94] for both implant and autologous results using same database; Kalmar, 2024 [101]	2010-2020	Autologous free flaps<br><br>Cases with mastectomy according to CPT codes (did not look for cancer codes so may include prophylactic or for other reasons)	15,596 pts: 7,907 immediate; 976 delayed immediate; 6713 delayed	Limited demographic information reported (age, race, BMI, diabetes, hypertension, ASA class); unclear what BMI categories are<br><br>3 timing modalities: mastectomy and concurrent flap reconstruction; flap with removal of expander (delayed immediate); flap alone (no mention of mastectomy; delayed reconstruction)	Queried database for top 4 diagnosis at time of reoperation<br><br>Multivariate analysis using single-step multinomial logistic regression with covariates of age, race, BMI, diabetes, hypertension, ASA classification, and laterality of reconstruction	No significant difference in surgical site infections, wound dehiscence, or DVT<br><br>Increase in transfusions and more reoperations in immediate group<br><br>Using multivariate analysis: odds of surgical complications higher for immediate vs. delayed (odds of complications 1.244, p<0.001), immediate vs. delayed-immediate (1.439, p<0.001), but not delayed immediate vs. delayed (0.864, p=0.223)	Authors note for immediate reconstruction that "it is difficult to distinguish between complications related to the mastectomy versus those related to reconstructive efforts"<br><br>High proportion of delayed in this study<br><br>Used less covariates than Knoedler, 2024 [94]
Kalmar, 2024 [101]	ACS NSQIP data<br><br>See also Knoedler, 2024 [94] for both implant and autologous results using same database;	2015-2020	Autologous free flaps, immediate vs. delayed<br><br>Epidemiologic trends and flap failure<br><br>Used CPT codes for mastectomy and for free flap reconstruction	9788 pts: 4451 immediate, 5337 delayed<br><br>65% unilateral mastectomy and reconstruction	Delayed reconstruction has increased over time (46.9% in 2015, 58.6% in 2020)	Retrospective cohort<br><br>Multivariate regression	Rate of flap failure 1.3% vs. 1.3%, p=0.920; multivariate aOR=1.060, p=0.836<br><br>Flap failure, unilateral subset 1.2% vs. 1.1%, p=0.701; bilateral subset 1.4% vs. 1.7%, p=0.516<br><br>Flap failure decreased over time (2.7% in 2015, 1.2% in 2020)	Report indicates flap failure was removed from the NSQIP database in 2015 and data are limited to pts with ICD codes indicating reoperation<br><br>Database only has complications within 30 days; no information on staging, chemotherapy,

Citation	Study name and location	Years of study	Topic or comparison	Number of patients	Patient characteristics	Design	Results	Other
	Marquez, 2023 [100]		(not codes for cancer)					RT; no information on plane or type of flap
**RT Studies**								
Immediate vs. immediate-delayed vs. delayed								
Ulrikh, 2024 [102]	N.N. Petrov National Medical Research Center of Oncology, St. Petersburg, Russia	2016-2021	Simultaneous or delayed implant-based reconstruction	466 pts<br>158 pts immediate 1-stage then PMRT<br>210 pts immediate 2-stage with PMRT to expander<br>98 pts delayed, with PMRT before reconstruction	Breast cancer with mastectomy, adjuvant RT, reconstruction<br>Hypertension varied 10.1% vs. 17.6% vs. 23.5%, as did smoking history 13.3% vs. 18.1% vs. 22.4%	Binary logistic regression models to identify risk factors	Complications:<br>Clavien-Dindo grade I-II is mild, Grade III is severe: any grade 15.8% immediate vs. 22.9% 2-stage vs. 26.5% delayed; grades I-II (seroma or infection requiring oral antibiotics) 6.3% vs. 5.3% vs. 6.2%; grade III 9.5% vs. 17.6% vs. 20.4%<br>Capsular contracture grades III-IV (clinically significant): 15.2% vs. 2.9% vs. 2.0%<br>Reconstructive failure (explantation) 10.8% vs. 19.1% vs. 17.3%	Age, obesity, diabetes mellitus, hypertension, smoking history were not correlated with occurrence of complications<br>Single stage has less complications except higher rate of capsular contracture
PMRT to expander vs. PMRT to implant								
Yoon, 2020 [68]<br>2-yr follow-up	The Mastectomy Reconstruction Outcomes Consortium (MROC) Study, USA (9 institutions) and Canada (1 institution)<br>NCT01723423<br>1 site contributed 67.5% of pts	2012-2015	Complications and PROs of immediate 2-stage implant reconstruction; PMRT to implant vs. PMRT to expander<br>Complications at one and 2 years after reconstruction<br>PRO surveys before reconstruction	• 80 PMRT to implant (expander/implant exchange then PMRT; 72 with PRO data)<br>237 PMRT to expander; 262 with PRO data)	SSM (≈90%) and/or NSM for breast cancer or prophylaxis; first time 2-stage reconstruction with expander-implants, and receipt of PMRT to either expander or implant<br>Excluded previous failed breast reconstruction, augmentation or reduction; autologous reconstruction<br>Excluded reconstructive failure, or pts without	Prospective, multicentre cohort design; followed STROBE guidelines for cohort studies;<br>Mixed-effects logistic regression models for complications adjusted for baseline pt characteristics; models for PRO adjusted for patient characteristics and baseline (before reconstruction) PROs	• Overall complications, PMRT to implant vs. PMRT to expander: 41.3% vs. 40.1%, OR=1.25, 95% CI=0.69-2.25, p=0.459<br>• Major complications 32.5% vs. 34.6%, OR=1.18, 95% CI=0.62-2.22, p=0.615<br>• Reconstructive failure 10.0% vs. 19.8%, OR=0.72, 95% CI=0.28-1.83, p=0.488<br>Preoperative PROs (not adjusted) were similar<br>2-year PROs in 72 vs. 190 pts, in adjusted results PMRT to implant group had lower anxiety (ß= -4.8, p=0.01), depression (ß= -2.9, p=0.05), fatigue (ß= -4.4,	

Citation	Study name and location	Years of study	Topic or comparison	Number of patients	Patient characteristics	Design	Results	Other
	who had PMRT to the implant		and 1 and 2 years afterwards<br><br>BREAST-Q (Satisfaction with Breasts, Psychosocial Well-Being, Physical Well-Being, and Sexual Well-Being)<br><br>PROMIS-29 (physical function, anxiety, depression, fatigue, sleep disturbance, satisfaction with participation in social roles, and pain interference; for negative constructs such as anxiety, depression, fatigue, and sleep disturbance, higher scores correspond to worse outcomes)<br><br>EORTC QLQ-BR23 (body image and sexual functioning subscales)		complete 2-year follow-up from PROs<br><br>Similar pt characteristics except ADM use 32.5% vs. 59.1%; adjuvant chemotherapy 92.5% vs. 60.8%		p=0.03); other PROs had no significant differences<br><br>Minimal clinically important difference of PROMIS is 3-4.5, and therefore clinical significance of differences found is debatable	

Citation	Study name and location	Years of study	Topic or comparison	Number of patients	Patient characteristics	Design	Results	Other
RT studies, immediate + PMRT vs. PMRT + delayed								
Billig, 2017 [67]	The Mastectomy Reconstruction Outcomes Consortium (MROC) Study, USA (10 institutions and Canada (1 institution);<br><br>NCT01723423	2012-2015	Immediate then PMRT vs. PMRT then delayed autologous reconstruction<br><br>Complications reported 1 and 2 years after reconstruction<br><br>PRO prior to reconstruction (but after mastectomy in delayed group) and 1 and 2 years after reconstruction<br><br>Used BREAST-Q domains of Satisfaction with Breasts, Psychosocial Well-Being, Physical Well-Being (chest and upper body), Physical Well-Being (abdomen), and Sexual Well-Being	108 immediate and 67 delayed	Mastectomy with immediate or delayed abdominally based autologous breast reconstruction, PMRT<br><br>Exclude pts with RT before mastectomy, tissue expanders/ implants at time of reconstruction, both immediate and delayed reconstruction<br><br>Groups had different types of reconstruction: type free TRAM 0.9% vs. 29.9%, DIEP 70.4% vs. 58.2%, SIEA 22.2% vs. 7.5%; bilateral 34.3% vs. 13.4%	Prospective, multicentre cohort design; followed STROBE guidelines for cohort studies<br><br>Immediate group recruited and had PRO assessment before mastectomy + reconstruction; delayed group recruited after mastectomy and therefore assessed between mastectomy and reconstruction [62]<br><br>Mixed-effects logistic regression model for complications and for PROs<br><br>Model for complications included RT timing, plus clinical and demographic characteristics<br><br>Model for PROs included baseline (prior to reconstructive surgery) values of outcome variable, clinical and demographic characteristics<br><br>At least 1 year follow-up after reconstruction	Any breast complication at 1 year: 25.9% vs. 26.9%, p=0.540; logistic regression immediate vs. delayed OR=0.64, 95% CI=0.20-2.04, p=0.442 (at 2 years OR=1.14, 95% CI=0.31-4.17, p=0.848)<br><br>Any donor-site complication 39.8% vs. 16.4%, p=0.244; adjusted data not reported<br><br>Significant baseline differences; immediate had better PROs preoperatively than delayed group<br><br>Prior to reconstruction (before mastectomy in immediate group but after mastectomy for delayed group), the immediate group had better Satisfaction with Breasts (59.5 vs. 36.3, p<0.001), Psychosocial Well-Being (66.1 vs. 50.0, p<0.001), and Sexual Well-Being (52.1 vs. 29.8, p<0.001) and differences are likely due to presence/absence of breasts; Physical Well-Being (abdominal) was 87.3 vs. 82.9 (p=0.058)<br><br>After reconstruction, in the adjusted data there were no significant differences between immediate and delayed at 1 year after reconstruction; at 2 years the delayed group had better Physical Well-Being (chest and upper body) (70.5 vs. 80.6, p=0.048) but no other differences	<25 events for individual complications<br><br>For PROs, baseline refers to before reconstruction [62], and therefore before mastectomy only for the immediate group; for delayed group the baseline would have been after mastectomy (and presumably after PMRT)<br><br>PRO model adjusted for baseline prior to reconstruction; there was no baseline prior to mastectomy for delayed group and therefore baseline does not measure the same state; adjustment in model may be inappropriate in determining effect of timing of reconstruction<br><br>p values at year 1 and 2 adjusted for covariates and baseline PROs; table indicates Mean values adjust for covariates but not Preoperative values and therefore difficult to interpret (should adjust both pre and post measures for covariates; unclear if this is an error)<br><br>Due to study design, cannot make conclusion from PRO data if there is long-term difference in

Citation	Study name and location	Years of study	Topic or comparison	Number of patients	Patient characteristics	Design	Results	Other
								immediate vs. delayed; delayed group appears to have sharp decline after mastectomy (and PMRT) then recovers after reconstruction<br><br>Ideal study design (not in this report): should have delayed group with PROs prior to mastectomy, prior to PMRT, after PMRT but prior to reconstruction, then 1 and 2 years after reconstruction and there should be standardized (or at least reported) time between mastectomy, PMRT, and reconstruction<br><br>Study is only partially prospective, as delayed group was recruited subsequent to mastectomy and delay between mastectomy and reconstruction not reported
Staged (delayed-immediate; expander + RT then reconstruction) vs. immediate +RT								
Christopher, 2022 [16]	University of Pennsylvania Health System, Philadelphia, Pennsylvania	2005-2018	Reconstructive outcomes with staged autologous reconstruction (delayed immediate: subpectoral tissue expander at time of mastectomy, followed by	132 pts, 66 immediate and 66 staged, matched 1:1 by propensity score	Autologous reconstruction with abdominal flaps and PMRT<br><br>Staged group had subpectoral expander<br><br>Groups were similar except for chemotherapy (51.1% vs. 87.9% before reconstruction; 60.0% vs.	Retrospective, propensity-matched 1:1 by age, BMI, comorbidities (diabetes, hypertension, tobacco use); univariate analysis	In staged group, 28 pts (42.4%) had any tissue expander complication, of which 4 were prior to PMRT, 8 during PMRT, 16 after PMRT [presumably captures both mastectomy and PMRT complications]; there were 7 wound dehiscence, 19 infection, 8 expander exposure, 2 failure to expand<br><br>Complications after reconstruction (immediate vs. staged):	Complications were not prohibitive but vary according to group<br><br>Staged group had less complications due to reconstruction but need to also consider expander phase complications (mostly related to RT); it is possible that some revision in immediate group was due to RT as well, but this

Citation	Study name and location	Years of study	Topic or comparison	Number of patients	Patient characteristics	Design	Results	Other
			PMRT then reconstruction) vs. immediate reconstruction then PMRT		13.6% after reconstruction)<br><br>In immediate group, RT was median 3 months (ICR 2-5) after reconstruction; in staged group there was median 9 months (ICR 6-12) after RT before reconstruction (waited at least 3-6 months)		• Surgical site infection 4.55% vs. 1.52%, p=0.619<br>• Delayed healing 18.2% vs. 6.06%, p=0.059<br>• Hematoma 6.06% in both<br>• Seroma 9.09% vs. 1.52%, p=0.115<br>• Fat necrosis 24.2% vs. 9.09%, p=0.034<br>• Skin necrosis 18.2% vs. 0, p<0.001<br>• Flap loss due to vascular compromise 1.52% vs. 0, p=1.00<br>• Other 12.1% vs. 4.55%, p=0.206<br><br>Revision Surgery for asymmetry 42.4% vs. 12.1%, p<0.001	information was not recorded<br><br>No mention of type of mastectomy or stage of cancer, although 52.3% vs. 61.3% had nipple-areolar reconstruction (suggested they did not have NSM)<br><br>Timing between mastectomy and PMRT not reported for delayed group
Delayed immediate (staged) vs. delayed								
Hassan, 2023 [103]	The University of Texas MD Anderson Cancer Center	2016-2022	[Delayed-immediate (staged; skin preserving with expander to maintain skin and breast mound appearance) then autologous] vs. [delayed autologous]<br><br>PMRT (if used) was to expander in delayed-immediate; it was prior to reconstruction in delayed cases<br><br>Note: reconstruction is delayed in both	812 pts with 1002 reconstruction: 330 staged vs. 672 delayed<br><br>With PMRT: 129 staged vs. 435 delayed<br><br>Without PMRT: 201 vs. 237 (includes 37 vs. 10 preoperative RT)	Autologous flaps<br><br>Expander in subpectoral or prepectoral plane, with or without ADM<br><br>Excluded immediate autologous reconstruction, or implant-based reconstruction<br><br>RT in subset of pts: preoperative 3.0% vs. 5.5%; PMRT 39.1% vs. 64.7%<br><br>More NSM (9.5% vs. 5.7%) and SSM (71.9% vs. 54.2%) in delayed-immediate group<br><br>ALND 27.2% vs. 50.5%; SLNB 62.4% vs. 35.7%	Multivariable regression<br><br>Expanders deflated to 150 mL prior to PMRT and then reinflated ≈ 2-4 weeks after PMRT completed<br><br>72 breast surgeons, 33 plastic surgeons<br><br>PMRT pts: time to PMRT median 1 month in both groups; median 1 month from start to end of PMRT; median 8 months staged vs. 10 months delayed from end of PMRT to reconstruction<br><br>Pts without PMRT: median 8 months staged vs. 15 months delayed from	Primary outcome was development of flap-related complications, including infection, would dehiscence, skin flap necrosis, seroma, hematoma, arterial and venous thrombosis, and flap loss; publication does not indicate if these were measured after reconstruction, mastectomy, or both<br><br>Expander complications of exposure, capsular contractures, explantation were reported separately and authors state pts and surgeons accept a higher complication rate to obtain better final aesthetic results<br><br>Reported mixture of univariate analysis (no correction for confounders), or multivariate odds ratios or beta coefficients<br><br>Pts without PMRT, excluding expander-related complications (only those with >25 events):<br><br>• Any breast-related complication 21.4% vs. 26.2%, p=0.244 (univariate)	Delayed group without PMRT had longer time from mastectomy to flap reconstruction; may be due to more complex pathologic findings and more comorbidities<br><br>Without PMRT, staged group had shorter hospital stay, and lower rates of 30-day readmissions, seroma<br><br>In pts with PMRT, staged group had shorter hospital stay, lower 30-day readmission, less infection<br><br>Similar patient satisfaction after multivariate adjustment for staged and delayed groups in long term; no data on short-term; baseline data not recorded

Citation	Study name and location	Years of study	Topic or comparison	Number of patients	Patient characteristics	Design	Results	Other
			arms, the difference is the staged arm has temporary expander		NACT 36.7% vs. 60.1%; adjuvant chemotherapy 72.7% vs. 76.3%	mastectomy to reconstruction<br>PROs measured in October 2022 for all patients (not at specific time after surgery)	• Surgical complications, OR=0.65, 95% CI=0.40-1.07, p=0.094<br>• Seroma OR=0.42, 95% CI=0.19-0.94, p=0.036<br>• 30-day readmission 5.5% vs. 9.3%, p=0.132; OR=0.44, 95% CI=0.20-0.97, p=0.042<br>• Reoperation 6.5% s 8.4%, p=0.436; OR=0.81, p=0.528<br>• Hospital stay 4.0 vs. 4.2 days, p=0.178; ß -0.32, p=0.045<br><br>• No significant differences in Satisfaction with Breast (*β* 1.4, 95% CI= − 9.9 to 12.7, *p*=0.806), Psychosocial Well-Being (*β* −2.2, 95% CI= −11.7 to 7.2, *p*=0.640), or Sexual Well-Being (*β* 6.7, 95% CI= −6.4 to 19.9, *p*=0.312)<br><br>Patients with PMRT<br>• Any breast-related complication 22.5% vs. 25.1%, p=0.550 (univariate)<br>• Surgical complications OR=1.68, 95% CI=1.28-2.21, p=0.579<br>• Infection 3.9% vs. 10.3%, p=0.023; OR=0.33, 95% CI=0.13-0.87, p=0.023<br>• 30-day readmission 4.7% vs. 14.7%, p=0.002; OR=0.29, 95% CI=0.12-0.69, p=0.005<br>• Reoperation 5.4% vs. 10.6%, p=0.078; OR=0.50, 95% CI=-0.21-1.15, p=0.104<br>• Hospital stay 3.0 vs. 4.2 days, p=<0.001; ß -1.15, p<0.001<br><br>• No significant differences in Satisfaction with Breast (β − 3.7, 95% CI= −15.7 to 8.2, p=0.543), Psychosocial Well-Being (β − 7.3, 95% CI= −18.5 to 4.0, p=0.203), or Sexual	Factors used in multivariate model not reported and adjustment may have been insufficient to adjust for baseline differences; the discussion notes factors may confound the complication rates<br>Authors indicate they did not assess cosmetic outcomes

Citation	Study name and location	Years of study	Topic or comparison	Number of patients	Patient characteristics	Design	Results	Other
							Well-Being (ß 0.5, 95% CI= −12.9 to 13.9, p=0.942)<br><br>Tissue expander capsular contraction was 14.7% with PMRT and 3.5% without PMRT, univariate analysis p<0.001	

Abbreviations:
ACS-NSQIP, American College of Surgeons National Surgical Quality Improvement Program; ADM, acellular dermal matrix; aOR, adjusted odds ratio; ALND, axillary lymph node dissection; ASA status, American Society of Anesthesiologists physical status classification system; BCS, breast-conserving surgery; BMI, body mass index; CI, confidence interval; CPT, Current Procedural Terminology; DBCG, Danish Breast Cancer Group; BCT, breast-conserving therapy = breast-conserving surgery +RT); DCIS, ductal carcinoma in situ; DIEP, deep inferior epigastric artery perforator; DM, diabetes mellitus; DTI, direct to implant; DVT, deep venous thrombosis; ECOG, Eastern Cooperative Oncology Group; EORTC QLQ-BR23, European Organisation for Research and Treatment of Cancer Breast Cancer-Specific Quality of Life Questionnaire; HR, hazards ratio; HRQoL, health related quality of life; HTN, hypertension; ICD-9, International Classification of Diseases, Ninth Revision; IMA, internal mammary artery; ICR, Institute of Cancer Research; LABC, locally advanced breast cancer; LR, local recurrence; MPQ, McGill Pain Questionnaire-Short Form (MPQ-SF); MROC, Mastectomy Reconstruction Outcome Consortium; MS-TRAM, muscle-sparing transverse rectus abdominus muscle; MV, multivariable analysis; NPRS, Numerical Pain Rating Scale; NSM, nipple-sparing mastectomy; OR, odds ratio; PMRT, postmastectomy radiotherapy; PRO, patient-reported outcomes; PROMIS-29, Patient-Reported Outcomes Measurement Information System-29; PSW, propensity score weighting; pts, patients; QoL, quality of life; RCT, randomized control trial; RR, risk ratio; RT, radiotherapy; SF-36, 36-Item Short Form Survey; SIEA, superficial inferior epigastric artery; SSM, skin-sparing mastectomy; SLN, sentinel lymph node biopsy; STROBE, Strengthening the Reporting of Observational Studies in Epidemiology; TRAM, transverse rectus abdominis musculocutaneous flap; XRT, external beam radiation therapy

Back to Start of Tables

Table S5. Question 3a: NSM versus SSM

Citation	Study name and location	Years of study*	Topic or comparison	Number of patients	Design	Patient characteristics	Evaluation	Results
NSM vs. SSM								
Gerber, 2003 [110]<br>Gerber, 2009 long-term follow-up [111]	University of Rostock, Rostock, Germany	1994-2000	NSM vs. SSM (NSM planned) vs. MRM<br>Pts with MRM did not have reconstruction and are not reported in this table	134 planned NSM but follow-up data only for 114 pts<br>61 had NSM, 51 had NAC resected (i.e., SSM), 2 converted to MRM	Not stated but appears prospective<br>Follow-up at least 18 months; mean 59 months	Inclusion criteria: breast cancer, margins by sonography or mammography ≥2cm from nipple, no skin involvement; age <75 y, BMI 21-35 kg/m^2, no central tumour location, no contraindication for flap reconstruction<br>Patients meeting inclusion criteria were offered choice of NSM with reconstruction or MRM (no reconstruction); pts in NSM group with intraoperative concerns received SSM<br>2 pts with planned NSM had involved margins <5 mm) and were included in MRM group	Intraoperative frozen section of the base of the NAC: 51 pts had NAC removed due to >25% tumours cells in ducts, with tumour <2 cm from NAC and suspicious cells in base not clearly identified as tumour/benign by frozen section<br>SSM group is those pts converted from NSM due to frozen section results	Pt characteristics similar<br>Complications:<br>20% vs. 20%<br>After mean 101 months follow-up there were no significant differences: local recurrence 11.7% vs. 10.4%, isolated distant metastasis 23.3% vs. 25.0%, breast-cancer specific death 21.7% vs. 20.8%<br>Aesthetic results evaluated by surgeons: excellent results 73.8% vs. 78.4% at 59 months; 51.7% vs. 47.9% at 101 months; RT and large weight change were risk factors for decreased scores; patient ratings did not change as much
Jeon, 2010 [112]	Yeungnam University College of Medicine, Daegu, South Korea	1996-2006	NSM vs. SSM, according to frozen section analysis<br>Mastectomy by 1 surgeon, reconstruction by 2 surgeons	133 NSM; 69 SSM	Mean follow-up 67.6 months	Breast cancer, no clinically palpable lymph nodes, no infiltration or abnormalities in NAC visually or radiologically, Tis-2, N0-1; <4 positive nodes on ALND for invasive cancer; immediate reconstruction<br>Adjuvant chemotherapy if lymph node metastases, or >T2; hormones if hormone receptor positive; no PMRT	Frozen section analysis of retroareolar resection margin determined NSM or SSM; NAC sacrificed if positive on frozen section<br>SSM group is those pts converted from NSM to SSM due to cancer in	Local recurrence 9.0% vs. 5.8%, p=0.585<br>5-y local recurrence-free survival 92.1% vs. 95.2%, p=0.652

Citation	Study name and location	Years of study*	Topic or comparison	Number of patients	Design	Patient characteristics	Evaluation	Results
							frozen section <2 mm from cut surface	
Kim 2010 [113]	Asan Medical Center, Seoul, Korea	2001-2006	NSM vs. SSM with immediate pedicled TRAM reconstruction<br>Reconstruction by 1 plastic surgeon	152 NSM (187 attempted but 35 converted to SSM), 368 SSM<br>115 of NSM pts had prospective follow-up	Retrospective; prospective follow-up of complications in subset<br>Kaplan-Meier and multivariate (Cox proportional hazards) survival analysis<br>Median follow-up 60 vs. 67 months	Stage 0-IIIa, indications for SSM or NSM were any stage, any tumour size, any tumour-areolar distance; NSM offered if clinically normal nipple and no skin involvement<br>NAC preserved when palpation, shape, and colour of nipple were normal and NAC ducts tumour-free in frozen biopsies<br>Reconstruction using TRAM flaps<br>For NSM, radioisotope used instead of blue dye for SLNB as dye is risk factor for skin necrosis	Thin layer of glandular tissue was taken from under the areola for frozen sectioning<br>NSM attempted in 187 pts but 35 had positive results on subareolar frozen biopsy and converted to SSM	5-y DFS 89% vs. 87.2%, p=0.695;<br>5-y OS 97.1% vs. 95.8%, p=0.669; local failure 2% vs. 0.,8%, p=0.27<br>After adjustment for patient and tumour characteristics, DFS HR=1.004, 95% CI=0.52-1.93, p=0.991; OS HR=0.866, 95% CI=0.26-2.85, p=0.813<br>In 115 NSM, any NAC necrosis in 26 pts: 11 (9.6%) complete, 15 partial; NAC recurrence 1.3%
Wang, 2020 [114]	Yale University School of Medicine,	2010-2017	Complication, reoperation, and length of stay for NSM vs. SSM<br>Single centre, 22 surgeons (although 5 surgeons conducted about 90% of operations)	217 NSM, 581 SSM	Retrospective<br>Bivariate analysis then multivariate analyses using clinicopathologi c variables that were significant (p≤0.05)	All pts with NSM or SSM; indications for NSM instead of SSM were at discretion of surgeon; individual surgeon was a predictor of receiving NSM<br>NSM pts were younger, more private insurance, lower BMI, smaller breasts, more prophylactic, lower stage disease, less diabetes, more implant-based reconstruction<br>Prophylactic 20.7% vs. 5.9%	None reported	Complication rates 27.6% vs. 21.9%, p=0.091; after multivariate analysis of therapeutic cases: NSM had higher complication rate, OR=1.822, 95% CI=1.163-2.853, p=0.009<br>Increased complication rate appears due to inclusion of nipple necrosis/ischemia (16.5% vs. 0%) as NSM was lower for most other complications<br>36 pts (16.5%) nipple ischemia, eschar, or necrosis; only 2 pts required nipple removal<br>Reoperation within 90 days: 8.3% vs. 12.6%, p=0.104; multivariate analysis of therapeutic cases OR=0.876, 95% CI=0.453-1.696, p=0.695

Citation	Study name and location	Years of study*	Topic or comparison	Number of patients	Design	Patient characteristics	Evaluation	Results
								Length of stay ≥2 days: 69.1% vs. 79.7%, p=0.002; after multivariate analysis of therapeutic cases there was no difference, OR=0.701, 95% CI=0.442-1.111, p=0.130<br><br>High volume surgeons had lower rates of complications and reoperation
Kelly, 2022 [115]	Massachusetts General Hospital (MGH) or Mass General Brigham Salem Hospital	2009-2019	Patient Satisfaction with Breasts after NSM vs. SSM; BREAST-Q ≥1 y after immediate reconstruction<br><br>Comparisons for groups with NSM/SSM 1-5 years ago and those with NSM/SSM 6-10 years ago	247 NSM and 184 SSM completed postoperative survey	Retrospective, from prospectively maintained NSM and SSM databases<br><br>Linear regression; multivariate logistic regression to identify risk factors for dissatisfaction	NSM or SSM and immediate reconstruction without reconstruction failure or operation within 2 months of survey<br><br>SSM was recommended instead of NSM if clinical or imaging evidenced of NAC involvement, LABC with skin involvement, inflammatory, significant size reduction, expected poor nipple position<br><br>NSM group had lower BMI, smaller breasts, were younger, more likely premenopausal, less hypertension and smoking, more genetic risk factors and prophylactic surgery<br><br>26.1% of SSM had nipple reconstruction	Surgical or pathological evaluation not mentioned<br><br>Used six BREAST-Q modules: Psychosocial Well-Being, Sexual Well-Being, Satisfaction with Breasts, Satisfaction with Implants, Physical Well-Being: Chest, and Effects of Radiation<br><br>Baseline values (before mastectomy) were not measured	NSM required less aesthetic revision operations (13.8% vs. 32.6%, p<0.001) (data not adjusted)<br><br>37.8% response rate (69.7% for paper and 13.0% for electronic surveys)<br><br>Dissatisfaction with breasts: multivariate analysis OR=0.95, p=0.812<br><br>Other outcomes were not adjusted using multivariate analysis; in the shorter follow-up group (1-5 years) NSM better than SSM for Psychosocial Well-Being and Sexual Well-Being modules and some components of Satisfaction with Breasts; differences were smaller and not statistically significant in the 6-10 years group<br><br>1.2% (3 pts) NSM group had nipple excision for positive margins
Racz, 2022 [116]	Mayo Clinic Rochester, MN	2011-2015	PROs with or without reconstruction and NSM vs. SSM using BREAST-Q for mastectomy with reconstruction	198 NSM, 336 SSM	Retrospective review of prospective database<br><br>BREAST-Q mailed to all pts with mastectomy in database	Identified pts by ICD-9 codes in database<br><br>NSM or SSM for breast cancer or significantly elevated risk of breast cancer, and reconstruction with expander/implant and autologous reconstruction	Reason for NSM vs. SSM not reported<br><br>No information on operation or pathology	NSM vs. SSM<br>• Satisfaction with Breasts mean 71.8 vs. 70.2, p=0.21, multivariable p=0.984<br>• Psychosocial Well-Being mean 81.9 vs. 81.3, p=0.47, multivariable p=0.929<br>• Sexual Well-Being mean 64.5 vs. 58.0, p=0.002, multivariable p=0.033 (effect size 4.69 points)

Citation	Study name and location	Years of study*	Topic or comparison	Number of patients	Design	Patient characteristics	Evaluation	Results
					Linear regression for multivariable analysis adjusting for potential confounders<br><br>PROs using BREAST-Q	NSM pts were younger, lower BMI, more prophylactic; less neoadjuvant chemotherapy, and axillary surgery, or PMRT<br><br>93% of reconstruction used implants<br><br>In SSM, 64% had no nipple reconstruction or tattoo, 12% had reconstruction, 8% tattoo, 17% reconstruction and tattoo		NSM vs. SSM + nipple reconstruction: Sexual Well-Being scores similar, multivariable p=0.182; NSM vs. SSM without tattoo or reconstruction p=0.008, 6.34 points in favour of NSM<br><br>Individual components of satisfaction score: NSM more favorable for natural appearance, appearance unclothed<br><br>Individual components of Sexual Well-Being score: NSM better sexual confidence unclothed, sexual attractiveness when unclothed
Cho, 2023 [118]	Severance dataset from Severance Hospital, Yonsei University College of Medicine, Seoul, Republic of Korea<br><br>Validated results by using the SEER database	2010-2017<br><br>SEER 2000-2018	Is NSM with immediate reconstruction inferior to total or SSM for 5-y DFS and OS?	Severance 611 pts: 151 NSM, 460 total/SSM<br><br>SEER 14,770 NSM; 485,245 total mastectomy	Propensity-score matching; survival by Kaplan-Meier and Cox proportional hazard regression<br><br>Severance: median follow-up 53 months NSM and 71 months other	Severance: pts with mastectomy; excluded LCIS, DCIS, bilateral, metastasis, or without immediate reconstruction<br><br>Pts with suspected NAC invasion were not eligible for NSM<br><br>SEER: breast cancer pts; excluded bilateral non-cancer deaths, SEER does not include DFS data<br><br>SEER data: NSM younger (51.3 vs. 59.5), different in most surgical groups, NSM younger, lower TNM stage, grade	Severance: frozen-section biopsy of retroareolar tissue in NSM, nipple removed if retroareolar margin was positive and excluded from NSM group	Severance: DFS; secondary endpoints LRFS, DMFS, OS<br><br>• LR 0 vs. 9 (2.0%)<br>• Regional recurrence 6 (4.0%) vs. 8 (1.7%)<br>• DM 10 (6.6%) vs. 27 (5.9%)<br>• Death: 0 vs. 4 (0.9%)<br>• 5-y LRFS 95.8% vs. 96.8%, HR=1.22, p=0.68<br>• 5-y DMFS 93% vs. 93.7%, HR=1.25, p=0.55<br>• 5-y DFS 89.1% vs. 90.7%, HR=1.15, 95% CI=0.65-2.03, p=0.64<br>• 5-y OS 97.3% vs. 97.4%, HR=1.05, 95% CI=0.34-3.20, p=0.93<br><br>With propensity score matching (NSM matched 1:2 with total/SSM):<br><br>• 5-y DFS 89.7% vs. 89.4%, HR=1.04, 95% CI=0.54-2.01, p=0.91<br>• OS 97% vs. 97.3%, HR=1.07, 95% CI=0.30-3.87, p=0.91<br><br>SEER database:<br><br>OS HR=0.60, 95% CI=0.54-0.67, p<0.001

Citation	Study name and location	Years of study*	Topic or comparison	Number of patients	Design	Patient characteristics	Evaluation	Results
Ogiya, 2023 [117]	12 hospitals, Japan	2008-2016	LR after immediate breast reconstruction	4153 cases:<br>1192 NSM (359 non-invasive, 831 invasive)<br>1409 SSM (445 non-invasive, 960 invasive)<br>1552 total mastectomy	STROBE reporting guidelines<br>Invasive and non-invasive analyzed separately<br>Stepwise regression, Cox proportional hazard model for LR	BCS or mastectomy depending on expected margin and breast shape, and pt preference; mastectomy if BRCA positive<br>NSM if nipple could be preserved based on imaging and palpation and pt wished to keep the nipple<br>Excluded NACT<br>Tissue expanders in 88.1% of pts<br>Median follow-up 75 months		Of 73 NSM with LR, 10 (13.7%) involved nipple<br>For 7y LR, due to missing variables, only data for 53% non-invasive and 91% invasive pts used for analysis:<br>Non-invasive cancer; univariate results for 187 NSM and 267 SSM: 7-y LR 2.6% vs. 3.8%<br>Invasive cancer, results for 776 NSM and 894 SSM: 7-y LR 6.6% vs. 4.8% (HR compared with total mastectomy 3.112 and 1.872, univariate; HR 2.738 and 1.723 multivariate
Sasada, 2024 [119]	Collaborative Study Group of Scientific Research of the Japanese Breast Cancer Society<br>12 institutions	2008-2016	RT for involved surgical margins after immediate reconstruction	1431 NSM, 1593 SSM<br>123 NSM and 102 SSM with involved margins	Retrospective<br>Median follow-up 75 months<br>HRs adjusted for previously reported risk factors	Breast cancer, mastectomy, immediate reconstruction<br>NSS, SSM, or total mastectomy depending on institutional standards and pt preference<br>PMRT if lymph node involvement, large tumours, involved margins<br>Involved margins are invasive or in situ tumour on ink	Not reported	Involved surgical margins: in 123 cases (8.6%) of NSM; 102 cases (6.4%) of SSM<br>Involved nipple margins in 26 cases (1.8%) of NSM<br>LR in pts with involved surgical margins, HR compared with total mastectomy<br>NSM: HR=1.26, 95% CI=0.37-4.03, p=0.696<br>LR, SSM: HR=0.65, 95% CI=0.18-2.36, p=0.515

*Usually year of diagnosis or initial surgery

Abbreviations:
ADM, acellular dermal matrix; ALND, axillary lymph node dissection; ASM, areolar-sparing mastectomy; BCS, breast conserving surgery; BCSS, breast cancer-specific survival; BMI, body mass index; BRCA, BReast CAncer gene; CI, confidence interval; DCIS, ductal carcinoma in situ; DFS, disease-free survival; DMFS, distant metastasis-free survival; DTI, direct to implant; ELIOT, intraoperative electron-beam radiotherapy; HR, hazard ratio; ICD-9, International Classification of Diseases, Ninth Revision; ILC, invasive lobular carcinoma; LABC, locally advanced breast cancer; LCIS, lobular carcinoma in situ; LR, local recurrence; LRR, locoregional recurrence; MGH, Massachusetts General Hospital; MRM, modified radical mastectomy; MRI, magnetic resonance imaging; NAC, nipple-areolar complex; NACT, neoadjuvant chemotherapy; NCDB, National Cancer Database, American College of Surgeons, with data from >1500 Commission on Cancer-accredited facilities in the United States and ≈70% of newly diagnosed cancers in the United States; NSM, nipple-sparing mastectomy; OR, odds ratio; OS, overall survival; PMRT, postmastectomy radiotherapy; PRO, patient-reported outcomes; pts; patients; RT, radiotherapy; SEER, Surveillance, Epidemiology, and End Results database, SLNB, sentinel lymph node biopsy; SSM, skin-sparing mastectomy; TND, tumour-nipple distance; TNM, tumour, node, metastasis staging system; TRAM, transverse rectus abdominis musculocutaneous flap; TSSM, total skin-sparing mastectomy; US, ultrasound

Back to Start of Tables

Table S6. Question 3b: Oncologic and nipple outcomes according to criteria for NSM patient selection & Question 3c: Surgical factors influencing nipple outcomes

Citation	Study name and location	Years of study*	Topic or comparison	Number of patients	Design	Population	Criteria for NSM	Pathology	Oncologic outcomes	Surgery Details	Nipple viability and necrosis
Tumour-Nipple Distance											
Ryu, 2016 [120]	Samsung Medical Center, Sungkyunkwan University, Seoul, Korea	2008-2014	NSM with TND <2 cm, negative frozen biopsy at nipple base	251 pts, 266 NSM: TND <2 cm in 145, TND ≥2 cm in 121	Retrospective review<br><br>2 groups by TND similar except TDN<2 cm group had lower tumour size, stage, chemotherapy use and more ER/PR+ and hormonal therapy<br><br>Mean follow-up 28.4 vs. 22.2 months	All pts who underwent NSM + IBR for breast cancer<br><br>Exclude collagen-vascular disease, prophylactic (TND not measurable)	Preoperative MRI to define TND measured as shortest distance between tumour border and base of nipple; in case of NACT used MRI after NACT completed<br><br>Inclusion: skin island (removed skin) <10 cm at largest dimension,<br><br>Exclusion: clinical evidence of NAC involvement, inflammatory cancer, LABC with skin involvement, bloody nipple discharge	Intraoperative frozen biopsy at nipple base; NAC or nipple removed if positive for invasive ductal or lobular carcinoma, lymphovascular invasion, ductal carcinoma in situ, atypical ductal hyperplasia with necrosis, or lobular neoplasia/lobular carcinoma in situ<br><br>371 attempted NSM but 78 excluded due to positive results under NAC at frozen biopsy (n=76) or permanent biopsy (n=2) and 27 excluded because TND not clearly defined	Very low event rates; no difference between TND < 2 cm or ≥2 cm: LRR 3 (2.1%) vs. 3 (2.5%), metachronous contralateral breast cancer 2 (1.3%) vs. 1 (0.8%), DM 1(0.7%) vs. 1 (0.8%) or death (none in either group)<br><br>DFS p=0.894, LRFS reported as p=0.594 in figure and p-0.509 in text	Incision placement at discretion of surgeons: 27.4% radial incision, 30.1% lateral, 32.0% periareolar<br><br>Subcutaneous dissection removed maximum amount of glandular tissue and raised NAC as full-thickness skin flap by electrocautery<br><br>Ducts just below NAC sharply excised and sent for intraoperative frozen biopsy and permanent biopsy	Did not report by TND<br><br>Reported by time periods 2008-2010 (n=43) and 2011-2014 (n=223): Total nipple necrosis 0 and 1 (0.4%); partial nipple necrosis 5 (11.6%) and 3 (1.3%); partial areola necrosis 2 (4.7%) and 3 (1.3%)<br><br>One NAC excision because of total nipple necrosis
Alsharif, 2019 [121]	Samsung Medical Center, Seoul, Korea	2008-2014	Long-term outcomes by TND <2 cm or ≥2 cm	245 pts; 128 <2 cm TND and 117 ≥2 cm TND	Mean follow-up 60.5 months<br><br>Note that groups were not equivalent	<2 cm TND group had smaller tumours (median 0.7 cm vs. 3.0 cm, p=0.005), more DCIS (32.0% vs.	Pts evaluated for signs of skin, NAC, or nipple involvement as indicated by retracted nipple	Intraoperative frozen biopsy to confirm negative margin at nipple base and also analyzed by permanent sections	LRR: 4 pts (3.1%) in <2 cm group vs. 6 pts (5.1%) including 1 (0.8%) vs. 3 (2.6%) in nipple	7.0 cm long radial incision, nipple tissue just behind the dermis was sharply dissected at the nipple-dermal junction and	

Citation	Study name and location	Years of study*	Topic or comparison	Number of patients	Design	Population	Criteria for NSM	Pathology	Oncologic outcomes	Surgery Details	Nipple viability and necrosis
					and event numbers are low	16.2%), less invasive ductal carcinoma (57.8% vs. 72.6%), and received less chemotherapy<br>IBR after mastectomy in all pts	Preoperative breast MRI to measure TND between tumour and base of the nipple; in pts with NACT, measurement was done after NACT and pts with pathological complete response were excluded	If frozen sections positive for invasive cancer, DCIS, atypical ductal hyperplasia with necrosis, or lobular neoplasia the NAC was removed and pts excluded from the study	DM 2.3% vs. 5.1%<br>3 deaths (1.2%): 1.6% vs. 0.9%<br>No significant difference in DFS or LRFS	dissected; glandular tissue removed by subcutaneous dissection using electrocautery<br>Follow-up every 6 months for 5 years and then annually	
Balci, 2019 [122]	Istanbul, Turkey	2007-2017	Oncologic safety with TND <2 cm	193 pts; 59 with TND <2 cm, 134 with TND ≥2 cm	Retrospective<br>Median follow-up 62 months<br>Groups similar in tumour size and nodal involvement	NSM with immediate reconstruction, invasive cancer<br>Excluded pure DCIS, neoadjuvant chemotherapy<br>Immediate implant or tissue expander reconstruction, with ADM if needed	Exclude if clinical evidence of NAC involvement (nipple retraction, Paget changes, and pathologic nipple discharge), inflammatory breast cancer, no MRI<br>MRI, mammography, and ultrasound used to determine TND and tumour size<br>TND defined as distance from base of the nipple to the closest edge of the tumour	Nipple base (biopsy of nipple/subareolar margin) negative for invasive or in situ cancer by frozen-section or paraffin histopathology; NAC removed if positive for carcinoma; LCIS or atypical hyperplasia was not counted as involvement	4 LR (6.7%) with TND <2 cm vs. 5 LR (3.73%) + 3 DM with TND ≥2 cm<br>Recurrence with invasion of NAC: 1 (1.69%) in TND <2 cm group; 2 (1.49%) in TND ≥2 cm<br>10-y DFS 93.2% vs. 96.3%, p=0.368	lateral incisions ≥2 cm from the nipple were preferred; IMF incisions less often; periareolar and skin-reducing incisions in sporadic cases with very large ptotic breasts<br>Hydro-dissection used for NAC and skin isolation	Wound dehiscence: 2 (3.38%) vs. 3 (2.23%)<br>Total nipple necrosis: 3 (5.08%) vs. 1 (0.74%)<br>Partial flap necrosis: 2 (3.38%) vs. 3 (2.23%)

Citation	Study name and location	Years of study*	Topic or comparison	Number of patients	Design	Population	Criteria for NSM	Pathology	Oncologic outcomes	Surgery Details	Nipple viability and necrosis
Fregatti, 2020 [123]	Ospedale Policlinico San Martino, Genoa, Italy	2012-2018	Effect of TND by MRI vs. permanent section analysis	246 pts	Retrospective<br><br>Median follow-up 31-33 months	Therapeutic NSM<br><br>Exclude large breast size or ptosis that would result in unacceptable nipple position<br><br>2-stage expander-implant in 214 pts, immediate implant in 32 pts<br><br>Pts with TND ≤2 cm compared with 2-5 cm had a significantly higher rate of invasive ductal carcinoma (72% vs. 51%, p<0.003), less lobular cancer (13% vs. 26%), and more excision margins less than 2 mm (42% vs. 5%,p<0.001)	No clinical signs of direct NAC involvement such as bloody nipple discharge, nipple retraction, Paget disease, and no NAC involvement by imaging<br><br>Excluded inflammatory, LABC with skin involvement or NACT<br><br>All pts had MRI to measure TND	Duct bundle by definitive pathology<br><br>Retroareolar margin excised and marked with a stitch<br><br>If definitive pathological examination of the retroareolar margin or nipple duct bundle detected invasive cancer, DCIS, or atypia the nipple was excised but often retaining areola (ASM); these pts were excluded from NSM group<br><br>11 retroareolar specimens positive at definitive pathology (6 DCIS, 4 close/positive margins, 1 atypia);<br><br>Nipple excised in 7 pts and NAC in 4 pts; final re-excision specimen examination showed residual disease in 7 pts (63.6%).	In NSM:<br><br>LRR 4 (3.4%) with TND ≤2 cm, 2 (2.6%) with TND between 2 and 5 cm, 1 (2.7%) with TND >5 cm<br><br>No NAC recurrence<br><br>LR 2.4% vs. 1.3% vs. 2.7%<br><br>DFS >96%<br><br>In pts with ASM or SSM: 4/11 (36%) had LR far from NAC excision	Incision at surgeon discretion; S italic incision was most frequent except for patients having skin-reducing NSM in which inframammary or inverted T incision was used<br><br>Skin flaps raised in Cooper's ligament plane up to the edge of the areola by electrocautery, tumescence, or sharp dissection; at areola edge the dissection was on the deep side of the areola dermis stopping beneath the nipple<br><br>Nipple duct bundle isolated and grasped with a curved clamp, most duct tissue removed while leaving intact the vessels travelling into the nipple skin, duct bundle divided above and below clamp, and contents of clamp sent for definitive pathology<br><br>Flap thickness of 3-5 mm carefully checked to avoid residual breast tissue on skin flaps	Nipple epidermolysis in 23/235 pts (9.8%) with NSM<br><br>Full-thickness nipple necrosis and loss in 3 pts (1.3%)

Citation	Study name and location	Years of study*	Topic or comparison	Number of patients	Design	Population	Criteria for NSM	Pathology	Oncologic outcomes	Surgery Details	Nipple viability and necrosis
										SLNB for invasive and DCIS	
Wu, 2021c [6]	Asan Medical Center, Seoul, Korea	2003-2015	Oncologic safety of NSM with TND ≤1 cm vs. >1 cm	1369 pts: after matching was 495 pts TND ≤1 cm and 495 pts TND >1 cm	Retrospective<br><br>Propensity-score matching to reduce effect of confounders on oncologic outcomes between TND groups<br><br>Median follow-up (matched groups) 109 and 112 months	Primary breast cancer with immediate reconstruction<br><br>NSM indications: any stage, size<br><br>Exclusions: pT4 disease, synchronous distant metastasis, incomplete data	Any TND allowed, calculated from tumour to nipple using MRI, ultrasound, or mammography; if multifocal or multicentric then used closest lesion to the nipple for calculations<br><br>Inclusion: clinically normal NAC and no skin involvement or inflammatory disease	Retroareolar frozen-section biopsy and permanent biopsy in all pts; nipple ± areola immediately removed if positive for malignancy and converted to SSM or ASM and excluded from NSM cohort	LR 54 vs. 42 cases (10.9% vs. 8.5%) LR in NAC 29 vs. 19 (5.9% vs. 3.8%) cases<br><br>Regional recurrence 16 vs. 23 pts (3.2% vs. 4.6%)<br><br>DM 33 vs. 29 cases (6.7% vs. 5.9%)<br><br>Death 5.5% vs. 4.8%<br><br>5-y cumulative LR 8.1% vs. 6.3%, p=0.268; NAC recurrence 5.1% vs. 2.8%, p=0.072; regional recurrence 2.0% vs. 3.6%, p=0.125; DM 5.9% vs. 4.8%, p=0.480<br><br>10-y LRFS 87.1% vs. 90.7%, p=0.164, HR=1.331, 95% CI=0.889-1.992<br><br>10-y DFS 77.9% vs. 81.6%, p=0.222, HR=1.191, 95%	Details not reported<br><br><u>Other</u><br><br>Authors concluded that TND ≤1 cm is not contraindication to NSM	Not reported

Citation	Study name and location	Years of study*	Topic or comparison	Number of patients	Design	Population	Criteria for NSM	Pathology	Oncologic outcomes	Surgery Details	Nipple viability and necrosis
									CI=0.899-1.577, p=0.222		
Kim, 2020 [124]	Pusan National University Hospital, Busan, Korea	2007-2015	Experience with NSM with immediate reconstruction<br><br>NSM outcomes<br><br>No comparison of selection criteria or surgical procedures	251 pts, 251 NSM<br><br>119 TND ≤2 cm; 132 TND >2 cm,	Retrospective<br><br>Mean follow-up 68 months	Excluded NACT<br><br>79% had adjuvant RT<br><br><25 events for multiple linear regression	Pts with breast cancer and no tumour invasion to NAC by bilateral mammography, ultrasound, MRI, and physical exam<br><br>Mean TND 2.5±1.7 cm; 119 (47.4%) ≤2 cm and 69 (27.5%) ≤1 cm	Frozen section biopsy of retroareolar tissue; central nipple duct margin evaluated by intraoperative and permanent pathology; NAC removed (converted to SSM or other procedure and excluded from study) if cancer or atypical cells found in frozen section or permanent section<br><br>15 pts (6.0%) positive surgical margins but no additional surgery as all breast tissue was removed; they received RT	11 (4.4%) LRR: 1 local recurrence in NAC,10 in chest wall or skin<br><br>Systemic recurrence in 16 pts (6.4%), consisting of 11 pts (4.4%) only systemic and 5 (2.0%) also with LRR<br><br>5 (2.0%) breast cancer related deaths<br><br>5-y OS 98.0% for invasive cancer and 100% in situ<br><br>4.4% LRR: 5.0% with TND ≤2 cm; 3.8% with TND >2 cm<br><br>6.4% DM: 5,0% with TND ≤2 cm; 7.6% with TND >2 cm	Mayo scissors for sharp dissection; electrocautery only used for bleeding control to minimize thermal injury to the skin envelope<br><br>Removed entire breast tissue at level of subcutaneous fat layer	4 (1.6%) partial nipple necrosis; no additional surgery needed
Non-comparative studies											
Beller, 1981 [125]	Münster University Women's Hospital,	1974-1980	No comparison; NSM + optional	135 NSM: 110 T1 and 25 T2	Prospective, non-RCT, no comparison	Initially stage T1N0, later allowed T+N+, (110 pts T1 and	≥3 cm from areola at start of study (later allowed closer to	Frozen section of areola base and wedge excision of nipple; NAC	T1: one death (0.9%)	Subcutaneous mastectomy including complete removal of visible	Necrosis of NAC: 0 if skin reduction not needed, 3% if

Citation	Study name and location	Years of study*	Topic or comparison	Number of patients	Design	Population	Criteria for NSM	Pathology	Oncologic outcomes	Surgery Details	Nipple viability and necrosis
	Münster, Germany		Delayed reconstruction (6-12 months; after RT)		Follow-up depended on time of enrolment (>72 months for first pts and 0-6 months for last pts)	25 pts >T1), ≤55 y old; chemotherapy (if node positive), lymphadenectomy, RT<br>Delayed reconstruction after skin healing and RT, generally 6-12 months later	areola if the areola was histologically tumour free)	removed if carcinoma found in frozen or permanent sections	T2+: 3 deaths (12%)<br>No recurrence in areola<br>Recurrence or metastasis: 6 pts T1 (5.5%), 6 pts T2+ (24%)<br>3% CBC: 2.2% T1 and 4.0 % T2	glandular tissue (≈95%) with a lateral IMF incision and excision of nipple and areola base, sharp dissection of glandular and fatty tissue from the skin, resection of fascia of pectoralis major muscle.<br>In large breasts, skin was reduced 5 cm below the lower edge of the areola	skin reduction needed (25 pts) due to large breasts (impairment of vascular supply)
Psaila, 2006 [126]	Universita Cattolica "Sacro Cuore" - Rome - Italy	2002-2004	NSM, no comparison	139 NSM	Retrospective, mean 18 months follow-up (12-36 months)	All pts with NSM and immediate reconstruction (127 implant and 12 DIEP flap); 103 T1, 26 T2; 10 pts had recurrence after QuaRT<br>Exclude age >70 y, LN+	NSM decided on after preoperative mammography, ultrasound, pathology<br>Exclusion criteria: nipple retraction, tumour >3.5 cm, centrally located, TND <2 cm, Paget disease, ematic nipple secretion with cytological evidence for neoplastic cells	Touch prep cytology biopsy and intraoperative touch prep cytology of undersurface of areolar flap<br>1 pt with NAC involvement converted to SSM	Recurrence rates similar to radical mastectomy but data not reported	Skin incisions near the tumour and a skin ellipse up to a distance of 0.5-1 cm to the areolar lateral border removed.<br>Subcutaneous dissection by scissors; dissection along the superficial fascia	1% NAC necrosis, 18% NAC depigmentation, 75% loss of NAC sensitivity
Benediktsson, 2008 [127]	Huddinge University Hospital, Stockholm, Sweden	1988-1994	Survival with NSM + immediate reconstruction	216	Prospective, follow-up every 3 months for 5 y then at least	Primary unilateral breast cancer, not suitable for partial mastectomy	See population	Biopsy from adjacent to the remaining gland tissue sent for frozen section; NAC removed (11	LRR 52 pts; 5-y 16.2%, 10-y 20.8%<br>DM 44 pts (20.4%)	A 5-mm thick plate of gland tissue with a 2 cm diameter was left beneath the nipple to preserve the blood supply of the NAC	Not reported

Citation	Study name and location	Years of study*	Topic or comparison	Number of patients	Design	Population	Criteria for NSM	Pathology	Oncologic outcomes	Surgery Details	Nipple viability and necrosis
					annually; median 13 y, minimum 11.6 y (or until death)<br>75% of operations by 2 co-authors, 6 others breast surgeons for rest	because of large (>3 cm) or multifocal carcinoma (74%); or reoperation after partial mastectomy <3 months earlier and multifocality or residual tumour highly suspected (47.7%))<br>40.3% LN+, 47 pts with RT<br>27 silicone submuscular implants, 189 subcutaneous saline implants<br>Authors indicate more pts would receive RT by policy in effect by time of publication		pts) if malignant cells found at frozen section (1 of these negative at final pathology)<br>3 additional positive at final pathology	DFS 51.3%; 5-y 68.0%, 10 y 60.0%<br>OS 76.4%; 5-y 83.5%, 10=y 80.5%<br>34 LRR were in same quadrant as primary tumour; authors state this may suggest inadequate surgery<br>LRR with RT: 4 pts (8.5%); LRR without RT: 48 pts (28.4%)<br>Note median 13 y follow-up, lack of RT, larger size and multifocality may account for higher rates of recurrence		
Jeon, 2010 [112]	Yeungnam University College of Medicine, Daegu, South Korea	1996-2006	NSM vs. SSM, according to frozen section analysis<br>No comparison of selection criteria or surgical procedures	133 NSM; 69 SSM	Mean follow-up 67.6 months (71.4 months NSM, 60.2 months SSM)<br>No multivariate analysis	Breast cancer<br>Adjuvant chemotherapy if lymph node metastases, or >T2; hormones if hormone receptor	NSM if no clinically palpable lymph nodes, no infiltration or abnormalities in NAC visually or radiologically, Tis-2, N0-1; <4 positive nodes on	Frozen section analysis of retroareolar resection margin determined NSM or SSM; NAC sacrificed if positive on frozen section	Local recurrence 12 pts (9.0%; 8 pts in NAC and 4 skin) vs. 4 pts (5.8%, all skin), p=0.585<br>5-y local recurrence-free survival 92.1%	Generally used hemispherical incision around areola and transverse incision extending ≈ 4 cm to outside and inside; if previous BCS then used previous radial incision as possible	Also in Q3a<br>For NSM: NAC necrosis in 3 cases (2.3%): 2 partial (1.5%) and 1 complete necrosis (0.8%, NAC removed

Citation	Study name and location	Years of study*	Topic or comparison	Number of patients	Design	Population	Criteria for NSM	Pathology	Oncologic outcomes	Surgery Details	Nipple viability and necrosis
			Mastectomy by 1 surgeon, reconstruction by 2 surgeons			positive; no PMRT<br>More chemotherapy in NSM group (60.1% vs. 44.9%)<br>Pts with involvement of NAC were more likely to have DCIS, central or diffuse tumours	ALND for invasive cancer; immediate reconstruction	52 patients had tumour invasion of the cut surface; 50 found in both frozen and 2 only on permanent sections<br>SSM group is those pts converted from NSM to SSM due to cancer in frozen section <2 mm from cut surface	vs. 95.2%, p=0.652	Removed subcutaneous fat and breast tissue leaving about 1 cm fat; preserved blood vessels flowing into areola by careful dissection<br>Cut surface of breast parenchyma below areola painted with blue dye and sectioned for frozen section examination; if cancer on cut surface or within 2 mm the NAC complex was resected for ≈0.5-1.0 cm	and converted to SSM)
Kim 2010 [113]	Asan Medical Center, Seoul, Korea	2001-2006	NSM vs. SSM with immediate pedicled TRAM reconstruction<br>No comparison of selection criteria or surgical procedures	152 NSM, 368 SSM<br>115 of NSM pts had prospective follow-up	Retrospective; prospective follow-up of complications in subset<br>Kaplan-Meier and multivariate (Cox proportional hazards) survival analysis<br>Median follow-up 60 vs. 67 months	Stage 0-IIIa, indications for SSM or NSM were any stage, any tumour size, any tumour-areolar distance<br>Reconstruction using TRAM flaps<br>For NSM, radioisotope used instead of blue dye for SLNB as dye is risk factor for skin necrosis	NSM offered if clinically normal nipple and no skin involvement NSM attempted in 187 pts but 35 had positive results on subareolar frozen biopsy and had SSM<br>NAC preserved when palpation, shape, and colour of nipple were normal and NAC ducts tumour-free in frozen biopsies	Thin layer of glandular tissue under areola taken for frozen section analysis	5-y DFS 89% vs. 87.2%, p=0.695; 5-y OS 97.1% vs. 95.8%, p=0.669; local failure 2% vs. 0.,8%, p=0.27<br>After adjustment for patient and tumour characteristics, DFS HR=1.004, 95% CI=0.52-1.93, p=0.991; OS HR=0.866, 95% CI=0.26-2.85, p=0.813	Lateral incision for SSM; either periareolar incision with lateral extension or lateral incision alone for NSM; periareolar used early in series but high skin necrosis so not used in later pts<br>Lateral margins dissected down to lateral border of pectoralis major, subdermal glandular tissue undermined in retroareolar area leaving 1-2 mm of intact dermis; NAC dissection by	In 115 NSM, any NAC necrosis in 26 (11 complete, 15 partial); NAC recurrence 1.3%

Citation	Study name and location	Years of study*	Topic or comparison	Number of patients	Design	Population	Criteria for NSM	Pathology	Oncologic outcomes	Surgery Details	Nipple viability and necrosis
										monopolar cautery using low level of cutting current	
Boneti, 2011 [128]	University of Arkansas for Medical Sciences, Little Rock, Arkansas, USA	1998-2010	TSSM vs. SSM<br>No comparison of selection criteria or surgical procedures	152 pts and 281 breasts TSSM	Retrospective review<br>Follow-up 25.3 ± 18.8 months for TSSM group<br>No multivariate analysis	Breast cancer (94.9%), high-risk lesions (1.7%), prophylactic (3.7%)<br>SLNB: stopped using blue dye because of skin flap necrosis and permanent staining of skin and switched to Tc99m sulfur colloid<br>Immediate reconstruction with implant or expanders	All SSM and TSSM cases with reconstruction; excluded LABC with skin involvement, inflammatory, collagen vascular disease, smoking within previous 6 months, RT if signs of radiation damage<br>TSSM or SSM decided based on NAC involvement, breast size, ptosis<br>TSSM if absence of NAC involvement on clinical exam or imaging (mammography, ultrasound, or MRI)	Nipple core biopsy for intraoperative touch prep and permanent evaluation<br>TSSM converted immediately to SSM if nipple core intraoperatively showed malignancy (2.5%); NAC removed in reoperation if involvement shown on final pathology	LRR in 7 of 152 pts (4.6%)<br>No recurrence in NAC	Skin flaps by developing dissection plane with serial dilation and blunt dissection in the plane between the breast and subcutaneous tissue; at NAC used cold blade or scissors to sharply separate overlying skin, aiming for thickness ≤7 mm	2 NAC necrosis requiring excision (1.3%)<br>9 skin flap necrosis (5.9%)<br>Vertical infra-areolar incision preferred as less severe skin loss; IMF incision can remove the inframammary blood supply
Fortunato, 2013 [129]	San Giovanni-Addolorata Hospital, Rome, Italy	2003-2012	NSM outcomes<br>No comparison of selection criteria or surgical procedures	121 pts, 138 NSM (122 for cancer); of these 124 NSM were in 2009-2012	Retrospective review<br>Median follow-up 26 months	Pts necessitating mastectomy<br>60% multifocal or multicentric cancers, 22% LABC, 3% contralateral, 1.5%	No clinical evidence of NAC invasion or retraction, ≥1 cm tumour to NAC<br>Since 2009 used mammography,	Intraoperative serial histologic exam of retroareolar tissue using toluidine blue and hematoxylin-eosin staining	1 LRR outside NAC; 6 systemic relapse (DM), 1 contralateral cancer, 1 death	Generally radial lateral or italic S incision, paying attention to not compromise periareolar blood supply; skin flaps carefully raised at level of Cooper's	4.3% total NAC necrosis; 4% partial skin flap necrosis; 10% skin desquamation/ minimal necrosis

Citation	Study name and location	Years of study*	Topic or comparison	Number of patients	Design	Population	Criteria for NSM	Pathology	Oncologic outcomes	Surgery Details	Nipple viability and necrosis
						recurrence, 11.5% prophylactic	sonogram, and MRI for all cases<br><br>Inclusion in pts with cancer: ≥1 cm from NAC, negative retroareolar margin; no clinical involvement of NAC, Paget disease, bloody discharge, inflammatory carcinoma	NAC removed (near margin or < 2mm from ink) in 11/31 cases (35%) with TND <1 cm and 8/62 cases (12.9%) with TND > 1 cm; NAC removed more in first half of study, 17/69 vs. 8/69. Of NAC removed, 58% had residual cancer at definitive exam		ligaments with electrocautery at low setting and avoiding excessive traction<br><br>Maintained 2-3 mm retroareolar thickness<br><br>Submuscular tissue expander or prosthesis used	Nipple necrosis or skin desquamation in 25 pts<br><br>8% had some NAC sensation in first month, 17% after 6 months (typically minimal or partial)<br><br>Table II with NAC events is missing from publication
Sakurai, 2013 [130]	Wakayama, Japan	1985-2004	NSM vs. mastectomy<br><br>Mastectomy group not relevant to current review and data not extracted<br><br>NSM outcomes<br><br>No comparison of selection criteria or surgical procedures	788 NSM	NO RT used<br><br>No multivariate analysis<br><br>Median follow-up 87 months	Invasive or non-invasive breast cancer, not restricted to small tumours/early stage; 38.6% stage 1, 28.3% stage 2A, 17.9% stage 2B, 12.1% Stage 3, 0.4% stage 4<br><br>Immediate reconstruction (autologous free dermal fat graft implant) at pt request	NSM if pt desired to preserve NAC, mastectomy if believed risk of recurrence outweighed benefit of NSM<br><br>Other criteria for NSM: macroscopically intact, no involvement by frozen section	NAC resected if evidence of neoplastic involvement on frozen sections	Local recurrence 8.2%: NAC relapse 3.7% (2.7% nipple, 0.5% areola, 0.5% nipple and areola); skin flap 4.6%<br><br>NAC removed if recurrence in this area<br><br>DFS: 5-y 86%, 10-y 83%, 15-y 75%<br><br>OS: 5-y 93%, 10-y 88%, 15-y 87%	NSM incision varied: periareolar, lateral, IMF depending on location and size of tumour<br><br>For NSM, thin flap (≈5 mm) of subcutaneous adipose tissue left close to the tumour and >1 cm thick in location >2 cm from tumour; nipple everted and major ducts removed from the lumen; tissue under NAC carefully treated to preserve vessels that feed the nipple	No nipple necrosis (0/788)

Citation	Study name and location	Years of study*	Topic or comparison	Number of patients	Design	Population	Criteria for NSM	Pathology	Oncologic outcomes	Surgery Details	Nipple viability and necrosis
Stanec, 2014 [131]	University Hospital ''Dubrava,'' Zagreb, Croatia	1997-2012	NSM and SSM experience<br>NSM outcomes<br>No comparison of selection criteria or surgical procedures	361 pts (421 breasts) with NSM or SSM<br>After imaging or frozen section analysis, 252 pts NSM (288 breasts of which 47 or 16% were prophylactic)	Retrospective using medical records<br>NAC sensation reevaluated each examination and end of study (most >1 y follow-up); 0=no sensation and 4=normal sensation	Pts could indicate preference for radical mastectomy, quadrantectomy, oncoplastic BCS, NSM.<br>Autologous reconstruction (mostly LD) in 65.5%, 3.8% LD plus implant, 30.6% implant only	Patients screened for NSM eligibility using mammography, ultrasound, MRI, and biopsy. Pts with cancer in NAC (125 breasts) received SSM.	For NSM, intraoperative frozen section of subareolar tissue found 25 occult involvement of NAC (7.7%) and NAC removed (followed as SSM); on final histological examination 12 (4.2%) had DCIS or invasive cancer and nipple was removed and followed up as NSM	Outcomes after 15 years: local recurrence 3.7%; local recurrence in NAC 1.2%; LRR 5.5%; death from DM 3.6%; DM still alive 0.8%	Omega pattern incision (periareolar with lateral extension) with modification according to breast anatomy	Partial necrosis (epidermolysis), of NAC in 27 pts (9.4%), total or full-thickness necrosis (eschar) in 2 pts (0.7%)<br>Depigmentation persisted in 12/27 pts<br>NAC sensation: 0 (none) 16%, 27% large decrease, 35% less decrease, 22% normal
Rossi, 2015 [132]	Morgagni-Pierantoni Hospital, Forli, Italy	2006-2014	NSM outcomes<br>No comparison of selection criteria or surgical procedures	252 NSM planned, 199 NSM (178 pts) and 53 SSM conducted		23 (9.1%) risk reducing (prophylactic) NSM, 83 (33%) in situ carcinoma, 127 (50.4%) invasive, 19 (7.5%) C5 preoperative cytology<br>Immediate reconstruction with subpectoral implant or expander	Mammography, ultrasound, MRI if available<br>Exclusion if infiltration of skin or NAC (within 2 cm of nipple base), inflammatory, pathologic nipple discharge, Paget disease<br>Relative contraindications: significant large/ptotic breast, active smoking, extensive lymphovascular	After mastectomy was completed a 3-5 mm thick layer of tissue removed from retroareolar area of specimen for frozen section analysis; if neoplastic tissue found then the NAC removed and converted to SSM. By nipple eversion the central ducts are transected at the base of the nipple and dissected, nipple is cored	1 LR<br>0 LR in NAC after NSM<br>6 DM (3%)	Italic S incision from lateral edge of areola to external equatorial line which permits access to axillary lymph nodes; NAC isolation by hydrodissection of the areola	0 complete necrosis of the NAC<br>25 partial transient ischemia of the NAC with epidermolysis<br>Nipple sensitivity and erectile capacity of the nipple insufficient in most pts

Citation	Study name and location	Years of study*	Topic or comparison	Number of patients	Design	Population	Criteria for NSM	Pathology	Oncologic outcomes	Surgery Details	Nipple viability and necrosis
							invasion, previous RT, diabetes, obesity	53 (21%) converted to SSM due to cancer found in retroareolar tissue (all confirmed at definitive histology)<br>Removed NAC had 9 invasive cancer, 38 in situ carcinoma, 6 LIN III<br>If definitive histology positive, then either NAC removal, RT, or follow-up			
Santoro, 2015 [133]	San Giovanni-Addolorata Hospital Rome, Rome, Italy	2007-2015	NSM after NACT<br>NSM outcomes<br>No comparison of selection criteria or surgical procedures	186 NSM, including 51 after NACT	Retrospective review<br>Median follow-up 35 months	Invasive or intraductal carcinoma<br>Minimum follow-up 12 months; 72% for multifocal or multicentric disease, 22% for LABC, 6% for contralateral cancer after previous mastectomy<br>Immediate reconstruction with tissue expander or prosthesis under pectoralis major and serratus	Pts requiring mastectomy, no clinical evidence of NAC invasion or retraction, ≥1 cm clinical-radiological distance between tumour and NAC, no Paget disease, bloody discharge, or inflammatory carcinoma<br>All pts evaluated by mammography, ultrasound, and MRI; whole body CT scan and bone scan if receiving NACT	Intraoperative serial histological exam of retroareolar tissue, using staining; 23 pts had nipple removal due to cancer cells <2 mm from margin and of these NAC showed no residual cancer in 11/23 (48%)	DFS at median 35 months was 89.7%<br>Local relapse 1.6%;<br>No NAC recurrence<br>8.6% systemic relapse (DM) and 5 pts died (3%)<br>4 new contralateral cancers (2%)	Radial lateral or italic “S” incision, paying attention not to compromise periareolar blood supply; skin flaps raised with low setting electrocautery at level of Cooper’s ligaments, avoiding excessive counter traction<br>No more than 2-3 mm retroareolar thickness maintained	Full thickness NAC necrosis in 8 pts (4%)<br>Minimal NAC necrosis or desquamation not requiring surgery, or simple debridement with primary skin closure in 31 pts (17%)<br>Skin flap complications 7%

Citation	Study name and location	Years of study*	Topic or comparison	Number of patients	Design	Population	Criteria for NSM	Pathology	Oncologic outcomes	Surgery Details	Nipple viability and necrosis
						anterior muscles	Excluded large and ptotic breasts at start of study but not later on				
Seki, 2015 [134]	Keio University Hospital, Shinjuku-ku, Tokyo, Japan	2003-2013	Local recurrence NSM vs. mastectomy<br>NSM outcomes<br>No comparison of selection criteria or surgical procedures	121 NSM	Retrospective review of prospective database<br>Median follow-up 28 months	Stage 0-III breast cancer without indication for BCS by MRI, ultrasound, and mammography	NSM not allowed if suspicion of tumour involvement in the NAC or skin by imaging	Frozen section analysis of subareolar tissue in NSM found tumour involvement of NAC in 5 pts (4.1%) and the NAC was removed; 2 (1.7%) additional cases of NAC involvement by permanent section (one had NAC removed, the other had RT)	NSM group:<br>LR in 5 pts (4.1%)<br>5-y LR 7.6%<br>DM in 6 pts (5.0%)<br>5-y distant metastasis 7.4%<br>5-y OS 98.4%<br>In pts with LR, 2 had NAC recurrence and 3 skin flap recurrence	Not reported	Nipple necrosis in 3 pts (2.5%)
Fujimoto, 2016 [135]	Yokohama City University Medical Center, Yokohama, Japan	2004-2010	Outcomes of immediate reconstruction using free flaps after NSM or SSM<br>No comparison of selection criteria or surgical procedures	136 pts: NSM attempted in 107 pts and received in 100 pts; 29 initially SSM plus 7 converted from NSM	Retrospective review<br>Median 75 months follow-up:	Operable stage 0-IIIa, immediate reconstruction using free flaps,	No clinical evidence of NAC involvement, self-selected to possibility of NSM or SSM<br>Exclusions: LABC with skin involvement, inflammatory breast cancer, nipple retraction or discharge	NSM converted to SSM (removal of NAC) because of intraoperative frozen section subareolar tumour positivity in 7/107 pts<br>11 pts (8%) with positive margins in permanent sections (3 partial breast RT, 5 whole breast RT, 3 no RT)	5-y RFS 91.9%<br>Recurrence: 9.6% overall<br>2.9% LR alone, including 0.7% NAC and 2.2% skin<br>2.2% both LRR and DM<br>2.9% DM alone<br>1.5% contralateral<br>2.2% death	Lateral incision along IMF line; subdermal glandular tissue undermined in retroareolar are leaving 1-2 mm intact dermis; SLNB in same incision with injection of indigo carmine around subareolar area	Total NAC necrosis in 1 pt (0.7%)<br>Partial NAC necrosis in 9 pts (6.6%)

Citation	Study name and location	Years of study*	Topic or comparison	Number of patients	Design	Population	Criteria for NSM	Pathology	Oncologic outcomes	Surgery Details	Nipple viability and necrosis
Moo, 2016 [136]	Weill Cornell Medicine, New York, NY, USA	2007-2013	Oncologic outcomes after NSM<br>No comparison of selection criteria or surgical procedures	413 pts (721 NSM)<br>Numbers add to 708 NSM but text says 721 NSM	Retrospective<br>Follow-up median 32 months		Pts given option of NSM depending on breast size and ptosis<br>NSM excluded if NAC involved clinically or on imaging, suspicion of carcinoma-related nipple discharge, inflammatory, Paget disease	Retroareolar biopsy with permanent section; frozen section according to surgeon preference; nipples excised if positive pathology (ASM)<br>28 NAC biopsies (7.6%) positive on either permanent or frozen section; of these 7 were negative on frozen section and positive on permanent section; 20 NAC were removed<br>Pt with ASM were excluded	23 pts (6.3%) with recurrence:<br>8 (2.2%) LRR (1 at NAC);<br>9 (2.4%) DM;<br>6 (1.6%) both LRR and DM<br>Estimated RFS 93.6% at 36 months	NSM via IMF incision	Not reported
Shimo, 2016 [137]	St. Marianna University School of<br>Medicine, Kanagawa, Japan	2000-2013	Surgical and oncologic safety of NSM vs. conventional total mastectomy<br>Only extracted NSM data; no comparison of selection criteria or surgical procedures	413 pts (425 breasts) NSM; 878 total mastectomy	Retrospective<br>Median 46.8 months follow-up<br>Multivariate analysis was not conducted	Primary breast cancer	NSM eligibility: no suspected cancer infiltration to NAC on MRI, extensive intraductal spreading and multicentric disease difficult to treat with BCS, and patient preference	Excluded if retroareolar frozen sections positive for carcinoma (all were negative)<br>Retroareolar biopsies were negative on both frozen and permanent histological diagnosis<br>Recurrence at NAC treated with complete NAC resection	DM 7.5%<br>Survival 96.8% (appears from graph to be 5-y OS)<br>Local recurrence 5.8%, including 10 cases (2.3%) at the NAC<br>NAC recurrence associated with DM and lower survival<br>Survival and recurrence did not differ from	Periareolar or lateral incision and an IMF incision depending on main tumour location, pt preference, and physician consideration; most were lateral incisions<br>Thick cutaneous adipose tissue left unless close to tumour to preserve blood flow to the NAC	96 ischemic nipple complications (22.6%), all which resolved with alprostadil ointment<br>Nipple erosion 0.9%<br>Nipple necrosis 1.4%

Citation	Study name and location	Years of study*	Topic or comparison	Number of patients	Design	Population	Criteria for NSM	Pathology	Oncologic outcomes	Surgery Details	Nipple viability and necrosis
									total mastectomy but no multivariate analysis (excluded from Q3a)	Tissue under NAC treated carefully to avoid nipple necrosis	
Tang, 2016 [138]	Massachusetts General Hospital, Boston, MA, USA	2007-2014	NSM outcomes<br><br>No comparison of selection criteria or surgical procedures	766 pts with 1326 NSM; 27% unilateral and 73% bilateral<br><br>642 (48%) therapeutic and 684 (52%) prophylactic	Median follow-up of 36 months	All pts with NSM and immediate reconstruction	Exclude if clinical or imaging evidence of NAC involvement, LABC with skin involvement, inflammatory cancer, bloody nipple discharge, marked ptosis (for cosmetic reasons)<br><br>TND measured in later pts but not used as criteria to include/exclude	Nipple/subareolar margin specimen containing superficial retroareolar tissue and ductal tissue from nipple sent for permanent pathology; final anterior margin is the underside of nipple and areola dermis; margins considered positive if they contained invasive cancer or DCIS<br><br>Decision to remove NAC or only the nipple made by breast and plastic surgeons. In therapeutic NSM group, nipple or NAC removed for 9/11 (82%) when margins had invasive cancer and 28/32 (88%) with DCIS<br><br>Of 19 pts with nipple-only excision for positive margins, 2	Positive margins in 6.7% therapeutic and 0.4% prophylactic NSM<br><br>In NSM with positive nipple margins, no recurrences occurred at nipple/NAC excision site<br><br>In breasts with positive nipple margins there were 3 chest wall recurrences (2 in observation alone and 1 with NAC excision); in breast with therapeutic NSM and negative margins there were no recurrences at nipple/NAC but 6 (1%) chest wall recurrences	Flaps raised in Cooper's ligament plane, leaving <1 cm subcutaneous fat in most pts<br><br>No subcutaneous fat was left under areola<br><br>Rate of positive margins decreased over time (11% in 2007-2011 vs. 5.4% in 2012 to 2014).<br><br>Removal of full NAC decreased over time; nipple only was removed in 38% in 2007-2011 and 62% in 2012-2014	Total nipple necrosis leading to nipple loss in 18 breast (1.4%)<br><br>In pts without previous RT or PMRT, nipple necrosis was 0.9% and breast skin necrosis 2.4%

Citation	Study name and location	Years of study*	Topic or comparison	Number of patients	Design	Population	Criteria for NSM	Pathology	Oncologic outcomes	Surgery Details	Nipple viability and necrosis
								had DCIS but clear areolar margins and the 3rd had ILC with partial areola excision<br><br>Precursor lesions such as LCIS, atypical lobular hyperplasia, atypical ductal hyperplasia, flat epithelial atypia were documented but not considered positive margins and nipple/NAC was not routinely excised			
Smith, 2017 [139]	Massachusetts General Hospital, Boston, MA, USA	2007-2012	Long term oncologic safety of NSM<br><br>No comparison of selection criteria or surgical procedures	297 pts, 311 NSM	Retrospective review<br><br>51 months median follow-up	Most pts had implant reconstruction (65.0% single stage, 31.4% expander, 2.2% tissue flap)<br><br>18% received PMRT	Exclusion: radiologic or clinical evidence of nipple involvement; LABC with skin involvement, inflammatory breast cancer, bloody nipple discharge, or if breast size and/or ptosis would result in unacceptable nipple location<br><br>Preoperative MRI used in 35.5% at surgeon discretion	If nipple margin in permanent sections contained invasive cancer or DCIS it was considered positive and nipple excised, often with retention of most of the areola<br><br>Nipple margin positive in 20 breasts (6.4%) of which 10 excised nipple papilla and 9 entire NAC, 1 pt had tumour 2mm from inked margin and had no additional surgery	3-y DFS 95.7%, 5-y DFS 92.3%<br><br>17 recurrence: 11 (3.7%) LRR and 8 (2.7%) DM (this includes 2 pts with both LRR and DM)<br><br>No recurrence in NAC in this group of 311 pts or entire 2182 NSM conducted from 2007-2016<br><br>No recurrence at site of excised nipple or NAC for pts treated for positive margins	Incision placement at surgeon discretion; majority used inferolateral incisions<br><br>Skin flap raised in Cooper's ligaments plane, usually with electrocautery<br><br>At NAC, areola skin flaps raised leaving nipple duct bundle intact to be sharply divided immediately below the NAC dermis and sent permanent pathology<br><br>Frozen section used rarely because less accurate, and difficult to	1.7% total nipple necrosis resulting in NAC excision in study with overlapping pt population that included 51.2% NSM for risk reduction (see Coopey, 2013 [140])

Citation	Study name and location	Years of study*	Topic or comparison	Number of patients	Design	Population	Criteria for NSM	Pathology	Oncologic outcomes	Surgery Details	Nipple viability and necrosis
										distinguish benign atypia from DCIS	
Agresti, 2017 [141]	NCT02471742<br><br>Fondazione IRCCS Istituto Nazionale dei Tumori, Milan, Italy	2009-2013	Oncologic safety of NSM after NACT (primary chemotherapy, PC)<br><br>No comparison of selection criteria or surgical procedures	422 pts NSM: 361 first line NSM (no NACT) and 61 after NACT (NSM-PC)	Propensity-score matching<br><br>Match 2: cT2-3 pts before NACT using clinical tumour size: 61 NSM-PC and 61 NSM;<br><br>Match 3 using pathological tumour size (after NACT if used): pT1-3 after NACT: 61 NSM-PC and 183 NSM<br><br>Annual follow-up; median follow-up 42.5 months NSM, 46.0 months NSM after NACT	Invasive breast cancer,<br><br>NACT group: T2-T3N0-N1<br><br>Non-NACT group: T1-T3N0-N1<br><br>Excluded progressive disease during NACT, synchronous DM, other clinical disease affecting optimal therapy<br><br>2-stage submuscular or 1 stage dual-plane implants	Tumour nodule without skin adherence<br><br>TND <1 cm allowed<br><br>Exclude if nipple retraction, Paget disease, inflammatory changes of the breast, bloody nipple discharge	Sample for frozen analysis from base of NAC analyzed; invasive or DCIS in main ducts evaluated<br><br>Include in study only if retroareolar main ducts free of neoplastic tissue at frozen section examination<br><br>NAC involvement in frozen section or final pathology in 54 pts (12.8%, of which 3.1% was infiltrating and 9.7% DCIS); subdivided by NACT it was 13.3% NSM, 9.8% NSM-PC<br><br>51/54 pts with involvement had NAC resection and 3 had RT<br><br>No further disease found in excised NAC in 30 pts; involvement found in 21 pts (5 infiltrating, 16 non-infiltrating)	NAC and NAC-PC groups not equivalent before matching so difference is likely not due to NACT<br><br>LR 2.8% NAC; 9.8% NAC-PC<br><br>1 pt in NAC-PC group had LR in NAC	Radial Italic S-like incision in equatorial/upper external site of the breast if A-C cup size [was very small number of larger breasts D or DD]<br><br>Skin layer 1-2 mm thick left to preserve essential capillaries supplying the skin; fascia of major pectoral muscle preserved if oncologic safety allowed, areola dissected away from underlying tissue even if thin disc of gland tissue remained<br><br>If tissue containing main ducts under nipple was observed, the nipple was inverted for complete removal of this tissue which was sent for frozen section examination	Data in Figure 2 appear mislabeled in comparison to text and Table 6<br><br>1 pt had NAC necrosis due to insufficient vascular supply and had NAC resection

Citation	Study name and location	Years of study*	Topic or comparison	Number of patients	Design	Population	Criteria for NSM	Pathology	Oncologic outcomes	Surgery Details	Nipple viability and necrosis
Cont, 2017 [142]	Candiolo Cancer Institute-FPO, IRCCS, Candiolo (Turin), Italy	2010-2015	Oncologic safety of NSM; factors correlated with subareolar and/or nipple duct involvement<br>No comparison of selection criteria or surgical procedures	518 pts	Retrospective<br>Mean follow-up of 33 months	Invasive or DCIS, not amenable to BCS, willing to have immediate reconstruction<br>Excluded: prophylactic mastectomy or for non-malignant lesions or LCIS	All pts with breast cancer and scheduled for NSM had preoperative breast MRI and intraoperative assessment of NAC status<br>No clinical evidence of NAC or skin involvement<br>Exclusion: LABC and no NACT or no response to NACT, inflammatory, Paget disease,	If subareolar ducts or proximal nipple ducts were involved (invasive carcinoma, DCIS, ductal intraepithelial neoplasia DIN1c-DIN3) in frozen section or definitive pathology (26.1% of cases), the NAC was removed unless the pt refused (19 pts)<br>Intraoperative pathology subareolar or nipple ducts found 100 involved vs. 135 final pathology of ducts; however, NAC was only involved in 45% of the NAC removed<br>Positive margin defined as margin with ink on tumour	2.7% local relapse 12/14 were in same quadrant as the primary tumour<br>No relapse in patients with PMRT<br>One case (0.2%) of NAC recurrence as Paget disease of the nipple	Details of biopsy reported but not mastectomy<br><u>Other</u><br>Involvement of subareolar ducts or nipple ducts only correctly predicted NAC involvement in 45% of NAC resected<br>Authors suggest adjuvant RT to the tumour site instead of NAC	Not reported
Huang, 2018 [143]	Guangxi Medical University Affiliated Tumor Hospital, Nanning, Guangxi, China; and Liuzhou People's	2007-2016	Recurrence and survival in young patients<br>NSM outcomes<br>No comparison of selection criteria or surgical procedures	163 NSM; including 58 pts stage IIA	Median follow-up 39 months	Stage 0-IIB breast cancer, age <35 y, adjuvant treatment; contraindication to BCS by imaging (MRI, ultrasound, mammography) or pts who	NSM excluded if possible tumour involvement of NAC or surrounding skin by imaging, tumour to NAC distance <2 cm, nipple discharge, Paget's disease	Frozen section of retroareolar tissue to confirm no invasion of NAC borders, but no mention of results	Recurrence 22 pts (13.5%)<br>LR only: 6 cases (3.7%)<br>Systemic recurrence only: (DM) 15 cases (9.2%)	Puncture point for biopsy as far from NAC and close to lump as possible	Nipple necrosis in 4 pts (2.5%)<br>Partial necrosis of breast skin or autologous flap 11.0%; complete necrosis 0.6%

Citation	Study name and location	Years of study*	Topic or comparison	Number of patients	Design	Population	Criteria for NSM	Pathology	Oncologic outcomes	Surgery Details	Nipple viability and necrosis
	Hospital Liuzhou, Guangxi, China					rejected BCS, immediate implant or autologous reconstruction (but allowed delayed in 9.2%)<br>Excluded bilateral cancer, neoadjuvant treatment, no pathological data, no follow-up records<br>NSM for pts contraindicated for BCS by imaging or pt preference			LR+DM: 1 pt (0.6%)<br>Of LR cases, 2 had NAC recurrence<br>5-y LR 4.3%<br>5-y DFS 86.5%<br>5-y OS 94.5%		
Dornellas de Barros, 2019 [144]	Hospital Sírio-Libanês, São Paulo, Brazil	2005-2015	Oncological safety of NSM<br>NSM outcomes<br>No comparison of selection criteria or surgical procedures	152 pts (161 breasts)	Retrospective<br>Mean follow-up 43.5 months	Infiltrating breast cancer, clinically negative axilla or axilla with movable level I-II lymph nodes cN0-cN1), negative SLNB<br>Reconstruction with silicone implants in 84.9%	Tumour diameter <3.0 cm, TND by imaging and physical examination >2.0 cm<br>Exclusion: clinical evidence of skin/NAC involvement, occult breast cancer, nipple discharge and more than 3	Include if clear margins on intraoperative and definitive analysis<br>Sub-NAC margin analyzed by imprint cytology and frozen sections; if negative in frozen and paraffinized sections the NAC was preserved; if positive margins in any examination	7 LR (4.4%), 4 DM (2.6%), 5 deaths (3.3%)<br>5-y LRFS 97.6%, 5-y RFS 98.3%, 5-y OS 98.3%<br>No NAC recurrence	Either total skin sparing or removing a small paddle of skin over the tumour<br>Vertical radial incision from areola to IMF going around up to 25% of the areolar circumference I the axillary direction was most common<br>Skin flaps raised with a diathermy knife: cut made in the thin fascia between the	Not reported

Citation	Study name and location	Years of study*	Topic or comparison	Number of patients	Design	Population	Criteria for NSM	Pathology	Oncologic outcomes	Surgery Details	Nipple viability and necrosis
							centres/foci of neoplasia	the NAC was removed<br><br>Nipple inverted, ducts arranged inside the central bundle were excised and examined in definitive analysis		subcutaneous fat and glandular tissue; removal of mammary glandular corpus and axillary Spence tail along the pectoralis major muscle fascia, leaving flaps ≈0.5 cm in sub-NAC area and 0.5-1.0 cm elsewhere<br><br>If superficial and peripheral neoplasia ≥2 cm from areolar border and ≤2 cm in depth from skin, an elliptical skin paddle incision was made in the overlying tumour area and might be extended to areolar border<br><br>Perforator branches from 2nd and 3rd internal thoracic vessels had to be preserved to maintain NAC irrigation<br><br>Nipple was inverted after gland removal and ducts in central bundle were excised with a pointed-end knife	
Ng, 2019 [145]	National Cancer Centre Singapore and	2005-2015	Surgical and oncologic outcomes after NSM	130 pts, 139 NSM	Median 43 months follow-up	Mostly early-stage cancer (89%), 86% for cancer treatment and	NSM not used if cancer involving NAC (assessed by imaging, with additional	Intraoperative frozen section of nipple base for all pts, nipple preserved only if	12 (10%) recurrence<br><br>5 (4%) local recurrences,	5 main incisions used: 65% periareolar with or without extension, 22% radial, 11% unknown	2 (1.4%) complete NAC necrosis

Citation	Study name and location	Years of study*	Topic or comparison	Number of patients	Design	Population	Criteria for NSM	Pathology	Oncologic outcomes	Surgery Details	Nipple viability and necrosis
	Singapore General Hospital		No comparison of selection criteria or surgical procedures			14% risk-reduction; 80% autologous reconstruction	magnification of retroareolar region or MRI if requested by surgeon) or inflammatory cancer	no malignancy or atypical cells	including 2 NAC recurrences<br>7 (6%) DM<br>2-y OS 97%, 5-y OS 90%		13 (9%) partial NAC necrosis
Valero, 2020 [146]	Memorial Sloan Kettering Cancer Center, New York, NY, USA	2003-2016	Indications, complication, and long-term outcomes of therapeutic NSM<br>No comparison of selection criteria or surgical procedures	449 pts, 777 NSM: 467 NSM for cancer and 310 contralateral prophylactic NSM	Retrospective<br>Median follow-up 39.4 months	Invasive cancer (72%) or DCIS (27%)<br>96.0% stages 0-II, 94.7% had SLNB or ALND, 7.9% PMRT<br>Excluded bilateral prophylactic or risk-reducing without cancer diagnosis<br>87.1% expander/implant reconstruction, 10.0% implant	375 (76.4%) of NSM were in 2011 or later, 65.5% had MRI<br>Exclusion: LABC, extensive disease in periphery of the breast, direct invasion of the nipple, tumours ≤1 cm from nipple on imaging,<br>Methods indicate pts with risk factors of nipple necrosis were excluded (prior radiation, smoking, cup size C or larger) but results state 5.6% were current smokers and 7.1% had prior radiation	21 (4.5%) nipple excisions, including 14 with involved nipple margin	15 (3.3%) recurrence, 7 (1.6%) deaths<br>3 LRR, 11 DM, 1 LRR plus DM<br>No recurrences in NAC	Not reported	Not reported
Vladimir, 2019 [147]	Oncology Institute of Vojvodina, Serbia	2010-2015	Complications and risk factors of NSM	246 pts	Retrospective<br>Median follow-up 5 y	Pts with cancer histopathologically confirmed on core biopsy or time of	Preoperative clinical and imaging (ultrasound, mammography;	All pts had fast frozen section examination of subareolar core tissue; cancer	4 (1.6%) LR<br>11 (4.5%) DM	88.62% curve incision in upper lateral quadrant, 11.38% semicircle incision on areola edge	2 (0.8%) NAC necrosis; 3 (1.2%) skin and NAC necrosis

Citation	Study name and location	Years of study*	Topic or comparison	Number of patients	Design	Population	Criteria for NSM	Pathology	Oncologic outcomes	Surgery Details	Nipple viability and necrosis
						surgery and having immediate submuscular reconstruction with silicone implants	MRI if could not otherwise exclude multicentricity)	detected 18 pts (6.82%) and converted to SSM and not included in study			Periareolar incision accounted for 11% of NSM but 51% of complications<br>Complication rate 70.4% with periareolar incision and 8.3% with lateral incision, p>0.05 (note based on only 27 periareolar and 218 lateral incisions)
Li, 2020 [148]	Affiliated Hospital of Xuzhou Medical University, Xuzhou, Jiangsu, China	2014-2015	Pt satisfaction and aesthetic outcome<br>No comparison of selection criteria or surgical procedures	215	Follow-up 24 months	Histopathologically diagnosed breast cancer<br>Excluded tumour completely adherent to chest wall, history of smoking	Tumour margin >2 cm from NAC by high resolution ultrasound (but results say mammography)<br>Excluded tumour approaching the NAC, tumour margin < 2 cm, NAC involvement	Biopsy tissue from nipple base, beneath the areola, and subcutaneous tissue of the skin of the tumour site were evaluated; none were positive by frozen-section biopsy or permanent section	No local recurrence	IMF incisions in methods but lateral incision in results<br>A very steep and sharp dissection for mastectomy, then fascia of pectoralis muscle removed; dissection under subdermal area and the flap of 2-3 mm dermis and very thin subcutaneous fat layer was raised<br>Papilla of nipple left untouched	No comparison of different incisions; type of incision used is ambiguous
Metere, 2020 [149]	Italy	2002-2017	Long-term outcomes of NSM	894 pts	Retrospective	>10 months follow-up	Tumour to NAC ≥2 cm	Histological examination of retroareolar ducts;	LRR in 76 pts (8.5%): local 44 pts (4.9%),	Skin incision on case-by-case basis: radial, italic S, IMF	NAC necrosis in 57 pts (6.4%)

Citation	Study name and location	Years of study*	Topic or comparison	Number of patients	Design	Population	Criteria for NSM	Pathology	Oncologic outcomes	Surgery Details	Nipple viability and necrosis
			No comparison of selection criteria or surgical procedures		Follow-up 18 to 60 months	Immediate reconstruction with tissue expanders or definitive implants		NAC removed if positive results	regional 32 pts (3.6%)<br>DM in 26 pts (2.9%)	Subcutaneous tissue dissected with electrical scalpel; thickness of subcutaneous tissue must be uniform, except 2-3 mm at NAC<br>Unstick the gland from the pectoral fascia and open axillary cavity for SLNB	Nipple necrosis in 25 pts (2.8%)<br>Depigmentation in 28 pts (3.1%)
Parvez, 2020 [8]	McGill University Health Centre, Montreal, Quebec, Canada	2013-2018	Surgical and oncologic outcomes of NSM<br>No comparison of selection criteria or surgical procedures	175 pts	Retrospective review<br>Median 24 months follow-up	Primary or recurrent invasive or in situ breast cancer	NAC decision made by surgeon; included NSM for revision of margins and incidental breast cancers following prophylactic NSM	Intraoperative frozen sections of NAC margin obtained if there was clinical suspicion of a close or involved margin; excluded if histological NAC involvement found<br>Positive margins defined as invasive or in situ disease at inked margin; close margin as DCIS within 2 mm of inked margin	LR in 8 cases (4.6%) including 1 NAC recurrence<br>12 (6.9%) DM<br>OS 98.3% (3 deaths)<br>DFS 88.6%<br>Positive margins 10 pts (5.7%), including 3 (1.7%) NAC and 7 (4.0%) non-NAC; also 4 (2.3%) NAC close margins; 4 NAC excised	52.6% used lateral incision, 18.9% inframammary, 15.4% wise pattern (skin reduction)<br>13 NSM involved free nipple grafting	Nipple necrosis, 4 cases (2.2%) requiring surgical debridement (2 of which had free nipple grafting)
Scardina, 2021 [150]	Sacred Heart Catholic University, Rome, Italy	2018-2021	Experience with NSM and immediate prosthetic prepectoral reconstruction without ADM	209 pts, 269 breasts	Retrospective review<br>Median follow-up 14 months	NSM when BCS could not give adequate local control (cannot get clear margins) or cosmetic results (large tumour	Clinical assessment, ultrasound, mammography, MRI<br>Exclusions for NSM: inflammatory,	Retroareolar tissue marked with surgical thread and excised for frozen section analysis	1 (0.48%) LR<br>2 (0.96%) regional axillary node recurrence	NSM through radial incision on external quadrants, with axillary or IMF incision in selected pts<br>Skin carefully dissected off breast:	2/209 (0.96%) had full thickness (complete) necrosis that required excision

Citation	Study name and location	Years of study*	Topic or comparison	Number of patients	Design	Population	Criteria for NSM	Pathology	Oncologic outcomes	Surgery Details	Nipple viability and necrosis
			No comparison of selection criteria or surgical procedures			compared with breast size, extensive or multicentric disease, contraindications to RT, patient preference)<br><br>Relative (not absolute) contraindications: obesity (BMI >30 kg/m^2, large breasts with severe ptosis, previous RT, active smoking<br><br>Implant with micropoly-urethane foam coated shell surface in subcutaneous plane	LABC infiltrating skin or NAC<br><br>Decision on reconstructive plane made in operating room after accessing flap thickness and perfusion: perfusion with indocyanine green dye fluoroangiography and photo dynamic eye (PDE) imaging system			mastectomy skin flap elevated from glandular tissue and dissected off breast by electrocautery; dissection of skin flaps and NAC; elevated gland on the PP plane preserving superficial pectoral fascia and avoiding medial perforators; retroareolar tissue marked with surgical thread and excised for frozen section analysis; skin flaps trimmed if needed to remove residual breast tissue	
Webster, 2023 [151]	Massachusetts General Hospital, Boston, MA, USA	2008-2019	Oncologic safety of NSM in BRCA mutation carriers with breast cancer<br><br>No comparison of selection criteria or surgical procedures	105 pts, 114 therapeutic NSM<br><br>99 (94%) pts had bilateral NSM, including 5 (4.8%) for bilateral cancer	Retrospective review of single institution database<br><br>Median 70 months follow-up<br><br>Intention-to-treat analysis, even if nipple	Pts with breast cancer and BRCA1 or BRCA2 mutation who had NSM and immediate reconstruction<br><br>Included contralateral prophylactic mastectomy where cancer was found on	Excluded if direct involvement of nipple or areola on physical examination or imaging, inflammatory breast cancer, skin involvement	Nipple/subareolar margin status by permanent histopathology assessment and excision recommended if invasive cancer or DCIS found<br><br>5 (4.4%) positive nipple margins on final pathology; all had nipple excision	No recurrence in retained NAC or site of nipple excised for positive margins<br><br>LRR 3 pts, 2.6%<br><br>DM 4 pts, 3.8%<br><br>OS 96% (4 deaths)<br><br>BCSS 97% (3 deaths)	Removal of ductal tissue from within and beneath nipple and areola<br><br>Indicates surgical details in Colwell, 2010 [152]<br><br>Inferolateral incision that preserves NAC viability	1 pt with bilateral cancer had partial NAC necrosis in both breasts; nipples were preserved after surgical debridement

Citation	Study name and location	Years of study*	Topic or comparison	Number of patients	Design	Population	Criteria for NSM	Pathology	Oncologic outcomes	Surgery Details	Nipple viability and necrosis
					subsequently removed	final histopathology examination (4 cases (4.3%)<br>Excluded if pt and surgeon believed final nipple position and cosmetic result would be poor, metastatic disease within 4 months of initial cancer diagnosis, and pts with <1 y follow-up		(3 with NAC excision)	Rates are for therapeutic NSM for LRR, per pt for DM and survival<br>No new cancers in concurrent prophylactic mastectomies that were negative on initial testing		
Zarba Meli, 2023 [153]	Italy	2010-2020	NSM after NACT<br>No comparison of selection criteria or surgical procedures	417 pts and 433 NSM: 111 pts with 112 NSM after NACT; 306 pts with 321 NSM without NACT	Retrospective<br>Median follow-up 69 months	Breast cancer requiring mastectomy<br>Excluded recurrent or bilateral cancer<br>Immediate reconstruction with expander or prosthesis	Excluded if bloody nipple discharge, Paget disease, clinical or radiologic evidence of NAC invasion, inflammatory<br>Pts receiving NACT were assessed for systemic disease using whole-body CT and bone scan or total body PET and CT; MRI used after NACT to assess response<br>In NACT group, 20 pts with TND	Close margins were <2 mm from inked surface; pts with positive margin in retroareolar area (ink on tumour) advised to have NAC resection; other cases discussed<br>NAC removed in 17 cases (3.9%) for inadequate margins intraoperatively or at definitive pathology; definitive pathology showed no cancer in NAC in 7 cases	At 5 years:<br>LR in 14 pts (3.2%); 7% with NACT, 2% without<br>LRR in 21 pts (4.8%); 9.8% vs. 3.2%<br>No significant difference between NACT and no NACT after adjusting for stage<br>No LRR in 34 pts with pCR and 14% in NACT pts with residual disease	In early phase used radial lateral or italic “S” incision; since 2015 used IMF approach<br>Obtained thin skin flaps (≈3-5 mm) especially in the retroareolar area	Full-thickness NAC necrosis 17 NSM (3.9%), minimal NAC necrosis 60 NSM (13.8%)

Citation	Study name and location	Years of study*	Topic or comparison	Number of patients	Design	Population	Criteria for NSM	Pathology	Oncologic outcomes	Surgery Details	Nipple viability and necrosis
							<1 cm before NACT and response allowed NSM in 16		1 NAC recurrence (pt without NACT)<br>5-y OS 95.1%<br>5-y DFS 87.6%		
Wu, 2019 [154]	Asan Medical Center, Seoul, Korea	2003-2015	Long-term outcomes after NAC in invasive cancer<br>No comparison of selection criteria or surgical procedures	944 pts, 962 NSM	Retrospective<br>Median follow-up 85 months; every 3-6 months for 5 years then annually	NSM and immediate reconstruction for invasive cancer<br>Excluded NACT or palliative surgery<br>Immediate autologous or prosthetic reconstruction	Indications for NSM: clinically normal NAC and no skin involvement offered option of NSM; shape, colour, palpated features of nipple normal	Retroareolar frozen section in all cases; subdermal glandular tissue undermined in retroareolar area leaving 1-2 mm intact dermis; thin layer of glandular tissue collected under areola for review; NAC preserved if NAC ducts tumour free	39 recurrence (4.1%) at NAC as first event<br>42 cases (4.4%) LR outside NAC as first event<br>5-y recurrence at NAC 3.5% (n=34); 5-y local recurrence outside NAC 3.4% (n=33)<br>10-y DMFS 89.3% in pts with recurrence at NAC and 94.3% in pts without recurrence<br>10-y OS 100% in pts with recurrence at NAC and 94.5% without recurrence (overall 94.7%)	85.9% lateral radial incision, 11.1% periareolar with lateral extension, 3% other<br>Subdermal glandular tissue undermined in retroareolar area leaving 1-2 mm intact dermis; thin layer of glandular tissue under areola for review after frozen section obtained; SLNB or ALND	Not reported
Wu, 2020 [12]	Asan Medical Center, Seoul, Korea	2003-2015	LRR after NSM in pts with DCIS and immediate	199 NSM	Retrospective<br>Median follow-up 97 months	Consecutive pts with pure DCIS and breast reconstruction	Inclusion: clinically normal nipple and no skin involvement;	Retroareolar frozen-section biopsy in all pts; nipple ± areola immediately	10-y LRR 4.5%, including 3% NAC recurrence<br>10-y OS 98.5%	SLNB in most (91.5%) pts and none were positive	Not reported

Citation	Study name and location	Years of study*	Topic or comparison	Number of patients	Design	Population	Criteria for NSM	Pathology	Oncologic outcomes	Surgery Details	Nipple viability and necrosis
			reconstruction without RT<br>No comparison of selection criteria or surgical procedures			NSM indications: significant extension of DCIS compared with breast volume, multicentric disease, margin involvement after BCS, pt preference<br>Exclusion: microinvasion, history of prior RT, synchronous contralateral invasive breast cancer	shape, colour, palpated features of nipple were normal<br>Positive surgical margin defined as tumour touching ink in the mastectomy specimen	removed if positive for malignancy and converted to SSM or ASM<br>If retroareolar tissue positive at final pathology, the nipple ± areola was removed and pt excluded from NSM cohort<br>No pts had evidence of tumour involvement at retroareolar resection margin	LRR as first event in 10 pts (5%): 5 had NAC recurrence, 3 chest wall, one axillary node, and 1 NAC + bilateral axillary lymph node metastasis	Surgical details not reported	
Wu, 2021a [155]; Wu, 2021b [156]; Wu, 2022b [157]	Asan Medical Center, Seoul, Korea	2003-2016	LRR; DM and survival after local recurrence after NSM<br>Reconstruction loss due to LR after NSM<br>No comparison of selection criteria or surgical procedures	1696 pts	Retrospective<br>Median follow-up 84 months: follow-up every 3-6 months for 5 y then annually	Primary breast cancer with immediate reconstruction<br>Any tumour stage, size, with indications for mastectomy<br>Immediate autologous or prosthetic reconstruction	Inclusion: clinically normal nipple and no skin involvement; shape, colour, palpated features of nipple were normal; any TND	Retroareolar frozen-section biopsy confirmed to be tumour-free	LRR as first event in 172 pts (10.1%): 117 (6.9%) LR alone, 44 (2.6%) regional alone; 11 pts (0.6%) LR + regional; therefore LR 128 pts or 7.5% and regional 55 pts or 3.2%<br>52 (3.1%) involving NAC<br>DM in 30 cases (1.8%)<br>Subset of pts with recurrence	SLNB or ALND in all pts<br>Surgical details not reported<br>Thickness of remaining skin flap generally 7-8 mm; SLNB or ALND	Not reported<br>Reconstruction loss in 21/128 (16%) of pts with LR

Citation	Study name and location	Years of study*	Topic or comparison	Number of patients	Design	Population	Criteria for NSM	Pathology	Oncologic outcomes	Surgery Details	Nipple viability and necrosis
									and median post-recurrence follow-up of 54 months:<br>• In 172 pts with LRR: 5-y post-recurrence DFS was 73.7% (89.1% for NAC recurrence, 73% for skin/chest wall, 59.4% for regional recurrence groups)<br>• In 172 pts with LRR: 5-y post-recurrence DMFS was 79.4% (96% for NAC, 82.8% for skin/chest wall, and 59.7% for regional recurrence groups)<br>• In 172 pts with LRR: 5-y post-recurrence OS 91%		
Wu, 2021d [158]	Asan Medical Center, Seoul, Korea	2010-2016	NSM after NACT<br>No comparison of selection criteria or surgical procedures	310 pts, 319 NSM	Retrospective<br>Mean follow-up 63 ± 22 months	All pts with NSM and NACT for breast cancer<br>Indications for NSM: any stage, size	Inclusion: any TDN, clinically normal nipple; normal shape, colour, and palpated features of nipple; no skin	Retroareolar frozen-section biopsy and permanent biopsy: nipple ± areola removed if nipple margin was positive for	LRR as first event in 38 cases (11.9%), including 6 (1.9%) in the NAC<br>Of LLR, 13 had isolated LRR, 16	Not reported	Not reported

Citation	Study name and location	Years of study*	Topic or comparison	Number of patients	Design	Population	Criteria for NSM	Pathology	Oncologic outcomes	Surgery Details	Nipple viability and necrosis
						Exclude synchronous distant metastasis, recurrence disease	involvement or inflammatory cancer	malignancy and converted to SSM or ASM and excluded from NSM cohort<br><br>pCR in 40 pts (12.5%)	had regional recurrence, 2 concurrent LR and regional recurrence, 7 LRR and DM<br><br>DM as first event in 37 pts (11.9%)<br><br>25 deaths due to breast cancer (8.1%)<br><br>5-y cumulative LRR 11.0% including 1.9% NAC<br><br>5-y LRR-free survival 87.3%, DM-free survival 87.8%, OS 91.3%<br><br>CBC 3.9%		
Wu, 2022a [159]	Asan Medical Center, Seoul, Korea	2010-2016	NACT: NSM + IBR vs. CM alone (data not extracted) in LABC<br><br>No comparison of selection criteria or surgical procedures	217 NSM	Follow-up every 3 to 6 months for 5 years then annually; mean follow-up 70 months	Clinical stage IIB to IIIC, NACT, indications for mastectomy; immediate reconstruction<br><br>Excluded T4, recurrent	Excluded inflammatory<br><br>NSM offered if no involvement of NAC or skin clinically and on imaging (MRI, ultrasound, or mammography) after NACT; also offered if initial nipple or subareolar involvement before but not after NACT	If retroareolar frozen section or permanent biopsy margin was positive, NSM was converted to SSM or ASM and excluded from NSM group	LR 6.7%, including 3 pts (1.4%) at the NAC<br><br>Regional recurrence (internal mammary or supraclavicular lymph nodes or ipsilateral axillary) 9.6%<br><br>Distant metastasis 19.6%<br><br>6-y local RFS 91.6%	Not reported	Not reported

Citation	Study name and location	Years of study*	Topic or comparison	Number of patients	Design	Population	Criteria for NSM	Pathology	Oncologic outcomes	Surgery Details	Nipple viability and necrosis
							In NSM group, median TND 2.4 cm; 29.2% had TND ≤1 cm before NACT; 30 pts (14.4%) with tumour extension in subareolar area before NACT but resolved after NACT and underwent NSM		6-y DFS 70.5%<br><br>6-y Distant metastasis-free survival 79.8%<br><br>6-y OS 87.6%<br><br>No nipple recurrence in pts converted by NACT from involved to resolved subareolar extension		
Petit, 2009 [160]	European Institute of Oncology, Milan, Italy	2002-2007	ELIOT (800 pts) or delayed one-shot RT following operation (201 pts)	1001 NSM, including 29 bilateral	Median follow-up 20 months based on 83% of pts	Carcinomas or DCIS	Inclusion criteria: primary tumours ≥1 cm outside areola margins, absence of nipple retraction or bloody discharge and absence of retroareolar microcalcifications; multifocality accepted as long as all tumour sites were distant from the areola	Intraoperative frozen section (repeated if initially positive) and assessment of blood supply by local bleeding and colour of the NAC by the plastic surgeon<br><br>1171 NSM, of which 131 excluded (68 positive intraoperative frozen section of retroareolar tissue on both sections; poor blood supply and high risk of NAC necrosis contraindicating RT); NAC removed and ELIOT not used<br><br>In 1,001 included pts, first frozen	14 (1.4%) LR of which 10 close to tumour site and all far from NAC (no recurrence in NAC)<br><br>LR ELIOT 1.6% vs. delayed 0.5%, p=0.22<br><br>No recurrence in preserved NAC with positive final pathology (86 cases) nor in those that were positive in first but not second frozen section (79 cases)<br><br>DM in 36 pts (3.6%)	Skin incision above the tumour, dissected glandular tissue from the plane of the dermis and from the pectoral fascia; thin layer of glandular tissue left beneath areola to preserve blood supply<br><br>Thin specimen from retroareolar area for immediate frozen section; when positive a further layer of tissue removed underneath the NAC and if also positive the NAC was removed<br><br>ELIOT delivered to NAC in a single fraction in 800 NSMs; in other pts RT was	NAC total necrosis in 35 cases (3.5%), NAC partial necrosis in 55 cases (5.5%); NAC was removed in 50 cases (5%)<br><br>Average sensitivity of areola and periareolar area was 2 out of 10; partial NAC sensitivity recovered in of in 15% of pts at one year after operation<br><br>Slight change in areolar

Citation	Study name and location	Years of study*	Topic or comparison	Number of patients	Design	Population	Criteria for NSM	Pathology	Oncologic outcomes	Surgery Details	Nipple viability and necrosis
								section was positive but not the second exam of retroareolar tissue in 81 cases and NAC was preserved<br>In 1,0001 included pts, final pathology found cancer cells in 86 cases (8.6%); 79 NAC were preserved (23 invasive, 53 intraductal)	DM ELIOT 3.5% vs. delayed 4%, p=0.74<br>4 deaths (0.4%)<br>No significant difference between ELIOT and delayed RT<br>Lack of recurrence in NAC is promising, but is short follow-up	delayed until after the operation due to poor vascularization of the nipple that required further observation	pigmentation in 20% of pts
Lohsiriwat, 2012 [161]	European Institute of Oncology, Milan, Italy	2002-2008	NSM with ELIOT; focus on Paget disease in recurrence	861 NSM	Median follow-up 50 months	713 invasive and 148 intraepithelial neoplasia; Stages I-III<br>ELIOT to areolar plus 1 cm margin around then immediate breast reconstruction with implant or autologous tissue	Inclusion: no clinical or radiological nipple involvement, no inflammatory signs, no previous irradiation<br>Excluded: bilateral, LABC, prophylactic mastectomy, neoadjuvant RT or chemotherapy	Excluded if positive margins on retroareolar frozen section	36 (4.18%) local recurrence, including 7 (0.8%) Paget disease<br>11 (1.3%) NAC recurrence (including 7 Paget disease)<br>Average latency from NSM to Paget disease LR was 32 months	Sharp dissection using surgical blade and/or diathermy knife; dissection at plane just beneath the dermis allowing total removal of breast parenchyma and retention of subdermal vessels and thin layer of subcutaneous fat and subdermal vascular network<br>5 mm extra-areolar flap and areolar flap recommended<br>Terminal ducts inside nipple removed	Paget disease recurrence: 4 cases with NAC erosions, 2 crusted lesions, 1 ulcerated NAC
Lohsiriwat, 2013 [162]	European Institute of Oncology, Milan, Italy	2002-2007	Pts with NAC excision because of necrosis to	934 NSM: 40 with NAC removal	Retrospective<br>Study of NAC necrosis	Pts with NSM for breast cancer treatment	NSM contraindications : tumour behind the NAC, nipple retraction,	Exclude if tumour cells present on retroareolar frozen section	Not reported	Various incision locations and mastectomy techniques used	40 (4.2%) NAC necrosis requiring removal

Citation	Study name and location	Years of study*	Topic or comparison	Number of patients	Design	Population	Criteria for NSM	Pathology	Oncologic outcomes	Surgery Details	Nipple viability and necrosis
			those with successful NSM		requiring excision<br>Median follow-up 50 months<br><50 per group; not enough pts with necrosis to analyze subgroups	Exclude if previous chest wall irradiation, bilateral breast cancer, benign disease, prophylactic mastectomy, neoadjuvant therapy	bloody nipple discharge, inflammatory cancer, retroareolar microcalcification	Pts with positive margins on frozen section of retroareolar tissue were excluded		Glandular tissue dissected between gland and subdermal layer leaving thin layer at least 5 mm to preserve subdermal vessels; 5 mm extra-areolar flap and areolar flap recommended to avoid flap necrosis; at areolar edge a separate slice of tissue sent for frozen examination; ELIOT on NAC (n=707) unless NAC perfusion seemed critical or ELIOT machine not available (174 received delayed ELIOT and 53 no ELIOT)	Group without IORT had highest incidence of NAC necrosis; this could be because surgeon withheld IORT due to poor perfusion and not related to IORT use<br>Group with expander had higher necrosis, but selection bias as expanders often because of risk factors (ptosis, large breast, smoker, poor vascularization)
Galimberti, 2018 [163]<br>May overlap with Petit, 2009 [160] which was restricted to primary tumours ≥1 cm outside areola margins	European Institute of Oncology, Milan, Italy	2003-2011	Oncologic outcomes of NSM for invasive or in situ breast cancer	1989<br>IORT to 1342 invasive and 197 in situ<br>No IORT in 369 invasive and 81 in situ	Retrospective<br>Follow-up every 6 months<br>Follow-up at least 5 y; median 94 months	Consecutive women who had NSM for invasive or non-invasive breast cancer; minimum follow-up 5 years<br>Exclude metastatic disease, bilateral synchronous breast cancer,	Exclude if pathological secretion from the nipple, Paget disease, phyllodes tumour, inflammatory or recurrent breast cancer,<br>Allowed: NACT, any TND	Excluded if positive intraoperative retroareolar frozen section, in which case SSM usually performed	102 (5.1%) LR: 11 cases (4%) in in situ group and 91 (5.3%) in invasive group<br>36 (1.8%) NAC recurrence: 9 (3.2%) in situ and 27 (1.6%) invasive groups<br>157 (7.9%) LRR 11 in situ group,	Not reported (assumed to be similar to Petit, 2009 [160]<br><u>Other</u><br>No statistically significant difference in OS or recurrence between pts with IORT to NAC and those without<br>ELIOT (IORT with electrons) to NAC in	66 (3.3%) NAC removed for necrosis: 6 (2.2%) in situ group, 60 (3.5%) invasive group<br>NAC necrosis declined over time: 4.8% in 2003-2005 to 1.4% in 2009-2011

Citation	Study name and location	Years of study*	Topic or comparison	Number of patients	Design	Population	Criteria for NSM	Pathology	Oncologic outcomes	Surgery Details	Nipple viability and necrosis
						other non-breast primary cancer, BRCA mutation carriers without cancer (bilateral prophylactic mastectomy)<br>SLNB or ALND in some cases<br>Immediate reconstruction in all cases: 1706 implant, 290 expander, 15 autologous			146 invasive group<br>199 (10.0%) DM: 2 (0.7%) in situ group and 197 (11.5%) invasive group<br>107 other cancer: 17 (6.1%) in situ and 90 (5.3%) invasive<br>131 (6.6%) deaths: 3 (1.2%) in situ group and 128 (6.5%) invasive group<br>109 breast cancer deaths: 1 (0.4%) in situ and 108 (6.2%) invasive group<br>5-y OS 96.5%: 99.2% in situ and 96.1% invasive group<br>10-y OS 91.2%: 98.8% in situ and 90.0% invasive	1342/1711 pts (78.4%) with invasive cancer and 197/278 (70.9%) with in situ disease; and additional 114 pts (6.7%) with invasive carcinoma received external beam RT<br>IORT use decreased over time and was abandoned in 2011 as they realized flap vascularization was the key to reducing necrosis and intraoperative retroareolar frozen section was a reliable way to ascertain NAC disease	
Vicini, 2021 [164]	European Institute of Oncology, Milan, Italy	2003-2017	Feasibility of NSM after previous breast surgery<br>No comparison of selection criteria or	368 pts, 387 NSM	Median follow-up 54 months from NSM	Previous (primary) surgery was 89.2% quadrantectomy; 2.8% non-oncologic resection; 8% cosmetic	NSM contraindicated for T4 neoplasms, involvement of retroareolar tissue, microcalcifications close to	Not reported	In 117 pts with recurrence at least 6 months after initial quadrantectomy (at median 36 months)	Not reported<br>Various skin incisions	Complete NAC necrosis in 11 cases (2.8%)<br>Partial NAC necrosis in 21 cases (5.4%)

Citation	Study name and location	Years of study*	Topic or comparison	Number of patients	Design	Population	Criteria for NSM	Pathology	Oncologic outcomes	Surgery Details	Nipple viability and necrosis
			surgical procedures			(augmentation, reduction, mastopexy)	subareolar region, malignant nipple discharge, Paget disease		5-y OS 99.1%, 95% CI=93.9-99.9 [1 event]<br>5-y DFS 93.8%, 95% CI=86.5-97.2 [11 events]		
Chirappapha, 2014 [165]	European Institute of Oncology, Milan, Italy	2012-2013	Breast morphology and necrotic complications	113 pts, 124 NSM	Prospective<br>At least 1 month follow-up for all pts	113 carcinoma, 11 prophylactic NSM<br>Exclude if previous RT, neoadjuvant chemotherapy<br>ELIOT delivered to NAC unless prophylactic NSM<br>7 autologous, rest implants ± expander	Primary tumours outside areolar margins and not centrally located<br>Excluded nipple retraction or bloody discharge, retroareolar microcalcifications, inflammatory signs, inflammatory cancer, Paget disease	Thin tissue beneath retroareolar area removed separately for frozen section examination; if positive the NAC was removed and pt excluded	Not reported	Recorded volume of breast removed by measurements of mastectomy specimen<br>Cutaneous incision (96 superolateral, 18 superior circumareolar, 10 other) located above the tumour site, glandular tissue dissected close to dermis and from pectoral fascia; ELIOT delivered unless poor blood perfusion (in which case NAC RT was delayed)<br>Expanders, when used, were inflated ≥3 weeks after NSM	Partial NAC necrosis in 15 NSM (12.1%)<br>Total NAC necrosis in 4 NSM (3.5%)<br>NAC removed in 5 cases (4%)<br>Association between NAC necrosis and volume of breast removed, p=0.04; NAC necrosis 6% when volume <750 cm^3 and 23% when volume >750 cm^3<br>25% NAC necrosis with superior circumareolar or periareolar incisions vs. 13% with other incisions

Citation	Study name and location	Years of study*	Topic or comparison	Number of patients	Design	Population	Criteria for NSM	Pathology	Oncologic outcomes	Surgery Details	Nipple viability and necrosis
Warren Peled, 2012a [166]	University of California, San Francisco, CA, USA	2001-2010	Oncologic safety and complications with TSSM	428 pts, 657 TSSM<br><br>399 pts with cancer<br><br>212 unilateral therapeutic; 187 therapeutic + contralateral prophylactic; 29 bilateral prophylactic	Retrospective 2001-2004; prospective 2005-2010<br><br>Median follow-up 28 months	245 breasts (37.3%) for risk-reduction so only results reported separately for therapeutic reasons meet this review inclusion criteria<br><br>Various autologous and one- or two-stage implants	MRI only if tumour is close to nipple on clinical examination or mammography; exclude if clear tumour involvement<br><br>Excluded clinical evidence of nipple or skin involvement at time of mastectomy (will perform TSSM if initial skin involvement shows good response to NACT)	Do not use frozen section analysis and instead use permanent pathology<br><br>If tumour near or in the nipple skin: repeat excision, resection of involved skin or PMRT; all repeat excisions were negative for cancer<br><br>If invasive cancer in nipple specimen: repeat excision if pt are highly motivated to preserve NAC<br><br>In final pathology, 20 (3%) of nipple tissue specimens had tumour (11 in situ, 9 invasive); 7 repeat excision; 9 NAC removal (8/9 invasive cases), 4 RT<br><br>Re-excision or NAC removal at time of expander exchange or autologous flap revision<br><br>All 16 removed NAC and re-excised nipple tissue specimens were	Outcomes for subgroup of 412 therapeutic cases:<br><br>4 (1%) LR alone<br><br>8 (1.9%) DM alone<br><br>4 (1%) LR + DM<br><br>16 (3.9%) any recurrence<br><br>0 NAC recurrence	Inversion of nipple and complete excision of all nipple tissue at the dermal junction<br><br>Various incisions	Therapeutic and prophylactic not reported separately<br><br><u>Other</u><br><br>Nipple necrosis decreased after periareolar incisions minimized and ceased used of free nipple grafts and NAC-crossing incisions<br><br>1.5% total nipple necrosis, 2% partial nipple necrosis, 11.9% flap necrosis

Citation	Study name and location	Years of study*	Topic or comparison	Number of patients	Design	Population	Criteria for NSM	Pathology	Oncologic outcomes	Surgery Details	Nipple viability and necrosis
								negative for residual tumour			
Wang, 2014 [106]	University of California, San Francisco, CA, USA	2005-2012	TSSM; effect of systemic changes in technique on complications	633 pts, 981 cases including 350 (36%) prophylactic and 626 therapeutic	Prospective collection and retrospective review<br><br>Median follow-up 29 months	Relative contraindications: gigantomastia and grade III ptosis	MRI used if tumour was close to nipple on clinical examination or mammography<br><br>Eligible for TSSM if MRI found no direct tumour involvement of the NAC, even if <1 cm TND<br><br>Allowed pts treated with NACT who met criteria after NACT<br><br>Excluded pts with evidence of nipple or skin involvement at time of mastectomy	On final pathology, nipple specimens in therapeutic cases had 15 (2.4%) in situ carcinoma and 17 (2.7%) invasive carcinoma: 2 in situ and 10 invasive had nipple skin resection; 3 in situ and 5 invasive had PMRT; 10 in situ and 2 invasive (with margins not involved) received no treatment	Oncologic outcomes in therapeutic cases (excluding stage IV):<br><br>5-y cumulative LRR 3% (14 cases): 3.7% stage 0, 0 stage I, 4.5% stage II, 6.9% stage III<br><br>No recurrence in NAC skin in cases that did not have nipple resection<br><br>5-y DM 4.2% (15 cases); 0.8% stage 0, 1.5% stage I, 4.6% stage II, 17.7% stage III<br><br>All patients (including 7 stage IV cases):<br><br>Overall 5-y survival 93% (19 deaths); 97% stage 0, 98% stage I, 96% stage II, 74% stage III, 33% stage IV	Significant changes in technique over time, stopped using free nipple grafts and NAC-crossing incisions, adopted TSSM as standard procedure in 2005<br><br>After 2005 any periareolar incisions incorporated <1/3 NAC circumference<br><br>Incisions 58% inframammary, 30% superior periareolar, 3% radial, 4% lateral<br><br>TSSM with inversion of nipple and excision of all nipple tissue at the dermal junction<br><br>Prophylactic antibiotics switched to trimethoprim-sulfamethoxazole instead of cephalexin in 2009	Prophylactic and therapeutic not reported separately<br><br>Complications decreased over time; by 2012, 3.5% superficial nipple necrosis, 1% complete nipple necrosis; 3.0% minor skin flap; necrosis, 4.4% major skin flap; necrosis<br><br>Discontinued immediate implants (0.3%) by 2006 in favour of gradual expansion (89%) to minimize NAC and skin-flap necrosis; 10.2% autologous

Citation	Study name and location	Years of study*	Topic or comparison	Number of patients	Design	Population	Criteria for NSM	Pathology	Oncologic outcomes	Surgery Details	Nipple viability and necrosis
											Other<br><br>When PMRT used, increased waiting time to 6 months after RT before expander-implant exchange
Amara, 2015 [167]	University of California, San Francisco, CA, USA	2005-2013	Strategies for managing nipple involvement; changes in outcomes over time for TSSM	748 pts, 1173 breasts; 440 (38%) prophylactic, 733 (62%) therapeutic	Retrospective review of prospectively collected database<br><br>31.3 months follow-up	Note: PMRT was determined by primary indications (tumour size or LN status and not only NAC involvement)	TSSM for all pts without clinical involvement of the NAC or skin at mastectomy and no significant ptosis or macromastia; also pts with skin involvement and good response to NACT<br><br>MRI initially used to assess NAC involvement, but no longer used routinely unless for other reasons such as NACT response	NAC had positive margin or involvement of nipple tissue in 32 breasts (2.7% of total, 4.7% of therapeutic, 0% prophylactic): 18 invasive and 14 in situ; treated by repeat incision (11 cases, 34%), RT to NAC as part of PMRT (5 cases, 16%), NAC removal (8 cases, 25%), or no further treatment (8 cases, 25%); complete NAC excision decreased over time<br><br>Of re-excisions, 5 had only scar/fibrous tissue, 4 had benign breast tissue, and	LRR 6.2% [unclear if this is therapeutic or all TSSM]<br><br>In pts with initial NAC involvement:<br><br>1 LR and 1 LR+DM<br><br>No recurrence in preserved NAC skin; no recurrence in DCIS<br><br>In invasive cancer with initially positive NAC pathology: 1/7 re-excisions had LR (not in nipple), 3/3 with RT had DM, 0/5 with NAC removal had recurrence, 1/3 without	Preferred incision was IMF (53%), superior areolar (38%)<br><br>Removal of nipple tissue through inversion of nipple and excision of nipple tissue at the dermal junction<br><br>Subareolar margin deep to the NAC marked with a suture on mastectomy specimen and closely examined<br><br>Nipple completely cored out and new nipple margin sent as a separate specimen	Not reported

Citation	Study name and location	Years of study*	Topic or comparison	Number of patients	Design	Population	Criteria for NSM	Pathology	Oncologic outcomes	Surgery Details	Nipple viability and necrosis
								2 are scheduled (not yet excised)	treatment had LR + DM		
Warren Peled, 2016 [168]	University of California, San Francisco, CA, USA	2005-2013	TSSM outcomes in LABC	139 pts with LABC (stage IIb-III)	Retrospective review of prospectively collected data<br>Mean follow-up 41 months	Stage IIb (25 pts) Stage III cancer (114 pts); most received neoadjuvant (77%) or adjuvant chemotherapy (20%)<br>Exclude if significant ptosis, large breast size<br>Immediate breast reconstruction (92% expander-implant, 8% autologous)	Exclude if clinical involvement of NAC in examination or imaging<br>TSSM offered to pts with initial skin involvement but no skin involvement after NACT	Nipple tissue evaluated during final pathology	LR as in 7 pts (5%), DM in 21 pts (15.1%), LR + DM in 3 pts (2.2%)<br>All LR was in pts with residual disease at mastectomy (not complete response to NACT), and all eventually developed DM<br>No recurrence in preserved NAC skin<br>5-y EFS 70%	Removal of nipple tissue through inversion of nipple and excision of nipple tissue at the dermal junction<br>Preferred incision was IMF or superior areolar/mastopexy<br>Other<br>PMRT in 63%; given before expander-implant exchange without deflation of expander	2 (1.4%) NAC necrosis<br>5 (3.6%) mastectomy skin flap necrosis
Holland, 2023 [169]	University of California, San Francisco, CA, USA<br>Single institution	2015-2018	Impact of incision location on NAC complications in prepectoral reconstruction	108 pts, 181 reconstructions (91 prophylactic); 113 (62%) superior periareolar incisions vs. 68 (38%) IMF incisions	Retrospective review<br>Multivariable binary logistic regression for outcome of any nipple necrosis	Immediate 2-stage prepectoral breast reconstruction after NSM	Pts with immediate two-stage prepectoral reconstruction after NSM<br>Eligibility of NSM based on accepted oncologic criteria, breast size, degree of ptosis	All retroareolar breast tissue removed in mastectomy and analyzed on permanent section	Not reported	Superior periareolar incision in 62% and IMF incision in 38%<br>NAC and skin flap viability assessed by clinical examination without indocyanine green angiography or other adjuvant assessments<br>Skin expanders filled at surgeon discretion to prevent skin wrinkling but not to place tension on	Without adjustment: any NAC complications by incision location: 25% periareolar vs. 7.4% IMF, p=0.003; nipple necrosis requiring debridement 9.7% vs. 1.5%, p=0.033<br>Other

Citation	Study name and location	Years of study*	Topic or comparison	Number of patients	Design	Population	Criteria for NSM	Pathology	Oncologic outcomes	Surgery Details	Nipple viability and necrosis
										closure or pressure on skin	In multivariable regression, hypertension (OR=4.1, p=0.004), smoking (OR=9.6, p=0.029), and a periareolar incision (OR=3.6, p=0.018) were independently associated with an increased odds of any nipple necrosis
Frey, 2019 [170]	New York University Langone Health, New York, NY, USA	2006-2017	Outcomes and risk factors including TND in NSM	312 pts, 496 NSM (all therapeutic) ; 128 pts with unilateral cancer and 184 pts with bilateral cancer	Retrospective<br>Binary logistic regression<br>Average follow-up 48.25 months	Pts with therapeutic NSM (biopsy proven or strongly suggestive imaging); excluded pts with prophylactic contralateral mastectomy<br>Immediate reconstruction	NSM candidate if therapeutic mastectomy without clinical evidence of NAC involvement<br>Relative contraindications (presence of multiple factors may exclude NSM): NACT, active smoking, severe macromastia or breast ptosis, significant chest/NAC asymmetry	Subareolar tissue sent for frozen (n=362) and permanent section (n=496) analysis; NAC removed if either was positive; positive rates 6.9% in both	LR in 8 NSM, 1.6% per NSM or 2.6% per pt<br>Two of the recurrences were in the NAC<br>Regional recurrence in 3 NSM, 0.6% per NSM or 1.0% per pt<br>LRR in 10 NSM in 9 pts; 2.0% per NSM or 2.9% per pt<br>DM in 4 pts (1.3%)	Not reported	Not reported<br><u>Other</u><br>MRI in only 5 pts with recurrence so too few to do subgroup analysis

Citation	Study name and location	Years of study*	Topic or comparison	Number of patients	Design	Population	Criteria for NSM	Pathology	Oncologic outcomes	Surgery Details	Nipple viability and necrosis
							TND assessed by MRI in 171 NSM				
Salibian, 2021 [171]	New York University Langone Health, New York, NY, USA	2007-2019	Incision choice in NSM and outcomes	163 pts, 279 NSM; 229 breasts with cancer	Retrospective	All pts with NSM and immediate reconstruction with microvascular tissue transfer and ≥1 y follow-up<br><br>Excluded delayed and delayed-immediate reconstruction	Excluded nipple-areola complex involvement, inflammatory cancer; TND ≤1 cm later in study<br><br>Relative contraindications: smoking, NACT, prior RT, poor breast skin quality, severe NAC/chest wall asymmetry, grade III ptosis and severe macromastia<br><br>Internal mammary vessels were preferred recipient vessels for reconstruction		Not reported	NSM using sharp dissection with minimal electrocautery at the level of the breast capsule; flaps assessed based on skin-edge bleeding, flap thickness, visible dermis; indocyanine green angiography was precluded by use of epinephrine-containing infiltration before mastectomy<br><br>Incision type was based on tumour size/location, breast size and skin excess, previous scars, lymph node status, and pt desires; periareolar incisions avoided	Full NAC necrosis in 11 cases (3.9%)<br><br>Partial NAC necrosis in 19 cases (6.8%)<br><br><u>Other</u><br><br>Subgroup analysis has <25 events so not extracted<br><br>Higher rates of major ischemic complications with IMF incisions (25%) and inverted-T incisions (36.1%) than vertical (5.8%) or lateral radial (7.8%) incisions (101). In multivariate analysis inframammary (OR=4.382) and inverted-T incisions (OR=3.952) were independently associated with increased risk

Citation	Study name and location	Years of study*	Topic or comparison	Number of patients	Design	Population	Criteria for NSM	Pathology	Oncologic outcomes	Surgery Details	Nipple viability and necrosis
Boyd, 2022 [172]	New York University Langone Health, New York, NY, USA	Review of data in 2021	Recurrence in therapeutic NSM with median 10 y follow-up	120 pts, 126 NSM	Retrospective<br>Median 10 y follow-up, average 124.4 months	Pts with therapeutic NSM (biopsy proven or strongly suggestive imaging); excluded pts with prophylactic contralateral mastectomy	Excluded nipple-areola complex involvement	Positive frozen subareolar biopsy 7.3% (6/82 NSM) and permanent subareolar pathology 9.5% (12/126); NAC removed if positive; these pts were kept in the recurrence analysis	4 recurrences, 3.17% per NSM or 3.33% per pt<br>2 LR, 1.59% per NSM or 1.67% per pt<br>2 regional recurrence, 1.59% per NSM or 1.67% per pt<br>3 LRR, 2.4% per NSM or 2.5% per pt<br>2 DM (1.7%)	Not reported	Not reported<br>Other<br>Recurrences too few so further data not extracted
Radovanovic, 2010 [173]	Oncology Institute of Vojvodina, Serbia	2004-2008	Early complications after NSM and immediate silicone implants	205 pts, 214 NSM	Prospective	Consecutive pts with breast cancer and NSM and immediate reconstruction with fixed volume silicone implants placed under the pectoralis major and serratus anterior muscles<br>Unilateral NSM in 196 pts, bilateral 9 pts	Preoperative diagnosis with physical examination, ultrasound, mammography, fine needle aspiration or core biopsy<br>Contraindications were inflammatory, extensive skin involvement, Paget disease	Frozen section analysis of subareolar tissue; if cancer cells the NAC was removed<br>NAC removed in 4 cases	Not reported	Lateral incision usually extended to upper outer quadrant for axillary access; other incisions if previous excisional biopsy or BCS	7.5% any skin flap or NAC necrosis:<br>1% NAC necrosis; major skin flap necrosis 4%, minor skin necrosis 3%
Folli, 2012 [174]	Italy	2006-2010	Use of hydrodissection	115 pts and NSM; 101 cancer and 14 risk-reduction		Retrospective in Cohort 1 and prospective in Cohort 2	Contraindications: carcinoma infiltration skin or NAC, inflammatory, pathologic	A 3-5 mm thick layer of tissue removed from retroareolar area and submitted for margin evaluation	1 LR (0.9%)<br>No recurrence in NAC	Italic S incision from lateral edge of areola to external equatorial line<br>Cohort 1 (until June 2009; 74 pts): NAC	No cases of complete necrosis requiring NAC removal

Citation	Study name and location	Years of study*	Topic or comparison	Number of patients	Design	Population	Criteria for NSM	Pathology	Oncologic outcomes	Surgery Details	Nipple viability and necrosis
						Median follow-up 19 months	discharge from nipple<br>Relative contraindications: previous RT<, smoking, diabetes, recent per/subareolar surgery, large breasts NAC to IMF > 8 cm	on frozen section; if neoplastic tissue detected the NAC was removed and converted to SSM<br>20 cases (17.4%) converted to SSM<br>Removed NAC were examined for breast glandular tissue in permanent sections; found in 12/13 (92%) in cohort 1 and 1/7 (14%) in cohort 2		dissected by sharp isolation, coring the nipple to remove all glandular tissue<br>Cohort 2 (July 2009 - 2010; 41 pts): as cohort 1 but preceded by hydrodissection of the areola by injection of saline/adrenaline into the deep subareolar dermis to obtain complete detachment of skin, then isolation of areola by dissecting the swollen plane with scissors and the nipple cored	
Lee, 2013 [175]	Samsung Medical Center, Sungkyunkwan University School of Medicine, Seoul, South Korea	2009-2012	Risk factors of mastectomy flap complications	125 pts, 130 NSM	Retrospective<br>Prospectively collected database<br>Multivariate analysis but too few events and did not report for NAC	Lateral incisions for NSM, immediate reconstruction; exclude prior partial mastectomy or RT<br>2 prophylactic, 47 stage 0, 45 stage I, 34 stage II, 2 stage III<br>70 autologous and 60 two-stage implants	Inclusion based on clinical, radiological, and pathological evaluations	Intraoperative frozen section analysis on retroareolar duct regions; NAC removed when neoplasm found	Not reported	Lateral incisions<br>Used electrocautery	15 nipple complications: total nipple necrosis in 5 cases (3.8%), partial loss in 10 cases (7.7%)<br>Necrosis rates decreased over time

Citation	Study name and location	Years of study*	Topic or comparison	Number of patients	Design	Population	Criteria for NSM	Pathology	Oncologic outcomes	Surgery Details	Nipple viability and necrosis
Huston, 2015 [176]	New York Presbyterian Hospital-Weill Cornell Medical Center, New York, NY, USA	2006-2012	Impact of scarring from previous BCS on NAC viability	318 NSM: 122 with previous lumpectomy and 196 without	Retrospective<br>Prospectively collected database<br>Follow-up at 2 weeks, 1 month, 2 months, 6 months, then yearly	Breast cancer (51% multifocal lesions), NSM via IMF incision<br>Single or 2-stage implant reconstruction	Candidate for SSM with no nipple involvement, tumour >2 cm from nipple, could attend frequent follow-up to examine NAC	Frozen section of deep dermis of nipple; if it had malignant or atypical cells then NSM was converted to ASM or SSM	In pts with prior lumpectomy:<br>LR 3 cases (2.5%)<br>DM 2 cases (1.6%)	Flaps infiltrated with local anesthetic, incision ≈12 cm long along IMF, subdermal dissection with sharp scissors leaving flap 3-5 mm thick, marking suture placed on breast gland immediately deep to NAC for the pathologist, NAC inverted and sharply cleaned of glandular tissue, additional specimen scraped from the deep dermis of the nipple for frozen section, gland resected off pectoralis muscle using electrocautery	NAC ischemia (epidermolysis or necrosis) in 65/318 NSM (20.4%)<br>Pts with prior lumpectomy: ischemia in 30/122 (24.6%), including 20 NSM (16.4%) epidermolysis and 10 (8.2%) necrosis; 2 (1.6%) required operative debridement; 7 NSM (5.7%) had NAC depigmentatio n<br>Without prior lumpectomy: ischemia 17.9%
Ahn, 2018 [177]	Yonsei University College of Medicine, Korea	2010-2016	Ischemia and necrosis after NSM	207 pts, 220 NSM (4 prophylactic)	Retrospective<br>Multivariate analysis	NSM and immediate reconstruction; implants in the subpectoral plane with ADM sling or autologous flaps	Breast cancer or phyllodes tumour (1 pt)	Intraoperative frozen section for sub-NAC tissue	Not reported	Glandular tissue removed along superficial mammary fascia and pectoral fascia, skin flap thickness 3-5 mm, various skin incisions used<br><u>Other</u><br>Authors suggest skin tension may interfere with blood flow and be the cause of	NAC ischemia (clinical ischemic colour change in any portion of NAC) in 141 cases (64.1%)<br>NAC necrosis (full thickness) requiring surgical reoperation (debridement

Citation	Study name and location	Years of study*	Topic or comparison	Number of patients	Design	Population	Criteria for NSM	Pathology	Oncologic outcomes	Surgery Details	Nipple viability and necrosis
										higher rates of necrosis with DTI and autologous reconstruction<br><br>Second intercostal artery was always sacrificed; it is the principal perforator for the NAC and may have been reason for very high rates of ischemia and necrosis<br><br>52.3% of incisions were periareolar plus radial	and repair) in 69 cases (31.3%)<br><br>Grade 4/5 ischemia 24.1%; necrosis in this group is 19.5% of all pts<br><br>Ptosis, periareolar incision, and reconstruction other than 2-stage (expander) implant were predictors of NAC necrosis
Pek, 2018 [178]	Singapore General Hospital	2005-2015	Aesthetic outcomes and NAC necrosis in Asian pts	133 pts, 142 NSM	Mean follow-up 37 months	NSM for cancer (85.9% of NSM) or risk reduction (14.1%)	Intraoperative frozen section of retroareolar tissue; NAC removed if involved<br><br>80% autologous reconstruction (115 NAC); 2 stage expander/ implant in subpectoral plane; DTI		LR in 5 NSM (3.52%)	Previous biopsy or BCS scars often incorporated into new incision; mastectomy skin flaps assessed for thickness and NAC viability; if not excessively thin and the NAC was healthy there was immediate closure; skin paddle from flap incorporated if excessive tension was anticipated otherwise used primary closure with release of sutures if	NAC necrosis in 17 breasts (12.0%): total NAC necrosis in 4 breasts (2.8%) and partial in 13 (9.2%)

Citation	Study name and location	Years of study*	Topic or comparison	Number of patients	Design	Population	Criteria for NSM	Pathology	Oncologic outcomes	Surgery Details	Nipple viability and necrosis
										signs of NAC necrosis developed<br>Delayed primary closure if NAC viability threatened and banked a skin paddle<br>The banked paddle was used in case of partial or total NAC necrosis	
Radovanovic, 2018 [179]	Oncology Institute of Vojvodina, Serbia	2004-2012	Surgical complications after NSM	435 pts, 441NSM	Retrospective<br>Follow-up weekly if complications, otherwise every 3 months the first year then 6 months for 2 years, then yearly<br>Mean follow-up 79 months	Pts with breast cancer, NSM as initial procedure or after BCS, implant reconstruction with contoured profile fixed-volume gel-filled prostheses	Not reported	Subareolar tissue excised and sent for frozen analysis<br>NAC excised in 24 cases (5.4%) due to cancer cells in subareolar tissue by frozen section or final analysis; NAC preserved if no malignant cells	LR in 32 pts (7.3%); 2 recurrences in NAC<br>DM 68 pts (15.6%)<br>Deaths 53 pts (12.2%)	Lateral incision in 81.2% extending to upper outer quadrant allowing axillary access; other incisions if previous excisional biopsy or BCS to incorporate previous incision<br>Breast tissue and fat entirely removed except under NAC; subareolar tissue removed for frozen analysis;	NAC necrosis in 1 cases (0.2%);
Pallara, 2019 [180]	San Giovanni-Addolorata Hospital and Campus Bio-Medico University of Rome, Rome, Italy	2013-2015	Expander-implant vs. DTI	162 pts: 56 expander, 106 DTI	Retrospective<br>Multivariate analysis<br>Median follow-up 35 months	Reconstruction in pts with breast cancer; either submuscular expander-implant or DTI	Main indicator was multicentric tumours without NAC involvement	Not reported<br>NAC removed due to positive retroareolar margins on definitive histological exam in 3 pts	1 recurrence in each group (1.79% expander/implant and 0.94% DTI	Radial incision in 90% of NSM (but discussion says IMF in most cases) skin flaps dissected with low-voltage electric scalpel, keeping ≈3 mm thickness, hydrodissection of NAC with saline solution to aid	8.0% partial NAC necrosis: expander 2 pts (3.6%), DTI 11 pts (10.4%)<br>4.3% total NAC necrosis: expander 3 pts (5.4%), DTI 4 pts (3.8%)

Citation	Study name and location	Years of study*	Topic or comparison	Number of patients	Design	Population	Criteria for NSM	Pathology	Oncologic outcomes	Surgery Details	Nipple viability and necrosis
										retroareolar dissection; viability of mastectomy skin flaps checked and nonviable flaps excised if no skin margin bleeding; expander or DTI determined by defect size	
Park, 2020 [181]	Gangnam Severance Hospital, Yonsei University College of Medicine, Seoul, South Korea	2009-2018	Complications according to incision type	275 pts, 290 NSM<br>61 periareolar, 53 radial, 176 IMF	Retrospective<br>Follow-up recommended every 6 months<br>Median follow-up 67 months, 54 months, 34.5 months<br>Multivariate analysis for risk factors of NAC necrosis<br>81% of cases by one surgeon	NSM and immediate reconstruction<br>Mostly invasive breast cancer<br>Exclude if previous augmentation<br>SLNB or ALND depending on nodal status<br>Implants inserted in subpectoral plane (89% DTI, 6.6% expander) and ADM used; or autologous (4.5%)	NAC invasion not suspected<br>Invasive or in situ cancer (283 NSM), phyllodes tumour (2 NSM), prophylaxis (5 NSM)<br>TND (to nipple base) by MRI except if NAC; used 1 cm increments in multivariate analysis<br>For breast weight, multivariate analysis is risk per 1 g increase in breast weight	Subareolar margin analyzed by frozen section; if cancer invasion found the NAC was resected then the pt excluded	LR by incision type: 2 (3.3%) periareolar, 1 (1.9%) radial, 6 (3.4%) IMF<br>2-y local RFS 98.4%, 98.1%, 97.6%, differences not significant<br>Overall complications 42.6% periareolar, 35.8% radial, 18.8% IMF incision	Incisions either periareolar (part of either upper or lower side of areola plus radial incision), radial (lateral side of NAC and extending obliquely to axilla), IMF (lower outer arc along crease of IMF)<br>Skin flap along superficial mammary fascia using electrocautery with thickness ≈7-15 mm	45 (15.5%) NSM with nipple necrosis (described as NAC necrosis or ischemia in abstract): 19 (31.1%) periareolar, 9 (17.0%) radial, 17 (9.7%) IMF<br>25 (8.6%) NSM with complete nipple necrosis: 13 (21.3%) periareolar, 6 (11.3%) radial, 6 (3.4%) IMF<br>Risk factors: periareolar incision (vs. IMF; OR=3.628, 95% CI=1.596-8.250, p=0.002); TND (OR=0.712, 95% CI=0.546-0.927, p=0.012);

Citation	Study name and location	Years of study*	Topic or comparison	Number of patients	Design	Population	Criteria for NSM	Pathology	Oncologic outcomes	Surgery Details	Nipple viability and necrosis
											breast weight (OR=1.002, 95% CI=1.000-1.004, p=0.014)
Seki, 2020 [182]	Saitama Medical Center, Saitama, Japan	2013-2019	Outcomes of periareolar incisions	181 pts; 31 IMF and 150 periareolar incision	Retrospective<br>Median follow-up 18.3 months IMF, 79.0 months periareolar	Primary operable breast cancer and NSM without intraoperative NAC resection<br>Submuscular expanders or implants, or autologous reconstruction (0% IMF, 13.3% periareolar)	Imaging including mammography, ultrasound, MRI to identify NSM candidates<br>Excluded if intraoperative NAC resection	NAC involvement by intraoperative sub-nipple biopsy; if involvement was suspected the NAC was resected<br>Surgical margins on side of skin and chest wall and under NAC by permanent pathology	RFS, HR=0.528, 95% CI=0.054-5.127, p=0.860	Incision based on pt preference, breast size, radiological findings<br>For IMF, incision ≈10 cm along IMF; for periareolar, incision was along lower areola<br>For periareolar: incision in lower half of areolar circumference, Lap protector attached to protect the wound, breast tissue dissected from subcutaneous tissue in all directions, mammary gland lifted from pectoralis major fascia and adhesion under pectoralis major muscle dissected; deflated expander folder and inserted<br>IMF incision details not reported	Complete nipple necrosis (spanning entire layer from epidermis to dermis of the nipple) in 0 pts IMF incision and 5 pts (3.3%) periareolar incision<br>Epidermal nipple necrosis (only in epidermis of nipple) in 5 pts (16.1%) and 24 pts (16.0%)
Najmiddinov, 2022 [183]	Seoul National University Bundang Hospital,	2014-2021	Conventional (c-NSM) or modified (m-NSM)	516 pts, 580 NSM: 143 c-NSM, 437 m-NSM	Retrospective<br>Average follow-up	Exclude stage IV, delayed reconstruction, PMRT	Excluded NAC or skin involvement, inflammatory	Not reported	10 recurrence (2.2%; 4 pts or 3% c-NSM, 6 pts or 1.8% m-NSM)	Lateral radial, IMF, and inverted T incisions most	4 (0.9%) partial NAC necrosis: 3 (2.3%) c-NSM,

Citation	Study name and location	Years of study*	Topic or comparison	Number of patients	Design	Population	Criteria for NSM	Pathology	Oncologic outcomes	Surgery Details	Nipple viability and necrosis
	Seongnam, South Korea		Modified preserves the anterior lamellar fat layer		41.92 and 31.98 months		cancer, Paget disease		LR 3 pts (0.6%; 2 pts or 1.5% c-NSM, 1 pt or 0.3% m-NSM)<br><br>DM 2 pts (0.4%; 1 pt or 0.8%, and 1 pt 0.3%)	common, periareolar not used<br><br>c-NSM: After incision, skin flaps elevated with the Bovie coagulator on the superficial fascial plane anteriorly; skin flaps developed along superficial layer of superficial fascia resulting in an even flap; plane between pectoralis major fascia and pectoralis major muscle was posterior plane of dissection<br><br>m-NSM: skin incision as c-NSM; main difference was anterior plane of dissection was performed along with the breast capsule (anterior capsule of corpus mammae), breast parenchyma separated from subcutaneous fat layer by the breast capsule to give the anatomic dissection plane which maximizes preservation of anterior lamellar fat layer and increases mastectomy flap thickness; when	1 (0.3%) m-NSM, p=0.074<br><br>1 (0.2%) total NAC necrosis: 1 (0.8%) c-NSM and 0 m-NSM, p=0.287<br><br>Wound healing complications (p=0.023), reconstruction failure (p=0.005) and implant rippling (p=0.06) higher in c-NSM<br><br>Panel assessment (blinded to treatment group) of aesthetics: 2.38±0.95 in c-NSM and 3.14±0.61 in m-NSM, p<0.001; scores 1-4 with 4 being excellent<br><br>Breast-Q reconstruction module (122 pts): m-NSM had improved QoL

Citation	Study name and location	Years of study*	Topic or comparison	Number of patients	Design	Population	Criteria for NSM	Pathology	Oncologic outcomes	Surgery Details	Nipple viability and necrosis
										tumour is close to the breast capsule dissection above the tumour area is along the superficial layer of superficial fascia as in c-NSM for oncologic safety; posterior dissection plane same as c-NSM<br><br>In both m-NSM and c-NSM sharp dissection was performed and electrocautery limited to hemostasis to prevent thermal damage to the flap<br><br>Pts evaluated intraoperatively with indocyanine green angiography for perfusion quality<br><br>Mean flap thickness by CT (n=37, n=41) after at least 12 months: 6.32±1.15 mm c-NSM, 8.48±1.81 mm m-NSM, p=0.02	(psychosocial, sexual)
Lai, 2023 [184]	Changhua Christian Hospital, Central Taiwan	2011-2021	Risk factors for NAC ischemia necrosis	441 NSM with 369 reconstructions	Retrospective<br><br>Multivariate logistics	Primary operable breast cancer, excluded if no information on skin excision or NAC ischemia necrosis status	Not reported<br><br>Ischemia necrosis grade 0 (normal), grade 1 (transient ischemia injury, <25% nipple volume loss after recovery), grade 2 (loss of 25-75%), grade 3	Not reported	Not reported	Skin incisions: 83 (18.9%) upper outer incision (radial incision), 107 (24.3%) peri-areolar-related incision (with or without axillary incision), 243 (55.2%) single axillary incision (endoscopic or robotic assisted),	41 NSM (9.3%) had NAC ischemia/ necrosis events (defined as grade 2-3)<br><br>6.6% (29 NAC) grade 1, 8.4% (37 NAC) grade

Citation	Study name and location	Years of study*	Topic or comparison	Number of patients	Design	Population	Criteria for NSM	Pathology	Oncologic outcomes	Surgery Details	Nipple viability and necrosis
							(loss of >75-100% of volume resulting in surgical excision or loss of NAC)			and 7 (1.6%) infra-mammary + axillary incisions	2, 0.9% (4 NAC) grade 3<br>In multivariate analysis, periareolar-related incision (compared with upper outer radial) had odd ratio=5.33 (p<0.001) of NAC ischemia necrosis; larger breast (mastectomy specimen >450 g also risk factor (OR=4.6, p=0.03)
Cadili, 2023 [185]	Providence Breast Centre, Providence Health Care, University of British Columbia	2012-2018	Nipple margin assessment in NSM	NSM 337 pts, including 242 for breast cancer	Retrospective<br>Median follow-up 33.7 months	72% for cancer	Not reported	Nipple margin assessments in 296/337 (87.8%) of NSM; either shave margin under NAC or coring of nipple<br>Surgical nipple margin assessment in 222/242 pts with cancer (91.7%)<br>Of 10 pts with positive nipple margin, 7 had NAC excised, 3 (all with DCIS) had observation; none had LR	Positive margins in 10 pts (3.4% of all NSM or 4.1% of NSM in pts with cancer)<br>15 recurrences in 222 pts with cancer who had margin assessment: 4 local to skin (not NAC), 4 regional, 7 distant; none of these had positive margins on mastectomy specimens	Not reported	Not reported

Citation	Study name and location	Years of study*	Topic or comparison	Number of patients	Design	Population	Criteria for NSM	Pathology	Oncologic outcomes	Surgery Details	Nipple viability and necrosis
Cavalcante, 2023 [186]	Fortaleza General Hospital, Fortaleza, Ceará, Brazil	2015-2022	Inframmamary vs. periareolar incision for NSM	152 pts, 180 NSM; 104 IMF, 76 periareolar	Retrospective<br>STROBE criteria<br>Multivariate analysis	Early-stage breast cancer or risk-reducing mastectomy and immediate reconstruction with IMF or periareolar incisions<br>ADM not used<br>Mastectomy weight mean 246.8 g vs. 312.7 g; BMI normal (<25 kg/m^2) in 70.2% vs. 55.6%<br>Therapeutic 43.3% vs. 55.3%<br>Direct to implant 50% vs. 31.6%<br>Prepectoral 81.7% vs. 96.1%	Not reported	Not reported	Not reported	IMF incision 6-8 cm in length following natural lower outline of breast but not exceeding the anterior axillary line; periareolar generally in lower portion between 3-0 o'clock or upper between 0-12 o'clock (with or without lateralization)<br>Incision type by surgeon's preference, clinical criteria (breast volume and ptosis), pt preference<br>NSM by international guidelines: electric scalpel following anatomic plane (superficial fascia of breast); no minimum flap thickness used; if axilla management needed then IMF fold group had separate incision in axilla while periareolar group used same excision	Complications 16 (15.3%) vs. 27 (35%), p=0.0002, OR=0.33, 95% CI=0.14-0.79<br>NAC necrosis 9 (8.5%) vs. 17 (22.4%), p=0.002, OR=0.33, 95% CI=0.14-0.79; adjusted OR=0.34, 95% CI=0.13-0.88, p=0.025<br>Too few other complications to do multivariate analysis
Moo, 2023 [187]	Memorial Sloan Kettering Cancer Center (MSKCC), New York,	2018-2020	Skin-flap necrosis after NSM<br>Quality improvement initiative to	299 pts with 515 NSM	Prospective<br>15 breast surgeons, 10 plastic surgeons	54.8% prophylactic, 45.2% therapeutic<br>85.4% tissue expander,	Not reported	Not reported	Not reported	Intraoperative variables are in data table<br>Data presentation for univariable and multivariable analysis	Necrosis, per pt basis: 71/299 (23.7%); necrosis occurred in 11% of those

Citation	Study name and location	Years of study*	Topic or comparison	Number of patients	Design	Population	Criteria for NSM	Pathology	Oncologic outcomes	Surgery Details	Nipple viability and necrosis
	New York, USA		identify modifiable risk factors for skin-flap necrosis after NSM		Necrosis categories by SKIN score<br>Multivariable logistic regression with model chosen by backward selection;	5.22% direct implant, 8.3% autologous flap				of variables associated with nipple and skin-flap necrosis after NSM is confusing<br>Results for most variables that were significant in univariable analysis were not reported after multivariable regression; those reported are often inconsistent with univariable results but not commented on by authors<br>Necrosis by incision type:<br>•12.1% lateral IMF, OR=0.41, aOR=0.35<br>•19.0% central IMF, OR=0.64, aOR=0.54<br>•29.8% lateral radial (OR=1.0 as reference)<br>•37.3% inferior periareolar/lateral extension, OR=1.25, aOR=1.24<br>•41.2% superior periareolar/lateral extension, OR=1.38, aOR=1.59<br>•61.5% inferior radial, OR=2.07, [aOR=0.56 but only 8 events so unreliable or error	with hypertension and 2.6% without (p=0.006)<br>Mastectomy skin flap necrosis by 2 weeks: 23.3% (120/515), including 45.8% (55/120) with nipple necrosis only, 27 superficial (SKIN category B), 73 partial (C), 20 full-thickness (D)<br>Higher necrosis with periareolar incision (see surgery details column), specimen weight >400 g, fill volume >200 mL, subpectoral expander, surgeon with less experience

Citation	Study name and location	Years of study*	Topic or comparison	Number of patients	Design	Population	Criteria for NSM	Pathology	Oncologic outcomes	Surgery Details	Nipple viability and necrosis
										in calculation for aOR]	
Serio, 2023 [188]	The Breast Center of the San Giovanni-Addolorata Hospital, Rome, Italy	2016-2021	NSM for cancer, omitting intraoperative examination of retroareolar margins (IERM)	162 pts	Retrospective<br><br>Median 46 months follow-up	NSM offered except if clinical and/or radiological NAC involvement, pathological nipple discharge, Paget disease of the nipple, breast cancer with skin involvement, inflammatory breast cancer, or severe comorbidities<br><br>Exclude if surgical delay (autonomization of the NAC)<br><br>NACT allowed if major or complete response allowing safe resection	Preoperative MRI to measure tumour NAC distance (TND)<br><br>15% of pts had TND <10 mm and 25% of these had close or very close margins, suggesting increased risk but not exclusion of NSM	Retroareolar margin marked with single stay suture and analyzed; margins classified as positive (ink on tumour), negative (no ink on tumour), <1 mm (very close), 1-2 mm (close), >2 mm	17 cases close or very close and 5 recommended for NAC excision and no residual cancer found<br><br>LR at median 46 months follow-up: 5 pts (3%), including 1 (0.6%) at NAC<br><br>DM 10 pts (6%)<br><br>Close or very close margin was not associated with LR or DM; but table suggests lack of statistical significance is due to low number of events (LRR OR=4.46, 95% CI=0.384-52.067, p=0.28; DM OR=3.8, 95% CI=0.640-22.459, p=0.12)<br><br>Authors suggest policy of not conducting intraoperative pathology has advantages including avoiding false	Skin flaps dissected at Coopers ligament (usually with min 3-5 mm thickness) using radiofrequency device to cut and coagulate soft tissue;	5 pts had NAC removed due to nipple necrosis

Citation	Study name and location	Years of study*	Topic or comparison	Number of patients	Design	Population	Criteria for NSM	Pathology	Oncologic outcomes	Surgery Details	Nipple viability and necrosis
									positives and second incision for NAC resection; this assumes final pathology analysis will occur		
Black, 2024 [189]	Weill Cornell Medical College, New York, NY.	Not stated	Impact of reconstruction on sensation	192 pts: 106 autologous via neurotized DIEP, 86 2-stage implants (64 prepectoral)	Single surgeon, single institution, retrospective, breast surgeons and 1 plastic surgeon	NSM with immediate reconstruction<br><br>2-stage implants with expander exchange after 3 months (no RT) or after 6 months (with RT); or use of DIEP flaps<br><br>Autologous group had more comorbidities (higher BMI, hypertension, diabetes) but better restoration of sensation; the relative contribution of type of implant and neurotization cannot be determined	Not reported	Not reported	Not reported	Patients with DIEP flaps had neurotization using donor and recipient nerves: donor nerves were a sensory branch of T10, T11, or T12 thoracoabdominal nerves within the DIEP flap, recipient nerve was anterior cutaneous branch of T3 intercostal nerve near the internal mammary recipient vessels; Avance nerve graft was used for nerve coaptation<br><br>Sensory testing using pressure-specified sensory device that measured 1-point static cutaneous sensation thresholds (0-100 g/mm^2) at 9 regions per breast (inner and outer superior, medial, inferior, lateral; and NAC)	Preoperative threshold 14.1 g/mm^2 for NAC, 16.5 to 20.0 for other sites<br><br>After mastectomy: sensation greatly reduced, most strongly at NAC and surrounding inner regions (threshold for implants 72.3-83.3 g/mm^2 and 64.1 -73.2 for autologous) at <1 year<br><br>Long term (>4 years) was further improvement (implant 45.2 at NAC, 50.9-66.2 inner, 35.2-58.4 outer areas; autologous 38.5 at NAC,

Citation	Study name and location	Years of study*	Topic or comparison	Number of patients	Design	Population	Criteria for NSM	Pathology	Oncologic outcomes	Surgery Details	Nipple viability and necrosis
										Testing pre-mastectomy and postmastectomy at various time points	37.5-47.7 inner, 25.9 - 40.8 outer) but still less than pre-surgery
Golijanin, 2024 [190]	Oncology Institute of Vojvodina, Sremska Kamenica, Republic of Serbia	2013-2016	LR by subtype (Ki67 or molecular) in NSM with primary implants	156 pts: 53 invasive	Retrospective<br>Follow-up mean 59.26 months<br>Cox proportional hazards model applied in multivariate analysis fir KR	Breast cancer and NSM with primary implant reconstruction with fixed volume silicone prosthesis<br>122 pts received NACT due to advanced disease<br>78.8% received RT<br>Implants were placed between pectoralis major and serratus anterior muscle	Excluded inflammatory breast cancer, extensive skin involvement, Paget's disease, breast cancer lesions with direct extension to skin beyond the dermis (considered LABC)<br>Preoperative clinical examination, ultrasound, mammograph (or MRI if mammogram unclear) and core biopsy	Subareolar tissue sample and frozen section assessed intraoperatively; NAC removed if positive findings<br>2 pts with T4 tumours had infiltration of pectoralis muscle but not skin; in pts with T3 tumours close to subcutaneous tissue a decision of safety of the margin was made (must not be present on margins)	LR in 17 pts (10.9%)<br>Stage, Ki67 and HER2, size of tumour or implant were not risk factors for LR<br>Low ER/PR was risk factor for LR (ER+ OR=1.238, p=0.010)	Lateral incision (adjusted if prior biopsy or BCS due to scars and aesthetics) and removal of breast and fat tissue;<br>NSM only continued if no tumour cells at margins of removed subcutaneous tissue	Not reported
Lin, 2024 [191]<br>Liston, 2024 [192]<br>See also Shanno, 2024 [193]	Massachusetts General Hospital, Harvard Medical School, Boston, MA, USA	2007-2019	NSM complications<br>Expander vs. direct-to-implant<br>Plane of reconstruction (Q4)	2043 direct to implant and 992 expander-implant	Single institution, retrospective<br>At least 2 years follow-up post-operatively	NSM and implant-based reconstruction<br>Excluded delayed or autologous reconstruction<br>Most operations by 3 breast	Not reported	Not reported	Not reported	Type of incision, no other details	Nipple necrosis by incision type: Inferolateral inframammary fold (reference, 1.0), horizontal radial OR=3.823, 95% CI=1.081-

Citation	Study name and location	Years of study*	Topic or comparison	Number of patients	Design	Population	Criteria for NSM	Pathology	Oncologic outcomes	Surgery Details	Nipple viability and necrosis
			Use of ADM (Q5)			surgeons and plastic surgeons					13.515, p=0.037; vertical inferior OR=2.124, 95% CI=0.453-9.944, p=0.339; periareolar OR=14.235, 95% CI=6.248-32.435, p<0.001; extension of prior incision OR=2.98, 95% CI=0.657-13.511, p=0.157
Shanno, 2024 [193]<br>See also Lin, 2024 [191]	Massachusetts General Hospital, Harvard Medical School, Boston, MA, USA	2007-2019	Management of NSM with tumour or atypia in margins	3158 NSM; 117 margins with tumour and 164 with atypia<br>1583 for invasive caner or DCIS; 1575 for risk reduction or symmetry	Median follow-up 67 months<br>6 breast surgeons and 8 plastic surgeons		Broad eligibility for NSM<br>Exclude if direct tumour involvement of NAC on examination or imaging, inflammatory breast cancer, most pts with bloody nipple discharge<br>ASM if nipple involvement without areola involvement on preoperative examination or imaging	Nipple margin specimens not oriented due to small size; frozen section analysis not performed<br>A positive nipple margin if invasive cancer, DCIS, LVI in the nipple margin on permanent analysis; atypia was documented but not considered positive<br>Decision about surgical management on case-by-case basis, but generally	Positive margins in 117 NSM (3.7%): 113 (7.1%) in NSM for known cancer, 4 (0.3%) in prophylactic NSM<br>Atypia in 154 (5.2%) nipple margins: 110 (6.9%) therapeutic NSM and 54 (3.4% prophylactic NSM<br>For pts with tumour in nipple margin: 4 excluded (too short follow-up), 64 (57%) nipple excision (2 with	Areola skin flaps raised, curved clamp grasps nipple duct bundle under the NAC dermis and clamped tissue is sharply divided above and below the clap and the tissue in the clamp comprises the nipple margin<br>Over time shifted towards removal of nipple and preservation of areola; 50% nipple-only in 2009-2015 and 89% nipple-only excisions in 2016-2019	

Citation	Study name and location	Years of study*	Topic or comparison	Number of patients	Design	Population	Criteria for NSM	Pathology	Oncologic outcomes	Surgery Details	Nipple viability and necrosis
							Excluded stage IV breast cancer at diagnosis or within 4 months<br><br>Excluded if nipple excised in initial NSM procedure due to poor position or gross tumour involvement	nipple or NAC excised when nipple margins were positive and retained if atypia<br><br>If nipple/NAC was excised the tissue was examined<br><br>75 (77%) excisions for positive nipple margins contained no residual tumour; 23 (24%) had malignancy<br><br>79% received systemic therapy, 35% PMRT, 21% neither	later NAC excision due to close/positive margins); 34 (30%) NAC excision, 15 (13%) no excision (9 with clear margins in nipple margin specimen, 2 planned RT, 2 small extent of cancer, 2 LVI only<br><br>_________<br><br>Of pts with positive nipple margins: 2 (1.8%) recurrence as subareolar nodule involving the dermis; both had had nipple-only excisions and no residual malignancy in excised nipples<br><br>No significant difference in periareolar recurrence for nipple-only vs. NAC excision; 2/64 (3.1%) vs. 0/34 (0%)<br><br>12 (11%) DM and/or LRR outside NAC,	Nipple or NAC excision at median 35 days after NSM, either alone or with other procedures	

Citation	Study name and location	Years of study*	Topic or comparison	Number of patients	Design	Population	Criteria for NSM	Pathology	Oncologic outcomes	Surgery Details	Nipple viability and necrosis
									including 2 (1.8%) LRR and 7 (6.1%) DM, and 3 (2.7%) both<br>Overall LRR including areola was 6.2% and DM 8.8%; overall recurrence 12%<br>OS at median 67 months follow-up 94%<br>BCSS was 96%<br>In pts with atypia in nipple margins: 164 nipples in 144 pts; in early pts 10 pts had excision (6 NAC and 4 nipple-only); no excisions in later pts<br>At median 60 months was no recurrence in NAC or periareolar skin; 2 (1.3%) other LRR (both in pts with malignant tumour)		
Nashimoto, 2024 [194]	Kameda Medical Center,	2006-2015	Recurrence and survival with NSM, SSM	245 cases: 152 NSM, 49 SSM, 44	Mean follow-up 78.42 months in NSM group	DCIS,<br>Excluded risk-reducing mastectomy	Clinical and imaging without skin or muscle involvement considered for	Intraoperative subareolar tissue biopsy in all cases of NSM; if positive	Of 152 NSM: 5 (3.3%) recurrence, 4 (2.6%) LRR, 0	SLNB with blue dye and radioisotopes; NSM used axillary incision plus partial periareolar (1/4	

Citation	Study name and location	Years of study*	Topic or comparison	Number of patients	Design	Population	Criteria for NSM	Pathology	Oncologic outcomes	Surgery Details	Nipple viability and necrosis
	Chiba, Japan		in Japanese pts with DCIS	simple mastectomy	Single centre, retrospective<br>Cox proportional hazards model for affect of surgical technique on RFS adjusting for age and BMI		NSM or SSM; NSM if no clinical nipple discharge, Paget disease, infiltration of NAC on MRI<br>If NAC infiltration was uncertain on MRI they were evaluated individually	the NSM was converted to SSM<br>No cases of positive surgical margins required reoperation or RT	DM, 1 (0.7%) death<br>Estimated 5-y LR (Kaplan-Meier) 2.4% (0-5.0)<br>Of 4 LR, 2 subcutaneous, 1 areola, 1 lymph node, 0 nipple; all were excised	circumference nearest axilla), mastectomy flaps created in subdermal plane using electrocautery or scissors; endoscopic assistance muscle dissection	
Sagir, 2024 [195]	Istanbul, Turkey	2020-2023	Lateralized parabolic multiplanar incision to reduce necrosis in NAC	243 pts, 326 breasts	Retrospective<br>Mean follow-up 24.6 months	NSM and immediate implant-based reconstruction; therapeutic or prophylactic (numbers not reported)<br>Anatomical textured implants and ADM in all pts<br>160 unilateral, 83 bilateral; 41 prepectoral, 202 dual-plane<br>Exclude autologous, tissue expanders, skin reduction, delayed reconstruction, previous breast surgery, PMRT or previous RT		Not reported	Not reported	Lateralized parabolic multiplanar incision (from axilla curved down to level of nipple but 4-5 cm away from areola)	Full-thickness necrosis in NAC in 12 cases (3.6%) and required debridement or excision<br>Full thickness skin flap necrosis in 9 pts (2.7%)

Citation	Study name and location	Years of study*	Topic or comparison	Number of patients	Design	Population	Criteria for NSM	Pathology	Oncologic outcomes	Surgery Details	Nipple viability and necrosis
Spoor, 2024 [196]	PALGA database, the Netherlands	2000-2021	Intraoperative frozen section in NSM; compared it to permanent section	640 pts, 662 intended NSM	Single centre retrospective<br>Median follow-up of 20 pts with positive histo-pathology was 53 months	Therapeutic NSM and immediate reconstruction with intraoperative frozen section and definitive analysis or SSM with nipple banking (56 SSM)<br>Specimen considered positive if invasive carcinoma non-special type, lobular carcinoma, DCIS, LCIS		Sample sent for intraoperative frozen section; if positive the nipple is excised immediately (if possible only protruding part with lactiferous ducts excised, leaving the areola, otherwise resect areola as well)<br>If negative frozen section but positive permanent section, excision of nipple is offered<br>If nipple or NAC excised it is analyzed<br>Frozen section analysis had sensitivity of 75.2% and specificity of 98.5%	105/662 (15.7%) positive on frozen section; of these 97 were positive on definitive analysis; 32 negative frozen section analysis were positive on definitive analysis<br>Positive on either analysis was 137 (20.7%); of these 115 nipples were resected and 68 (59.5%) had tumour cells and 47 (40.5%) had no tumour cells; 22 pts did not have nipple resection<br>In 20 pts without nipple resection none had LRR by 53 months follow-up	Base of nipple through periareolar incision with lateral extension; nipple everted and gland tissue excised with sharp dissection; in early years used nipple banking as a graft in groin	Not reported
Zhu, 2024 [7]	Zhejiang Cancer Hospital, Hangzhou Institute of Medicine, Hangzhou China	2015-2020	Accuracy of subareolar frozen section in NAC	137 pts, nipple retained in 126 pts	Retrospective<br>Median follow-up 48 months<br>Cox regression analysis; prognostic factors	Primary invasive breast cancer, consecutive cases with NSM<br>Immediate reconstruction (autologous or implant)	Excluded if clinical NAC involvement (nipple depression or discharge), radiologically suspected NAC involvement, inflammatory,	Subareolar frozen sections for all cases; if positive margin could either (a) subareolar reshaving, (b) nipple excision with areolar preservation, (c)	Nipple retained in 126/137 pts<br>Areola retained but nipple excised in 5 breasts<br>Of 15 breasts with positive nipple margin in	A piece of tissue ≈5 mm thick under areola and including retroareolar tissue and ductal tissue beneath bottom of nipple excised using a cold knife and sent	16/127 nipples had necrosis

Citation	Study name and location	Years of study*	Topic or comparison	Number of patients	Design	Population	Criteria for NSM	Pathology	Oncologic outcomes	Surgery Details	Nipple viability and necrosis
					entered in multivariate analysis to identify risk factors of recurrence		Paget's disease of the nipple	NAC excision, (d) observation as determined by surgeon<br>Samples sent for permanent histology; NAC if removed had permanent histology evaluation<br>Frozen section analysis had sensitivity of 81.8% and specificity of 95.3%	frozen section, 3 (20%) had subareolar shave, 5 (33%) nipple excision, 6 (40%) NAC excision, 1 (6.7%) observation only<br>Final pathology found tumour involvement or ADH in 9/15 breasts<br>2/123 negative frozen sections had ADH<br>All positive margins with NAC retained had ADH on final pathology<br>1/11 nipples removed had residual tumour involvement (9.1%)<br>5 (4.3%) had NAC recurrence as first event, 4 (3.4% breast skin or chest wall recurrence, 2 (1.7%) DM	for frozen section analysis	
Braun, 2023 [197]	University of Kansas Medical Center,	2015-2019 training;	Model of factors	Retrospective (training cohort), 181	Included predictors within clinical	NSM and immediate implant-based reconstruction	Pts with tumour >2 cm from NAC or risk-reduction;	Not reported	Not reported	In retrospective set, type of incision was 66% IMF, 33% radial, 2% Wise pattern;	Rates of necrosis in

Citation	Study name and location	Years of study*	Topic or comparison	Number of patients	Design	Population	Criteria for NSM	Pathology	Oncologic outcomes	Surgery Details	Nipple viability and necrosis
	Kansas City, KS	2020-2021 test	affecting NAC necrosis<br><br>Derived model used age, BMI, pack-years smoking, hypertension, NACT, history of breast RT, history of breast augmentation, previous breast incision and type, breast cup size, planned implant size or expander fill (use 0 if filled with air).<br><br>Incision type is important but not included due to data limitations (only had 2 types with non-significant necrosis difference)	pts and 305 breasts<br><br>Prospective (test cohort) 62 pts and 119 breasts	history, physical examination, or surgeon-control<br><br>Mean follow-up 17.7 months	(expander or direct-to-implant)<br><br>Exclude autologous, NAC excision due to surgical pathology results<br><br>Diabetes, hypertension, smoking higher in pts with necrosis	surgeon also considered BMI, breast size, ptosis, prior RT, uncontrolled diabetes, and smoking but were no absolute contraindications			plane of reconstruction was 73% prepectoral and 27% submuscular<br><br>Details from records for retrospective set, necrosis vs. none:<br>• Specimen weight mean 477 g with necrosis vs. 371 g without necrosis, p<0.001<br>• Implant weight mean 221 g vs. 128 g, p<0.001<br>• Implant volume mean 306 mL vs. 238 mL, p=0.008<br><br>Results for prospective set not reported<br><br>There were significant differences between retrospective and prospective data sets in use of IMF incision (increased from 65.9% to 82.3%), prepectoral implants (increased from 73.1% to 95.2%), implant weight (increased from mean 142.31 g to 233.47 g), use of intraoperative SPY (increased from 37.7% to 61.3%)	retrospective/ training set:<br><br>12.4% IMF incision vs. 21.2% radial, p=0.1 [not used in model because other types of incisions were rare]<br><br>14.8% prepectoral vs. 15.9% submuscular, p=1<br><br>NAC necrosis was 46/305 breasts (15.1%) in retrospective set and 8/119 breasts (6.7%) in prospective set

*Year of diagnosis or initial surgery

Abbreviations:
ADH, atypical ductal hyperplasia; ADM, acellular dermal matrix; ALND, axillary lymph node dissection; ASBrS NSMR, American Society of Breast Surgeons Nipple Sparing Mastectomy Registry; ; aOR, adjusted odds ratio; ASM, areola-sparing mastectomy; BCS, breast conserving surgery; BCSS, breast cancer-specific survival; BMI, body mass index; CBC, contralateral breast cancer; CI, confidence interval; CSS, cancer-specific survival; CT, computed tomography; DCIS, ductal carcinoma in situ; DFS, disease-free survival; DIEP, deep inferior epigastric perforator; DM, distant metastasis; DMFS, distant metastasis free survival; DTI, direct to implant; EFS, event-free survival; ELIOT, intraoperative electron-beam radiotherapy; ER/PR, estrogen receptor/progesterone receptor; HER2, human epidermal growth factor receptor 2; HR, hazard ratio; IDC, invasive ductal carcinoma; IBR, immediate breast reconstruction; IMF, inframammary fold; ILC, invasive lobular carcinoma; IORT, intraoperative radiation therapy; LABC, locally advanced breast cancer; LCIS, lobular carcinoma in situ; LD, latissimus dorsi island flap; LCIS, lobular carcinoma in situ; LIN III, lobular intraepithelial neoplasia Grade 3; LN+, lymph node positive (at least one axillary lymph node contains cancer); LLR, locoregional recurrence; LR, local recurrence; LRFS, local recurrence-free survival; LVI, lymphovascular invasion; MRI, magnetic resonance imaging; ms-TRAM, muscle-sparing transverse rectus abdominis musculocutaneous flap; NAC, nipple-areolar complex; NACT, neoadjuvant chemotherapy; NME, non-mass enhancement on MRI; NSM, nipple-sparing mastectomy; OR, odds ratio; OS, overall survival; PALGA, 'Pathologisch Anatomisch Landelijk Geautomatiseerd Archief', Pathological Anatomy National Automated Archive, The Netherlands, www.palga.nl; pCR, pathologically complete response; PET, positron emission tomography; PMRT, postmastectomy radiotherapy; PRO, patient-reported outcomes; pts; patients; QuaRT, quadrantectomy, complete axillary dissection and radiotherapy; RFS, recurrence-free survival; RT, radiotherapy; SEER, Surveillance, Epidemiology, and End Results database, USA; SIEA, superficial inferior epigastric artery flap; SKIN, Skin Ischaemia and Necrosis score, a validated tool [339]; SLNB, sentinel lymph node biopsy; SSM, skin-sparing mastectomy; SPY, SPY fluorescence imaging platform using indocyanine green angiography; TND, tumour-to-nipple distance; TRAM, transverse rectus abdominis musculocutaneous flap; TSSM, total skin-sparing mastectomy

Back to Start of Tables

Table S7. Question 3b/c: Summary Statistics for Patients with NSM, Excluding Four Studies*

Measure	LR	LR in NAC	RR	LRR	CBC	DM	Death	5-y OS	5-y DFS	Total nipple necrosis	Partial NAC necrosis	Flap necrosis
# Studies	42	39	11	22	6	35	13	12	10	44	36	26
Minimum (%)	0.0	0.0	0.7	0.9	0.9	0.0	0.4	90.0	86.0	0.0	0.9	0.4
Maximum (%)	10.9	6.0	3.9	10.1	2.6	15.6	12.2	99.1	98.3	19.5	23.7	23.3
Average (%)	3.9	1.2	2.0	4.3	1.7	4.3	3.3	95.9	91.1	2.8	7.9	7.5
Median (%)	3.4	0.5	1.7	3.8	1.8	3.6	2.2	96.7	92.0	1.9	6.9	6.6

Abbreviations: CBC, contralateral breast cancer; DFS, disease-free survival; DM, distant metastasis; LR, local recurrence; LRR, locoregional recurrence; NAC, nipple-areolar complex; OS, overall survival; RR, regional recurrence

*Excluded two studies in patients who received neoadjuvant chemotherapy (which is correlated with higher-stage disease), one in patients with locally advanced breast cancer, and one with survival rates far outside the range of all other studies (68% 5-y DFS, compared to the next lowest value of 86%).

Back to Start of Tables

Table S8. Question 4: Comparisons of Prepectoral, Subpectoral, and/or Dual-plane Reconstruction. A. Prepectoral versus Other, ADM use Similar

Citation	Study name and location	Years of study*	Topic or comparison	Number of patients	Design	Population	ADM use	Prepectoral details	Subpectoral details	Outcomes	Other
Prepectoral vs. submuscular											
Franceschini, 2021 [215]	Fondazione Policlinico Universitario Agostino Gemelli IRCCS, Università Cattolica del Sacro Cuore, Rome, Italy	2018-2019	Prepectoral without ADM vs. submuscular after NSM<br>Single institution	82 prepectoral, 95 submuscular	Retrospective<br>Follow-up 20 months submuscular and 16 months prepectoral	NSM and immediate implant reconstruction<br>Exclude from NSM: inflammatory carcinoma, LABC infiltrating the skin or NAC, previous RT<br>Relative contraindications to NSM: obesity (BMI >30 kg/m^2), large breasts with severe ptosis, and active smoking<br>Prepectoral or submuscular decision using algorithm including anamnestic, morphological, functional, and oncologic criteria; digital mammography to predict postmastectomy skin flap thickness; final decision in operating room based on flap thickness and perfusion assessment using	Not used	Prepectoral micro-polyurethane-foam-coated implants (microthane) without ADM in subcutaneous plane	Anatomical textured implants, submuscular-subfascial pocket dissection<br>Total coverage of implant beneath the pectoralis major and serratus anterior	Mean operative time: unilateral 247 vs. 319 min, p<0.001; bilateral 306 vs. 368 min, p=0.041<br>Length of hospital stay: no difference<br>Overall major complications: no difference<br>NAC recurrence 0% vs. 1.05%; regional recurrence 1.2% vs. 2.1%; 1 DM (brain) in submuscular group<br><u>PRO for QoL at 6 months after surgery</u><br>Aesthetics: excellent 65.6% vs. 11.3%, good 26.6% vs. 21.0%, satisfactory 6.3% vs. 46.8%, insufficient 1.6% vs. 12.9%, poor 0 vs. 8.1%, p<0.001<br>Chronic pain in pectoral region: none 50% vs.	Other: use blunt dissection to separate skin flap from mammary gland, preserving medial perforators; use perfusion testing

Citation	Study name and location	Years of study*	Topic or comparison	Number of patients	Design	Population	ADM use	Prepectoral details	Subpectoral details	Outcomes	Other
						indocyanine green dye fluoroangiography<br>NSM usually by radial incision on external quadrants, with axillary or IMF in select cases				12.9%, very mild 29.7% vs. 8.1%, mild 12.5% vs. 12.9%, tolerable 6.3% vs. 43.5%, distressing 1.6% vs. 17.7%, very intense 0 vs. 4.8%, p<0.001<br>Shoulder dysfunction/ impaired arm motility: 4.7% vs. 40.3%, p<0.001<br>Skin sensibility: 48.4% vs. 29.0%, p=0.025<br>Sexual/ relationship life compromised: 28.1% vs. 38.7%, p=0.208<br>Contralateral operation for symmetry in unilateral NSM: 3.6% vs. 100%, p<0.001	
Scardina, 2022 [216]	Fondazione Policlinico Universitario Agostino Gemelli IRCCS, Rome, Italy	2018-2021	Prepectoral vs. submuscular after NACT, mostly NSM (92%)<br>Single institution	90 prepectoral, 56 submuscular	Retrospective<br>Follow-up 33 months submuscular and 20 months prepectoral	Histologically proven breast cancer, use of NACT, conservative mastectomy (NSM or SSM), immediate implant<br>Excluded: inflammatory or LABC, BMI >30	Not used	Polytech implant with micro-polyurethane foam-coated shell surface that does not require ADM	Beneath the pectoralis major muscle	Mean operating time 244 vs. 300 min<br>Implant loss 1.11% vs. 1.80%<br>NAC recurrence 0% vs. 1.78%<br>LR 1.11% vs. 3.57%<br>DM 1.11% vs. 1.78%	

Citation	Study name and location	Years of study*	Topic or comparison	Number of patients	Design	Population	ADM use	Prepectoral details	Subpectoral details	Outcomes	Other
						kg/m^2, previous RT, active smoking<br>Digital mammogram to assess breast tissue coverage and potential flap thickness, quality, vascularization<br>NSM 135 pts, SSM 8 pts, bilateral skin-reducing mastectomy 3 pts<br>Therapeutic unilateral 51.4%, bilateral 48.6%<br>Type of reconstruction based on flap thickness and perfusion assessed with indocyanine green dye fluoro-angiography				Symmetrization procedure required in 28% vs. 82% (p=0.03) of pts with unilateral mastectomy	
Prepectoral vs. subpectoral (submuscular or dual-plane)											
Darrach, 2021 [217]	Johns Hopkins University, Baltimore, MD	2017-2018	Prepectoral vs. subpectoral (submuscular or dual-plane); in-patient and prescribed opioid use<br>Single institution, 5 oncologic surgeons, 6	133 pts prepectoral, 89 pts subpectoral (complete or dual-plane)	Retrospective	Pts with breast cancer (except 1 prophylactic due to BRCA2 mutation)<br>Immediate expander-based reconstruction; all pts ordered acetaminophen, celecoxib, and gabapentin, and subpectoral patients	All prepectoral pts<br>Subpectoral in pts with dual-plane placement	Placement between mastectomy flap and pectoralis major<br>ADM either complete coverage or shelf for the lower pole; secured to	Placement beneath the plane of the pectoralis major and atop the pectoralis minor/chest wall<br>Either full coverage with pectoralis major and serratus	Opioid use in first 24 h: 22.2 vs. 44.5 OME, p=0.003<br>Opioid prescribed on discharge: 308.42 vs. 336.99 OME, p=0.3197	

Citation	Study name and location	Years of study*	Topic or comparison	Number of patients	Design	Population	ADM use	Prepectoral details	Subpectoral details	Outcomes	Other
			plastic surgeons			cyclobenzaprine; narcotics as needed<br>Excluded if preoperative opioids, return to operating room or ICU<br>Prepectoral group had more NSM (56% vs. 27%)<br>59% vs. 51% bilateral mastectomy		expander tabs and chest wall	anterior or partial with pectoralis major superiorly and ADM shelf inferiorly		
Kraenzlin, 2021 [218]	Johns Hopkins Hospital, Baltimore, MD	2016-2018	Prepectoral vs. subpectoral (total submuscular or dual-plane) complications<br>5 general surgeons, 6 plastic surgeons, single institution	169 pts (308 breasts) prepectoral; 117 pts (184 breasts) subpectoral	Retrospective<br>Multilinear regression for infection	Tissue expanders<br>56.2% vs. 28.2% NSM<br>Bilateral mastectomy 59.2% vs. 53.0%<br>Prepectoral had lower weight mastectomies (565 vs. 656 g)<br>Technology such as fluorescein or indocyanine green for mastectomy skin flap perfusion were not used	100% prepectoral covering either anterior surface or lower pole (ADM secured to expander tabs and chest wall); 65.8% subpectoral (cases with partial subpectoral coverage)	In space between mastectomy flap and pectoralis muscle with no manipulation of pectoralis major<br>4/6 surgeons determined safest plane by visual assessment of flaps, mastectomy weight, BMI, smoking status, comorbidities	Implant between pectoralis major and pectoralis minor/chest wall with surgical elevation of pectoralis major<br>Partial subpectoral coverage along with ADM or total subpectoral coverage with elevation of pectoralis major and serratus anterior	Clinic visits before definitive reconstruction 6.4 vs. 8.8, p<0.01; anesthesia time lower for prepectoral (p<0.01), partial necrosis rates higher (21.7% vs. 10.9%, p<0.01; mastectomy flap 15.6% vs. 7.1%, p<0.01; NAC 10.9% vs. 4.9%, p=0.02; return to operating room for debridement 6.2% vs. 3.8%, p=0.26)<br>Infection 11.0% vs. 17.4%, p=0.045, p=0.21 after logistic regression to adjust for mastectomy weight	

Citation	Study name and location	Years of study*	Topic or comparison	Number of patients	Design	Population	ADM use	Prepectoral details	Subpectoral details	Outcomes	Other
										Hematoma 2.0% vs. 4.9%, p=0.07<br>Cellulitis 7.8% vs. 12.5%, p=0.09	
Prepectoral vs. subpectoral (dual-plane)											
Sbitany, 2017 [107]	University of California, San Francisco	2012-2016	Prepectoral vs. dual-plane after NSM<br>5 surgical oncologist for mastectomies, 1 reconstructive surgeon	51 prepectoral (84 breasts)<br>115 partial submuscular (dual-plane) (186 breasts)	Retrospective<br>Follow-up 12.5 ± 5.1 months submuscular and 11.1 ± 5.8 months prepectoral	Immediate expander-implant reconstruction after NSM using superior periareolar incision<br>Prepectoral if intraoperative assessment of adequate and viable skin flaps, skin envelope not excessively large or ptotic, oncologically safe; if skin flap thin then used little or no expansion at placement and waited 3 weeks before starting<br>Dual-plane if threatened or exceedingly thin skin flaps, tumour within 1 cm of chest wall, stage 3 or 4 or inflammatory<br>Same incisions used to replace expander with implant, unless PMRT was used	ADM in all pts: for prepectoral expander ADM covered anterior surface and posterior lower pole; for dual-plane expander ADM covered lower half<br>Author now fenestrates ADM to allow more rapid integration and fluid drainage (may have not occurred in earlier pts)	No muscle manipulation; location of inframammary suture line marked about 0.5 cm below planned IMF location; ADM placed in breast pocket and sutured in place with double layer used at lower pole; expander inserted; ADM folder up and over the entire anterior surface of the expander and sutured to chest wall	After NSM, pectoralis major lifted off chest wall from its lateral border and pocket created; inferior border released at its insertion to ribs VI and VII; muscle covered upper half of tissue expander; ADM used to cover and support lower half of expander and sutured to lower released border of pectoralis muscle and to chest wall about 1 cm below planned IMF and carrying onto the serratus muscle<br>Partial submuscular /partial ADM	Overall complications for initial surgery 17.9% vs. 18.8%, p=0.49<br>Revision operation for IMF repositioning: 2.4% vs. 3.2%	

Citation	Study name and location	Years of study*	Topic or comparison	Number of patients	Design	Population	ADM use	Prepectoral details	Subpectoral details	Outcomes	Other
									coverage (dual-plane)		
Sinnott, 2018 [219]	South Nassau Communities Hospital, Oceanside, New York<br>Bridgeport Hospital-Yale New Haven Health, Bridgeport, Connecticut	2010-2017	Prepectoral vs. partial subpectoral; impact of PMRT on outcomes<br>Single plastic surgeon	274 (426 breasts) prepectoral (241 pts or 370 breasts without PMRT, 45 pts or 56 breasts with PMRT)<br>100 pts (163 breasts) partial subpectoral (87 pts or 140 breasts without PMRT, 21 pts or 23 breasts with PMRT)	Retrospective<br>Multivariable logistic regression for outcome of capsular contraction only in PMRT pts<br>Mean follow-up subpectoral 31.9 ± 22.4 months and 19.0 ± 16.9 months	Direct-to-Implant (97.2% vs. 72.4%) or expander-implant<br>Unilateral (% breasts): prepectoral 44.5%, subpectoral 37%<br>Prophylactic (% breasts): prepectoral 45.5%, subpectoral 37.4%<br>No preoperative RT; TND >2 cm on MRI, negative retroareolar biopsy<br>Wise-pattern or modified-Wise pattern incisions only<br>NAC harvested as full thickness graft and grafted to new location<br>PMRT to stage III cancers and selectively to stage II cancers with 1-3 positive nodes	Strattice ADM in all pts	Deepithelialized dermal flap was created from surplus lower-pole breast skin and soft tissue; ADM sewed to medial aspect of pocket, superior aspect of pectoralis major, and superior aspect of inferior deepithelialized dermal flap; implant placed in pocket and ADM sewed down to the chest wall, serratus fascia, and inferior deepithelized dermal flap	Deepithelialized dermal flap was created from surplus lower-pole breast tissue; pectoralis major muscle dissected off the chest wall and inferior aspect sewed to the superior aspect of the inferior deepithelialized dermal flap; a lateral Strattice ADM patch sewn to the dermal flap, pectoralis major muscle, and lateral chest wall fascia; implant placed in the pocket which was closed by placing the last lateral sutures into the ADM.	Capsular contracture (4-grade Baker scale) 5.2% vs. 9.8%, p=0.0588; multivariate analysis p=0.198	Note: <50 pts in each PMRT groups so exclude this analysis<br><25 events so exclude multivariate results except capsular contraction; groups not equivalent and high portion prophylactic so exclude other non-adjusted results
Copeland-Halperin, 2019 [220]	Northern Virginia	2015-2017	Prepectoral vs. dual-plane postoperative opioid use	94 prepectoral (37 direct-to-implant and 56 expander, 1 both), 58 dual-plane (6 direct-to-	Retrospective<br>Multivariate regression for opioid use	Excluded pts with history of substance abuse, chronic pain, opioid medication<br>Flaps evaluated with SPY fluorescent	AlloDerm or DermaCell in all pts	Not reported	Not reported	No significant differences in age, BMI, hypertension, diabetes, tobacco use	Groups similar so unadjusted results may be valid; <25 events so does not meet inclusion

Citation	Study name and location	Years of study*	Topic or comparison	Number of patients	Design	Population	ADM use	Prepectoral details	Subpectoral details	Outcomes	Other
			2 surgeons, one hospital	implant, 52 expander)		indocyanine green imaging				Median postoperative opioid use: 4 vs. 7 days, p=0.009; adjusted IRR=0.68, p=0.016<br>Opioid refills 10.6% vs. 29.3%, p=0.005; adjusted OR=0.34, p=0.027<br>Any complication 0% vs. 5.3%, p=0.298	criteria for multivariate analysis
Avila, 2020 [221]	MedStar Georgetown University Hospital, Washington, DC, or New York University Langone Health	2014-2018	Prepectoral vs. subpectoral after NSM<br>6 breast surgeons, 7 plastic surgeons, 1 institution	228 pts (405 breasts): 202 subpectoral, 203 prepectoral	Retrospective<br>Mixed-effects multivariate logistic regression only for composite outcome of any complications	NSM and implants<br>86.2% bilateral mastectomy<br>Direct-to-implant 73.9% vs. 33.2%<br>Prepectoral use increased over time and subpectoral decreased<br>Prepectoral not used if active smoking, tumour abutting pectoralis<br>Underfill expanders or delay reconstruction if question of poor flap perfusion intraoperatively<br>All prepectoral NSM and consecutive subpectoral until	ADM (DermaCell) in all/most pts<br>Method of use varied by surgeon: most used anterior ADM wrap with a slip of pectoralis muscle (or none) to minimize superior pole contour abnormalitie s; others used anterior wrap with posterior spanning sutures on	Not reported	Not reported	Overall complications (composite complications including NAC necrosis, skin flap necrosis, infection, would dehiscence, hematoma, seroma): 5.91% vs. 9.41%, p=0.1842; multivariate OR=0.61, p=0.190<br>Ischemic complications (including nipple loss due to necrosis) 0.49% vs. 2.97%, p=0.015<br>Nipple necrosis 0.49% vs. 2.97%, p=0.0676	Other: evolution of technique to preserve subdermal vascular supply, retraction is gentle and calculated, careful dissection in supra-areolar region has improved results.<br>Indocyanine green to assess the mastectomy flap vascular supply used earlier in case series but decreased as

Citation	Study name and location	Years of study*	Topic or comparison	Number of patients	Design	Population	ADM use	Prepectoral details	Subpectoral details	Outcomes	Other
						same number as prepectoral pts<br>Meticulous attention during NSM to stay within plane of dissection in the anterior breast capsule, pointing heat sources away from mastectomy flaps, and limiting over-retraction	the back table			Mastectomy flap necrosis 0.49% vs. 5.45%, p=0.0030<br>Nipple loss 1.48% vs. 3.96%, p=0.14 (due to disease 1.48% vs. 0.99%, p=1.0; due to necrosis 0 vs. 2.97%, p=0.015)<br>Unintended reoperation within 30 days: 3.94% vs. 8.42%, p=0.0663; not significant in multivariate analysis<br>No significant differences in infection, hematoma, seroma, implant loss/exchange	experience improved
Banuelos, 2020 [222]	Mayo Clinic, Rochester, Minn.	2012-2016	Prepectoral vs. subpectoral complications in obese pts<br>Five plastic surgeons and four breast surgeons, 1 institution	110 pts (189 breasts) prepectoral<br>83 pts (147 breasts) subpectoral<br>Prepectoral: 131 breasts BMI <35 kg/m^2, 58 BMI ≥35 kg/m^2<br>Subpectoral: 103 breasts	Retrospective<br>Median follow-up 17 months prepectoral and 18.1 months subpectoral	BMI ≥30 kg/m^2<br>Immediate 2-stage implants using textured expanders initially filled to 50% to 75% capacity with air then switched to saline after 2 weeks<br>Bilateral prophylactic 11% vs. 18% of pts<br>Prophylactic mastectomy 28.8%	ADM used in 93.7% vs. 93.2% of breasts<br>Expanders completely wrapped with thick ADM with fenestrations	At discretion of plastic surgeon and pt<br>Intraoperative indocyanine green chorioangio-graphy using the SPY system in 57.7%	Intraoperative indocyanine green chorioangio-graphy using the SPY system in 34%	Any complication 19.6% vs. 20.4%, p=0.773.<br>3.2% vs. 3.4% hematomas, 5.3% vs. 3.4% seromas, 2.7% vs. 4.1% wound dehiscences, 3.2% vs. 4.1% skin flap necrosis, 7.9% vs. 11.6% infections, 6.4% vs. 8.2% device losses (explantation); all	Methods indicate multivariate analysis but cannot find results<br>Rate of bilateral cancers (33% vs. 30%) is extremely high

Citation	Study name and location	Years of study*	Topic or comparison	Number of patients	Design	Population	ADM use	Prepectoral details	Subpectoral details	Outcomes	Other
				BMI < 35 kg/m^2, 44 BMI ≥35 kg/m^2		vs. 36.7% of mastectomies<br>42.9% of mastectomies were NSM vs. 21.1%				differences not significant<br>Authors found some complications increase with BMI, with cutoff around 35 to predict increased risk	
Gabriel, 2020 [104]	Loma Linda, California; Winfield, Illinois; Portland, Oregon; Vancouver, Washington	2009-2017	Prepectoral vs. dual-plane complications in pts with high BMI<br>Single surgeon	Prepectoral June 2013-October 2017; 68 pts (129 breasts)<br>Dual-plane July 2009-August 2017; 65 pts (128 breasts)	Retrospective<br>Stepwise multivariate logistic regression for outcome of any complication<br>Average follow-up 24.1 ± 2.0 months dual-plane vs. 22.7 ± 3.5 months prepectoral	Oncologic pts with immediate reconstruction<br>BMI >30 kg/m^2<br>Excluded from study if direct-to-implant, delayed, revision surgery, hemoglobin A1c >7.5%, active smoking<br>Excluded from prepectoral if not clear margins, extensive skin involvement, chest wall involvement, inflammatory<br>In early part study additional prepectoral exclusion were BMI >40 kg/m^2 with comorbidities, prior RT, immune-compromised, size > 5 cm, deep tumours, late-stage, chest wall involvement, grossly	AlloDerm Select ADM in all pts	1 or 2 pieces AlloDerm (1 AlloDerm Ready to Use, plus if needed 2 piece Contour AlloDerm; majority or all of expander was wrapped with ADM before insertion into prepectoral space<br>Initially filled with air to 70-80% capacity, then exchanged for saline during expansion starting 14-21 days after operation<br>Closed negative-pressure therapy for 7 days	1 large piece ADM sutured to edge of elevated pectoralis major muscle and anchored to IMF<br>Expander filled to 70% to 80% capacity with saline then expanded starting 14-21 days after operation<br>Closed negative-pressure therapy for 7 days	Dual-plane offered to pts with oncologic contraindications to prepectoral and had more RT and chemotherapy<br>Any complication 14.7% vs. 25.8%, p=0.030; p=0.013 in multivariate analysis<br>Capsular contraction 0.8% vs. 7.0%, p=0.019; infection 2.3% vs. 9.4%, p=0.018; seroma 3.1% vs. 13.3%, p=0.003<br>8.6% of dual-plane pts were converted to prepectoral	

Citation	Study name and location	Years of study*	Topic or comparison	Number of patients	Design	Population	ADM use	Prepectoral details	Subpectoral details	Outcomes	Other
						positive axillary involvement<br>Decision on immediate reconstruction based on mastectomy flap perfusion (delayed if poor perfusion)<br>RT if used was applied to fully inflated or partially deflated expander					
Kim, 2020 [223]	Ewha Womans University Mokdong Hospital, Seoul, Korea	2015-2020	Prepectoral vs. dual-plane complications<br>Single institution, single plastic surgeon	53 prepectoral, 114 subpectoral	Retrospective<br>Hierarchical regression analysis controlling for demographic characteristics and ADM size for outcome of hemovac duration and pain score	Unilateral immediate direct-to-implant with ADM<br>Demographics similar in the two groups, except BMI higher in prepectoral group (mean 23.92 vs. 22.65 kg/m^2, p=0.01)<br>Excluded previous BCS, RT<br>NSM used (88.7% vs. 88.6%) unless preoperative evidence of nipple involvement; nipple excised if intraoperative frozen biopsy found malignant cells in tissue under the	MegaDerm or CG CryoDerm ADM in all pts	No muscle manipulation; implant wrapped to cover entire anterior surface and as much posterior surface as single large ADM sheet allowed or using 2 small sheets; placed in prepectoral location	Inferior costal origin of pectoralis major muscle detached; ADM placed between pectoralis major muscle and IMF and sutured; ADM used to cover inferior pole of implant; implant placed in subpectoral-ADM pocket	Pts received postoperative patient-controlled analgesia for 48 h<br>Pain reported by pt using visual analogue scale at 12 h, 24 h, 7 days: mean pain score at 12 h 4.49 vs. 4.10, p=0.18; at 24 h 2.66 vs. 2.36, p=0.29; at 7 days 1.08 vs. 0.80, p=0.14<br>Days to drain removal: 11.09 vs. 14.93 days, p<0.01<br>No significant difference in total complications or specific compilations	Pain may have been masked by patient-controlled analgesia

Citation	Study name and location	Years of study*	Topic or comparison	Number of patients	Design	Population	ADM use	Prepectoral details	Subpectoral details	Outcomes	Other
						nipple and defined as SSM<br>Lateral straight radial incisions and periareolar incisions; care to maintain well vascularized subcutaneous tissue layer<br>Decision of prepectoral or dual-plane made considering intraoperative assessment and discussion with pt				Regression model not suitable for pain scores	
Nealon, 2020 [224]	Massachusetts General Hospital, Boston, Mass.<br>5 surgical oncologists and 2 plastic surgeons	2014-2018	Prepectoral with Vicryl or Vicryl + ADM<br>Subpectoral with ADM or Vicryl	114 prepectoral vs. 142 subpectoral with various mesh and/or ADM<br>104 ADM vs. 67 Vicryl vs. 85 Vicryl + ADM<br>Prepectoral: 0 ADM alone, 30 Vicryl, 85 Vicryl + ADM<br>Subpectoral: 104 ADM, 37 Vicryl, 0 Vicryl+ ADM	Retrospective<br>Univariate analysis<br>Penalized logistic regression	Direct-to-implant	ADM (AlloDerm or FlexHD), Vicryl (synthetic)	Prepectoral: Vicryl mesh folded into a pocket then used to envelop and support the silicone implant; in cases where ADM was also used it was fashioned on the anterior side of the Vicryl pocket and sutured along the IMF toward the axilla for added support	Subpectoral: pectoralis major muscle elevated along inferior and lateral margin, ½ sheet Vicryl or ADM sutured on each side along IMF and lateral mammary crease; implant placed in pocket	Prepectoral vs. subpectoral<br>Overall complications 14.0% vs. 19.7%, p=0.151; penalized regression OR=0.85 (95% CI=0.25-2.75), p=0.785<br>Revision 10.5% vs. 28.2%, p<0.0001; penalized regression OR=0.35 (95% CI=0.08-1.31), p=0.122<br><25 events for individual complications, so data not extracted	

Citation	Study name and location	Years of study*	Topic or comparison	Number of patients	Design	Population	ADM use	Prepectoral details	Subpectoral details	Outcomes	Other
Belmonte, 2021 [225]	University of Virginia, Charlottesville, VA	2017-2019<br>Control 2015-2019	Prepectoral vs. partial submuscular<br>1 surgeon prepectoral; 4 surgeons partial submuscular	Prepectoral: 131 pts, 224 breasts<br>Partial submuscular: 347 pts, 535 breasts	Retrospective	Prepectoral: All consecutive pts (2017-2019) with immediate expander-implant reconstruction<br>Control was partial submuscular (2015-2019)<br>NSM 36% vs. 31%; 57% vs. 63% SSM of breasts<br>Bilateral 71% vs. 54% of pts<br>41% vs. 35% of mastectomies were contralateral	Prepectoral: used confluent contoured Flex HD meshed intraoperatively 1:1.5<br>Partial submuscular: with AlloDerm RTU or Flex HD either confluent or perforated	Not reported	Not reported	On breast basis<br>Major infection 2% vs. 3%, p=0.37<br>Minor infection 5% vs. 6%, p=0.89<br>Seroma 6% vs. 10%, p=0.054<br>Explantation 5% vs. 6%, p=0.77<br>Skin necrosis 10% vs. 8%, p=0.34<br>Wound dehiscence requiring repair 11% vs. 6%, p=0.028; but surgeon used a lower threshold for repair in prepectoral cases	Multinomial logistic regression only for aesthetics; plane of implant not included in analysis
Bozzuto, 2021 [226]	MedStar Georgetown University Hospital, Washington, DC	2015-2017	Prepectoral vs. subpectoral after NSM: postoperative pain during hospital stay and opioid use<br>Single institution, 7 breast surgeons (2 did 88% of cases)	73 pts prepectoral, matched to 73 pts subpectoral (dual-plane?)	Retrospective<br>Matched by age and stage<br>Multivariate analysis	NSM, immediate reconstruction with implant or expander-implant<br>Mostly pts with cancer; 9.6% vs. 15.1% of pts prophylactic<br>80.8% vs. 75.3% bilateral mastectomy<br>≈48% vs. 53% of all mastectomies were prophylactic	Used in both groups, details not reported	Pt selection at discretion of reconstructive surgeon preoperatively and depending on mastectomy flap perfusion<br>Small slip (<2 cm) pectoral muscle raised in some pts as part of ADM attachment	Behind pectoralis muscle, additional ADM support<br>Standard subpectoralis dissection by dividing the junction of serratus anterior and pectoralis major border to the sternal border, IMF,	On multivariate analysis: prepectoral group had lower postoperative pain on visual analogue scale (3.94 vs. 5.25, p<0.001), in-hospital opioid use until hospital discharge (17.14 mg OME vs. 63.03 mg, p=0.03), length of stay (21.36 h vs. 26.28 h, p=0.02)	

Citation	Study name and location	Years of study*	Topic or comparison	Number of patients	Design	Population	ADM use	Prepectoral details	Subpectoral details	Outcomes	Other
									and second costal cartilage		
Haddock, 2021 [227]	University of Texas Southwestern Medical Center	2012 onward (≈2020)	Prepectoral vs. dual-plane perioperative outcomes<br>Single institution, 3 breast surgeons, 2 reconstructive surgeons	102 prepectoral matched to 102 pts subpectoral	Retrospective, propensity-score matched; multivariate analysis	Immediate bilateral tissue expanders; excluded direct-to-implant<br>Standard of care by reconstructive surgeons switched from dual-plane to prepectoral reconstruction in 2017, but without changes in mastectomy technique; <5% were poor candidates for prepectoral reconstruction (high BMI, smoking, large pendulous breasts, poor tissue quality) and decision made considering intraoperative flap quality	AlloDerm; see next columns	Tent-type approach with single sheet of AlloDerm covering anterior aspect of expander; deflated expander positioned under the AlloDerm avoiding the skin then AlloDerm sutured to pectoralis major muscle; intraoperative saline expansion up to but not after detectable tension on skin closure	Single sheet of AlloDerm for dual-plane reconstruction; intraoperative saline expansion limited by skin flap tension	Overall perioperative complications 32% vs. 31%, p=1.000<br>Major complications requiring surgery 21% vs. 21%<br>Hematomas, seromas, impaired would healing, infection not significantly different<br>No increased risks of prepectoral reconstruction in multivariable analysis	
Plachinski, 2021 [228]	The Medical College of Wisconsin, Wauwatosa, Wis.	2016-2019	Prepectoral vs. subpectoral (partial or dual-plane) complications<br>Single institution	83 pts vs. 103	Retrospective<br>Mean follow-up 15.59 months prepectoral and 21.39 months subpectoral<br>Logistic regression modeling	Therapeutic or prophylactic mastectomy, ≥2 y follow-up<br>Immediate expander or permanent implant; decision based on clinical assessment	AlloDerm ADM<br>Prepectoral: 69.9% with complete or anterior coverage<br>Subpectoral: 90.3% with ADM for	Subcutaneous pocket, use of ADM depending on quality of tissue coverage and surgeon preference	Partial muscle coverage with ADM to coverage the lower pole	No difference in major complications, 30.1% vs. 30.1%, p=0.997<br>Minor complications 21.7% vs. 7.8%, p=0.006 mostly due to seroma: seroma 20.5% vs. 4.9%, p=0.001;	Authors have since implemented steps to try to reduce seroma

Citation	Study name and location	Years of study*	Topic or comparison	Number of patients	Design	Population	ADM use	Prepectoral details	Subpectoral details	Outcomes	Other
						Skin flap viability assessed with SPY angiography<br>Mean BMI 28.12 vs. 26.14 kg/m^2, p=0.023; mean mastectomy weight 559.6 g vs. 428.4 g	coverage of lower pole			prepectoral had shorter hospital stay (p=0.007), fewer visits for expansion (p<0.001), less animation deformity (p=0.005); less muscle relaxant prescriptions (13.7% vs. 86.6%, p<0.001); similar pain score (p=0.65), capsular contraction (p=0.791), infections p=0.826)	
Ribuffo, 2021 [229]	Italy	2010-2018	Prepectoral vs. dual-plane complications<br>7 breast-dedicated centres<br>Prepectoral approach adopted in 2015 on	172 pts (207 breasts) prepectoral<br>470 pts (509 breasts) dual-plane<br>Prepectoral: 137 unilateral therapeutic, 35 bilateral (therapeutic or prophylactic)<br>Dual-plane: 431 unilateral therapeutic, 39 bilateral therapeutic or prophylactic	Retrospective<br>Minimum follow-up 1 year<br>Mean follow-up 27.8 months dual-plane and 16.5 months prepectoral	Pts with breast cancer, immediate direct-to-implant reconstruction<br>Prepectoral approach adopted in 2015 on<br>Small-medium size breasts and ptosis grade 1-2 by Regnault scale<br>Exclude BMI >30 kg/m^2, age >65 years, active smoking, previous breast surgery, comorbid conditions (uncontrolled diabetes, immunogenic disorders,	ADM in all pts	Complete coverage with preshaped Strattice or bovine pericardium-derived ADM using overlay tenting technique; implant placed inside ADM then placed in prepectoral space and sutured to muscular fascia	ADM hammock to cover lower lateral pole of implant<br>Pectoralis major muscle dissected and detached from chest wall to create retropectoral pocket; implant put in place, SurgiMend ADM used as sling to cover lower lateral pole of implant and suture to pectoralis major muscle	Complications 20.77% vs. 32.02%, p=0.026<br>Seroma 4.34% vs. 11.2%, p=0.004<br>Hematoma 1.45% vs. 4.71%, p=0.045<br>Surgical site infection 1.93% vs. 3.93%, p=0.2518<br>Wound dehiscence not different, p=0.7893<br>Animation deformity 0% vs. 68.7%<br>Capsular contracture 8.7%	

Citation	Study name and location	Years of study*	Topic or comparison	Number of patients	Design	Population	ADM use	Prepectoral details	Subpectoral details	Outcomes	Other
						congestive heart failure, cardiovascular diseases including hypertension, pulmonary disease, chronic hepatic diseases), previous RT<br>Pt characteristics well balanced including reasons and type of mastectomy and use of chemotherapy				vs. 13.87%, p=0.1818<br>Implant removal 2.42% vs. 3.93%, p=0.3766<br>Aesthetics by blinded evaluators better in prepectoral: bilateral prepectoral 8.3, unilateral prepectoral 7.2, bilateral dual-plane 6.8, unilateral dual-plane 5.3	
Walker, 2021 [230]	Wake Forest Baptist Hospital, Winston-Salem, NC	2014-2018	Prepectoral vs. subpectoral complications in pts stratified by BMI<br>Reconstruction by a single surgeon, switched from subpectoral to prepectoral in summer 2016	92 pts prepectoral, 103 pts subpectoral<br>BMI 25-35 kg/m^2: 54 pts and 60 pts; not enough pts in other BMI groups	Retrospective<br>Mean follow-up 10.9 months prepectoral and 16.1 months subpectoral	Implant-based reconstruction (with or without expander depending on preoperative and desired breast size); delayed or immediate depending on pt circumstances (RT, preference, flap viability)<br>Most pts bilateral reconstruction (89% vs. 96%)<br>BMI higher in prepectoral pts (30.2 vs. 27.8 kg/m^2, p=0.0088)	Prepectoral: wrapped entire prosthesis<br>Subpectoral: used inferolateral ADM sling	ADM wrapped entire prosthesis, sewn to itself on the posterior surface, then inserted in breast pocket just superficial to pectoralis major muscle; sutured to chest wall; expander if used was filled to 50-55% of total volume	Pocket developed using electrocautery in loose areolar tissue plane between pectoralis major and minor; inferolateral sling by suturing ADM to chest wall at IMF and lateral breast curve then inserted prosthetic; expander if used was filled to 25%-30% of total volume	Any major complication: 10.9% vs. 4.9%, p=0.18 (no seroma or flap necrosis; infection 5.4% vs. 3.9%, hematoma 2.2% vs. 0%; asymmetry 1.1% vs. 0%, implant exposure 2.2% vs. 1.0%)<br>Any minor 25.0% vs. 22.3%, p=0.74 (seroma 6.5% vs. 8.7%; infection 16.3% vs. 12.6%; hematoma 0% vs. 1%, flap necrosis 0% vs. 1.9%, asymmetry 9.8% vs. 2.9%)	

Citation	Study name and location	Years of study*	Topic or comparison	Number of patients	Design	Population	ADM use	Prepectoral details	Subpectoral details	Outcomes	Other
Holland, 2022 [231]	University of California, San Francisco	2012-2019	Prepectoral vs. submuscular opioid use after reconstructio n<br>Single surgeon	117 pts prepectoral<br>211 pts subpectoral	Retrospective<br>Multivariate linear regression to control for confounding for outcome of opioid use and NRS pain scores	NSM (76.9% vs. 88.6%) or SSM plus lymph node surgery as indicated; immediate reconstruction with tissue expanders<br>Excluded if preoperative OME ≥60, methadone use, fibromyalgia, concurrent surgeries (hysterectomy, fat grafting)<br>34.2% vs. 34.6% bilateral<br>Reason for mastectomy not reported, but 62.4% vs. 78.2% had either SLNB or ALND	ADM in both groups: prepectoral to wrap entire expander, subpectoral as sling	Choice of plane directed by preoperative discussion and intraoperative skin flap evaluation<br>ADM to wrap entire expander	ADM as a supportive sling between IMF and inferior border of pectoralis major muscle	Prepectoral had lower opioid use (45.0 vs. 80.0 OME, p<0.001; p=0.048 on multivariable analysis), maximum pain scores while admitted (5/10 vs. 7/10, p<0.004; p=0.001 on multivariable analysis)<br>Pain Score using Numerical Rating Scale (NRS), with 0 is no pain and 10 the worst imaginable pain	
Houvenaeghel, 2024 [232]<br>See also Houvenaeghel, 2022 [245]<br>NEW	Marseille, France<br>M-IBR-PPRP-IPC 2022-014	2019-2023	Prepectoral vs. subpectoral complications and satisfaction<br>11 surgeons, surgeons were significantly different between groups	324 prepectoral (increased from 3.1% in 2019 to 61.7% in 2023)<br>529 subpectoral	Retrospective<br>Univariate and multivariate (binary logistic regression) for complications	Immediate implant-based reconstruction (100% vs. 94.7% direct-to-implant)<br>66.4% vs. 44.6% NSM; 33.0% vs. 54.8% SSM<br>108 bilateral prophylactic and 61 bilateral primary breast cancer, 1 bilateral for LR	Resorbable synthetic TIGR Matrix<br>9 (1.7%) subpectoral, 176 (54.3%) prepectoral<br>Prepectoral use varied by year: 20%, 14,%, 92%, 85%, 6.7% for years 2019 to 2023;	Use increased with time (compared with 2019, OR=209.79 for prepectoral in 2023)<br>Many surgeons had strong preference for placement	Not reported<br>(no information on submuscular location)	Complications within 90 days of surgery (all or Grade 2-3; Clavien Dindo classification)<br>• Complications 20.4% vs. 15.3%, p=0.036; regression OR=0.846, p=0.529<br>• G2-3 complications 13.0% vs. 9.8%, p=0.097;	In regression analysis:<br>Smoking, larger breasts (cup size >C), higher ASA status, mesh use, incision (areolar, inverted T) had higher complications<br>Smoking, mesh use, mastectomy

Citation	Study name and location	Years of study*	Topic or comparison	Number of patients	Design	Population	ADM use	Prepectoral details	Subpectoral details	Outcomes	Other
						Implant position and mesh use was at surgeon discretion	note dramatic fall in 2023 due to reports of negative impact on complication although this study data do not support this conclusion			regression OR=0.916, p=0.784<br>• Implant loss 6.5% vs. 4.7%, p=0.271<br>• Reoperation 10.8% vs. 7.4%, p=0.056<br>Pt satisfaction: very good/good 75.9% vs. 73.1%<br>Incision: central 33.0% vs. 50.9%; inferior fold 42.6% vs. 16.3%; rest similar	weight >300 g, diabetes increased grade II-III complications
Asaad, 2023a [233]	Corewell Health, Grand Rapids, MI, USA<br>MD Anderson Cancer Center, Houston, Texas, USA	2017-2019	Effect of obesity on prepectoral vs. subpectoral outcomes	209 pts and 284 breasts: 184 prepectoral, 100 subpectoral	Retrospective<br>PROs using BREAST-Q<br>Univariate and multivariable marginal Cox proportional hazard models to calculate hazard ratios for risk factors	BMI ≥30 kg/m² at time of immediate reconstruction with expander placement, with or without implant exchange<br>Exclude direct-to-implant or delayed-immediate, or change in plane<br>Plane was at surgeon's discretion<br>NSM 4.9% vs. 4%, SSM 94.6% vs. 96%<br>Immediate 92.9% vs. 90%<br>Expander-implant 72.3% vs. 62%;	ADM use 95.1% vs. 70%<br>AlloDerm, Surimend, or Dermacell, depending on surgeon preference	Above pectoralis muscle	Dual-plane or submuscular	• Overall complications 37% vs. 50% p=0.047<br>• Device explantation 12.5% vs. 25%, p=0.008; multivariate HR=0.51, p=0.034<br>• Infections 25% vs. 38%, p=0.024; multivariate HR=0.61, p=0.022<br>• Other outcomes (wound dehiscence, necrosis, implant exposure, capsular contracture, seroma, hematoma) not	Prepectoral had less complications, infections, explanations in obese pts<br>No difference in PROs, but noted that there was no preoperative data

Citation	Study name and location	Years of study*	Topic or comparison	Number of patients	Design	Population	ADM use	Prepectoral details	Subpectoral details	Outcomes	Other
						expander alone 27.7% vs. 38%<br>ADM use: AlloDerm 71.7% vs. 61%, Surgimend 15.2% vs. 7%, Dermacell 8.2% vs. 2%; none: 4.9% vs. 30%				significantly different<br>• Satisfaction With Breast 45.50 vs. 41.57, p=0.469<br>• Psychosocial Well-Being 39.30 vs. 39.43, p=0.915<br>• Sexual Well-Being 17.0 vs. 17.17, p=0.931<br>• Subset with BMI ≥35 kg/m^2 (38 vs. 22 pts): any breast-related complication 38% vs. 68%, p=0.032; explantation 16% vs. 32.3%, p=0.13	
Asaad, 2023b [234]	Corewell Health, Grand Rapids, MI, USA<br>University of Texas M. D. Anderson Cancer Center, Houston, TX, USA	2018-2019	Prepectoral vs. subpectoral (dual-plane) complications and satisfaction	481 pts, 694 breasts: 573 prepectoral, 121 subpectoral	Retrospective<br>Univariate and multivariable frailty models to calculate HR for risk factors at surgery; PRO using BREAST-Q scales of Satisfaction with Breast, Psychosocial Well-Being, and Sexual Well-Being	2-stage implant-based reconstruction<br>Excluded direct-to-implant, delayed immediate, switch in planes<br>Plane was based on surgeon practice<br>Immediate reconstruction in 96% in both groups	ADM used in 97.2% vs. 65.3%<br>AlloDerm 76.4% vs. 58.7%, Surgimend 13.6% vs. 5.8%, Dermacell 7.2% vs. 0.8% based on surgeon preference		Dual-plane	Overall complications 29.3% vs. 28.9%, p=0.887<br>Explantation 11.3% vs. 14%, p=0.436<br>Rates for individual complications were similar (no significant differences)<br>Tissue expander placement complications: any complications 26% vs. 25.6%, no	PROs, 33.7% response rate; 121 prepectoral and 28 subpectoral (not enough to meet our inclusion criteria), no differences<br>Median time to permanent implant exchange shorter in prepectoral group, 150 vs.

Citation	Study name and location	Years of study*	Topic or comparison	Number of patients	Design	Population	ADM use	Prepectoral details	Subpectoral details	Outcomes	Other
										differences in any complication<br>Outcomes of permanent Implants: Any complication 3.3% vs. 3.3%, no significant differences in any	200 days, p<0.001
Hassan, 2024 [235]	University of Texas MD Anderson Cancer Center	2016-2019	Prepectoral vs. subpectoral	172 pts with 179 reconstruction s: 101 prepectoral, 78 subpectoral	Retrospective<br>Univariate and multivariable marginal Cox proportional hazards models to generate hazard ratios<br>Mean follow-up 39.7 months	Pts with immediate expander-implant reconstruction: expander then PMRT then exchange for implant<br>Excluded delayed-immediate, delayed, direct-to-implant<br>PMRT to chest wall, undissected lymphatics, boost to mastectomy flap and nodes	94.1% vs. 69.2%: AlloDerm 72.3% vs. 60.3%; SurgiMend 10.9% vs. 1.3%; DermACELL 10.9% vs. 7.7%		Not reported; does not mention whether dual-plane or submuscular, but use of ADM in 67.2% suggests large portion is dual-plane	Breast-related complications 26.7% vs. 21.8%, p=0.274; HR=1.33, 95% CI=0.74-2.44, p=0.347<br>Device infection 18.8% vs. 15.4%, p=0.307; HR=1.37, 95% CI=0.67-2.86<br>Skin flap necrosis 5.0% vs. 1.3%, p=0.232<br>Device explantation 20.8% vs. 14.1%, p=0.117; HR=1.72, 95% CI=0.84-3.57	
Hung, 2023 [9]	Vanderbilt University Medical Center, Nashville, TN, USA	2013-2019	Early and late complications prepectoral vs. subpectoral expanders	854 pts: 649 submuscular expanders, 205 prepectoral	Retrospective<br>Surgical outcomes up to 1 year after final reconstruction, median follow-up 13.1 months	2-stage expander-implants; 96; 25.4% vs. 14.8% NSM; 96.1% vs. 95.1% immediate	AlloDerm and later Allergan<br>ADM in 93.2% vs. 73.7%	Surgeon assesses skin flap perfusion prior to deciding plane; subpectoral dual-plane if flaps deemed too thin for prepectoral placement;		Complications within 30 days<br>First stage: early complications similar (38.6% vs. 32.6%, p=0.2); failure 8.1% vs. 2.2% (p<0.01), seromas (18.4% vs. 9.1% (p<0.01),	Early and late complications for each stage

Citation	Study name and location	Years of study*	Topic or comparison	Number of patients	Design	Population	ADM use	Prepectoral details	Subpectoral details	Outcomes	Other
					Cox proportional hazard models to predict risks of complications			prepectoral if flaps have appropriate thickness and perfusion		infection 14.8% vs. 6.6% (p<0.01); these were all non-significant (P>0.05) after multivariate analysis<br><br>First stage: late complications 46.3% vs. 33.3% (p<0.01), infection 19.9% vs. 6.1% (p=0.01; HR=2.4), wound dehiscence 6.3% vs. 2.4% (p=0.01)<br><br>Second stage: early 17.5% vs. 16.0% (p=0.63), no differences<br><br>Second stage: late complications 16.5% vs. 16.0% (p=0.97), infection 6.3% vs. 2.0% (p=0.02; HR=5.3, p=0.03)<br><br>Pooled early and late infection: first stage HR=2.2 (p=0.02), second stage HR=4.9 (p=0.01)	
ElSherif, 2024 [236]	Cleveland Clinic, Cleveland, OH, USA	2016-2019	Prepectoral vs. submuscular (but used ADM so	320 pts, 525 NSM: 203 prepectoral, 322 submuscular	Retrospective<br><br>7 breast surgeons and 7 plastic surgeons	NSM for cancer or prophylactic (51% vs. 49%), immediate tissue expander or direct implant reconstruction	ADM in 99% vs. 88%	2 techniques used.<br><br>A. Sizer covered with ADM then sutured to pectoralis	Generally if tumour close to or involving muscle as a posterior margin, in a larger breast	Nipple necrosis 7% vs. 9%, p=0.71<br><br>Reconstruction failure 8% vs. 4%, p=0.093	

Citation	Study name and location	Years of study*	Topic or comparison	Number of patients	Design	Population	ADM use	Prepectoral details	Subpectoral details	Outcomes	Other
			maybe dual-plane)		Mixed effects logistic regression models to compare nipple necrosis and construction failure by adjusting other covariates<br>Multivariate analysis could not be reliably performed due to low numbers of complications and nipple loss	Direct implant in 300 mastectomies<br>23% vs. 56% expanders<br>Inframmamary (75% vs. 67%) and lateral incisions (11% vs. 15%) were most common		major muscle; implant or partially filled expander introduced with Keller Funnel<br>B. ADM sutured around implant in burrito fashion then sutured to pectoralis muscle, drains placed, wound closed	volume, or poor flap/skin quality		
Min, 2024 [237]	University of Ulsan, College of Medicine, Seoul, Korea	2017-2020	Prepectoral vs. partial muscle-splitting subpectoral vs. dual-plane subpectoral<br>2017-2018 generally used dual-plane; subsequently plane according to surgeon preference	349 pts: 92 prepectoral, 169 partial muscle-splitting, 86 dual-plane	Retrospective<br>1 institution, 3 plastic surgeons<br>At least 2 years follow-up<br>Multivariate linear regression	Breast cancer, unilateral direct-to-implant reconstruction<br>Excluded conversion to other method, implant removal, bilateral reconstruction, history of breast surgery<br>Circulation of mastectomy skin flap evaluated with indocyanine green angiography	Human ADM, either CGDerm or MegaDerm	Prepectoral pocket<br>ADM to encase entire anterior surface and most of the posterior surface using a square piece folded/trimmed /sutured to wrap the implant and inserted in prepectoral plane, sutured to pectoralis muscle only if breast was too ptotic or had	Partial muscle-splitting: pectoralis major muscle split parallel to muscle fibre at upper portion of muscle to slightly cover upper edge of sizer; muscle partially elevated from chest wall, manually fenestrated ADM fixed to edge of splitting muscle and IMF;	No differences in rippling (p=0.62), visible implant edges on upper pole (p=0.62), capsular contracture<br>Prepectoral had lower seroma (p=0.008), animation deformity (0 vs. 0 vs. 5.81%, p<0.001), breast pain (3.26% vs. 3.51% vs. 13.95%, p=0.002), upward migration (1.09%	

Citation	Study name and location	Years of study*	Topic or comparison	Number of patients	Design	Population	ADM use	Prepectoral details	Subpectoral details	Outcomes	Other
						No aesthetic revisions such as fat grafting		wide pocket and was risk of implant malposition	implant inserted, ADM trimmed and sutured to lateral chest wall<br>Dual-plane subpectoral: elevate at inferior border of pectoralis major muscle, manually fenestrated ADM sutured along edge of detached pectoralis muscle and IMF, implant inserted, ADM trimmed and sutured to lateral chest wall	vs. 4.68% vs. 38.37%, p<0.001)<br>In multivariate analysis, dual-plane had more seroma (OR=4.223, p=0.002) and implant upward migration (OR=74.292, p<0.001) than prepectoral	

*Year of diagnosis or initial surgery

Abbreviations:
ADM, acellular dermal matrix; ALND, axillary lymph node dissection; ASA status, American Society of Anesthesiologists physical status classification system; BCS, breast conserving surgery; BMI, body mass index; CI, confidence interval; DM, distant metastasis; HR, hazard ratio; IMF, inframammary fold; IRR, incidence rate ratio; LABC, locally advanced breast cancer; LR, local recurrence; NAC, nipple-areolar complex; NACT, neoadjuvant chemotherapy; NSM, nipple-sparing mastectomy; OME, oral morphine equivalents; OR, odds ratio; PMRT, postmastectomy radiotherapy; PRO, patient-reported outcomes; pts, patients; QoL, quality of life; RT, radiotherapy; SLNB, sentinel lymph node biopsy; SSM, skin-sparing mastectomy; TND, tumour-to-nipple distance

Table S9. Question 4: Comparisons of Prepectoral, Subpectoral, and/or Dual-plane Reconstruction. B. Conversion of subpectoral to prepectoral

Citation	Study name and location	Years of study*	Topic or comparison	Number of patients	Design	Population	ADM use	Prepectoral details: revision reconstruction, prepectoral	Subpectoral details: original implant (subpectoral or dual-plane)	Outcomes	Other
Conversion subpectoral to prepectoral (pts are own controls)											
Gabriel, 2018 [238]<br>May overlap with Sigalove, 2019 [239]	Loma Linda University Medical Center, Loma Linda, California<br>Central DuPage Hospital/ Northwestern Medicine, Winfield, Illinois<br>Compass Oncology, Portland, Oregon and Vancouver, Washington	2011-2016	Prepectoral revision of dual-plane reconstruction due to animation deformity<br>Author's practices	57 pts (102 breasts)	Retrospective<br>Mean 16.7 months follow-up after revision surgery	Previous 2-stage implant reconstruction and animation deformity<br>Excluded current smokers, poor skin quality/perfusion, uncontrolled diabetes, RT (except for implant-based latissimus flap reconstruction)	AlloDerm ADM, perforated or pie-crusted	**Revision reconstruction, prepectoral**<br>New pocket above pectoralis muscle; direct-to implant or expander depending on skin flap thickness and tightness; implant or expander placed in pocket and covered with ADM; ADM tacked to subcutaneous tissue superiorly and IMF inferiorly with 3-4 cm cuff on chest wall<br>Expander (if used) was fully inflated at time of operation	**Original implant, dual-plane**<br>Dual-plane<br>Original implant removed via IMF incision, removal of lower-pole capsule and ADM (if present) and anterior capsule if possible<br>Facelift scissor used instead of electrocautery if mastectomy flap was too thin<br>Pectoralis major muscle sutured down to lower pole and chest wall	Presenting complaint (% breasts): 94.1% animation deformity, 95.1% pain, 89.2% animation deformity and pain, 7.8% implant malposition<br>After revision: no animation deformity; pain not measured but not pts complained of pain<br>Complications with revision surgery 3.9% of breasts: 2% seroma, 2% skin necrosis, 1% wound dehiscence, 3.9% implant removal and replacement	

Citation	Study name and location	Years of study*	Topic or comparison	Number of patients	Design	Population	ADM use	Prepectoral details: revision reconstruction, prepectoral	Subpectoral details: original implant (subpectoral or dual-plane)	Outcomes	Other
								Autologous fat grafting used at secondary surgery in pts requiring additional soft tissue coverage			
Sigalove, 2019 [239]<br>May overlap with Gabriel, 2018 [238]	Loma Linda University Medical Center, Loma Linda, California<br>Central DuPage Hospital/ Northwestern Medicine, Winfield, Illinois<br>PeaceHealth Southwest Medical Center, Vancouver, Washington	2015-2018	Prepectoral revision with Inspira implants of dual-plane reconstruction<br>Pts in one author's practice<br>Inspira round implants have higher fill ratio than standard round implants and higher gel cohesivity	64 pts (124 breasts)	Retrospective<br>Mean 18.9 months follow-up	Consecutive pts with dual-plane implants undergoing revision due to animation deformity (99.2% of breasts), pain (99.2%), asymmetry (96.0%), implant malposition (68.5%), size change (26.6%), capsular contracture (16.9%), or rippling (1.6%)<br>Contraindications of revision surgery: poor skin quality or perfusion, thin subcutaneous tissue, uncontrolled diabetes, current smoking<br>½ pts obese; ¼ pts had controlled diabetes<br>93.8% bilateral; 50.8% SSM, 49.2% NSM	AlloDerm ADM, perforated or pie-crusted and GalaFLEX bioabsorbable mesh	Site change to prepectoral; see Gabriel, 2018 [238]<br>ADM placed anterior to the implant, GalaFLEX mesh posterior to the implant and both secured to the chest wall and IMF inferiorly<br>Implants were wrapped with ADM ± absorbable mesh depending on implant size<br>Autologous fat grafting used as needed (47% of breasts) in secondary procedure; if tissues were thin at presentation	Implant removal; see Gabriel, 2018 [238]<br>96% IMF incision; new incision in IMF created if earlier scar was a central mastectomy scar unless it used latissimus dorsi flap<br>Lower pole ADM, if present, was removed together with capsule	3.2% complications (4 breasts in 3 pts):<br>1.6% implant loss<br>1.6% seroma; 0.8% each hematoma, surgical site infection, skin necrosis<br>No capsular contracture<br>All presenting complaints (prior to revision surgery) were resolved and did not recur	

Citation	Study name and location	Years of study*	Topic or comparison	Number of patients	Design	Population	ADM use	Prepectoral details: revision reconstruction, prepectoral	Subpectoral details: original implant (subpectoral or dual-plane)	Outcomes	Other
								then fat grafting was used preoperatively for thickening of the flaps			
Jones, 2019 [240]	University of Illinois College Medicine, Peoria, Illinois<br>Brown University Warren Alpert Medical School at Rhode Island Hospital, Providence, Rhode Island<br>Louisiana State University School of Medicine, New Orleans, Louisiana	2015-2018	Subpectoral to prepectoral conversion<br>Single plastic surgeon	90 pts (142 breasts)	Retrospective<br>Average follow-up 77 weeks (17.8 months)	Patients with subpectoral reconstruction and complaints of animation deformity, implant distortion and tightness<br>Subpectoral to prepectoral implant change<br>Excluded pts with RT only if skin was thin, tight, and telangiectatic, no other exclusions	AlloDerm ADM for prepectoral replacement	**Revision reconstruction, prepectoral**<br>Prepectoral plane identified between superficial aspect of pectoralis major muscle and overlying upper mastectomy flap; ADM trimmed to create teardrop shape and sutured to anterior aspect of pectoralis major muscle at its cusp with the overlying mastectomy skin flap and sutured to leave an inferior access window for implant insertion;	**Original implant, subpectoral or dual-plane**<br>Incision through original mastectomy scar; upper flap everted, junction of pectoralis major muscle and ADM incised with electrocautery; broad-based dissection allows muscle to be returned to the chest wall without tension; pectoralis major sutured to rib periosteum	100% resolution of animation deformity<br>Most pts reported improved range of shoulder movement<br>Improvement in overall breast aesthetics including better cleavage appearance<br>Complications with revision surgery: 4.2% infection; 2.1% seroma; 0.7% hematoma, dehiscence, partial necrosis, explantation<br>28.9% minor contour deformity and 4.9% rippling treated with fat grafting in 18.3% of pts<br>No capsular contraction by 44 months follow-up; attributed to use of anterior ADM coverage and use of	Note: authors indicate skin flaps can be considered vascular-delayed

Citation	Study name and location	Years of study*	Topic or comparison	Number of patients	Design	Population	ADM use	Prepectoral details: revision reconstruction, prepectoral	Subpectoral details: original implant (subpectoral or dual-plane)	Outcomes	Other
								implant inserted under the ADM and anterior ADM sutured to chest wall along the IMF		biofilm reduction protocol including using a Keller funnel to insert the implant	
Holland, 2020 [241]	University of California, San Francisco	2015-2018	Conversion of subpectoral to prepectoral due to animation deformity<br><br>Single institution, single surgeon	80 breasts in 45 pts, including 35 pts with bilateral conversion in 70 breasts<br><br>52.5% had preoperative fat grafting due to <1 cm subcutaneous tissue	Retrospective<br><br>Median follow-up 15.2 months	All pts who had subpectoral to prepectoral conversion to treat animation deformity<br><br>Required to have enough overlying subcutaneous ties to mask contour of ne implant and provide durable layer of vascularized tissue above the implant; ideal candidates had ≥1 cm of subcutaneous tissue on the pinch test and otherwise had preoperative fat grafting (52.5%) if appropriate donor sites; if no donor sites either did not do exchange or removed stretched and excess thin skin<br><br>Smoking cessation and improvement of poorly controlled diabetes and other	AlloDerm ADM used in 81.3% of revisions to prepectoral implants	**Revision reconstruction, prepectoral**<br><br>Plane between pectoralis major and overlying subcutaneous tissue developed using low electrocautery and sharp dissection to free the pectoralis muscle from the overlying muscle and create a pocket; pectoralis major muscle sutured to chest wall<br><br>Cohesive gel implants were preferred to reduce rippling; envelope	**Original implant, subpectoral or dual-plane**<br><br>Removal of implant, separation of the pectoralis major muscle from overlying skin flap, attachment of muscle to chest wall<br><br>Typically used same incision as previously; in NSM generally a superior periareolar on IMF; in other pts was transverse breast scar<br><br>Inferior border of pectoralis muscle incised and capsule and implant	Resolution of animation deformity in all pts<br><br>Complications: 2 (2.5%) required reoperation (1 hematoma and 1 infection); 11 (13.8%) treated for infection<br><br>No reconstructive failures<br><br>21.25% had asymmetry and 11.25% had cosmetic revision<br><br>Capsular contraction in 6.25%<br><br>Pts with ADM had less asymmetry (15.4% vs. 47.0%, p=0.01), capsular contracture (1.5% vs. 26.7%, p=0.01)<br><br>Pts with preoperative fat grafting had less asymmetry (11.9% vs. 31.6%, p=0.05), less	

Citation	Study name and location	Years of study*	Topic or comparison	Number of patients	Design	Population	ADM use	Prepectoral details: revision reconstruction, prepectoral	Subpectoral details: original implant (subpectoral or dual-plane)	Outcomes	Other
						comorbidities was required prior to surgery<br>10 breasts (12.5%) had had RT<br>63.8% had NSM		around implant supported with ADM covering the entire anterior surface and small portion of posterior/ inferior surface; no touch technique used to place implants in pocket; implant not sutured in place; skin closed in layers	removed along with ADM if present unless there was thin overlying skin	capsular contracture (0% vs. 13.2%, p=0.02), cosmetic revision surgery (4.8% vs. 18.4%)<br>Postoperative prophylactic antibiotics in 60% of pts and associated with fewer infections (2.1% vs. 31.3%, p<0.01)	
Salgarello, 2023 [242]	Fondazione Policlinico Universitario Agostino Gemelli IRCSS, Rome, Italy	2018-2022	Submuscular (subpectoral?) to prepectoral conversion	63 pts, 87 breasts	Retrospective	Complaints of animation deformity, chronic pain, poor cosmetic results (retained breast, soft tissue waterfall, asymmetry)<br>Adequate layer of subcutaneous fat (about 8 mm) in mastectomy skin flaps as determined by pinch test in upper pole and ultrasound<br>Lipofilling in some patients with thinner flaps	Used in first 7 pts, complete coverage	18 pts (7 no RT and 11 with RT) had lipofilling prior to exchange<br>Subpectoral pocket opened at lower edge of pectoralis major muscle and expander or implant explanted; capsule from lower edge of pectoralis major to IMF was excised and lower edge sutured to	7 pts with anatomical microtextured implants and ADM, rest with polyurethane foam-coated implants	Cosmetic evaluation with 0-2 points Likert scale<br>Breast contour score of 2 for 81.6% and score of 1 for 18.4%<br>IMF crease was 2 in 74.7% and 1 in 25.3%<br>Breast position 2 in 87.36% and 1 in 12.64%<br>Breast projection 2 in 84% and 1 in 16%<br>Breast shape 2 in 97.7% and 1 in 2.3%	

Citation	Study name and location	Years of study*	Topic or comparison	Number of patients	Design	Population	ADM use	Prepectoral details: revision reconstruction, prepectoral	Subpectoral details: original implant (subpectoral or dual-plane)	Outcomes	Other
						If previous RT, damage was evaluated and exchange only if LENT-SOMA score grades 1-2; they were candidates for fat grafting		posterior capsule on the chest wall		Overall score was 2 in 96.5% and 1 in 3.5%<br>Capsular contracture score Baker 1a in 47.1%, 1b in 42.5%, 2 in 10.4%<br>Pain resolved in all pts<br>BREAST-Q improved from preoperative values: Satisfaction with Breasts 37 to 94 at 12 moths; Psychosocial Well-Being 40 to 86; Sexual Well-Being 34 to 83; Physical Well-Being Chest 23 to 94, animation deformity 38 to 96	

*Year of diagnosis or initial surgery

Abbreviations:
ADM, acellular dermal matrix; IMF, inframammary fold; LENT-SOMA, Late Effects on Normal Tissue—Subjective, Objective, Management, Analytic system for grading of side effects after radiotherapy; NSM, nipple-sparing mastectomy; pts, patients; RT, radiotherapy; SSM, skin-sparing mastectomy

Back to Start of Tables

Table S10. Question 4: Comparisons of Prepectoral, Subpectoral, and/or Dual-plane Reconstruction. C. Prepectoral, Subpectoral, and/or Dual-plane Reconstruction; ADM different in each arm

Citation	Study name and location	Years of study*	Topic or comparison	Number of patients	Design	Population	ADM use	Prepectoral details	Subpectoral details	Outcomes	Other
Prepectoral vs. submuscular											
Bettinger, 2017 [108]	Kaiser Permanente Medical Center San Jose, San Jose, Calif<br><br>Single institution	2008-2015	Prepectoral with ADM<br><br>Dual-plane with ADM sling (Classic method, <50 pts)<br><br>Submuscular (no ADM)	213 pts, 294 breasts: 110 pts (165 breasts) prepectoral; 40 pts (52 breasts) classic (ADM sling), 63 pts (77 breasts) no ADM	Retrospective<br><br>Multivariate analysis<br><br>Minimum follow-up ranged from 6 months to 6 years	Consecutive reconstructions for breast cancer or prophylactic<br><br>Surgical technique left to discretion of surgeon; no information reported about pt selection/assignment to plane of implant<br><br>Exclude mesh other than AlloDerm<br><br>34% prophylactic<br><br>28% of mastectomies were contralateral<br><br>Implant placement typically 3 months after chemotherapy or 6 months after RT	AlloDerm (thick)<br><br>Prepectoral with ADM<br><br>Dual-plane with ADM sling (Classic method)<br><br>Submuscular (no ADM)			Complications limited to Clavien score grade IIIB (requiring surgical, endoscopic, or radiological intervention under general anesthesia) for seroma, infection, hematoma, skin and nipple necrosis, expander deflation, expander or implant loss<br><br>Expander complications: 13.33% prepectoral with ADM vs. 6.49% submuscular without ADM<br><br>With Classic =1, prepectoral adjusted RR=0.25 (95% CI=0.06-1.00; no ADM adjusted RR=0.12 (95% CI=0.02-0.72); prepectoral to no ADM RR=2.08 (calculated)	Note: <50 pts classic so should be excluded from comparisons

Citation	Study name and location	Years of study*	Topic or comparison	Number of patients	Design	Population	ADM use	Prepectoral details	Subpectoral details	Outcomes	Other
										Implant complications: too few for analysis	
Talwar, 2024 [243]	University of Pennsylvania<br>Protocol no. 850387	2018-2021	Prepectoral vs. submuscular implants	350 pts, 634 breasts: 197 prepectoral, 437 total submuscular<br>After matching, 146 breasts in each group<br>Only difference is ADM use, which depends on plane	Retrospective<br>3 plastic surgeons<br>1:1 propensity score match to control for baseline difference	Direct-to-implant or staged implants<br>Excluded dual-plane implants, prepectoral implant with latissimus flap<br>52.7% breast cancer, 47.3% prophylactic mastectomy<br>65% SSM; 89.7% 2-stage reconstruction	AlloDerm and FlexHD (not sterile) used in most prepectoral (97.5%); ADM not in submuscular 3%); 96.6% and 4.8% in matched cohorts	Pts with smaller breasts and confined postmastectom y pocket or health-appearing mastectomy skin flaps from well-trusted breast surgeon;<br>Less likely if PMRT or unilateral reconstruction		Entire and matched cohorts<br>Surgical site infection 14.7% vs. 3.9%, p<0.001; matched 15.8% vs. 3.4%, p<0.001<br>Cellulitis 6.6% vs. 2.3% p=0.014; matched 6.2% vs. 3.4%, p=0.411<br>Seroma 21.8% vs. 11.4%, p<0.001; matched 26.0% vs. 10.3%, p<0.001<br>Expander loss 16.2% vs. 2.3%, p<0.001; matched 20.5% vs. 2.7%, p<0.001<br>Implant loss 5.1% vs. 1.8%, p=0.044; matched 2.7 vs. 2.1%, p=1<br>Total explantation 21.3% vs. 4.1%, p<0.001; 23.3% vs. 4.8%, p<0.001<br>Mastectomy flap necrosis 3.0% vs. 9.2%, p=0.01; matched 4.1% vs. 10.3%, p=0.070	Time-to-event analysis: time to infection 95.6 versus 160.8 days, p=0.034; prepectoral infections more often treated surgically 93.1% vs. 47.1%, p<0.001<br>Non-sterile ADM, and longer use of drains may contribute to infection

Citation	Study name and location	Years of study*	Topic or comparison	Number of patients	Design	Population	ADM use	Prepectoral details	Subpectoral details	Outcomes	Other
										NAC necrosis 2.5% vs. 11.0%, p<0.001; matched NAC necrosis 0.7% vs. 7.5%, p=0.008	
Prepectoral vs. subpectoral (dual-plane)											
Chen, 2023b [244]	Weill Cornell Medicine, New York, NY	2012-2021	Prepectoral (no ADM) vs. dual-plane with ADM vs. dual-plane with P4HB	220 pts, 393 samples: 161 prepectoral (no mesh), 122 dual-plane with ADM, 96 dual-plane with P4HB [and 14 with total submuscular, less than current review threshold]	Retrospective, univariate; Cox proportional-hazards model	2-stage reconstruction<br>No significant baseline differences between groups	Prepectoral (no ADM) vs. dual-plane with ADM vs. dual-plane with P4HB	Anterior to pectoralis muscle and directly under the soft tissue	Dual-plane: partially subpectoral with support material<br>Total submuscular: complete envelopment by pectoralis and serratus anterior muscles	Mean time to full expansion 53.4 vs. 104 vs. 68.8 days<br>Necrosis 11.8% vs. 13.1% vs. 4.2%<br>Infection 13.0% vs. 13.1% vs. 7.3%<br>Revision surgery 46.6% vs. 46.7% vs. 41.7%<br>Capsular contraction 30.4% vs. 34.4% vs. 47.9%;<br>Capsular contraction, univariate: OR=1.20 (95% CI=0.73-1.98, p=0.47) compared with dual + ADM; OR=2.10 (95% CI=1.25-3.56, p=0.005) compared with dual + P4HB<br>Capsular Contracture, multivariate: dual + ADM vs. prepectoral HR=1.00 (95%	

Citation	Study name and location	Years of study*	Topic or comparison	Number of patients	Design	Population	ADM use	Prepectoral details	Subpectoral details	Outcomes	Other
										CI=0.66-1.52, p=0.99); dual + P4HB vs. prepectoral HR=1.58 (95% CI=1.05-2.36, p=0.03)	
Houvenaeghel, 2022 [245]<br>See also Houvenaeghel, 2024 [232]	Marseille, France<br>M-IBR-PPRP-IPC 2022-014	2020-2022	Prepectoral vs. subpectoral post-surgical outcomes and pt satisfaction<br>8 surgeons (4 only did subpectoral)	98 prepectoral, 218 subpectoral	Retrospective<br>Univariate and multivariate analysis (binary logistic regression) for complications	Immediate implant-based reconstruction<br>68.4% vs. 36.2% NSM; 31.6% vs. 62.8% SSM<br>48 bilateral (19 prepectoral and 29 subpectoral)<br>14.3% vs. 15.1% prophylactic<br>Incisions for NSM usually (91%) in breast inferior fold	TIGR Matrix (resorbable synthetic mesh): 86.6% prepectoral pts, 0.9% subpectoral pts	Most used TIGR Matrix<br>Other details not reported	Not reported<br>(no information on implant location)	Complications 17.3% vs. 12.9% (p=0.301; multivariate OR=1.172, p=0.664); Grade 2-3 13.2% vs. 10.1% (p=0.441; multivariate OR=1.193, p=0.672)<br>Patient satisfaction (good or very good): 74.5% vs. 62.4%, p=0.035<br>Duration of surgery 80 vs. 100 min, p<0.0001; multivariate OR=0.48, p=0.007	
Houvenaeghel, 2024 [232]<br>See also Houvenaeghel, 2022 [245]	Marseille, France<br>M-IBR-PPRP-IPC 2022-014	2019-2023	Prepectoral vs. subpectoral complications and satisfaction<br>11 surgeons, surgeons were significantly different	324 prepectoral (increased from 3.1% in 2019 to 61.7% in 2023)<br>529 subpectoral	Retrospective<br>Univariate and multivariate analysis (binary logistic regression) for complications	Immediate implant-based reconstruction (100% vs. 94.7% direct-to-implant)<br>66.4% vs. 44.6% NSM; 33.0% vs. 54.8% SSM	Resorbable synthetic TIGR Matrix<br>9 (1.7%) subpectoral, 176 (54.3%) prepectoral<br>Prepectoral mesh use	Use increased with time (compared with 2019, OR=209.79 for prepectoral in 2023)<br>Many surgeons had strong	Not reported<br>(no information on submuscular location)	Complications within 90 days of surgery (all or Grade 2-3; Clavien Dindo classification)<br>• Complications 20.4% vs. 15.3%, p=0.036; regression	In regression analysis:<br>Smoking, larger breasts (cup size >C), higher ASA status, mesh use, incision (areolar, inverted T)

Citation	Study name and location	Years of study*	Topic or comparison	Number of patients	Design	Population	ADM use	Prepectoral details	Subpectoral details	Outcomes	Other
			between groups			108 bilateral prophylactic and 61 bilateral primary breast cancer, 1 bilateral for LR<br>Implant position and mesh use was at surgeon discretion	varied by year: 20%, 14,%, 92%, 85%, 6.7% for years 2019 to 2023; note dramatic fall in 2023 due to reports of negative impact on complication s	preference for placement		OR=0.846, p=0.529<br>• G2-3 complications 13.0% vs. 9.8%, p=0.097; regression OR=0.916, p=0.784<br>• Implant loss 6.5% vs. 4.7%, p=0.271<br>• Reoperation 10.8% vs. 7.4%, p=0.056<br>Pt satisfaction: very good/good 75.9% vs. 73.1%<br>Incision: central 33.0% vs. 50.9%; inferior fold 42.6% vs. 16.3%; rest similar	had higher complications<br>Smoking, mesh use, mastectomy weight >300 g, diabetes increased grade II-III complications

*Year of diagnosis or initial surgery

Abbreviations:
ADM, acellular dermal matrix; ASA status, American Society of Anesthesiologists physical status classification system; CI, confidence interval; HR, hazard ratio; LR, local recurrence; NSM, nipple-sparing mastectomy; OR, odds ratio; P4HB, poly-4-hydroxybutyrate; PMRT, postmastectomy radiotherapy; pts, patients; NAC, nipple-areolar complex; RT, radiotherapy; SSM, skin-sparing mastectomy

Back to Start of Tables

Table S11. Types of Mesh

Human ADM	Porcine ADM	Bovine ADM	Synthetic, absorbable	Synthetic, partially absorbable	Synthetic, permanent
• AlloDerm<br>• Allomend<br>• Dermacell<br>• DermaMatrix<br>• Cortiva (formerly AlloMax and NeoForm)<br>• FlexHD<br>• SimpliDerm<br>• CGCryoDerm, CGDerm<br>• Epiflex<br>• MegaDerm	• Strattice<br>• Artia<br>• Permacol<br>• Meso Biomatrix<br>• Native<br>• Protexa<br>• Braxon	• SurgiMend<br>• I-real<br>• Veritas<br>• Tutomesh	• Vicryl/polyglactin mesh<br>• Poly-4-hydroxybuyrate (P4HB); bioabsorbable in 18-24 months<br>• GalaFLEX (P4HB)<br>• Phasic (P4HB)<br>• TIGR Matrix (long-term resorbable mesh with dual stages of fast and slow resorbing fibres)<br>• Seri Surgical Scaffold, bioresorbable silk (discontinued)<br>• DuraSorb (resorbable polydioxanone mesh)	• Seragyn: polypropylene and absorbable polyglycolic acid	• TiLOOP Bra (titanium coated polypropylene mesh)<br>• Surgimesh (non-woven polypropylene microfibers)<br>• SURGIMESH®PET (polyester)<br>• Breform (woven polyester)

Back to Start of Tables

Table S12. Question 5: Acellular Dermal Matrix. A. ADM versus none

Citation	Study name and location	Years of study*	Design	Type of ADM or mesh	Plane of implant, mesh location	Number of patients	Population	Other details	Outcomes	Notes
ADM (human) vs. none										
Seth, 2012 [13]	Northwestern Memorial Hospital, Chicago, Illinois<br>1 institution, 7 mastectomy surgeons, 6 reconstructive surgeons	2006-2008	Retrospective<br>Multiple regression<br>Mean follow-up 23.2 vs. 24.4 months	AlloDerm or FlexHD vs. none	Under pectoralis major and ADM vs. submuscular	417 pts (592 breasts): 199 breasts ADM, 393 breasts no ADM	Consecutive series of immediate expander reconstruction and implant exchange<br>RT, if used, was before implant exchange<br>Preoperative RT was exclusion factor for ADM use<br>Calculated 30% of mastectomies were contralateral; cancer not mentioned	With ADM: Pectoralis muscle disinserted, ADM sutured to lower pole defect, inferior aspect of ADM sutured to IMF and lateral aspect to serratus muscle fascia, expander inserted<br>Authors stated no difference in complications between AlloDerm and FlexHD, but data not shown	Effect of ADM (multiple regression):<br>• Total complications OR=1.37, 95% CI=0.87-2.17, p=0.17;<br>• Infection OR=1.67, 95% CI=0.81-3.47, p=0.16;<br>• Major flap necrosis OR=1.32, 95% CI=0.70-2.49, p=0.41;<br>• Non-operative OR=1.36, 95% CI=0.7-2.39, p=0.33;<br>• Operative OR=1.64, 95% CI=0.95-2.83, p=0.08;<br>• Explantation or conversion to flaps OR=1.17, 95% CI=0.63-2.19, p=0.62	
Jordan, 2014 [262]	Northwestern University, Chicago, Illinois<br>1 surgeon	2011-2012 after ADM algorithm; 2008-2009 before ADM algorithm	Retrospective<br>Multiple logistic regression for effect of algorithm	ADM (AlloDerm or FlexHD) or none	ADM: Inferior aspect of pectoralis major released; crescent-shaped piece of ADM affixed to reconstitute the	193 breasts before algorithm (84% used ADM)<br>179 breasts after	NSM or SSM and immediate tissue-expander reconstruction<br><u>Algorithm for ADM use vs. total muscular coverage</u><br>BMI: high and large breasts use delayed reconstruction	Resource-sensitive algorithm for selective acellular dermal matrix use with indications and contraindications	Complications 22.8% before algorithm vs. 20.7% after, p=0.138; after adjusting for confounders no difference (p>0.05) for infection, seroma, flap	Follow-up too short for long-term complications (capsular contraction)

Citation	Study name and location	Years of study*	Design	Type of ADM or mesh	Plane of implant, mesh location	Number of patients	Population	Other details	Outcomes	Notes
			Algorithm to decide on ADM use vs. no algorithm		inferior and lateral borders of the breast<br>No ADM: serratus anterior fascia or muscle elevated to cover expander/ implant	algorithm (36% used ADM)	or ADM; high and small breasts ± ADM; low and large breasts ± ADM; low and small breasts use no ADM<br>Radiation: preoperative no ADM; anticipated PMRT use ADM<br>SLND+ use ADM<br>Pectoralis: intact and wide ± ADM; intact and narrow use ADM; compromised use ADM<br>Flap vascularity: good and skin excess use ADM; good and no skin excess do not use ADM; poor do not use ADM	based on body mass index, breast size, radiation therapy, flap vascularity, and pectoralis anatomy	necrosis, explantation, overall complications; aesthetics 2.75/4 vs. 3.03/4, p=0.138	Note: algorithm appears to be for type of surgery and not specifically ADM use as ADM is used in absence of complete submuscular coverage
Woo, 2017 [10]	Sungkyunkwan University School of Medicine and Ewha Womans University Mokdong Hospital, Seoul, South Korea<br>5 ablative surgeons and 4 reconstructive surgeons	2010-2016	Retrospective propensity-score matched cohort analysis using mastectomy weight, drain days in situ, and initial inflation<br>Multivariable generalized estimating equation analysis to adjust for surgeons	ADM (human, non-fenestrated) vs. none	ADM covered inferolateral portion of expander (dual-plane, or to fill gap in otherwise submuscular insertion)<br>Total submuscular in group without ADM (use of serratus anterior muscle and/or serratus fascia)	Before matching 533 pts (574 breasts)<br>Matched groups: 199 ADM reconstructions and 199 non-ADM	Immediate expander-implant reconstruction<br>ADM used non-selectively (consecutively) from Nov 2013 if the pt agreed and it was available	Not reported	No significant difference in skin flap complications (16.1% vs. 16.1%, p>0.999), seroma (4.0% vs. 8.5%, p=0.065), infection (3.0% vs. 3.5%, p=0.781), overall complications (21.1% vs. 26.1%, p=0.251), major complications (13.1% vs. 19.1%, p=0.110), reconstruction failure (2.0% vs. 2.0%, p>0.999)	
Lee, 2020 [263]	Sungkyunkwan University School of Medicine,	2010-2018	Retrospective<br>Multivariate for effect of BMI and	ADM (not specified)<br>Mainly AlloDerm	ADM: coverage of inferolateral aspects of tissue expander	738 pts ADM, 693 pts no ADM	Immediate unilateral reconstruction with expander in subpectoral pocket, ADM		Hematoma: ADM vs. no ADM by multivariate analysis, OR=0.919, 95%	

Citation	Study name and location	Years of study*	Design	Type of ADM or mesh	Plane of implant, mesh location	Number of patients	Population	Other details	Outcomes	Notes
Lee, 2018, 2019 [264,265]	Seoul, South Korea		predictors of hematoma	or CGDerm/ CGCryoDerm based on Lee, 2018, 2019 [264,265]	No ADM: serratus anterior muscle fascia		according to surgeon preference		CI=0.409-2.068, p=0.839	
Pires, 2024 [266]	University of Utah School of Medicine	2020-2022<br>Stopped use of ADM in May 2021 and excluded dates May-July 20-21 to allow learning without ADM	Retrospective review<br>1 institution, 3 plastic surgeons<br>Multiple variable mixed effects logistic regression for effect of ADM or no-ADM on outcomes	AlloDerm, DermACELL, or Cortiva, meshed at 1:2 or 1:1.5 ratio using a skin graft mesher	Prepectoral<br>ADM vs. no ADM	69 pts (98 breasts) with ADM<br>55 pts (98 breasts) no ADM	Consecutive pts with mastectomy and concurrent prepectoral expander<br>ADM group had less NACT but more adjuvant chemotherapy<br>Used tabbed tissue expanders<br>Any concern for mastectomy flap viability prompted reconstructive delay (and not addition of ADM)	Concerns of mastectomy flap viability prompted reconstructive delay	Post-operative outcomes within 3 months<br>•Hematoma 1% vs. 3.1%, p=0.62<br>•Seroma 27.6% vs. 37.8%, p=0.10; multivariate OR=3.21 (95% CI=0.9-11.44, p=0.07)<br>•Dehiscence 3.1% vs. 5.1%, p=0.52<br>•Minor infection 10.2% vs. 14.3%, p=0.38<br>•Major infection 8.2% vs. 8.2%, p=1.00<br>•Any infection OR=0.89, 95% CI=0.40=1.95, p=0.76<br>•Mastectomy flap necrosis 8.2% vs. 5.1%, p=0.39<br>•Unplanned reoperation 15.3% vs. 17.3%, p=0.70; OR=1.41, 95% CI=0.43-4.69, p=0.57	No independent associations between seroma, hematoma, wound dehiscence, mastectomy skin flap necrosis, infection, unplanned return to the operating room, or explantation after controlling for age, body mass index, history of diabetes, tobacco use, neoadjuvant chemotherapy, and postoperative RT<br><br>Note that other than seroma, any infection, and return to operating room, the number of events was small multivariate

Citation	Study name and location	Years of study*	Design	Type of ADM or mesh	Plane of implant, mesh location	Number of patients	Population	Other details	Outcomes	Notes
									•Explantation 6.1% vs. 12.2%	results were not extracted
ADM (not specified) vs. none**										
Sorkin, 2017 [69]	MROC study<br>10 centres (USA and Canada), 58 surgeons	2012-2015	Prospective<br>Bivariate analysis and mixed-effects regression<br>2-year follow-up from time of expander placement	Did not attempt to differentiate between ADM types	Not reported	1297 pts: 655 ADM, 642 no ADM	Tissue expander for immediate unilateral or bilateral reconstruction after mastectomy for breast cancer or prophylaxis (14.0% vs. 6.7%)	Most surgeons either used ADM in most pts (49.6% of surgeons) or rarely used ADM (25.8% of surgeons)	Overall complications, OR=1.21, p=0.263; major complications OR=1.43, p=0.052; wound infections OR=1.49, p=0.118, reconstructive failure OR=1.55, p=0.089 at 2 years after reconstruction<br>PRO with BREAST-Q: no difference in Satisfaction with Breasts, Psychosocial Well-Being, Sexual Well-Being, Physical Well-Being, and Postoperative Pain.	Trend but not significant towards higher risks with ADM for major complication and failure
Ganesh Kumar, 2021 [70]	MROC study<br>10 centres in USA and 1 in Canada, 58 plastic surgeons	2012-2015	Prospective<br>Multivariable analysis for interaction of moderators and ADM for complications, wound infection	MROC did not record implant pocket type as a variable	Not reported	1451 pts: 738 with ADM, 713 no ADM	Immediate expander-implant-based reconstruction<br>DTI group small (102 ADM and 9 without ADM) and was excluded<br>ADM use determined primarily by surgeon preference and practice patterns<br>Data not collected on RT technique, implant/expander volumes, intraoperative details		There was an interaction between ADM and BMI for complications<br>No significant ADM effect (measured with BREAST-Q) on Satisfaction with Breast (ß= −1.95, p=0.20), Psychosocial Well-Being (ß= −0.26, p=0.85), Sexual Well-Being (ß= −2.28, p=0.18), or Physical Well-Being (ß= −0.82,	

Citation	Study name and location	Years of study*	Design	Type of ADM or mesh	Plane of implant, mesh location	Number of patients	Population	Other details	Outcomes	Notes
							40% drop rate (dropout rate?) over 2 years of study		p=0.46) 2 years postoperatively	
Pannucci, 2013 [267]	ASPS-TOPS database, USA	2008-2011	Multivariable logistic regression	CPT codes 15330 or 15331 (ADM but not specified)	Available in <30% of cases and therefore not used	14,249 pts: ADM 3,450 pts<br>8,746 pts had complete data and included in regression (2,905 with ADM)	Expander/implant-based reconstruction, immediate or delayed; excluded mastopexy/augmentation pts		30-day rates of tissue expander or implant loss 2.58% vs. 1.88%, use of ADM associated with increase in expander/implant loss, OR=1.42, 95% CI=1.04-1.94, p=0.026	Note that 30 days is too short for several outcomes<br>Authors indicate that clinically trivial results may exhibit statistical significance, and that ADM may improve surgeons' ability to perform breast reconstruction
Kilmer, 2024 [268]	PearlDiver, a national (USA) insurance-based database	2011-2019	Query of CPT codes for mastectomy and immediate implant or expander<br>Proportion matching for age, region, comorbidities<br>Complications within 90 days by univariate and multivariate analysis	ADM vs. none, type not reported	Not reported	26,266 ADM vs. 23,100 non-ADM	Pts with mastectomy in insurance database<br>CPT codes for mastectomy and immediate expander or implant, with or without same-day ADM<br>Exclude flaps, pts without insurance	Insurance databased does not have surgical details, implant placement, skin thickness or quality, ADM placement, ADM type, incision type	Infection 4.7% vs. 4.4%, p=0.178<br>Seroma 3.9% vs. 4.0%, p=0.520<br>Implant removal 4.9% vs. 3.9%, p<0.001<br>Explantation in direct-to-implant 8.2% vs. 6.3%, p=0.02<br>• Multivariate analysis for factors in implant removal: ADM OR=1.22, p<0.001<br>• Direct-to-implant OR=2.00<br>• COPD, depression, diabetes, hypertension, obesity, tobacco us, coronary artery	Risk factors for implant removal were tobacco use, hypertension, depression, obesity, ADM use, direct-to-implant surgery

Citation	Study name and location	Years of study*	Design	Type of ADM or mesh	Plane of implant, mesh location	Number of patients	Population	Other details	Outcomes	Notes
									disease all had OR between 1.19 and 1.26 (similar to ADM impact)	
Lin, 2024 [191]<br><br>Liston, 2024 [192]<br><br>See also Shanno, 2024 [193]	Massachusetts General Hospital, Harvard Medical School, Boston, MA, USA	2007-2019	Single institution, retrospective<br><br>At least 2 years follow-up post-operatively<br><br>NSM complications<br><br>Expander vs. direct-to-implant	AlloDerm (most common), FlexHD, Vicryl, Vicryl/ADM hybrid, SurgiMend	Not reported	1705 pts and 3035 breasts: number with ADM not reported	NSM and implant-based reconstruction<br><br>Excluded delayed or autologous reconstruction<br><br>Most operations by 3 breast surgeons and plastic surgeons<br><br>Surgeons chose plane of reconstruction and type of ADM/mesh based on experience or preference, pt characteristics, and treatment plan<br><br>Mostly subpectoral dual-plane or prepectoral, but also total muscle coverage; these are not mentioned as factors in the multivariable analysis		ADM or mesh vs. muscle only complications:<br><br>• Overall 9.06% vs. 10.33%; multivariate OR=0.749 (95% CI=0.404-1.391, p=0.361)<br>• Nipple necrosis 1.07% vs. 2.89%, p<0.05; multivariate OR=1.087 (95% CI=0.346-3.415, p=0.886)<br><br>Without multivariable analysis:<br><br>• Skin flap necrosis 3.44% vs. 5.37%<br>• Infection 3.26% vs. 3.72%<br>• Explantation 4.15% vs. 3.31%<br>• Hematoma 1.32% vs. 2.89%, p<0.05<br>• Seroma 1.00% vs. 1.65%<br>• Ruptured prosthesis 0.61% vs. 0.41%	Complications, AlloDerm vs. Flex HD vs. Vicryl (or Vicryl hybrid) [estimated from graphs]<br><br>Overall: 8.3% vs. 10.2% vs. 11.8%<br><br>Skin flap necrosis; 2.5% vs. 5.8% vs. 5.8%<br><br>Infection 3.1% vs. 2.4% vs. 4.5%<br><br>Explantation 3.4% vs. 6.3% vs. 6.3%<br><br>Rates of nipple necrosis, hematoma, seroma, ruptured prosthesis similar between groups

Citation	Study name and location	Years of study*	Design	Type of ADM or mesh	Plane of implant, mesh location	Number of patients	Population	Other details	Outcomes	Notes
									• Reconstruction failure 2.86% vs. 3.31%	
Plotsker, 2024 [269]	Memorial Sloan Kettering Cancer Center, New York, NY	2018-2021	Retrospective<br>Post hoc power analysis, 28.5% for expander loss with and without ADM; 66% power to detect difference in overall complications<br>Logistic regression	ADM, type not stated	Prepectoral	741 pts, 1225 breasts: 643 pts ADM, 98 pts no ADM; 1060 breasts ADM, 165 breasts no ADM	Tissue expander-based reconstruction in prepectoral plane<br>Exclude if direct-to-implant<br>ADM decided on basis of quality and thickness of mastectomy flap or at surgeon's discretion<br>Hypertension 18.4% vs. 29.6%; cardiovascular diseases 14.5% vs. 27.6%; SPY angiography 54.8% vs. 23.5%; NSM 21.2% vs. 11.2%, mean mastectomy weight 540.8 g vs. 750.3 g<br>Horizontal incision except 5 Wise pattern without ADM	Not reported	• Tissue expander loss 3.8% vs. 6.7%, OR=0.55 (95% CI= 0.27-1.22, p=0.095); multivariate OR=0.73 (95% CI=0.358-1.523, p=0.413)<br>• Any complication 20.8% vs. 22.5%, OR=0.92 (95% CI=0.61-1.41, p=0.683)<br>• TE exposure 1.8% vs. 4.2%, OR=0.42 (95% CI=0.16-1.19, p=0.073)<br>• Infection/cellulitis 7.6% vs. 12.1%, OR=0.61 (95% CI=0.35-1.08, p=0.067)<br>• Full-thickness mastectomy flap necrosis 2.6% vs. 1.2%, OR=2.23 (95% CI=0.55-19.46, p=0.416)<br>Seroma 8.6% vs. 9.1%, OR=0.95 (95% CI=0.53-1.81, p=0.882)	Expander loss might be clinically significant, need larger sample size as study was underpowered<br>Study suggests ADM may not be necessary in some patients with prepectoral expanders

Citation	Study name and location	Years of study*	Design	Type of ADM or mesh	Plane of implant, mesh location	Number of patients	Population	Other details	Outcomes	Notes
ACS-NSQIP database ADM type not specified)										
Davila, 2013 [270]	ACS-NSQIP database (250 sites)<br>Northwestern University, Feinberg School of Medicine, Chicago, IL, USA	2006-2010	Retrospective<br>Multivariate analysis	Any ADM; used ICD and CPT codes	ADM-assisted vs. submuscular (authors define as partial or total pectoralis and/or serratus muscular coverage without ADM)	12,249: 1717 ADM and 7442 without ADM	Total mastectomy and tissue expander simultaneously (immediate)	Not reported	Total complications 5.5% vs. 5.3%, p=0.68; multivariate 5.6% vs. 5.3%, OR=1.07 (95% CI=0.85-1.35), p=0.57<br>Infections 3.8% vs. 3.3%, p=0.27; multivariate 3.9% vs. 3.3%, OR=1.20 (95% CI=0.91-1.60), p=0.20<br>Prosthesis failure 1.0% vs. 0.8%, p=0.30; multivariate 1.1% vs. 0.8%, OR=1.39 (95% CI=0.82-2.37), p=0.23<br>Reoperation within 30 days: 6.9% vs. 6.9%, p=1.00; multivariate 7.0% vs. 6.9%, OR=1.01 (95% CI=0.82-1.25), p=0.91	
Winocour, 2015 [271]	ACS-NSQIP database<br>Mayo Clinic, Rochester, MN	2005-2011	Retrospective<br>Multivariable analysis<br>Focused on surgical site infections		Not reported	12,163 pts: 1890 ADM, 10,273 no ADM	Mastectomy as primary procedure and with concurrent (immediate) tissue expander (CPT 19357); ADM (CPT 15330) or no ADM	Not reported	Focused on surgical site infections: 4.5% vs. 3.2%, p=0.005; multivariable analysis OR=1.2, 95% CI=0.9-1.5, p=0.26	
Luo, 2022 [272]<br>Wells, 2022 [11]	ACPS-NSQIP database<br>University of Utah School of Medicine, Salt	2012-2019	Retrospective<br>Multivariable modified Poisson regression for	ADM vs. non-ADM	Not reported	49,049 cases<br>20,776 (42.4%) ADM, 28,273	Tissue expander (CPT 19357); immediate if expander and mastectomy codes together, otherwise considered delayed	Not reported	Surgical site infection 3.9% vs. 3.4%, p=0.003; multivariate RR=1.10 (1.01-1.21), p=0.03	Notes that database does not record ADM type; plane of ADM (complete

Citation	Study name and location	Years of study*	Design	Type of ADM or mesh	Plane of implant, mesh location	Number of patients	Population	Other details	Outcomes	Notes
[background to database]	Lake City, Utah		surgical site infection, dehiscence, reoperation			(57.6%) no ADM	Excluded BMI outside of 1.5 interquartile range (18.2 to 35.0 kg/m^2); or if missing data from ASA classification, height, weight, operating time		Reoperation 7.4% vs. 6.0%, p<0.001; multivariate RR=1.15 (1.08-1.23), p<0.001<br><br>Dehiscence 0.7% vs. 0.7%, p=0.73; multivariate RR=1.02 (0.82-1.27), p=0.86	submuscular, subpectoral, dual-plane); modifications; surgical technique; does not measure outcomes specific to breast reconstruction such as seroma, flap necrosis, reconstructive failure, capsular contracture; tracks outcomes for only 30 days<br><br>Wells, 2022 [11] also indicates it does not include plastic surgery-specific variables; does not specify type of ADM, type of expander, anatomic location of expander (prepectoral, subpectoral), antibiotics, preoperative RT, chemotherapy; outcomes limited to 30 days after surgery (cannot measure effectiveness such as aesthetics, pt satisfaction, time to exchange,

Citation	Study name and location	Years of study*	Design	Type of ADM or mesh	Plane of implant, mesh location	Number of patients	Population	Other details	Outcomes	Notes
										capsular contraction)
Graziano, 2024 [273]	ACPS-NSQIP database<br>Memorial Sloan Kettering Cancer Center	2015-2020	Retrospective; logistic regression for complications	Not available	ADM use increased over time: 44.2% in 2015 and 65.8% in 2020	22,087 ADM<br>17,713 no ADM	Immediate breast reconstruction; CPT codes for direct to implant reconstruction, or immediate or delayed 2-stage implant-based reconstruction or autologous immediate breast reconstruction; plus codes for ADM<br>Only codes for simple, modified radical, or radical mastectomy		30-day complications (not multivariate analysis)<br>• Overall complications 9.8% vs. 9.3%, p=0.072<br>• Surgical complications 9.3% vs. 8.6%, p=0.027 (including deep infections, 0.6% vs. 0.7% and superficial infection 1.9% vs. 1.6%)<br>• Major medical complications 1.1% vs. 1.1%, p=0.810	On multivariate analysis, ADM use had association with superficial wound infection, OR=0.997, p=0.017
AlloDerm vs. none										
Nahabedian, 2009 [274]	Georgetown University Hospital, Washington, DC<br>8 breast surgeons, 1 reconstructive surgeon, 2 institutions	2006-2008	(unclear whether prospective or retrospective)<br>Mean follow-up 17 months	AlloDerm vs. none	Dual-plane with upper 2/3 under pectoralis major muscle and lower 1/3 under AlloDerm or mastectomy skin flap	361 pts (476 breasts): 76 pts (100 breasts) with ADM and 285 pts (376 breasts) without ADM	In AlloDerm group, 60 immediate, 7 delayed, 6 revision, 3 revision augmentation<br>Expanders generally filled 40%-70% capacity with AlloDerm and 10%-20% capacity without AlloDerm; determined by contact point between upper and lower mastectomy skin flaps to ensure no undue tension<br>Cancer in 66/100 breasts with ADM		Infections 5.85% vs. 5.0%	33% breasts not cancer in ADM group, no details of non-ADM group<br>Calculated 24% contralateral

Citation	Study name and location	Years of study*	Design	Type of ADM or mesh	Plane of implant, mesh location	Number of patients	Population	Other details	Outcomes	Notes
Sbitany, 2009 [275]	Rochester, New York<br>2 reconstructive surgeons at different practices	2004-2007	Retrospective	AlloDerm	ADM partial subpectoral (extension of pectoralis muscle), one author<br>No ADM submuscular (under pectoralis major and serratus anterior) one author	100 pts (172 expanders): 50 ADM, 50 no ADM	100 consecutive expander reconstructions by 2 surgeons<br>In submuscular group, at exchange a capsulotomy performed along inferior third allowing IMF to descend		Mean expander size 482 mL vs. 393 mL, p=0.002<br>Intraoperative fill volume 412 mL vs. 130 mL, p=0.0001<br>Mean number of fills 1.72 vs. 4.31, p=0.0001<br>Complications 9 vs. 7, p=0.79	Calculated 42% contralateral; no mention of whether pts have cancer<br>Pt groups similar; differences in age, BMI, smoking, PMRT but not statistically significant as only 50 pts/group
Chun, 2010 [276]	Brigham and Women's Hospital/ Faulkner Hospital, Boston, Mass.<br>21 mastectomy surgeons, 7 plastic and reconstructive surgeons	2002-2008	Retrospective	AlloDerm	ADM: released inferior origin of pectoralis major muscle and inset ADM between IMF and inferior border of pectoralis major<br>No ADM: either total submuscular coverage or partial submuscular and subcutaneous tissue coverage	283 pts (415 breasts): 269 ADM, 146 no ADM	Expander or implants for immediate reconstruction<br>Autologous flap included if used in conjunction with tissue expander or implant (68 latissimus dorsi flaps and 1 pedicled transverse rectus abdominis flap); this was 3.3% of ADM group and 41% of non-ADM group	Pts similar for age, smoking RT, cancer stage<br>BMI and mastectomy specimen weight higher in ADM group<br>Diabetes (3.7% vs. 0.68%) and hypertension (12.6% vs. 0.69%) higher in ADM group but not included in multivariate analysis	Seroma 14.1% vs. 2.7%; OR=4.24, 95% CI=1.28-14.0, p=0.018<br>Necrosis 23.4% vs. 8.9%<br>Infection 8.9% vs. 2.1%; OR=5.37, 95% CI=1.63-17.6, p=0.006<br>Mean implant volume 459.3 mL vs. 340.8 mL, p<0.001<br>Mean intraoperative fill volume 322.7 mL vs. 131.2 mL, p<0.0001<br>Analysis excluding flap reconstructions: seroma 13.9% vs. 2.4%, p=0.0038; major infection 8.5% vs. 1.2%, p=0.0199	32% of mastectomies were contralateral<br>Discussion indicates BMI and preoperative RT used in multivariate analysis; other factors may be needed<br>Subsequent modification in technique resulted in lower seroma rates

Citation	Study name and location	Years of study*	Design	Type of ADM or mesh	Plane of implant, mesh location	Number of patients	Population	Other details	Outcomes	Notes
Ganske, 2013 [277]	Brigham and Women's Hospital/ Faulkner Hospital, Boston, Mass.	2008-2010<br>2002-2008 prior to modifications	Retrospective<br>Results after implementing modifications to reduce seroma<br>Univariate analysis	AlloDerm	See Chun, 2010 [276]: inserted expander under pectoralis major muscle, lower portion covered with ADM Without ADM: either total submuscular coverage or partial submuscular and subcutaneous tissue coverage	179 after seroma-reducing modification: 106 ADM and 73 no ADM<br>150 pts (64 ADM and 86 no ADM) before modification (see Chun, 2010 [276]) but including only YC's pts	Changes were drainage of submastectomy skin flap pocket and sub-ADM pocket, addition of soft compression dressings and surgical bras<br>Bilateral mastectomies and reconstructions considered as 2 cases and statistics were adjusted for clustering	Age, BMI, and mastectomy specimen weight higher in ADM group	No multivariate analysis:<br>Seroma 4.7% vs. 1.4%, p=0.2277<br>Infection 3.8% vs. 0<br>Skin flap necrosis 28.3% vs. 5.5%, p=0.0003<br>Mean intraoperative fill volume 298.1 mL vs. 96.5mL, p<0.001	Rates of seroma and infection significantly lower than earlier study<br>Necrosis still very high with ADM
Liu, 2011 [278]	Brigham and Women's Hospital, Boston, Mass.	2004-2009	Retrospective<br>Multivariate analysis for overall complications, infection<br>At least 3 months follow-up	AlloDerm	ADM: implant under pectoralis major muscle and inferior (lower) portion subcutaneous with ADM<br>No ADM: completely submuscular or dual-plane	343 pts (470 breasts): 192 pts (266 breasts) with ADM, 151 pts (204 breasts) without ADM	Consecutive immediate prosthetic reconstruction<br>Mostly expander used, though in small portion of ADM group it was direct to implant<br>Excluded delayed or autologous flap reconstructions<br>ADM group had higher mean mastectomy weight (526 g vs. 457 g), higher initial volume (implant or expander; 188 mL v 75 mL), larger final implants (434 mL vs. 356 mL), more smokers (11.5% vs. 5%)<br>27% of mastectomies were contralateral	Authors indicate groups well-matched in RT, BMI, comorbidities	Surgical complications 19.5% vs. 12.3%, p=0.034; multivariate OR=1.76 (95% CI=1.03-3.01), p=0.036<br>Infection 6.8% vs. 2.5%, p=0.031; multivariate OR=3.25 (95% CI=0.80-13.12), p=0.097<br>Major skin necrosis 11.7% vs. 8.3%, p=0.282<br>Any skin necrosis 13.9% vs. 10.8%, p=0.310<br>Seroma 7.1% vs. 3.9%, p=0.136	

Citation	Study name and location	Years of study*	Design	Type of ADM or mesh	Plane of implant, mesh location	Number of patients	Population	Other details	Outcomes	Notes
Vardanian, 2011 [279]	University of California, Los Angeles<br>Single academic medical centre<br>4 primary surgeons performed reconstruction s	2000-2008: ADM starting July 2004	Retrospective<br>Multivariate logistic regression<br>Same surgeons in both cohorts	AlloDerm	ADM: elevated pectoralis major, insert ADM between muscle and IMF and insert expander in pocket<br>No ADM: total muscular or partial with creation of pocket deep to pectoralis major and serratus anterior muscles with external bolsters to cover inferolateral aspect of breast	203 pts (337 breasts): 123 pts (208 breasts) ADM, 80 pts (129 breasts) no ADM	Implant-based immediate reconstruction<br>Same surgeons operated on both cohorts (no ADM until June 2004; ADM starting July 2004)<br>Most non-ADM used partial muscle coverage<br>40% of mastectomies were contralateral<br>93.5% vs. 83.7% of pts had cancer	Multivariate logistic regression adjusted for clinical characteristics (BMI, smoking, indication for reconstruction) and postoperative complications (infection, seroma, inframammary fold problems, capsular contracture, mechanical shift, bottoming-out, rippling, and wound problems)	Complications: 29.3% vs. 40.3%, univariate OR=0.61, 95% CI=0.38-0.97, p=0.038<br>Capsular contracture: 3.8% vs. 19.4%, univariate OR=0.16; multivariate OR=0.18, 95% C=0.08-0.43<br>IMF problems: 8.2% vs. 19.4%, univariate OR=0.37; multivariate OR=0.49, 95% CI=0.23-1.01, p=0.055<br>Mechanical shift: 1.9% vs. 9.3%, univariate OR=0.19; multivariate OR=0.23, 95% CI=0.06-0.78<br>Seroma/hematoma 2.4% vs. 1.6%<br>Infections 1% vs. 2.3%<br>Wound problems 1% vs. 0%<br>Dehiscence 1% vs. 0%<br>Skin thinning 1% vs. 3.1%<br>Aesthetics on 4-point scale, with 4 being excellent: Overall	

Citation	Study name and location	Years of study*	Design	Type of ADM or mesh	Plane of implant, mesh location	Number of patients	Population	Other details	Outcomes	Notes
									aesthetics 3.26 vs. 2.87, p<0.05; IMF outcome 3.35 vs. 2.94, p<0.05	
McCarthy, 2012 [280]	NCT00639106<br>Memorial Sloan-Kettering Cancer Center, New York, NY<br>University of North Carolina at Chapel Hill, Chapel Hill, N.C	2008-2011	RCT, terminated early<br>Primary outcome of postoperative pain	AlloDerm	ADM: inferior-lateral portion of pocket created using ADM, neither serratus muscle /fascia nor rectus abdominis fascia elevated<br>No ADM: submuscular pocket involving serratus muscle/fascia and rectus abdominis fascia	49 ADM, 49 no ADM planned; actual 70 pts total	Age ≥21 years, immediate expander/implant reconstruction<br>Exclude if prior RT or ALND, significant mastectomy flap ischemia or ALND at time of mastectomy	Difference of 2 points on 1-10 scale (20 points on 1-100 scale) considered clinically significant	Pain at baseline 7 vs. 1.4; removed 3 pts with unusually high baseline pain<br>Pain in 24h postoperative period 54.6 vs. 42.8, p=0.19<br>Pain in expansion phase 17.0 vs. 4.6, p=0.65<br>Pain at completion of expansion 5.6 vs. 4.6, p=0.93<br>Patient-reported Physical Well-Being (Chest and upper Body scales of BREAST-Q) were not different: 85.6 vs. 86.9 baseline; 65.8 vs. 68.2 immediate 24-hour postoperative; 68.6 vs. 69.3 expansion phase; 79.7 vs. 80.5 completion of expansion<br>No difference in narcotic use, p=0.38<br>Long-term aesthetic outcome, rate of capsular contracture,	Trial closed early at interim analysis due to slow accrual with 65 pts randomized; probability of obtaining positive result for primary endpoints was at most 11% for immediate postoperative pain and <1% for pain at expansion<br>ADM did not reduce pain, but numbers are small<br>Pain may be due to mastectomy process and not reconstruction; or may be due to sutures during reconstruction

Citation	Study name and location	Years of study*	Design	Type of ADM or mesh	Plane of implant, mesh location	Number of patients	Population	Other details	Outcomes	Notes
									patient satisfaction, and QoL (Phase 2, will be separate publication)	
Parks, 2012 [281]	Metropolitan private practice group, Memphis, Tenn.<br>5 breast surgeons, 3 plastic surgeons	2001-2011; ADM 2005-2010; no ADM 2001-2005	Retrospective<br>Logistic regression model for tissue expander loss	AlloDerm	Subpectoral pocket with ADM sewn to pectoralis and IMF<br>No ADM: submuscular	346 pts (511 breasts): 232 pts (346 breasts ADM and 114 pts (165 breasts) without ADM	SSM or modified SSM, immediate reconstruction, tissue expander<br>Cancer or prophylaxis<br>32% of mastectomies were contralateral	Expander loss was a bright-line definition of infection	ADM was not a risk factor for expander loss: 11.6% vs. 8.5%; logistic regression OR=0.84 (95% CI=0.38-1.88), p=0.677<br>Seroma 30.0 vs. 15.7%, p<0.001<br>Skin necrosis 11.9% vs. 11.5%, p=0.88	
Warren Peled, 2012b [105]	University of California, San Francisco<br>4 breast surgeons, 1 plastic surgeon	2006-2010	Prospective<br>Mean follow-up 25.5 months<br>Logistic regression for complications in cases with PMRT; ORs adjusted for age and BMI using logistic model [note less than 25 pts per group for RT subsets]<br>No correction for comparison	AlloDerm	Inferior aspect of pectoralis major is left intact at inferior origin superior to IMF; ADM sutured inferiorly and laterally to chest wall at inferolateral aspect of expander to complete the pocket for expander<br>No ADM: expander placed in subpectoral pocket without added coverage of	450 cases in 288 pts<br>Cohort 1: 90 cases, years 2006-2007, no ADM<br>Cohort 2: 100 cases (65 pts), years 2007-2008, all used ADM<br>Cohort 3: 260 cases (160 pts), years 2008-2010, selective ADM	NSM (inversion and complete excision of nipple core at dermal junction) and immediate expander-implant reconstruction; earlier studies had found lower ischemic complications with 2-stage procedures and using initial minimal expansion<br>Excluded pts with clinical involvement of NAC or skin or with significant breast ptosis<br>Cohort 1: consecutive cases without ADM<br>Cohort 2: the next consecutive cases with ADM<br>Cohort 3: selective ADM in next 250 cases based on	By time of this study, technical refinements had reduced NAC complications to <5%<br>Use of PMRT was significantly different (23.3% vs. 14% vs. 12.7%) and PMRT was shown to have negative effect on infection, return to OR and expander-implant loss<br>The group without ADM had	<u>No multivariate analysis</u><br>Infection (requiring oral or iv antibiotics): 15.8% selective ADM; 20% consecutive ADM; 27.8% no ADM. p=0.04<br>Unplanned return to operating room: 10% selective ADM; 11% consecutive ADM; 23.3% no ADM, p=0.004<br>Expander-implant loss 5% selective ADM; 7% consecutive ADM; 17.8% no ADM, p=0.001	Concluded that ADM reduced complications; maximum benefit in selected pts with thin mastectomy skin flaps and in pts with PMRT<br>The risk of infections, unplanned return to OR and expander-implant loss was higher in group with selective ADM than with consecutive ADM, suggesting ADM should be used in

Citation	Study name and location	Years of study*	Design	Type of ADM or mesh	Plane of implant, mesh location	Number of patients	Population	Other details	Outcomes	Notes
			between groups		inferolateral expander		mastectomy skin flap thickness (ADM used in thinner skin flaps)<br>PMRT: 12.7% selective ADM, 14% consecutive ADM, 23.3% no ADM<br>40.7% prophylactic (41.5% selective ADM, 45% consecutive ADM, 33.3% no ADM)	more neoadjuvant chemotherapy, more therapeutic mastectomy, more ALND<br>Downward trend with time could have been partially related to other improvements and not just ADM	Skin flap necrosis: 6.2% selective ADM, 6% consecutive ADM, 11.1% no ADM, p=0.26<br>Seroma 5.8% vs. 4% vs. 4.4%<br>Hematoma 2.7% vs. 3% vs. 3.3%<br>Nipple necrosis 1.2% vs. 1% vs. 0<br>PMRT increased risk of infection (OR= 2.35 selective and OR=2.08 consecutive), return to OR (OR= 5.75 and 1.69) and expander-implant loss (OR=6.07 and 4.87	pts who are likely to need PMRT<br>No explanation of why ADM decreased infection, and selective ADM had bigger increase than use in all pts
Weichman, 2012 [282]	New York University Langone Medical Center<br>Single institution<br>7 ablative surgeons and 5 reconstructive surgeon	2007-2010	Retrospective<br>Multivariate logistic regression but results not reported	AlloDerm	All used pectoralis major muscle for coverage<br>ADM: implant covered superiorly with pectoralis and inferior-laterally with ADM sling<br>No ADM: implant total submuscular position with serratus muscle flap and rectus fascia if needed	407 pts (628 breasts): 442 ADM, 186 no ADM	Immediate 2-stage expander-implant<br>No criteria for use of ADM<br>Prophylactic 44.3% vs. 36.0%<br>35% of mastectomies were contralateral	Expander size 408 mL vs. 385 mL, p=0.054<br>Expander fill 169 vs. 135 mL, p=0.0013	<u>No multivariate analysis</u><br>Major complications 15.3% vs. 5.4%, p=0.001<br>Infections requiring intravenous antibiotics 8.6% vs. 2.7%, p=0.001; any infection 13.6% vs. 7.5%, p=0.017<br>Flap necrosis requiring excision 6.7% vs. 2.7%, p=0.015; any flap	ADM results in more complications and should be used selectively

Citation	Study name and location	Years of study*	Design	Type of ADM or mesh	Plane of implant, mesh location	Number of patients	Population	Other details	Outcomes	Notes
									necrosis 8.3% vs. 3.2%, p=0.005<br>Explantation of tissue expander 7.7% vs. 2.7%, p=0.004<br>Seroma 1.8% vs. 3.2%, p=0.326<br>Hematoma 0.5% vs. 1.1%, p=0.586	
AlloDerm (various forms) vs. none										
Weichman, 2013 [283]	New York University Langone Medical Center<br>5 reconstructive surgeons	2010-2012<br>AlloDerm RTU Nov 2011-Oct 2012	Prospective cohort study<br>Univariate logistic analysis	AlloDerm RTU vs. none	Dual-plane with ADM vs. submuscular	546 breasts: 105 AlloDerm RTU, 351 no ADM	All pts undergoing immediate implant-based reconstruction, either with expander or direct implant<br>Indications for ADM use included lack of muscular coverage, cancer invasion to pectoralis major muscle, immediate implant reconstruction; relative indications included NSM, prior submuscular augmentation<br>AlloDerm RTU vs. none<br>Specimen weight 587 g vs. 514 g, p=0.0746; 1-stage implant 15.2% vs. 0.8%, p=0.0001; NSM 49.2% vs. 31.9%, p=0.0012; ALND 24.8% vs. 14.5%, p=0.0141; expander size 440 vs. 397 mL, p=0.002; fill volume 206 vs. 148 mL, p=0.001; % fill 46.7% vs. 36.6%, p=0.0001	ADM: release of pectoralis major from IMF, addition of ADM as a sling<br>Submuscular (no ADM) by elevating pectoralis major muscle, serratus anterior muscle, and partial rectus fascia	AlloDerm RTU vs. none<br>Flap necrosis 10.4% vs. 5.1%, p=0.0658<br>Major flap necrosis 5.7% vs. 3.4%, p=0.2678<br>Infection 8.5% vs. 5.7%, p=0.3602<br>Cellulitis (deep infection) requiring IV antibiotics 4.7% vs. 1.4%, p=0.0551	

*Year of diagnosis or initial surgery
**Studies only indicated use of ADM but not specific type. Studies in the United States have been included based on primarily human ADM being used in Canada and USA (although Strattice is also common in US), but studies in Europe excluded as non-human ADM is more common

Abbreviations:
ACS-NSQIP, American College of Surgeons National Surgical Quality Improvement Program; ACPS-TOPS, American Society of Plastic Surgeons Tracking Outcomes and Operations in Plastic Surgery database; ADM, acellular dermal matrix; ASA status, American Society of Anesthesiologists physical status classification system; BRCA, breast cancer gene; BMI, body mass index; CI, confidence interval; COPD, chronic obstructive pulmonary disease; CPT, Current Procedural Terminology codes (USA); DTI, direct to implant; IMF, inframammary fold; ICD, International Classification of Diseases; MROC, Mastectomy Reconstruction Outcomes Consortium Study; NAC, nipple areolar complex; NACT, neoadjuvant chemotherapy; NSM, nipple-sparing mastectomy; OR, odds ratio; PMRT, postmastectomy radiotherapy; PRO, patient reported outcomes; pts, patients; RR, relative risk; RT, radiotherapy; RTU, ready to use; SSM, skin-sparing mastectomy; SLND, sentinel lymph node dissection

Back to Start of Tables

Table S13. Question 5: Acellular Dermal Matrix. B. Comparison of ADMs

Citation	Study name and location	Years of study*	Design	Type of ADM or mesh	Plane of Implant	Number of patients	Population	Surgical details	Outcomes	Other
Arnaout, 2021 [284]<br>Stein, 2021 [285]	NCT03064893<br>REaCT investigators<br>Ottawa Hospital, Ottawa<br>3 oncologic and 3 reconstructive surgeons	2016-2018	RCT, randomized at the pt level using a web-based program and permuted variable block design, no stratification<br>If bilateral mastectomy, they received same type of ADM in both breasts	AlloDerm RTU vs. DermACELL	Subpectoral (dual-plane)	62 pts randomized (31 per group); 81 mastectomies (41 AlloDerm RTU, 40 DermACELL)	Immediate subpectoral reconstruction with permanent implant<br>ReACT prospective pragmatic clinical trial methodology<br>ADM was non-fenestrated<br>Excluded tissue expander, excluded prepectoral reconstruction<br>51.3% NSM (55.3% vs. 47.5%), 48.7% SSM (44.7% vs. 52.5%<br>Mastectomy weight 600 g vs. 387 g<br>ADM prepared according to manufacturer instructions: AlloDerm RTU soaked at least 2 minutes in sterile saline or lactated Ringer solution 2 times; DermACELL was ready for use from the package<br>32% prophylactic mastectomies	Subpectoral (dual-plane): implant pocket created by elevation of pectoralis major muscle, ADM anchored to IMF and inferior part of the lateral boundary along the anterior maxillary line<br>Implant placed under pectoralis major muscle and free muscle edge secured to ADM<br>2 closed suction drains kept in place until output <20 mL per 24 h; maximum 14 days<br>IV antibiotics upon induction of anesthesia; oral antibiotics for 1 week after surgery	Primary outcome:<br>•Seroma (assessed by drain duration), 10.8 d vs. 9.2 d, p=0.15<br>Secondary outcomes:<br>Major complications<br>•Unplanned return to operating room 15.8% vs. 7.5%, p=0.28<br>•Loss of implant 5.3% vs. 5.0%, p=0.96<br>Minor complications<br>•Seroma after drain removal 5.3% vs. 12.5%, p=0.28<br>•Infection requiring antibiotics 7.9% vs. 2.5%, p=0.32<br>• Red breast syndrome 2.6% vs. 2.5%, p=0.97<br>•Wound dehiscence 7.9% vs. 5.0%, p=0.68<br>•Hematoma 5.3% vs. 0, p=0.23<br>•Skin necrosis 5.3% vs. 10.0%, p=0.77<br>•Capsular contracture 2.6% vs. 0<br>Baseline BREAST-Q scores similar; at 3 months the AlloDerm	Despite being RCT, due to small size the two arms have some differences e.g., mastectomy weight (600 g vs. 387 g), location of incision, smoking status (9.7% vs. 3.2%), heart disease (16.1% vs. 9.7%), breast size ≥D cup 12.9% vs. 22.6%, Ptosis grade III or more (0% vs. 10.0%)<br>Multivariate controlling for preoperative Psychosocial Well-Being, Satisfaction with Breasts, Physical, and Sexual Well-Being found same results

Citation	Study name and location	Years of study*	Design	Type of ADM or mesh	Plane of Implant	Number of patients	Population	Surgical details	Outcomes	Other
									group had higher satisfaction:<br>•Satisfaction with Breasts 66.6 vs. 52.5, p=0.03<br>•Overall Satisfaction with Results 84.6% vs. 60.9%, p=0.003<br>•No significant difference in Psychosocial, Sexual, Physical Well-Being<br><br>At 12 months no significant differences in PROM (p>0.05)	
Chu, 2023 [286]	Memorial Sloan Kettering Cancer Center, New York, NY, USA	2018-2021	Retrospective<br>Multivariate logistic regression for impact of ADM type on tissue expander loss	726 pts: 115 AlloDerm, 57 FlexHD, (554 SurgiMend - outside scope of review)<br>Expanders: 194 AlloDerm, 93 FlexHD, 767 SurgiMend<br>Perforated: 94.8% AlloDerm 98.2% FlexHD, 100% SurgiMend	Prepectoral	726 pts, 1054 expanders	2-stage prepectoral reconstruction<br>Excluded direct-to-implant, subpectoral plane	ADM sutured to suture tabs then the chest wall	Complications in 90 days, AlloDerm vs. FlexHD<br>Seroma 11.9% vs. 4.3%<br>Infection 5.7% vs. 3.2%<br>Expander loss 4.1% vs. 3.2%; multivariate OR=1.37, p=0.658<br>Expander exposure 1.5% vs. 1.1%	
Berger, 2024 [287]	MedStar Georgetown University Hospital,	2014-2022	Retrospective<br>6 plastic surgeons	231 pts AlloDerm vs. 405 pts DermACELL	55.0% prepectoral but varied by ADM	738 pts, 1228 breasts	Patients with CPT code for biologic implant for breast or trunk		90-day complication rates are similar between groups	

Citation	Study name and location	Years of study*	Design	Type of ADM or mesh	Plane of Implant	Number of patients	Population	Surgical details	Outcomes	Other
	Washington, DC (4 hospitals within multicentre hospital)		Multivariate logistic regression model for postoperative outcomes	vs. 102 pts SurgiMend PRS<br>(351 vs. 712 vs. 165 breasts)	type: 47.6% vs. 58.6% vs. 55.8%		Immediate prosthesis-based breast reconstruction, either expander or direct-to-implant<br>Excluded if not breast reconstruction, was delayed, or if previous failed reconstruction<br>Surgery varied: NSM 41.9% vs. 72.1% vs. 47.3%		In multivariate analysis, the type of ADM was not independently predictive of any postoperative complication; authors used the reference to be SurgiMend so p values are not available for AlloDerm vs. DermaCell	
Johnson, 2022 [288]	University of Colorado, Aurora, Colorado<br>Single institution; 2 surgeons	2016-2020	Retrospective<br>Multiple logistic regression models for primary outcomes of seroma and infection	AlloDerm RTU vs. DermACELL	Subpectoral or prepectoral	150 pts (241 breasts): 88 pts (143 breasts) AlloDerm, 62 pts (98 breasts) DermACELL	AlloDerm RTU is a perforated sheet whereas DermACELL is not<br>AlloDerm RTU is partially terminally sterilized to 10^{-3}<br>ADM prepared according to manufacturer guidelines<br>Prepectoral 30.0% vs. 37.8%; subpectoral 70.0% vs. 62.2%<br>NSM 47.6% vs. 41.8%; SSM 52.4% vs. 58.1%<br>Immediate 2-stage reconstruction and had undergone at least first stage<br>38% contralateral mastectomy; 41% for prophylaxis	Subpectoral: pectoralis elevated and divided at inferomedial origin, ADM inset to IMF and lateral breast border<br>Prepectoral: pectoralis muscle not elevated; ADM placed in breast pocket and inset into superior, medial, and latera border with sutures; second sheet of ADM inset into IMF, tissue expander inserted and tabs secured to superficial chest wall	Seroma: univariate 21.7% vs. 8.2%, p=0.005; multivariate p=0.04 (95% CI=1.02-6.07) in abstract; p=0.01, 95% CI=1.29-6.95 in text<br>Surgical site infection: univariate 13.3% vs. 13.3%, p=0.99; multivariate not analyzed<br>BMI was associated with higher rate of infection	Slight variations of results in different tables and in text
Zenn, 2016 [289]	Duke University Medical Center	Not stated;	Retrospective<br>Minimum follow-up 6	AlloDerm RTU vs. DermACELL	Not reported	140 pts (249 implants)	Consecutive pts before and after switch from AlloDerm to		Complications low in both groups	

Citation	Study name and location	Years of study*	Design	Type of ADM or mesh	Plane of Implant	Number of patients	Population	Surgical details	Outcomes	Other
	and Mt. Sinai Health System<br><br>2 surgeons	≈2012-2015	months and ranged 6 months to 2 years			70 pts (130 implants) AlloDerm RTU, 70 pts (119 implants) DermACELL	DermACELL; no difference in techniques<br><br>No statistical difference in age, indication, RT, or chemotherapy<br><br>44% contralateral mastectomy (46% vs. 41%)		Seroma, none<br><br>Hematoma 0 vs. 1 (0.8%)<br><br>Infection 1 (0.8% vs. 2 (1.7%)	
Agarwal, 2015 [14]<br><br>Design and methods	BREASTrial; NCT00872859<br><br>University of Utah, Salt Lake City, Utah<br><br>Single centre, 1 reconstructive surgeon	2008-2011	RCT<br><br>2-year follow-up from definitive reconstructio n<br><br>Multivariate analysis to correct for differences in pt/disease characteristic in Stage I and III of the trial, but not in Stages II due to fewer pts and events [290-292]	AlloDerm vs. DermaMatrix	ADM as inferolateral sling of the breast pocket	128 pts randomized (199 breasts)<br><br>64 pts AlloDerm (101 breasts), 64 pts DermaMatrix (98 breasts)<br><br>Loss to follow-up 5 vs. 7 pts (7 vs. 10 breasts)	Immediate expander for breast cancer or BRCA1/BRCA2 mutations<br><br>Definitive reconstruction with prosthesis (implant) or autologous tissue after PMRT (41%) and chemotherapy (49%)<br><br>At least 12 weeks after last PMRT before definitive reconstruction; at least 3 weeks after chemotherapy before definitive reconstruction<br><br>Prophylactic (breast basis): 49% vs. 45%;<br><br>NSM 49% vs. 48%<br><br>Prophylactic (pt basis, no cancer): 21.9% vs. 17.2%	ADM prepared according to manufacturer instructions<br><br>Elevation of pectoralis major muscle, releasement of inferior attachments, placement of tissue expander, placement of ADM to constitute inferolateral pocket, placement of drains, inflation of expander filling as much dead space as possible without creating excessive tension on the skin	See other publications<br><br>•Stage 1 (from mastectomy to definitive reconstruction)<br>•Stage II (definitive reconstruction until 3 months<br>•Stage III (3 months to 2 years after definitive reconstruction)	Arms not equivalent: smokers 0% vs. 9.4%; chemotherapy 39.1% vs. 59.4%; PMRT 31.3% vs. 50.0%; ALND 26.6% vs. 39.1%; cancer stage III or IV 18.7% vs. 37.6%<br><br>In general, DermaMatrix arm had more advanced disease
Mendenhall, 2015 [290]<br><br>Stage I of trial	BREASTrial; NCT00872859<br><br>University of Utah, Salt Lake City, Utah	2008-2011	RCT<br><br>For stage I, variable significant on univariable analysis or clinically	AlloDerm vs. DermaMatrix		See above<br><br>Sample size too low as it is based on 2.5-fold difference in rate of grade 1-4	Both AlloDerm and DermaMatrix are decellularized, free-dried, aseptically processed, but not terminally sterile		Stage I results<br><br>Overall complications 33.6% vs. 38.8%, p=0.52; multivariate p=0.68	Multivariate analysis controlled only for obesity, RT, chemotherapy<br><br>Results appear to be per breast (not per patient);

Citation	Study name and location	Years of study*	Design	Type of ADM or mesh	Plane of Implant	Number of patients	Population	Surgical details	Outcomes	Other
			relevant were included in multivariable logistic regression model			complications when comparing arms and this is unrealistic			Major complications 13.8% vs. 21.4%<br><br>Skin necrosis 17.8% vs. 21.4%, p=0.66; multivariate p=0.74<br><br>Infection 13.9% vs. 16.3%, p=0.29; multivariate p=0.71<br><br>Seroma 6.1% vs. 3.1%, p=0.34<br><br>Hematoma 0 vs. 2.0%, p=0.24<br><br>Tissue expander loss 5% vs. 11.2%, p=0.11; too few events for multivariate analysis<br><br>Time for expansion 42 days vs. 70 days, p<0.001	cancer status/ indication not included in multivariate analysis
Mendenhall, 2017 [291]<br><br>Stage II of trial	BREASTrial; NCT00872859<br><br>University of Utah, Salt Lake City, Utah	2008-2011	RCT<br><br>Authors indicate there were too few pts and complications to allow multivariate analysis	AlloDerm vs. DermaMatrix		111 pts and 173 breasts were available for analysis in stage II of the trial			Stage II (0-3 months after definitive reconstruction)<br><br>Overall complications 15.4% vs. 18.3%, p=0.8<br><br>Major complications 5.5% vs. 9.6%, p=0.2<br><br>Infection 3.3% vs. 6.1%, p=0.3<br><br>Skin necrosis 2.2% vs. 3.7%, p=0.5<br><br>Implant loss 2.2% vs. 3.7%, p=0.5	DermaMatrix group had more adjuvant chemotherapy, PMRT, higher stage disease, autologous reconstruction

Citation	Study name and location	Years of study*	Design	Type of ADM or mesh	Plane of Implant	Number of patients	Population	Surgical details	Outcomes	Other
Mendenhall, 2023 [292]<br><br>Stage III of trial	BREASTrial; NCT00872859<br><br>University of Utah, Salt Lake City, Utah	2008-2011	RCT<br><br>Multivariable logistic regression for effect of matrix type, age, obesity, Radiation therapy, chemotherapy, type of reconstruction on complications	AlloDerm vs. DermaMatrix		108 pts (167 breasts available for analysis			Stage III (3 months to 2 years after definitive reconstruction)<br><br>Overall complications 6% vs. 13.2%, p=0.3; multivariate p=0.52<br><br>Patient satisfaction questionnaire not validated (was before BREAST-Q and BRECON-31 became popular)	Only 10 complications so too few events for multivariate analysis
Palaia, 2015 [293]	North Westchester Surgical Services group<br><br>2 oncologic breast surgeons, 3 reconstructive plastic surgeons<br><br>Single centre	2006-2011	Retrospective<br><br>Multivariate regression<br><br>Stratified by ADM type and fenestration status<br><br>Postoperative data for at least 6 months after last reconstruction or complication	AlloDerm (± fenestration) vs. FlexHD (± fenestration)	Not reported	450 pts, 603 breasts<br><br>134 pts (179 breasts) AlloDerm (103 pts fenestrated, 31 without)<br><br>316 pts (424 breasts) FlexHD (259 pts fenestrated, 57 without)	Immediate 2-stage reconstruction with ADM<br><br>6x16 cm ADM in all pts<br><br>AlloDerm rehydrated in saline ≥45 minutes; FlexHD is prehydrated<br><br>Fenestration by cutting through full thickness of ADM with an 11 or 15 scalpel set at intervals of ≈1 cm; decision to fenestrate at surgeon's discretion	Not reported	Per breast outcomes<br><br>AlloDerm vs. FlexHD:<br>• Seroma 14.0% vs. 12.3%, p=0.562; OR=0.84, p=0.7408<br>• Infection 11.2% vs. 9.2%, p=0.4558; OR=1.19, p=0.7877<br>• Extrusion 6.2% vs. 1.9%, p=0.0062; [multivariate OR=4.30, p=0.0031 but <25 events]<br>• Explantation 8.9% vs. 7.3%, p=0.4959; OR=1.25, p=0.7547<br><br>Fenestrated vs. not:<br>• Seroma 11.1% vs. 20.0%, p=0.0098; multivariate OR=0.34, p=0.0026;	Cosmetic score 8.7±1.5 AlloDerm vs. 8.4±1.7 FlexHD, p=0.0717; multivariate p=0.0466. It is unclear whether this small difference is meaningful to patients

Citation	Study name and location	Years of study*	Design	Type of ADM or mesh	Plane of Implant	Number of patients	Population	Surgical details	Outcomes	Other
									• No differences in infection, extrusion, explantation, cosmetic score	
Seth, 2013 [294]	Northwestern Memorial Hospital, Chicago, Illinois<br>2 plastic surgeons	2006-2011	Retrospective<br>Multiple regression<br>Mean follow-up 60.6 weeks	AlloDerm vs. FlexHD	Dual-plane	255 pts, 369 breasts<br>96 pts (136 breasts AlloDerm; 159 pts (233 breasts) FlexHD	All pts in which AlloDerm (cryopreserved) or FlexHD (prehydrated) ADM was used; choice of product based on surgeon preference and pt factors<br>Expander/implant reconstruction (4.4% and 6.4% autologous)<br>Significant differences in age, BMI, indication, chemotherapy, size of ADM, expander size and intraoperative fill volume	Lower border of pectoralis muscle disinserted with bovie electrocautery and ADM secured to lower pole defect, lower portion of ADM sutured to IMF and lateral aspect to serratus muscle fascia	Total complications 19.1% vs. 19.3%, p=1.00; multiple regression OR=0.99, 95% CI=0.58-1.69, p=1.00<br>Infection 10.3% vs. 5.2%, p=0.09; multivariate OR=2.11, 95% CI=0.95-4.71, p=0.09<br>Flap necrosis (8.1% vs. 9.0%, p=0.85; multiple regression OR=0.88, p=0.85)<br>No differences in univariate analysis (too few events multivariate) for expander migration (none), hematoma 2.9% vs. 1.3%, p=0.43), seroma (2.2% vs. 2.1%, p=1.00), exposure/dehiscence (5.9% vs. 6.4%, p=1.00)	P values are identical before and after multiple regression analysis
Liu, 2014 [295]	University of Washington School of Medicine, Seattle, Washington	2006-2011	Retrospective<br>Multivariate analysis; multiple logistic	AlloDerm vs. FlexHD vs. none	With ADM (not in methods, but background indicates ADM for	165 AlloDerm, 97 FlexHD, 177 no ADM	Consecutive pts with 1 or 2-stage immediate reconstruction<br>89.2% SSM, 4.3% NSM, 6.5% simple/total mastectomy		AlloDerm vs. FlexHD<br>Surgical site infection 8.5% vs. 14.4%, p=0.15; multivariate	Include in 5B as used multivariate analysis but only enough pts or events for

Citation	Study name and location	Years of study*	Design	Type of ADM or mesh	Plane of Implant	Number of patients	Population	Surgical details	Outcomes	Other
	Single institution		regression models		coverage of lower pole of the breast<br>No ADM: either partial submuscular with part of lower pole bare or coverage using serratus anterior or rectus abdominis fascia		34.7% of breasts prophylactic; 30% contralateral mastectomy<br>65% of mastectomies for oncologic reasons		OR=0.44, 95% CI=0.19-1.01, p=0.053<br>Delayed healing 21.2% vs. 18.6%, p=0.64; multivariate OR=1.12, 95% CI=0.58-2.19, p=0.74	infection and delayed healing
Ranganathan, 2015 [296]	University of Michigan Health System, Ann Arbor, Mich.<br>Reconstruction by 7 surgeons	1998-2013	Retrospective<br>Multivariate analysis: multinomial logistic model to investigate association of infection with pt characteristic<br>Mean follow-up 20.0 months (2.0 years AlloDerm, 1.4 years FlexHD)	AlloDerm vs. FlexHD	Not reported	309 pts (521 breasts)<br>AlloDerm: 123 pts (206 breasts)<br>FlexHD: 186 pts (315 breasts)	Implant-based reconstruction<br>93.2% immediate, 4.2% delayed, 2.6% both<br>Oncologic indication (by breast): 50.5% vs. 46.7%<br>ADM choice based on surgeon preference<br>41% contralateral mastectomy	Not reported	Results per pt basis<br>•Seroma 6.5% vs. 3.8%, p=0.27; OR=1.24 (95% CI=0.28-5.48), p=0.77<br>•Hematoma 6.5% vs. 5.4%, p=0.68<br>•Major infection (requiring IV antibiotics) 8.1% vs. 17.7%, p=0.039; OR=0.50 (95% CI=0.16-1.00), p=0.049<br>•Delayed wound healing 3.3% vs. 5.4%, p=0.38; OR=0.53 (95% CI=0.13-2.17), p=0.38	Results per breast basis<br>•Seroma 4.4% vs. 2.9%, p=0.36; OR=0.63 (95% CI=0.20-2.02), p=0.44<br>•Hematoma 4.4% vs. 3.2%, p=0.48<br>•Major infection (requiring IV antibiotics) 5.3% vs. 12.7%, p=0.011; OR=0.35 (95% CI=0.17-0.71), p=0.004<br>•Delayed wound healing 2.4% vs. 4.1%, p=0.30; OR=0.51 (95%

Citation	Study name and location	Years of study*	Design	Type of ADM or mesh	Plane of Implant	Number of patients	Population	Surgical details	Outcomes	Other
									•Return to operating room 20.3% vs. 20.4%, p=0.98<br>•Implant exposure 2.4% vs. 2.2%, p=1.0<br>Infection lower with AlloDerm	CI=0.17-1.52), p=0.23<br>•Return to operating room 13.6% vs. 14.3%, p=0.82<br>•Implant exposure 1.5% vs. 1.3%, p=0.86
Sobti, 2016 [297]	A tertiary academic medical centre, Boston, Mass.<br>Single plastic surgeon	2009-2015	Retrospective<br>Unadjusted logistic regression by pt and breast<br>Binomial regression to investigate association between covariates	AlloDerm (RTU or freeze-dried) vs. FlexHD (primarily Pliable Perforated)	Not reported	132 pts (224 breasts) with AlloDerm, 101 pts (170 breasts) FlexHD	ADM-based reconstruction; choice of ADM at surgeon's preference<br>AlloDerm when used: 68.9% RTU, 31.1% freeze-dried<br>FlexHD when used: 80.2% Pliable/Perforated, 18.8% Pliable, 0.9% Structural<br>90.2% vs. 98.0 direct to implant<br>41% contralateral reconstruction	Not reported	Infection (by pt) 4.6% AlloDerm vs. 5.0% FlexHD, univariate p=0.89, binomial regression OR=0.69, 95% CI=0.17-2.56, p=0.56; by breast OR=0.78, 95% CI=0.22-2.72, p=0.69<br>No differences in seroma, hematoma, explantation, delayed would healing, explantation	
Broyles, 2021 [298]	Reconstruction Outcomes in Immediate Post-mastectomy Breast Reconstruction with ADM<br>NCT03145337<br>Multicentre, United States (7 sites)	2016-2018	RCT<br>1:1 blocked randomizatio n per site<br>Designed as noninferiority trial<br>Multivariate models<br>Mean follow-up 10.7 months after completing	AlloDerm RTU vs. FlexHD Pliable; all matrixes were perforated	Prepectoral or partial submuscular	230 pts (384 breasts) were randomized<br>117 pts (197 breasts) AlloDerm RTU; 113 pts (187 breasts) FlexHD Pliable	Immediate implant-based reconstruction<br>Decisions of expander or direct-to-implant (25.6% vs. 21.2%) and of prepectoral (20.8% vs. 20.3%) or partial submuscular plane were at discretion of surgeon<br>SSM (53.0% vs. 60.2%) or NSM<br>40% contralateral reconstruction<br>Study did not measure PROs, aesthetics, long-term	Prepectoral: ADM placed to cover anterior surface of the tissue expander and sutured to the chest wall<br>Partial submuscular: inferior origin of pectoralis major muscle detached from chest wall and ADM inset between inferior	Overall complications 7.1% vs. 4.3%, p=0.233<br>Seroma 4.6% vs. 3.7%, p=0.801<br>Infection 2.5% vs. 1.1%, p=0.450<br>Explantation due to seroma or infection 2.0% vs. 1.6%, p=1.000<br>Only 22 events overall, so multivariate results not extracted	Sample size of 106 pts/group calculated assuming complication rate and noninferiority limit of 8%; final sample size had 80% power to detect effect size odds ratio of 1.83<br>Study underpowered due to lower than

Citation	Study name and location	Years of study*	Design	Type of ADM or mesh	Plane of Implant	Number of patients	Population	Surgical details	Outcomes	Other
			reconstructio n				outcomes such as capsular contraction	border of muscle and IMF		anticipated complication rates
Keifer, 2016 [299]	Emory University (2 reconstructive surgeons) and a private practice (1 reconstructive surgeon), Atlanta, Ga	2010-2015	Retrospective<br>At least 60 days follow-up<br>Binary logistic regression using GEE approach; data reported are uncorrected	AlloDerm vs. Cortiva	Not reported	166 pts, 298 breasts: 174 AlloDerm, 124 Cortiva	Tissue expander (198 breasts) or direct-to-implant (100 breasts)<br>51% vs. 39% of mastectomies due to cancer diagnosis<br>NSM 13.2% vs. 26.6%<br>44% contralateral reconstruction	Not reported	Overall complications 9.2% vs. 14.5%, p=0.195; logistic regression OR=0.55 (95% CI=0.24-1.27), p=0.160<br>Seroma/hematoma 5.2% vs. 5.6%, p=1.000<br>Infection 3.4% vs. 4.0%, p=1.000<br>Mastectomy flap necrosis 0.6% vs. 4.8%, p=0.022; logistic regression OR=0.089 (95% CI=0.007-1.092), p=0.059 [based on only 8 events]	Authors concluded that Cortiva has equivalent complication frequency to AlloDerm
Hadad, 2015 [300]	Brigham and Women's Hospital, Boston, Mass.<br>Single surgeon	2006-2011	Retrospective<br>At least 3-month follow-up	AlloDerm (aseptic), more vs. less	Traditional is dual-plane; minimal use ADM is closer to submuscular	265 pts, 380 breasts<br>108 breasts traditional ADM<br>225 breasts minimal-use ADM<br>35 breasts no ADM in high-risk reconstructions<br>12 breasts did not meet criteria	Prosthesis-based reconstructions (1 or 2 stage); did not include outcomes after exchange of expander for permanent implant<br>Traditional ADM sling with large piece of ADM (167.9 cm^2) along entire released inferior and lateral borders of pectoralis major and chest wall; mostly before 2006-2008; 28 breasts after 2008 because of unusually high pectoralis muscle with respect to IMF or tissue at IMF had excess skeletonization	No ADM: mostly for morbidly obese, used serratus and pectoralis minor muscle/fascia to form later border of breast pocket<br>With ADM: assess viability of skin, muscle integrity especially pectoralis major at inferior and medial origin; create wide subpectoral plane at lateral border of pectoralis, aggressive medial	Seroma: 3% traditional vs. 0% minimal ADM, p=0.01<br>Major skin necrosis 12.0% vs. 12.4%<br>Infection and reconstruction loss 9% traditional vs. 1% minimal ADM, p<0.05<br>Comparison for obese pts (BMI >30 kg/m^2) before and after 2009 when new pathway for ADM use started: 13 pts vs. 53 pts; ADM 207 cm^2 vs. 49 cm^2; major necrosis 23% vs.	Note that it is not only a decrease in ADM, but also alterations in surgery to allow this

Citation	Study name and location	Years of study*	Design	Type of ADM or mesh	Plane of Implant	Number of patients	Population	Surgical details	Outcomes	Other
							Minimal-use ADM (76 cm^2) from 2009-2011 wherever feasible: patching of lateral area of reconstruction; pectoralis is more widely undermined but not released<br><br>No ADM: high-risk reconstructions, mostly morbidly obese with excessive lateral chest wall tissue redundancy or comorbidities that would impair wound healing or ADM incorporation	elevation of the muscle; inferiorly elevate the pectoralis to just below IMF; overall attachment of pectoralis remains intact; lateral opening under pectoralis starting at IMF curves towards axilla; one 8x16 cm thick ADM often sufficient for bilateral reconstruction	17%; seroma 15% vs. 6%, infection and loss of reconstruction 38% vs. 9%	
Lin, 2024 [191]<br><br>Liston, 2024 [192]<br><br>See also Shanno, 2024 [193]	Massachusetts General Hospital, Harvard Medical School, Boston, MA, USA	2007-2019	Single institution, retrospective<br><br>At least 2 years follow-up post-operatively<br><br>NSM complications<br><br>Expander vs. direct-to-implant<br><br>Plane of reconstruction (Q4)<br><br>Use of ADM (Q5)	FlexHD, AlloDerm, Vicryl, Vicryl/ADM hybrid, Surgimend	Not reported	1705 pts and 3035 breasts: number with ADM not reported	NSM and implant-based reconstruction<br><br>Excluded delayed or autologous reconstruction<br><br>Most operations by 3 breast surgeons and plastic surgeons<br><br>Surgeons chose plane of reconstruction and type of ADM/mesh based on experience or preference, pt characteristics, and treatment plan		ADM or mesh vs. muscle only complications:<br>• Overall 9.06% vs. 10.33%; multivariate OR=0.749 (95% CI=0.404-1.391, p=0.361)<br>• Nipple necrosis 1.07% vs. 2.89%, p<0.05; multivariate OR=1.087 (95% CI=0.346-3.415, p=0.886)<br>• Skin flap necrosis 3.44% vs. 5.37%<br>• Infection 3.26% vs. 3.72%<br>• Explantation 4.15% vs. 3.31%<br>• Hematoma 1.32% vs. 2.89%, p<0.05	Complications, AlloDerm vs. Flex HD vs. Vicryl (or Vicryl hybrid) [estimated from graphs]<br><br>Overall: 8.3% vs. 10.2% vs. 11.8%<br><br>Skin flap necrosis; 2.5% vs. 5.8% vs. 5.8%<br><br>Infection 3.1% vs. 2.4% vs. 4.5%<br><br>Explantation 3.4% vs. 6.3% vs. 6.3%<br><br>Rates of nipple necrosis, hematoma, seroma, ruptured

Citation	Study name and location	Years of study*	Design	Type of ADM or mesh	Plane of Implant	Number of patients	Population	Surgical details	Outcomes	Other
									• Seroma 1.00% vs. 1.65%<br>• Ruptured prosthesis 0.61% vs. 0.41%<br>• Reconstruction failure 2.86% vs. 3.31%	prosthesis similar between groups

*Year of diagnosis or initial surgery

Abbreviations:
ADM, acellular dermal matrix; ALND, axillary lymph node dissection; BREASTrial, Breast Reconstruction Evaluation Using Acellular Dermal Matrix as a Sling Trial; BRCA, breast cancer gene; BMI, body mass index; CI, confidence interval; CPT, Current Procedural Terminology codes (USA); GEE, general estimating equation approach; IMF, inframammary fold; IV, intravenous; NSM, nipple-sparing mastectomy; OR, odds ratio; PMRT, postmastectomy radiotherapy; PRO, patient reported outcome; PROM, patient reported outcome measure; pts, patients; REaCT, Rethinking Clinical Trials program; RCT, randomized control trial; RTU, ready to use; SSM, skin-sparing mastectomy

Back to Start of Tables

Table S14. Question 5: Acellular Dermal Matrix. C. ADM Treatments

Citation	Study name and location	Years of study*	Design	Type of ADM or mesh	Plane of Implant, mesh location	Number of patients	Population	Surgical details	Outcomes	
Frey, 2015 [301]<br>Overlaps with Weichman, 2013 [283]	NYU Langone Medical Center, New York, NY<br>Multiple surgeons	2010-2014	Retrospective<br>Univariate analysis for risk factors for infection	Total submuscular coverage (no ADM)<br>AlloDerm: aseptic vs. sterile RTU vs. contoured & fenestrated	Submuscular without ADM<br>Dual-plane with ADM as an inferolateral sling	620 pts (1019 breasts)<br>Total submuscular 645 breasts, aseptic AlloDerm 91 breasts, AlloDerm RTU 164 breasts, AlloDerm contour fenestrated 119 breasts	Immediate tissue expander (86.5%) or permanent implant (13.4%)<br>Total submuscular position with elevation of pectoralis and serratus anterior muscles when possible<br>ADM use if congenital insufficiency or iatrogenic injury making total submuscular placement not possible; decision at surgeon discretion<br>Trends in type of surgery (more NSM) and direct to implant reconstruction and type of ADM over time (switch from aseptic to sterile to sterile contoured) may impact complication rates<br>39% of mastectomies were contralateral		Infection (oral antibiotics) 2.5% vs. 7.7% vs. 3.0% vs. 0<br>Major infection (iv antibiotics) 1.2% vs. 11.0% vs. 4.3% vs. 1.7%<br>Explantation 1.2% vs. 7.7% vs. 3.0% vs. 5.0%<br>Seroma 1.1% vs. 4.4% vs. 1.2% vs. 2.5%<br>Hematoma 1.2% vs. 1.1% vs. 0 vs. 0<br>Minor flap necrosis 2.9% vs. 2.2% vs. 4.9% vs. 10.9%<br>Major flap necrosis 2.9% vs. 11.0% vs. 3.7% vs. 6.7%	Groups were not equivalent:<br>Age 51.0 vs. 49.1 vs. 49.4 vs. 46.4 years old<br>Previous RT 6.7% vs. 3.3% vs. 7.3% vs. 0<br>PMRT 5.9% vs. 14.3% vs. 10.4% vs. 0<br>NSM 30.7% vs. 27.5% vs. 51.8% vs. 68.0%<br>Expander 96.6% vs. 83.5% vs. 77.4% vs. 46.2%<br>Direct implant 3.3% vs. 16.5% vs. 22.6% vs. 53.8%<br>Aseptic had more infections and flap necrosis than sterile RTU (see also Weichman, 2013 [283]) or submuscular<br>Contour had more flap necrosis than sterile RTU, possibly due to more direct implants
Weichman, 2013 [283]	New York University	2010-2012	Prospective cohort study	AlloDerm (aseptic) vs.	Dual-plane with ADM	90 aseptic AlloDerm,	All pts undergoing immediate implant-based	ADM: release of pectoralis major	AlloDerm aseptic vs. RTU	

Citation	Study name and location	Years of study*	Design	Type of ADM or mesh	Plane of Implant, mesh location	Number of patients	Population	Surgical details	Outcomes	
Overlaps with Frey, 2015 [301]	Langone Medical Center<br>5 reconstructive surgeons	Aseptic AlloDerm Nov 2010-Oct 2011<br>AlloDerm RTU Nov 2011-Oct 2012	Univariate logistic analysis	AlloDerm RTU<br>[see other table for AlloDerm RTU vs. none]		105 AlloDerm RTU	reconstruction, either with expander or direct implant<br>Indications for ADM use included lack of muscular coverage, cancer invasion to pectoralis major muscle, immediate implant reconstruction; relative indications included NSM, prior submuscular augmentation<br><u>AlloDerm aseptic vs. RTU</u><br>ADM groups similar in age, 1 or 2-stage implant, SLNB and ALND, indication for surgery, chemotherapy, expander size, fill amount and size, comorbidities, smoking<br>Specimen weight 697 g vs. 587 g, p=0.0485<br>BMI 26.6 vs. 24.92 kg/m^2, p=0.0376<br>NSM 27.5% vs. 49.2%, p=0.0021<br>Prophylactic 38.8% vs. 32.3%	from IMF, addition of ADM as a sling	Flap necrosis 13.3% vs. 10.4% p=0.6571<br>Major flap necrosis 11.1% vs. 5.7%, p=0.1977<br>Infection 20.0% vs. 8.5%, p=0.0088<br>Cellulitis (deep infection) requiring IV antibiotics 12.2% vs. 4.7%, p=0.069	
Yuen, 2014 [302]	University of Arkansas for Medical Sciences<br>Mastectomy by 3 surgeons; single plastic surgeon	Feb-Aug 2012<br>Control June 2011-Jan 2012	Retrospective<br>Multivariable generalized linear mixed model for cellulitis	AlloDerm: aseptic/ freeze-dried vs. sterile/RTU	Dual-plane: internal hammock over lower one-third to one-half of the expander or implant	103 pts: 51 pts (96 breasts) AlloDerm (freeze-dried), 52 pts (100 breasts) AlloDerm RTU	Consecutive pts with immediate singe or 2-stage implant reconstruction with ADM: AlloDerm RTU compared with historical controls in the previous 7 months with AlloDerm<br>Mean BMI ≥30 kg/m^2: 53% vs. 37%<br>Hypertension 39% vs. 23%	2 pieces 8×12 cm AlloDerm in medium-sized breasts or 10×20 cm pieces in larger breasts	Complication per breast, freeze-dried vs. RTU:<br>Seroma 18.8% vs. 22.0%, p=0.599;<br>Cellulitis 12.5% vs. 21.0%, p=0.129; multivariate analysis	Too few patients (27 and 19 per group with BMI ≥30 kg/m^2) for subgroup analysis by BMI<br>Results opposite of those found in other studies, but small number of pts and groups not equivalent

Citation	Study name and location	Years of study*	Design	Type of ADM or mesh	Plane of Implant, mesh location	Number of patients	Population	Surgical details	Outcomes	
							Bilateral reconstruction 90% vs. 92%<br>Mostly NSM (TSSM) 80% vs. 90%<br>47% of mastectomies were contralateral		aOR=0.269, p=0.011<br>Explantation (all due to infection) 7.3% vs. 6.0%, p=0.780	
Parikh, 2018 [246]	Siteman Cancer Center in Saint Louis<br>6 breast surgeons, 3 plastic surgeons	2005-2015	Retrospective<br>Multivariate regression for primary outcomes<br>≥2 years follow-yup	AlloDerm: aseptic/ freeze-dried (2005-2010) vs. sterile/RTU (2010-2015)	Dual-plane (partial submuscular)	1,285 pts (2,039 breasts)<br>612 pts (910 breasts) AlloDerm aseptic; 673 pts (1,129 breasts) AlloDerm RTU	Consecutive pts with immediate implant-based with expander or direct-to-implant reconstruction and AlloDerm ADM<br>Excluded prepectoral<br>Prophylactic mastectomy 0 vs. 7.3%<br>NSM 1.0% vs. 21.4%<br>Bilateral procedure 48.7% vs. 67.8%<br>Direct to implant 0 vs. 9.4%	Elevation of pectoralis major muscle, ADM used for coverage of lower pole as inferolateral sling<br>Non-perforated 8×16 cm sheet for expander or 10×20 cm sheet for direct to implant	Any complication, multivariate analysis OR=1.149 (95% CI=0.843-1.568), p=0.3794<br>Explantation 18.0% vs. 12.0%, p=0.0036; multivariate OR=1.570 (95% CI=1.087-2.267), p=0.0161<br>Infections requiring iv antibiotics 11.3% vs. 10.4%, p=0.6075; multivariate OR=1.064 (95% CI=0.7320-1.546), p=0.7455<br>Wound dehiscence 2.5% vs. 0.9%, p=0.3243<br>Flap necrosis 2.5% vs. 0.7%, p=0.1908<br>Seroma 5.2% vs. 6.0%, p=0.5735<br>Hematoma 1.1% vs. 1.9%, p=0.2531	Smoking, PMRT, BMI were independent predictors of any complication

Citation	Study name and location	Years of study*	Design	Type of ADM or mesh	Plane of Implant, mesh location	Number of patients	Population	Surgical details	Outcomes	
									Implant exposure, malposition, deflation, or rupture 2.5% vs. 0.7%, p=0.1908	
Widmyer, 2019 [303]	Summa Health System, Akron, Ohio<br>Single surgeon	2009-2016	Retrospective<br>Mean follow-up longer in freeze-dried group	AlloDerm: aseptic/ freeze-dried vs. sterile/ RTU	Subpectoral in aseptic group; 92% subpectoral and 8% prepectoral in sterile/RTU group	236 pts (378 breasts); 151 AlloDerm aseptic and 227 AlloDerm sterile RTU	Consecutive implant-based reconstructions, first pts (until 2011) received AlloDerm aseptic and later pts (starting in 2011) received AlloDerm sterile RTU<br>The last 123 breasts using AlloDerm RTU used perforated contoured form<br>Used broader definition of infection than most publications<br>94 unilateral, 142 bilateral<br>Cancer in 116 breasts (49% of pts, 31% of breasts), prophylaxis in 262 breasts (69%)<br>AlloDerm aseptic group younger and lower BMI<br>Expanders 87% vs. 36%<br>38% of mastectomies were contralateral	Not reported	Infection 17% vs. 7.9%, p=0.0083<br>Seroma 9.3% vs. 8.4%, p=0.85<br>Hematoma 2.0% vs. 1.8%, p=1.00<br>Skin necrosis 6.6% vs. 9.3%, p=0.45<br>Dehiscence 5.3% vs. 3.1%, p=0.29<br>Implant loss 9.3% vs. 3.5%, p=0.02<br>Unplanned reoperation 22.5% vs. 9.7%, p=0.001	Infection, explantation, reoperation lower in sterile AlloDerm RTU group, but no multivariate analysis<br>Results for perforated contoured ADM not reported separately
Hanson, 2018 [304]	The University of Texas M. D. Anderson Cancer Center	2006-2016	Retrospective<br>Propensity-score matching, stratified on unilateral and bilateral pts	AlloDerm: aseptic/dried vs. sterile/ RTU	Not reported (but excluded submuscular)	988 breasts; 53.8% freeze-dried, 46.2% RTU<br>384 propensity-score-	Immediate expander/implant (2-stage) reconstruction<br>Excluded total muscle coverage<br>Occurrence at surgical site included seroma, dehiscence, surgical-site		After matching:<br>Early complication 37.5% vs. 28.9%, p=0.011<br>Surgical site occurrence (first	

Citation	Study name and location	Years of study*	Design	Type of ADM or mesh	Plane of Implant, mesh location	Number of patients	Population	Surgical details	Outcomes	
			≥30 days postoperative follow-up (not part of matching) median 50 vs. 24 months			matched pairs	infection, or reconstructive failure<br>≈1/3 cases included prophylactic mastectomies<br>11% NSM<br>Well matched except in ADM area (125.3 vs. 166.7 cm^2) and initial expansion (68.6 vs. 51.4 mL); final volume was similar (530.9 vs. 513.0 mL)		stage) 21.4% vs. 16.7%, p=0.103<br>Infection 9.6% vs. 7.8%, p=0.354<br>Failure 7.8% vs. 4.4%, p=0.050<br>Flap necrosis 24% vs. 18.2%, p=0.054<br>2nd stage complication 0.3% vs. 3.9%, p<0.001	
Han, 2023a [305]	Yonsei University College of Medicine; & Asan Medical Center, University of Ulsan College of Medicine, Seoul, Korea	2018-2020	Retrospective<br>Multivariate analysis was not used<br>Single plastic surgeon	DermACELL or MegaDerm (both gamma-irradiated)<br>Fenestrated during operation to enlarge size of ADM	Prepectoral; wrap-around or anterior coverage with ADM	159 pts: 87 wrap-around, 72 anterior coverage	Immediate prepectoral direct to implant reconstruction<br>Presurgical ptosis evaluated using Regnault classification to determine placement: ptosis grade II or III had wrap-around placement; rest had anterior placement<br>Preoperative characteristics similar between groups	Skin flap evaluated by indocyanine green laser fluorescence; used direct-to-implant if skin flap viability was acceptable<br>Pocket, including IMF defined with sutures<br>For anterior coverage, ADM vs. sutured to anterior surface of pectoralis major, along superomedial part and IMF to create pocket<br>In wrap-around, meshed ADM completed wrapped around implant on a sterile table then inserted over pectoralis major	Overall complications in 13 pts (14.9%) vs. 9 pts (12.5) within 6 months, p=0.82<br>Infection 5.75% vs. 1.39%, p=0.38 early; 1.15% vs. 2.78%, p=0.59 late<br>Seroma 6.90% vs. 5.56%, p=1.0 early; 1.15% vs. 1.39%, p=1.0 late<br>Mastectomy flap necrosis 3.45% vs. 8.33%, p=0.38<br>Total drainage amount 762.1 mL vs. 805.9 mL, p=0.45<br>Capsular contracture 4.6% vs. 1.39%, p=0.38	Complication rates similar; wrap-around can make breast more ptotic in shape than anterior coverage

Citation	Study name and location	Years of study*	Design	Type of ADM or mesh	Plane of Implant, mesh location	Number of patients	Population	Surgical details	Outcomes	
								muscle with ≈ 4 fixation points to upper pole of pectoralis muscle or fascia	Sternal notch-to-nipple distance change 4.44% vs. 2.08%, p=0.03<br>Midclavicle-to-nipple distance change 4.94% vs. 2.64%, p=0.04	
Han 2023b [306]	Yonsei University College of Medicine; Asan Medical Center, University of Ulsan College of Medicine, Seoul, Korea	2019-2020	Retrospective<br>Multivariate analysis not used<br>2 surgeons	ADM, radiation sterilized or not<br>Irradiated: DermACELL, MegaDerm<br>Non-irradiated: CGCryoderm<br>Fenestrated during operation to enlarge size of ADM	Prepectoral<br>ADM product randomly chosen depending on availability	357 pts: 175 sterilized, 182 not sterilized ADM	Immediate prepectoral direct-to-implant breast reconstruction<br>Comorbidities, sterilized vs. non-sterilized: hypertension 4.0% vs. 13.3%; diabetes 2.9% vs. 3.3%, dyslipidemia 1.1% vs. 4.9%, current smoker 2.3% vs. 3.3%<br>BMI and age similar	Skin flap evaluated by indocyanine green laser fluorescence; used direct-to-implant if skin flap viability was acceptable<br>Pocket, including IMF defined with sutures<br>ADM wrapped and sutured around the implant; implant inserted above pectoralis major muscle and incision site closed with sutures	Surgical complications<br>Seroma 9.7% vs. 6.6%, p=0.281<br>Infection 5.7% vs. 4.4%, p=0.634<br>Mastectomy flap necrosis 8.6% vs. 7.7%, p=0.761<br>Capsular contracture 8.0% vs. 7.1%, p=0.759<br>Implant failure 3.4% vs. 0, p=0.013	Similar rates of infection but sterilized group had more return to operating room (6 vs. 1 pt), explantation (4 vs. 0 pts)

*Year of diagnosis or initial surgery

Abbreviations:
ADM, acellular dermal matrix; ALND, axillary lymph node dissection; aOR, adjusted odds ratio; BMI, body mass index; CI, confidence interval; IMF, inframammary fold; IV, intravenous; pts, patients; RTU, ready to use; NSM, nipple-sparing mastectomy; OR, odds ratio; PMRT, postmastectomy radiotherapy; pts, patients; RT, radiotherapy; SLNB, sentinel lymph node biopsy; TSSM, total skin-sparing mastectomy

Back to Start of Tables

Table S15. Question 5: Acellular Dermal Matrix. D. Comparison of synthetic mesh

Citation	Study name and location	Years of study*	Design	Type of ADM or mesh	Plane of Implant, mesh location	Number of patients	Population	Surgical details	Outcomes	Other
Sigalove, 2022 [307]	Washington State University, Vancouver, Wash.<br><br>3 breast oncologic surgeons, 1 reconstructive surgeon	2015-2020	Retrospective<br><br>Chi-square test or one-way analysis of variance<br><br>Follow-up average 41.9 vs. 15.0 months	AlloDerm (early period) vs. GalaFLEX + AlloDerm (later pts)	GalaFLEX-AlloDERM: lower third of expander covered by AlloDerm and rest by GalaFLEX	AlloDerm alone 128 pts (249 breasts); AlloDerm-GalaFLEX 135 pts (250 breasts)	NSM, SSM, or skin-reducing mastectomy<br><br>GalaFLEX investigated as a partial replacement of AlloDerm to reduce cost<br><br>Consecutive pts with immediate expander-implant (2-stage) prepectoral reconstruction<br><br>Both groups well-matched except that BMI and obesity, preoperative chemotherapy, skin-reducing mastectomy, and bilateral reconstruction higher in AlloDerm alone group; NSM lower in AlloDerm alone group (33.7% vs. 54.0%)	Reconstruction when flap perfusion deemed adequate, as assessed clinically (early part of study) or a perfusion assessment device<br><br>Prepectoral space collapsed and adjusted to accommodate expander; expander wrapped with matrix (AlloDerm or AlloDerm on lower anterior portion and inferior gutter and GalaFLEX covering remainder of expander both anteriorly and posteriorly); matrix secured to pectoralis major muscle and subcutaneous tissue<br><br>2 drains with AlloDerm, or one drain with AlloDerm-GalaFLEX	Any complication 7.6% vs. 6.4%, p=0.590; all were in first year<br><br>Infection 1.6% vs. 2.0%, p=0.741<br><br>Major skin necrosis 2.8% vs. 0.8%, p=0.091<br><br>Any skin necrosis 5.2% vs. 1.2%, p=0.011<br><br>Seroma 2.8% vs. 3.2%, p=0.799<br><br>Capsular contracture 0.8% in each, p=0.997<br><br>Prosthesis exposure/ extrusion 1.6% vs. 3.2%, p=0.245<br><br>Prosthesis loss	Skin necrosis lower in AlloDerm-GalaFLEX group, but could be due to differences in pt/disease characteristics and operating factors (expertise development and transition in methods to evaluate perfusion)<br><br>Authors indicate GalaFLEX is stiffer and therefore gives a more stable pocket but is not preferred for lower pole to allow for expansion

Citation	Study name and location	Years of study*	Design	Type of ADM or mesh	Plane of Implant, mesh location	Number of patients	Population	Surgical details	Outcomes	Other
Levy, 2020 [308]	Weill Cornell Medicine, New York, NY<br>Protocol 1604017199 R001	2011-2016	Retrospective<br>3 breast surgeons, 1 plastic surgeon<br>Follow-up 26 vs. 15 months<br>Multivariate analysis not used	AlloMax vs. Phasic™ (P4HB)<br>ADM was fenestrated with No. 15 scalpel blade<br>ADM or P4HB soaked in antibiotic saline for 60 seconds prior to use	Subpectoral (dual-plane)<br>AlloMax for first 112 pts, P4HB for next 62 pts	107 pts, 192 reconstructions with ADM vs. 62 pts (112 cases) with P4HB mesh	INSM or SSM, immediate expander-based reconstruction for breast cancer or prophylaxis<br>Baseline characteristics similar<br>2 different time periods: 2011-Oct 2014 for AlloMax; Oct 2014-2016 for P4HB	Pectoralis major released from inferior and inferomedial attachments to create submuscular pocket; ADM or mesh fixed to IMF and lateral chest wall; expander placed in pocket and cephalad portion of ADM or mesh trimmed and secured in place; expander filled with minimal amount of saline to unfold (no pressure on overlying skin)	Infection: 17.71% vs. 11.21%, p=0.18; OR=0.59, p=0.15<br>Time to drain removal 18 vs. 15 days, p=0.008<br>Major complications 24.11% vs. 14.52%, p=0.17, OR=0.55, p=0.14<br>Minor complications 9.82% vs. 11.29%, p=0.80; OR=1.11, p=0.83<br>Seroma 3.13% vs. 0.93%, p=0.43, OR=0.17, p=0.11<br>Necrosis 8.85% vs. 9.35%, p=1.00; OR=0.77 p=0.54<br>Need for reoperation 16.15% vs. 13.08%, p=0.51, OR=0.78, p=0.48<br>Explant 10.00% vs. 11.21%, p=0.70	Shorter follow-up in P4HB group<br>No significant differences in univariate analysis (ORs)<br>In pts with chemotherapy there were more reoperations in the ADM group, 14.94% vs. 0, p=0.0021
Chen, 2023b [244]	Weill Cornell Medicine, New York, NY	2012-2021	Retrospective, univariate; Cox proportional-hazards model	Prepectoral (no ADM) vs. dual-plane with ADM vs.	Prepectoral (no ADM) vs. dual-plane with ADM vs. dual-plane with P4HB	220 pts, 393 samples: 161 prepectoral (no mesh), 122 dual-plane with ADM, 96 dual-	2-stage reconstruction<br>No significant baseline differences between groups	Prepectoral: anterior to pectoralis muscle and directly under the soft tissue	Mean time to full expansion 53.4 vs. 104 vs. 68.8 days<br>Necrosis 11.8% vs. 13.1% vs. 4.2%	

Citation	Study name and location	Years of study*	Design	Type of ADM or mesh	Plane of Implant, mesh location	Number of patients	Population	Surgical details	Outcomes	Other
				dual-plane with P4HB		plane with P4HB [and 14 with total submuscular, less than current review threshold]		Dual-plane: partially subpectoral with support material	Infection 13.0% vs. 13.1% vs. 7.3%<br>Revision surgery 46.6% vs. 46.7% vs. 41.7%<br>Capsular contraction 30.4% vs. 34.4% vs. 47.9%;<br>Capsular contraction, univariate: OR=1.20 (0.73-1.98, p=0.47) compared with dual + ADM; OR=2.10 (95% CI=1.25-3.56, p=0.005) compared with dual +P4HB<br>Capsular Contracture, multivariate: dual + ADM vs. prepectoral HR=1.00 (95% CI=0.66-1.52, p=0.99); dual + P4HB vs. prepectoral HR=1.58 (95% CI=1.05-2.36, p=0.03)	

Citation	Study name and location	Years of study*	Design	Type of ADM or mesh	Plane of Implant, mesh location	Number of patients	Population	Surgical details	Outcomes	Other
Houvenaeghel, 2024 [232]<br>See also Houvenaeghel, 2022 [245]	Marseille, France<br>M-IBR-PPRP-IPC 2022-014	2019-2023	Retrospective<br>Univariate and multivariate (binary logistic regression) for complications<br>Prepectoral vs. subpectoral complications and satisfaction<br>11 surgeons, surgeons were significantly different between groups	Resorbable synthetic TIGR Matrix	176 (54.3%) prepectoral with TIGR Matrix, 9 (1.7%) subpectoral with TIGR Matrix<br>Prepectoral use varied by year: 20%, 14,%, 92%, 85%, 6.7% for years 2019 to 2023; dramatic decrease in 2023 due to reports of negative impact on complication	324 prepectoral (increased from 3.1% in 2019 to 61.7% in 2023)<br>529 subpectoral	Immediate implant-based reconstruction (100% vs. 94.7% direct-to-implant)<br>66.4% vs. 44.6% NSM; 33.0% vs. 54.8% SSM<br>108 bilateral prophylactic and 61 bilateral primary breast cancer, 1 bilateral for LR<br>Implant position and mesh use was at surgeon discretion		In regression analysis:<br>Smoking, larger breasts (cup size >C), higher ASA status, mesh use, incision (areolar, inverted T) had higher complications<br>Smoking, mesh use, mastectomy weight >300 g, diabetes increased grade II-III complications	

*Year of diagnosis or initial surgery

Abbreviations:
ADM, acellular dermal matrix; ASA status, American Society of Anesthesiologists physical status classification system; BMI, body mass index; CI, confidence interval; GalaFLEX is a synthetic mesh that retains strength for at least 12 months and degrades to water and carbon dioxide in 18-24 months; HR, hazard ratio; IMF, inframammary fold; LR, local recurrence; NSM, nipple-sparing mastectomy; OR, odds ratio; P4HB, poly-4-hydroxybutyrate; pts, patients; SSM, skin-sparing mastectomy

Back to Start of Tables

Table S16. Question 6: Autologous Fat Grafting

Citation	Study name and location	Years of study*	Topic or comparison	Design	Number of patients	Patient characteristics	Surgery/technique	Results	Other
Schop, 2021 [57] (protocol)<br>See Piatkowski, 2023 [55]	BREAST Trial<br>NCT02339779<br>7 hospitals in the Netherlands	2015-2019; final completion 2026 for secondary oncologic outcomes (estimated)	Fat grafting only<br>Pre-expansion + autologous fat transfer for full breast reconstruction vs. expander-implant	RCT, multicentre<br>Primary outcome QoL at 12 months after final surgery	196 planned: 98 autologous fat transfer, 98 expander-implant	History of or candidate for mastectomy; scheduled mastectomy (cancer or prophylactic) or previous mastectomy and desire breast reconstruction<br>Exclude active smoking, uncontrolled diabetes, BMI >30 kg/m^2, breast size more than C cup unless contralateral reduction desired, substance abuse, chemotherapy within 4 weeks prior, history of or planned RT	Fat harvested with 2-3 mm incisions, infiltration with solution of saline/ lidocaine/ epinephrine, fat harvested with 10 mL syringe and blunt cannula, fat processed in Puregraft system to wash out contaminants (blood, oil from ruptured adipocytes), fat injected in microdroplets and aliquots fanning in different planes (prepectoral, interpectoral, retropectoral) and into deep and superficial dermis; shaping by layering the fat into different levels until desired contour achieved<br>Timing of BRAVA use is ambiguous:<br>NCT registry indicates the first fat transfer to deep tissue planes during primary mastectomy surgery<br>Schop, 2021 [57] indicates pre-expansion with BRAVA (external suction, 10+ hours/day for 4 weeks); figure suggests this is only prior	Outcomes:<br>Primary outcome is QoL by BREAST-Q questionnaire, using Emotional, Sexual and Physical Well-Being subscales<br>Other outcomes are quality of reconstruction by volume and shape (3D photos or MRI), patient satisfaction by BREAST-Q, aesthetic judgement by panel<br>Complications<br>Oncological outcomes up to 5 years	Power calculation assumes clinically relevant change in QoL is half a standard deviation, 90% power, dropout 15%

Citation	Study name and location	Years of study*	Topic or comparison	Design	Number of patients	Patient characteristics	Surgery/technique	Results	Other
							to 2nd and 3rd fat injections<br>BRAVA worn for 2 weeks after initial grafting and pressure compression garment for 2-4 weeks<br>Additional sessions of fat grafting (up to 5) as needed; on average 2 or more<br>Control: tissue expander under major pectoralis muscle and 10 mL saline inserted; edges of major pectoralis and serratus anterior muscle sutured, drain inserted, skin closed; expansion starting after 14 days with 50-100 mL saline per visit (at least 3 weeks apart); after expansion the mastectomy scar, expander, and capsule removed, and implant inserted in muscular pocket		
Piatkowski, 2023 [55]	BREAST Trial	2015-2019, with 12-month follow-up to 2021	See above	RCT<br>12 months follow-up	193 randomized; this publication 91 autologous fat transfer, 80 implants (18/98 refused implants); 64 and 68	See Schop, 2021 [57]<br>Type of surgery (NSM, SSM, other) not reported<br>Pathological staging missing for 26.4% vs. 37.5% of patients and clinical staging not reported	After mastectomy, fat grafting in subpectoral and intra-pectoral areas<br>Pre-expansion for 4 weeks and post-expansion for 2 weeks using EveBra Nonsurgical Natural Breast Enlargement [additional publication [54] states BRAVA device was used]	Breast-Q scores (scores 0-100 with higher scores being better satisfaction or QoL); result at 12 months; difference of ≥4 was clinically relevant (≥3 for Physical Well-Being)<br>Baseline scores were heterogeneous as some patients had already had	Additional publications with <50 pts/group explored donor site satisfaction [309] and breast sensibility [54]<br>Authors graded this as Level I evidence

Citation	Study name and location	Years of study*	Topic or comparison	Design	Number of patients	Patient characteristics	Surgery/technique	Results	Other
					completed follow-up	Staging not conducted in 17.6% vs. 16.3% denoted as preventive<br><br>Mean BMI 23.8 vs. 23.2 kg/m^2	Harvested fat as above, purified using PureGraft 250 for unilateral cases or PureGraft 850 for bilateral cases	mastectomy and some also had an implant<br><br>Satisfaction with Breasts 70.3 vs. 60.4, p=0.002<br><br>Psychosocial Well-Being 69.6 vs. 68.3, p=0.66<br><br>Physical Well-Being: Chest 79.9 vs. 72.3, p=0.007<br><br>Sexual Well-Being 61.5 vs. 58.6, p=0.37<br><br>Satisfaction with Outcome 73.9 vs. 66.3, p=0.04<br><br>QoL change over time favoured fat transfer<br><br>Mean breast volume 300.3 ±111.4 mL vs. 384.1 ± 86.6 mL<br><br>Mean treatment duration 13.4 vs. 5.1 months<br><br>Oncologic serious adverse events, 4 vs. 5, not significant<br><br>Non-oncologic serious adverse events 4 vs. 13<br><br>Adverse events 43 vs. 25; included 10 vs. 1 seroma, 7 vs. 2 hematoma, 2 vs. 6 infection, 7 vs. n/a abscess or fat necrosis, 7 vs. n/a blisters due to external expander (BRAVA), 4 vs. n/a irritation due to external	

Citation	Study name and location	Years of study*	Topic or comparison	Design	Number of patients	Patient characteristics	Surgery/technique	Results	Other
								expander, 0 vs. 4 skin necrosis, n/a vs. 7 rupture or migration or capsular contraction (expander or implant)	
Gentilucci, 2020 [53]	Italy	2016-2017	Prophylactic lipofilling after RT vs. no lipofilling in expander-implant reconstruction	RCT<br>Follow-up of 1 year after expander-implant exchange	60 pts: 30 pts with fat injections and 30 without	Excluded if medical history of connective, metabolic, or skin diseases; metastatic breast cancer; family history of breast cancer or genetic background; intraepithelial malignancies<br>Chemotherapy used if positive nodes; hormone therapy if ER+ and/or PR+<br>All pts had immediate expander-implant reconstruction and external RT to whole breast and tumour bed boost	Fat injections using Coleman's technique after RT and before expander removal<br>Fat purified and injected multi-directionally from superficial to deep layers throughout irradiated area; 3 fat injections were used with the first session 2-3 months after end of RT and thereafter every 3 months; expander removed 3 months after last fat injection and replaced with implant<br>In group without lipofilling, expander replacement was 3-6 months after end of RT<br>Skin biopsies at 5 cm from medial edge of mastectomy scar at time of each fat injection and expander removal; in second group at time of expander removal; samples measured for thickness and histological examination	Liponecrosis in 3.3% of lipofilling cases and was treated conservatively<br>Delayed wound healing 3.3% vs. 13.2%; hematoma 3.3% vs. 0; seroma 10% vs. 16.5%; implant extrusion 0 vs. 6.6% (treated with latissimus dorsi flap reconstruction)<br>Group without lipofilling had more complications, p=0.07<br>Soft tissue thickness similar at end of RT, but increased in lipofilling group (51.8%, 109.6%, and 178.3% after 1st, 2nd, and 3rd fat injections)<br>Dermal fibrosis similar after RT, but disappeared in lipofilling group<br>Capsular contraction Grade II 12 cases vs. 10 cases; Grade III 18 cases vs. 12 cases; Grade IV 0 cases vs. 8 cases; p<0.01<br>LENT-SOMA scale to measure degree of disability: there was significant reduction in	

Citation	Study name and location	Years of study*	Topic or comparison	Design	Number of patients	Patient characteristics	Surgery/technique	Results	Other
								scores after lipofilling but not in group without lipofilling<br>Aesthetic evaluation by patient and independent plastic surgeon using visual analogue scale of 1 to 10: better in lipofilling group but data not reported	
Bennett, 2017 [58]	The Mastectomy Reconstruction Outcomes Consortium (MROC) Study, USA (10 institutions) and Canada (1 institution);<br>NCT01723423	2012-2016	PROs comparing pts with fat grafting between years 1 and 2 vs. those without fat grafting between years 1 and 2; pts could have had fat grafting prior to end of 1st year after start of reconstruction<br>Implant or autologous	Prospective, multicentre cohort design; followed STROBE guidelines for cohort studies<br>Multivariable analysis (mixed-effects regression)	4436 pts in full study; 2048 in this publication<br>165 pts with fat grafting between years 1 and 2	First-time reconstruction after mastectomy and ≥2 years follow-up; breast mound reconstruction completed within 1 year of starting reconstruction<br>Breast cancer (90.9% vs. 89.3%) or prophylaxis; primary reconstruction; implant (52.7% vs. 60.5%) or autologous<br>Fat grafting used for contour irregularities or volume deficits<br>Primary outcome: change from baseline PROs using BREAST-Q; 0 to 100 point scales with higher number indicating better HRQoL	Not reported	Patients who later had fat grafting had lower QoL before grafting compared with pts without, but similar QoL after fat grafting<br>Results at 1 year after start of reconstruction; pts with subsequent fat grafting vs. pts without<br>• Breast Satisfaction 60.1 vs. 66.1, adjusted p=0.008<br>• Psychosocial Well-Being 67.2 vs. 73.5, adjusted p=0.03<br>• Physical Well-Being 72.5 vs. 76.2, adjusted p=0.33<br>• Sexual Well-Being 48.0 vs. 54.7, adjusted p=0.008<br>Results at 2 years after start of reconstruction; pts with fat grafting during 2nd year vs. pts without	Suggests that for pts with defects amenable to fat grafting, fat grafting improves QoL to level similar to those who did not require fat grafting

Citation	Study name and location	Years of study*	Topic or comparison	Design	Number of patients	Patient characteristics	Surgery/technique	Results	Other
								• Breast Satisfaction 65.6 vs. 66.0, adjusted p=0.72<br>• Psychosocial Well-Being 73.2 vs. 75.3, adjusted p=0.73<br>• Physical Well-Being 74.8 vs. 76.8, adjusted p=0.73<br>• Sexual Well-Being 52.8 vs. 55.4, adjusted p=0.15	
Petit, 2012 [310]	European Institute of Oncology (IEO) Breast Cancer Database, Milan, Italy	1997-2008	Lipofilling vs. none	Retrospective, matched 1 lipofilling to 2 without<br>Median follow-up 56 months from primary surgery and 26 months from lipofilling	321 lipofilling, 642 matched pts without lipofilling<br>Invasive cancer: 284 pts vs. 568 pts<br>In situ cancer 37 pts vs. 74 pts	Primary breast cancer<br>Excluded distant metastasis at diagnosis, recurrence prior to lipofilling, bilateral tumour, previous cancer, NACT<br>Controls matched for age, year of surgery, type of surgery (quadrantectomy (38.9%) or mastectomy 61.1%), invasive (88.5%) or ductal intraepithelial neoplasia (10.9%) or lobular intraepithelial neoplasia (0.6%)), tumour size, ER status<br>Controls were disease free at least until time of fat grafting of matched case<br>Cases but not controls had complete clinical examination at time of lipofilling and declared	Method as published by Coleman: fat removed by liposuction from subcutaneous tissue, soft centrifugation to remove blood cell contaminants, and injected in area where needed<br>Recently new techniques to increase percentage of preadipocytes and better graft take, but publication does not indicate whether these were used	Local event (local or locoregional recurrence) HR=1.11, 95% CI=0.47-2.64, p=0.792; mastectomy subgroup HR=1.92, 95% CI=0.68-5.43, p=0.211	

Citation	Study name and location	Years of study*	Topic or comparison	Design	Number of patients	Patient characteristics	Surgery/technique	Results	Other
						disease free, which may create a bias			
Gale, 2015 [311]<br>Authors aimed to replicate study of Petit, 2012 [310]	Nottingham Breast Institute, Nottingham, UK	Initial treatment 1977-2013<br>Fat grafting 2007-2013	Fat grafting vs. none	Retrospective, matched 1:2 for date of operation, age, type of surgery, tumour histology, ER status, disease-free status at time equivalent to that of fat grafting<br>Multivariate Cox proportion hazard regression for recurrence<br>Mean follow-up 88 months after primary surgery and 32 months after fat grafting	211 pts fat grafting, 422 controls	Pts with previously treated malignant breast disease; 83.4% vs. 84.8% had mastectomy<br>Fat grafting indications: breast asymmetry, contour deformity, correction of RT-induced fibrosis, volume enhancement<br>Excluded if recurrence before fat grafting; controls had disease-free period at least as long as time from oncologic surgery and fat grafting of corresponding study pt	Fat grafting by Coleman technique without stem cell enhancement: injected tumescence solution of 150 mg of levobupivacaine in 1 liter of 0.9% normal saline using a blunt cannula<br>1:1,000,000 adrenaline	LR 0.95% vs. 1.90%, p=0.327<br>Regional nodal recurrence 0.95% vs. 0%, p=0.164<br>LRR 1.9% vs. 1.9%<br>DM 3.32% vs. 2.61%, p=0.691<br>New contralateral cancer 1.90% vs. 0.24%, p=0.224<br>Breast cancer-related death 1.90% vs. 3.32%, p=0.297<br>Any oncologic event: 7.1% vs. 4.7%, p=0.654	
Seth, 2012 [312]	Northwestern Memorial Hospital, Northwestern University, Chicago, Illinois<br>15 mastectomy surgeons, 6	1998-2008	Long-term outcomes with vs. without fat grafting	Retrospective<br>Mean follow-up 43.6 and 42.1 months (24.8 months after first fat-grafting)<br>Multiple linear regression for association of	886 pts (1202 breasts): 69 pts (90 breasts) fat-grafting and 817 pts (1112 breasts) non-fat grafting	Mastectomy with immediate tissue expander reconstruction, RT if needed, 2nd-stage expander-implant exchange<br>Independent variables in multiple linear regression included age, BMI, smoking status, RT before or after	Fat harvesting and grafting using techniques of Coleman: harvest by syringe, fat separated and concentrated using gravity and manual separation of fluid from fat on a Telfa dressing without use of centrifugation; 20-200 cm^3/breast injected	Local recurrence 0 vs. 17 breasts (0% vs. 1.5%), p=0.63<br>Survival 100% vs. 95.5% of pts, p=0.10<br>Pts were more likely to undergo subsequent fat grafting if they had a first-stage complication following tissue expander	

Citation	Study name and location	Years of study*	Topic or comparison	Design	Number of patients	Patient characteristics	Surgery/technique	Results	Other
	reconstructive surgeons			first stage complications and subsequent fat grafting<br>Mean follow-up 43.6 and 42.1 months		mastectomy and reconstruction, ADM use, the individual mastectomy and reconstructive surgeon, and each complication subtype	(majority received 20-50 cm^3), injected primarily in superior portion of breast but generally in areas of visual depression; injections subcutaneously and intramuscularly	placement (p<0.0001), particularly an operative complication (p<0.0001)	
Kim, 2014 [313]	Yonsei University College of Medicine, Seoul, Korea	2005-2013	Long-term efficacy and safety of fat graft vs. none	Retrospective<br>28.7 months average follow-up	102 pts with fat grafting; 449 controls without fat graft	Breast cancer; fat grafting for secondary revision<br>Autologous (56 pts) and implant (46 pts) with fat grafting; controls were from same time period but without fat grafting	Donor sites were abdomen (91 pts) or thigh (11 pts); fat harvested by Coleman's technique: a tumescent solution of 1,000 mL normal saline, 20 mL 2% lidocaine, and 1 mL 1:100,000 epinephrine was injected into the fat harvest area, waited 20 minutes, fat harvested with two-hole 3 mm Coleman cannula on 50 mL Luer Lock syringe, centrifuged at 3000 rpm for 3 minutes, supernatant oil wicked off and discarded, fluid decanted, fat layer placed into syringes for transfer through Coleman cannulas into the soft-tissue deformities suing small aliquots and multiple passes and tissue planes<br>Average 49.3 mL fat injected per breast	During follow-up of average 28.7 months, 17.6% had minor complications of fat necrosis (10 pts) or cyst formation 8 pts) and all were conservatively managed<br>Complications increased with graft volume: mean volume 45.2 mL without complications and 67.5 mL with complications<br>Fat resorption 32.9%<br>No infections or implant rupture<br>LRR 0.9% (1 pt) vs. 2% (9 pts)	
Masia, 2015 [314]	2 institutions in Barcelona, Hospital de	1989-2011	Fat grafting vs. none	Retrospective<br>Median follow-up 60 months	207 pts: 100 pts (107 breasts) fat	Consecutive pts with mastectomy (for invasive or in situ carcinomas)	Fat harvested without anesthetic infiltration from fat deposits using 2-	LR in 3 pts before and 3 pts after fat grafting; LR in 6 pts in control group:	

Citation	Study name and location	Years of study*	Topic or comparison	Design	Number of patients	Patient characteristics	Surgery/technique	Results	Other
	Santa Creu i Sant Pau and Clinica Planas			from surgery to baseline and 29 months from baseline to most recent follow-up<br><br>Control group follow-up 120 months<br><br>Median follow-up for lipofilling group 60 months from surgery to baseline and 29 months baseline to most recent follow-up	grafting vs. 107 controls	and reconstruction using free flaps<br><br>Exclude distant metastasis at diagnosis, recurrent tumours, BCS, <12 months follow-up after fat grafting<br><br>Overweight (BMI 25-30 kg/m^2) 14% vs. 32.7%; alcohol use 10.2% vs. 3.7%, tobacco 31.8% vs. 40.2%, other characteristics similar<br><br>Fat transfer group had more involved nodes (N2 13.6% vs. 4.9% and N3 4.8% vs. 2%) and more stage 3 cancers, more ER+ (69.2% vs. 50.9%)	3 mm diameter 1-2 blunt opening cannulas on 10 cc Luer-Lok syringes; centrifuged in Coleman machine for 1 min at 2000 rpm, fat layer transferrin in 3 cc syringes for injection<br><br>Average 102.8 cc in 1st session and 96.2 cc in 2nd session	2.8% vs. 5.6%, HR=0.66, 95% CI=0.16-2.66<br><br>Metastasis 1 vs. 4 pts<br><br>DFS HR=0.66, 95% CI=0.16-2.66, p=0.555	
Fertsch, 2017 [315]	Department of Plastic and Reconstructive Surgery at the SANA Klinik, Düsseldorf, Germany	2009-2013	Lipofilling vs. none	Retrospective<br><br>Matched controls<br><br>Median follow-up 72.5 vs. 76.5 months from mastectomy; 32 vs. 31 months from startpoint to end of follow-up	100 pts lipofilling, 100 pts matched control without lipofilling	Pts with breast cancer, total mastectomy, and delayed DIEP flap reconstruction<br><br>Exclude bilateral cancer, prophylactic mastectomy, recurrence prior to DIEP-flap or lipofilling<br><br>Controls were recurrence-free up until start of study follow-up<br><br>Matching by age (within 5 years), year of surgery, year of DIEP-flap, histopathology, HR and HER2 status, stage,	Fat harvesting technique of Coleman	Recurrence 7 vs. 11 pts, HR=0.57, 95% CI=0.22-1.47, p=0.24	

Citation	Study name and location	Years of study*	Topic or comparison	Design	Number of patients	Patient characteristics	Surgery/technique	Results	Other
						grade, recurrence risk factor group<br><br>29% vs. 52% overweight pts (BMI ≥25 kg/m^2)			
Myckatyn, 2017 [316]	4 centres (Memorial Sloan Kettering, M. D. Anderson Cancer Center, Alvin J. Siteman Cancer Center, and the University of Chicago)	2006-2011 study period (including recurrence monitoring)	Association between fat transfer and time to recurrence	Retrospective case cohort study; powered to detect risk ratio of ≥2 for recurrence<br><br>Cox proportional hazards regression for association between fat transfer and recurrence in bivariate and multivariate models	225 recurrences (cases), 972 without recurrence (controls)<br><br>Fat transfer in 64 pts	Mastectomy and immediate reconstruction; invasive ductal carcinoma (stage I-III)<br><br>Cases were all recurrences; controls were 30% random sample of pts without recurrence during study period<br><br>Covariates: fat transfer, age, stage, smoking status, BMI, ER/PR/HER2 status, chemotherapy, RT, endocrine therapy	Fat transfer recorded as yes or no, no details	Recurrence, fat transfer vs. none: unadjusted HR=0.99 (95% CI=0.56-1.7), p=0.99; adjusted HR=0.97, (95% CI=0.54-1.8), p=0.93<br><br>Fat transfer not associated with higher risk of recurrence	
Silva-Vergara, 2017 [317]	Universidad Autónoma de Barcelona; and Hospital Clinic of Barcelona, Barcelona, Spain	2007-2015	Effect of lipofilling on recurrence	Retrospective<br><br>Matched 1:2 (fat-grafting and controls)<br><br>Multivariate Cox proportional hazards regression model for LRR<br><br>Follow-up 88.7 vs. 86.8 months	205 fat grafting and 410 matched controls	Pts with history of cancer and 2-stage breast reconstruction<br><br>Mastectomy 71.7% vs. 69.8%; BCS 28.3% vs. 30.2%<br><br>Excluded prophylactic mastectomy, recurrence before fat grafting<br><br>Controls matched for date of primary cancer operation (within 3 years), age (within 5 years), type of cancer surgery, histopathology,	Fat grafting with few variations from Coleman technique and without stem cell enhancement; cites previous publication Silva-Vergara 2016 [318]<br><br>Tumescence included 1 L of 0.9% normal saline with adrenaline (1:1,000,000); lipoaspiration with 3 mm cannulas and vacuum pump at 40 kPa and intermediary 400 mL drainage bottle for fat storage; fat washed with saline solution,	Recurrence 14 vs. 32 pts, 6.8% vs. 7.8%, p=0.526<br><br>LR 2.4% vs. 3.2%, p=0.485<br><br>LRR 3.4% vs. 3.9%, p=0.525<br><br>DM 3.4% vs. 3.9%, p=0.590<br><br>Locoregional PFS HR=0.749, 95% CI=0.31-1.83, p=0.525<br><br>Mortality 2.9% vs. 3.4%, p=0.400<br><br>Subgroups with mastectomy:	

Citation	Study name and location	Years of study*	Topic or comparison	Design	Number of patients	Patient characteristics	Surgery/technique	Results	Other
						lymphatic involvement, and ER status<br>Control had disease-free period at least as long as time between oncologic surgery and fat grafting for corresponding fat grafting pt<br>Fat grafting mean 167 mL per session and mean of 1.5 sessions (mean 257 mL total); in pts with mastectomy mean volume 277.9 mL vs. 202.7 mL in BCS	centrifuged at 2000 rpm (400 g) for 2 minutes, cellular fraction transferred to 10 mL syringes and injected with 1.9 mm blunt cannulas through several punctures	LR 2.7% vs. 3.5%, p=0.567<br>DM 4.1% vs. 4.9%, p=0.587<br>Mortality 2.0% vs. 2.8%, p=0.323	
Calabrese, 2018 [319]	Italy<br>1 different reconstructive surgeon for each fat grafting group	2007-2011	SVF-enriched fat transfer vs. fat transfer vs. none	Prospective study<br>Logistic regression for factors associated with recurrence<br>≥5 years follow-up from 2nd stage of reconstruction<br>Median follow-up 84, 75, 72 months from expander-implant exchange	41 pts SVF + fat transfer, 64 pts fat transfer, 64 pts control	Pts undergoing NSM and 2-stage reconstruction; age 18-75 years, histologically proven Tis-T2N0-N2M0 breast adenocarcinoma; no documented recurrence or systemic disease at enrollment<br>Exclude other cancers except cervical/vulvar intraepithelial neoplasia, previous breast cancer, severe comorbidities<br>Controls were pts who did not require fat grafting upon enrollment<br>Fat grafting was at time of expander-implant exchange (median 10, 9, 12 months from first stage)	Coleman's technique for standard fat transfer using centrifugation of the lipoaspirate<br>SVF-enrichment conducted as in RESTORE-2 trial [320] with adipose tissue divided into two parts: portion to enrich added to Celution system, Celase proteolytic enzyme added, then residual enzyme removed and cells (adipose-derived regenerative cells, ADRC) concentrated with the automated system; second portion purified by gravity sedimentation/ floatation; ≈5 mL A added to second portion and this enriched fat graft transferred to breast	Any recurrence event: 4 vs. 4 vs. 3 events; 9.8% vs. 6.3% vs. 4.7%<br>• Enriched vs. control adjusted OR=1.92, 95% CI=0.36-10.31, p=0.477<br>• Normal fat transfer vs. control adjusted OR=1.26, 95% CI=0.25-6.42, p=0.778<br>LR 0, 4.7%, 1.6%<br>LRR 2.4% vs. 4.7% vs. 1.6%<br>Systemic (distant) recurrence 7.3% vs. 3.1% vs. 3.1%<br>DFS 19, 22, 25 months from last reconstructive stage; 37, 34, 38 months after NSM	

Citation	Study name and location	Years of study*	Topic or comparison	Design	Number of patients	Patient characteristics	Surgery/technique	Results	Other
						Pts with surgeon CC were in group 1 (SVF-enriched) and pts with surgeon DC were in group 2 (fat-grafting without enrichment)	using 60 mL Toomey syringes<br><br>Tissue expander removed, implant positioned, mastectomy flap dissection using a blunt cannula in a fan-shaped direction to include all over the breast mound represented by the new implant; dissection carried out from the surgical incision, in the subcutaneous space between skin and implant capsula along with pectoralis muscle fibers; fat transfer in this pre-tunneled plane using the Celbrush® for enriched group and standard cannulas for regular fat grafting<br><br>Yearly follow-up including ultrasound; MRI for suspicious cases; continued for 5 years after 2nd stage procedure		
Calabrese, 2020 [321]	Academic Hospital of Udine, University of Udine, Udine, Italy<br><br>1 surgeon<br><br>Single institute	2010-2014	Fat grafting prior to or at time of exchange vs. no fat grafting at time of exchange in 2-stage implants<br><br>Note fat grafting allowed in both groups as a secondary	Retrospective<br><br>Used STROCSS reporting criteria<br><br>Follow-up average 32.91 vs. 36.15 months	84 pts vs. 130 pts	Unilateral mastectomy with implant-expander reconstruction; including 13.10% vs. 10% SSM and 8.33% vs. 6.92% NSM<br><br>1 to 3 deflation-lipofilling sessions; if only 1 session it was at time of expander/implant exchange; 2 lipofilling sessions before	After expander, there were serial deflation-lipofilling (if multiple sessions of lipofilling planned) then positioning of definitive implant during last lipofilling session<br><br>Fat positioned at the level of the mastectomy flap between skin and	Capsular contracture 7.14% vs. 21.53%, p=0.004<br><br>Complications<br><br>• Hematoma 0 vs. 4.62%, p=0.045<br>• Seroma 2.38% vs. 3.85%, p=0.556<br>• Implant infection 1.19% vs. 3.08%, p=0.372	Expander/ implant and lipofilling procedure details not reported; number of pts with deflation-lipofilling (more than 1 session of planned

Citation	Study name and location	Years of study*	Topic or comparison	Design	Number of patients	Patient characteristics	Surgery/technique	Results	Other
			procedure subsequent to final implant insertion, occurred in 21.43% vs. 26.15% of pts, p=0.431			expander-implant exchange for pts with RT or skin flap thickness <0.5 cm (pinch test with calipers and ultrasound)<br>Control without lipofilling<br>Tissue expanders were left in place for 13-19 weeks	periprosthetic capsule; deflation to be 10 cc superior to total amount of fat injected; if skin too tight after fat grafting a greater amount of saline was removed from the expander<br>Silicone gel implants were placed in subpectoral pocket	• Implant exposure 0 vs. 3.08%, p=0.104<br>• Rupture 0 vs. 0.77%, p=0.420<br>• Displacement/ rotation 10.72% vs. 29.24%, p=0.001<br>• Asymmetry 11.09% vs. 16.92%, p=0.314<br>• Pain 3.57% vs. 13.85%, p=0.013<br>Reoperations<br>• Prosthesis replacement 5.95% vs. 12.31%, p=0.127<br>• Prosthesis removal 0 vs. 1.54%, p=0.253<br>• Revision surgery within 3 years 21.43% vs. 34.62%, p=0.038<br>• Lipofilling 21.43% vs. 26.15%, p=0.431<br>Aesthetics and PROs (BREAST-Q): fat grafting group reported significantly better for several scales of satisfaction (softness, natural appearance and feel to touch, natural part of body) and of Physical Well-Being (pain in chest muscles; breast tightness, pulling, nagging feeling, sharp pains, aching feeling, throbbing feeling)	lipofilling) not reported

Citation	Study name and location	Years of study*	Topic or comparison	Design	Number of patients	Patient characteristics	Surgery/technique	Results	Other
								No differences in Psychosocial and Sexual Well-Being questions	
Krastev, 2019 [322]	Tergooi Hospital in Hilversum, the Netherlands<br>One surgeon for fat transfer	2006-2014; analyze 2016-2017	Autologous fat transfer vs. none<br>Mean follow-up 9.3 years (5.0 years after fat transfer) vs. 8.6 years from primary surgery	Retrospective matched cohort<br>Mean follow-up 9.3 years vs. 8.6 years (5.0 vs. 4.4 years after fat-grafting time point)	287 pts (300 breasts) fat grafting, matched 1:1 with 300 controls<br>161 vs. 150 mastectomy	Histologically confirmed breast cancer and who received fat transfer for correction of contour deformities; matching to pts without fat transfer based on age, type of oncologic surgery, tumour invasiveness, stage, same locoregional recurrence-free interval at baseline<br>Excluded prophylactic mastectomy, LRR	Tumescent infiltration, harvest (abdomen or upper legs) using closed low-pressure suction system (0.5 atm) and 3 mm multiple-hole cannula; centrifugation occasionally used to remove excess blood or oil from ruptured adipocytes<br>Purifie fat transferred to 10 mL syringes and injected percutaneously into breast deformity with 2 mm blunt cannula in multiple passes and tissue planes; forked cannula used to perforate scar adhesions and fibrosis whenever necessary	Results in pts with BCS or mastectomy<br>LRR: 8 pts vs. 11 pts, 2.67% vs. 3.67%, rates per breast: HR=0.63 (95% CI=0.25-1.60), p=0.33; rates per pt: HR=0.64; 95%CI=0.25-1.62, p=0.34<br>DM adjusted HR=0.98, 95% CI=0.54-1.79, p=0.95<br>Breast cancer related mortality adjusted HR=0.38, 95% CI=0.15-0.92, p=0.03<br>Overall mortality 2.8% vs. 11%; adjusted HR=0.20, 95% CI=0.09-0.44, p<0.001 [OS 97.2% vs. 89.0%]<br>In pts with mastectomy: LRR unadjusted HR=0.78, 95% CI=0.23-2.73, p=0.71	Authors cannot explain the excess mortality in the non-fat graft group
Cason, 2020 [323]	Duke University Medical Center, Durham, NC	2010-2018	Influence of fat grafting on incidence of imaging and biopsies vs. no fat grafting	Retrospective<br>Propensity-matched 1:1<br>≥6 months follow-up since fat grafting	93 matched pairs (93 fat grafted, 93 without)	Autologous or implant reconstruction<br>Excluded metastatic, bilateral prophylactic mastectomy, BCS<br>Propensity score using laterality, BMI, reconstruction timing	98.9% of liposuction was suction assisted<br>Fat processing: 12.5% centrifugation, 53.4% revolve system, 19.3% gravity, 14.98% other	Palpable masses 38.0% vs. 18.3%, p=0.003<br>Post-reconstruction imaging 47.3% vs. 29.0%, p=0.01<br>Biopsies performed 11.8% vs. 7.5%, p=0.32<br>Imaging predominately interpreted as normal	Higher detection of palpable masses but not biopsies as fat-related changes are identifiable on imaging

Citation	Study name and location	Years of study*	Topic or comparison	Design	Number of patients	Patient characteristics	Surgery/technique	Results	Other
				Mean follow-up 4.4 vs. 4.1 years		Multivariate model to predict receipt of fat grafting		(27.9% vs. 33.3% BI-RADS 1) or benign (48.8% vs. 50.0% BI-RADS 2); 7.0% vs. 0% BI-RADS 3, 16.3% vs. 16.7% BI-RADS 4<br>Fat necrosis most frequent radiologic interpretation, 45.5% vs. 14.8%<br>5-y OS: 94.6% vs. 95.0%, p=0.49<br>5-y locoregional RFS 95.1% vs. 96.2%, p=0.888	
Vyas, 2020 [324]	University of Kentucky Markey Cancer Center, Lexington, KY<br>5 surgeons for lipofilling	2000-2017	Fat grafting vs. none	Retrospective, matched case control study<br>Follow-up ≥12 months<br>Cox proportional hazard model for recurrence using age, stage, fat grafting status<br>Median follow-up 42.5 months	72 pts (116 breasts) fat grafting, 181 pts (312 breasts) without fat grafting<br>Therapeutic mastectomy: fat grafting in 73 breasts vs. 200 breasts without<br>Prophylactic: fat grafting in 43 breasts vs. 112 without	Prophylactic or therapeutic mastectomy and breast reconstruction and at least 12 months oncologic follow-up<br>Matching 1:3 by age (5-year increments), surgical service, ASA class, wound class<br>Mean fat grafting 97.9 mL per pt; 74.1% had one grafting procedure	Fat harvested using 30 mL or 60 mL syringes and suction cannulas; centrifuged and oil and serous components decanted; processed fat transferred to 3 mL syringes and injected using blunt infiltration cannulas	Therapeutic mastectomy: LRR 8.2% vs. 8.5%, p=1.000<br>LRR and/or DM 8.2% vs. 9.0%, p=1.000<br>Median time to recurrence 31.5 vs. 37.0 months, p=0.973<br>Adjusted model for time to recurrence: HR=0.807, 95% CI=0.31-2.1, p=0.66	Suboptimal matching<br>Fat grafting group has less comorbidities that could affect short-term outcomes; higher stage disease, bilateral disease, BRCA1/2 positive<br>High rate of unknown data for pt/disease characteristics
Casarrubios, 2021 [325]	University Hospital Dr Negrín, Las Palmas de Gran Canaria, Spain	2011-2019	Fat grafting vs. none	Retrospective<br>Matched controls<br>Used STROBE reporting checklist	125 fat grafting, 125 matched controls<br>Of these, 80 vs. 82 pts with	History of breast cancer and reconstructed with fat grafting (alone in BCS pts; together with flaps or implants in pts with mastectomy) vs. no fat grafting; exclude if LR	Coleman technique with no additional cell enhancement<br>Fat grafting was prior to tissue expander insertion (6-183 months after oncologic surgery) in all	Pts with mastectomy:<br>LRR 1.3% vs. 3.7%, p=0.429<br>DM 1.3% vs. 6.1%, p=0.135	<25 events so multivariate results not extracted<br>Control group had more lymphadenectom

Citation	Study name and location	Years of study*	Topic or comparison	Design	Number of patients	Patient characteristics	Surgery/technique	Results	Other
				Mean follow-up 47.2 months after lipofilling	mastectomy (rest had BCS)	before fat grafting, positive margins, prophylactic mastectomy<br>36.0% vs. 34.4% BCS<br>Fat grafting alone in 45 pts with BCS; in rest of pts was used in combination with flaps or implants to improve shape (56 pts) or to improve skin quality prior to implants in pts with previous RT (24 pts)<br>Matched by date of first oncological surgery, age, type of oncological surgery, histological subtype, HER-2 status, pN, smoking habit, diabetes mellitus<br>Selected control pt had a disease-free period at least as long as the time window between oncologic surgery and the fat grafting procedure of the corresponding study patient	except 1 pt; in 32.5% of mastectomy cases lipofilling was >3 years from cancer surgery	Total recurrences 2.5% vs. 9.8%, p=0.097<br>All pts:<br>Locoregional PFS HR=0.40, 95% CI=0.10-1.61, p=0.183	y and chemotherapy
Klinger, 2022 [326]	17 Senonetwork breast units, Italy	2000-2018	Recurrence and survival with vs. without fat grafting	Retrospective<br>Median 60 months follow-up (63 vs. 58 months)<br>Reporting using STROBE guidelines	After matching: 466 fat graft and 923 no fat graft<br>Mastectomy subgroup: 228 pts fat grafting and	Early breast cancer pts with invasive cancer: BCS (51.5% vs. 53.4%) or mastectomy (48.9% vs. 46.6%) in matched cohort<br>Fat grafting only used for regenerative purpose of addressing painful scars,	Fat grafting using Coleman technique	LRR (includes both mastectomy and BCS) 3.9% vs. 6.1%, p=0.084<br>LRFS calculated from lipofilling to last contact or recurrence for fat grafting; calculated from primary surgery to last contact or recurrence for no fat grafting; matched	5% difference in LRR considered to be equivalent (5% alpha error and 80% power)<br>LRFS had different follow-up starting points so

Citation	Study name and location	Years of study*	Topic or comparison	Design	Number of patients	Patient characteristics	Surgery/technique	Results	Other
					430 non-fat grafting	post-actinic dystrophic tissue, local deformities		patients for follow-up time<br>LRFS aHR=0.73, 95% CI=0.41-1.30, p=0.291;<br>for mastectomy subgroup: aHR=0.32, 95% CI=0.11-0.92, p=0.034	difficult to interpret
Palve, 2022 [327]	Tampere University Hospital, Tampere, Finland	2008-2019	Techniques in latissimus dorsi reconstruction: addition of implants, lipofilling, or none	Retrospective<br>Univariable and multivariable logistic regression to compare reconstruction techniques<br>Median follow-up 88 months, 67 months, 117 months	291 flaps in 283 pts<br>161 implant enhanced, 73 lipofilled, 57 plain flaps	Latissimus dorsi reconstruction<br>Delayed reconstruction 89% vs. 78% vs. 77%	Not reported	Overall complications 66% vs. 75% vs. 74%, p=0.228<br>Seroma (all fluid collections requiring aspiration) 95% vs. 84% vs. 92%<br>Hematoma 58% vs. 33% vs. 80%<br>Major complications<br>• Partial flap necrosis 19% vs. 50% vs. 20%<br>• Deep infections 23% vs. 17% vs. 0%	Note: breast reconstruction but cancer not mentioned<br>Use of implant decreased over time (39% in 2008 to 11% in 2019) and fat grafting increased (11% in 2008 and 89% in 2019); authors suggest fat grafting can replace implant and allows less aggressive subcutaneous tissue harvesting in latissimus dorsi site than in none group (no implant or fat grafting)
Sorotos, 2022 [328]	Sapienza University of Rome, Rome, Italy<br>Single centre	2005-2017	Impact of fat transfer vs. none on LR	Retrospective<br>Cases matched 1:n with controls for age, histology, year of	902 pts, 1025 breasts: 464 fat transfer and 561 no fat transfer	Breast cancer, mastectomy (NSM, SSM, other), and breast reconstruction (implant or flap)	Fat harvested using dry technique (no tumescence) and processed by centrifugation (Coleman procedure) at 3000 rpm	Primary endpoint was LR<br>LR 3.3% vs. 10.9%, HR=0.337, 95% CI=0.173-0.658, p=0.00007<br>Metastasis 2.5% vs. 15.4%	Large difference in LR before and after multivariate analysis (significant and in favour of fat

Citation	Study name and location	Years of study*	Topic or comparison	Design	Number of patients	Patient characteristics	Surgery/technique	Results	Other
				surgery, type of mastectomy<br>Average follow-up 62.98 months (12-144 months) from primary reconstruction surgery<br>Minimum follow-up of 3 years after first fat transfer	before matching<br>After matching: 425 cases and 494 controls	Excluded distant metastases at diagnosis, recurrence, incomplete data of primary tumour, prophylactic mastectomy, BCS<br>Fat transfer for total volume restoration (only in NSM) or aesthetic refinements after reconstruction<br>Median time from primary surgery to first fat transfer was 11 months<br>Control pts had disease-free period at least as long as interval between oncologic surgery and fat transfer of corresponding case<br>Arms unbalanced for LN status (LN+ 34.0% vs. 47.3%), grade (grade 3 38.4% vs. 44.0%), HR+ (67.5% vs. 79.8%), HER2+ (21.5% vs. 31.3%), RT (31.6% vs. 46.7%), chemotherapy (46.9% vs. 72.7%)	for 3 minutes with no stem cell enhancement<br>Fat injected into breasts in thin continuous strips with crisscrossed strokes at multiplanar levels using a 3-way stopcock connecting 10 mL syringes to 1 mL syringes for more precise delivery	Multivariate analysis LR HR=1.29, 95% CI=0.59-2.79	grafting to non-significant in favour of no fat grafting); maybe due to baseline imbalance of non-matched characteristics
Lee, 2023 [329]	Sungkyunkwan University of Medicine, Seoul, South Korea<br>Single institution	2011-2016	Association of fat grafting during expander-implant exchange or not on cancer prognosis	Retrospective cohort<br>Univariable and multivariable Cox regression analyses	Second stage (exchange) at ≤12 months: 112 fat grafting, 91 no fat grafting	Unilateral invasive cancer and immediate 2-stage implant after SSM or NSM mastectomy; contralateral prophylactic mastectomy allowed	Tissue expanders in subpectoral space with lateral surface covered with ADM or serratus anterior fascia; inflation within 1 month if there was complete wound healing; second stage	*Expander-implant exchange within 1 year*<br>Recurrence 8.9% vs. 3.3%<br>LR 4.5% vs. 2.2%<br>Regional lymph node recurrence 2.7% vs. 0	Groups unbalanced in baseline characteristics: fat graft group had higher rate of SLNB + axillary dissection (30.4%

Citation	Study name and location	Years of study*	Topic or comparison	Design	Number of patients	Patient characteristics	Surgery/technique	Results	Other
			Stratified by timing of exchange (≤12 months, >12 months)	Median follow-up 65.0 months from mastectomy and 53.0 months from 2nd stage operation	Only 64 cases with exchange at >12 months (29 fat graft and 35 without) so data not extracted	Excluded in situ cancer, bilateral cancer, distant metastasis at time of operation, neoadjuvant chemotherapy, prophylactic mastectomy without therapeutic mastectomy, recurrence after BCS; recurrence prior to insertion of implant<br>Decision of fat grafting based on breast shape, expected aesthetic outcomes, and cost; was independent of oncologic status<br>Most implants were textured, so only reported results for textured implants	operation ≥3 months after completion of expansion<br>Tissue expander removed and implant inserted in same space<br>Fat graft harvested using Coleman technique: infiltrating tumescent solution injected, adipose tissue harvested using closed manual pressure suction and 3 mm multiple-hole cannula; aspirate centrifuged to remove excel blood or oil from ruptured adipocytes; purified fat transferred to 10 mL syringe and injected to breast percutaneously into subcutaneous and prepectoral planes with 2 mm blunt cannula with maximum scatter of fat droplets	DM 3.6% vs. 1.1%<br>Breast cancer-related death 2.7% vs. 0<br>5-y LRRFS 95.0% vs. 100%, p=0.026; univariable HR=6.325 (95% CI=1.114-35.905, p=0.037); multivariable HR=33.127 (95% CI=1.332-823.935, p=0.033)<br>5-y DFS 93.4% vs. 98.7%, p=0.037; univariable HR=4.238 (95% CI=1.011-17.759, p=0.048); multivariable HR=5.701 (95% CI=1.164-27.927, p=0.032)<br>Very high survival and therefore too few events for reliable multivariable analysis<br>_Subset with expander-implant exchange at >1 yr_<br>Any recurrence 6.9% vs. 14.3%; LR 0% vs. 5.7%; regional recurrence 3.4% vs. 2.9%; DM 3.4% vs. 5.7%; 5-y LRRFS 95.2% vs. 89.0%; 5-y DFS 95.2% vs. 82.1%<br>_Both subgroups combined_<br>Any recurrence 8.5% vs. 6.3%; LR 3.5% vs. 3.2%; regional recurrence 2.8% vs. 0.8%; DM 3.5% vs. 2.4%; 5-y LRRFS 95.0% vs.	vs. 23.1%), node positive cancer (21.4% vs. 14.3%), higher stage (stage II 40.2% vs. 35.2%), LVI (31.3% vs. 24.2%), chemotherapy (49.1% vs. 41.8%)<br>Suggests fat transfer group had higher baseline risk of recurrence<br>In multivariate analysis, stage, LVI, were risk factors<br>Using HRs for survival endpoints is problematic to interpret, as survival is a positive outcome, and HRs are normally used for events (not absence of events). It is unclear whether HR=5.701 means better survival or worse survival for the fat grafting arm after

Citation	Study name and location	Years of study*	Topic or comparison	Design	Number of patients	Patient characteristics	Surgery/technique	Results	Other
								96.8%; 5-y DFS 94.3% vs. 94.4%	multivariate analysis
Strong, 2024 [330]	Data from Optum Clinformatics Data Mart<br><br>University of Michigan; and dVA Center for Clinical Management Research, VA Ann Arbor Healthcare, System, Ann Arbor, MI	2001-2018	Fat grafting vs. none	Retrospective from claims data for privately insured pts<br><br>Bivariate analysis on entire patient sample<br><br>Propensity score-matching estimated using multivariable logistic regression based on 60 baseline characteristics<br><br>At least 5 years enrollment time for assessment of metastasis and mortality	4709 pts:<br><br>361 fat grafting, matched to 361 controls	Patients with breast reconstruction after lumpectomy or mastectomy for breast cancer (including carcinoma in situ) according to ICD-9 or ICD-10<br><br>Fat grafting identified by CPT code and within 2 years of reconstruction<br><br>Excluded lymph node metastasis or distant metastasis at the time of initial breast cancer diagnosis<br><br>97.5% in each group had mastectomy; >99% had invasive carcinoma, 82.5% vs. 84.2% implant-based	Not reported	Matched groups:<br><br>Metastasis: 57/361 (15.8%) vs. 65/361 (18.0%)18.0%, p=0.427<br><br>Lymph node metastasis 35/361 (9.7%) vs. 41/361 (11.4%), p=0.467; HR=0.831 (95% CI=0.529-1.305, p=0.421)<br><br>Distant metastasis 33/361 (9.1%) vs. 38/361 (10.5%), p=0.532; HR=0.848 (95% CI=0.532-1.352, p=0.489)<br><br>Mortality 14/361 (3.9%) vs. 24/361 (6.6%), p=0.096	
Escandon, 2024 [331]<br><br>NEW	University of Rochester Medical Center, NY, USA	2011-2021	Latissimus dorsi + immediate fat transfer vs. standard latissimus dorsi reconstruction	Retrospective	130 pts with 195 reconstructions:<br><br>119 with immediate fat transfer; 76 standard	Autologous breast reconstruction after total mastectomy using latissimus dorsi flaps, with or without immediate fat transfer<br><br>94.9% SSM	Volume of fat, plane of injection, and skin paddle de-epithelialization based on surgeon preference<br><br>Fat harvested from flanks/abdomen or thighs, processed using REVOLVE fat system, injected in latticed	Anesthesia time 541 vs. 520 min<br><br>Secondary fat grafting 61.3% vs. 23.7%, p<0.001<br><br>Less wound disruption at donor site 12.6% vs. 23.7%; authors indicated this could be due to more	Higher secondary fat grafting could be due to patient factors regarding those who chose to have initial fat grafting

Citation	Study name and location	Years of study*	Topic or comparison	Design	Number of patients	Patient characteristics	Surgery/technique	Results	Other
			Cox proportional-hazards models<br>Median follow-up 147.9 weeks		latissimus dorsi flap	Mean BMI 30.8 vs. 34.7 kg/m^2<br>Smokers 4.2% vs. 11.8%<br>Similar comorbidities<br>69.7% vs. 71.1% therapeutic	multilayer technique with a 10 mL syringe and 3 mm Coleman cannula; quilting sutures for closure of the donor site<br>Median volume fat harvested was 125 mL and median volume transferred into latissimus dorsi flap was 70 mL; median volume to pectoralis major was 62 mL; 8.4% of mastectomy flaps received fat transfer (median 35 mL)	aggressive flap harvest in the absence of fat grafting and not due to fat grafting itself<br>Breast site complications were similar (no significant differences)	
Navarro, 2024 [332]	Institut Universitaire du Cancer de Toulouse - Oncopole, France	2007-2015	Impact of lipofilling on survival	Retrospective<br>Univariable and multivariable analyses using log-rank test and Cox proportional hazards model<br>Median follow-up 55.2 months	550 breasts: 136 lipofilling, 414 control without lipofilling	Immediate breast reconstruction after mastectomy for breast cancer<br>Exclude prophylactic, sarcoma, delayed reconstruction, reconstructive failure, or local recurrence before fat grafting<br>Excluded pts who had oncologic event or died within 24 months of surgery or without 24 months follow-up<br>SSM 89.0%% vs. 88.2%; NSM 8.8% vs. 10.9%<br>73.5% vs. 88.2% implant<br>25.7% vs. 13.5% autologous with latissimus dorsi flap	Lipofilling using the Coleman technique<br>No pre-expansion<br>Fat harvested from knees, flanks, or thighs with closed suction system or a 10 mL syringe; centrifugation (1000 g for 2 min) sometimes used to remove excess blood or oil; densest fat reinjected with 10 cc syringe percutaneously	Recurrence or survival events in 7.4% vs. 10.4%<br>5-y RFS 90.9% vs. 92.3% p=0.9569; by multivariate analysis, aHR=0.93 (95% CI=0.46-1.88, p=0.833)<br>Recurrence 8/136 (5.9%) vs. 38/414 (9.2%)<br>LRR 6/136 (4.4%) vs. 20/414 (4.8%)<br>DM 5/136 (3.7%) vs. 22/414 (5.3%)<br>Cancer-related deaths 1/136 (0.7%) vs. 11/414 (2.7%)<br>Median time to recurrence after first surgery 52.3 vs. 45.7 months	

Citation	Study name and location	Years of study*	Topic or comparison	Design	Number of patients	Patient characteristics	Surgery/technique	Results	Other
						Multifocal tumours (34.6% vs. 25.2%)			

*Usually year of diagnosis or initial surgery

Note: Coleman's technique, Coleman, 2006 [340]; Coleman, 2007 [341]

Abbreviations:
ADM, acellular dermal matrix; ADRC, adipose-derived regenerative cells; aHR, adjusted hazard ratio; ASA status, American Society of Anesthesiologists physical status classification system; BCS, breast conserving surgery; BI-RADS, Breast Imaging Reporting and Data System; BMI, body mass index; BRAVA, BRA like VAcuum-based external tissue expander; BREAST Trial, Breast Reconstruction With External Preexpansion & Autologous Fat Transfer vs. Standard Therapy Trial; CI, confidence interval; CPT, Current Procedural Terminology codes (USA); DFS, disease-free survival; DIEP, deep inferior epigastric perforators; DM, distant metastasis; ER, estrogen receptor; HER2, human epidermal growth factor receptor 2; HR, hazard ratio; ICD, International Statistical Classification of Diseases and Related Health Problems; LENT-SOMA scale, Late Effects Normal Tissues (LENT)-Subjective, Objective, Management, Analytic (SOMA) scale; LR, local recurrence; LRR, locoregional recurrence; LRRFS, locoregional recurrence-free survival; LVI, lymphovascular invasion; MROC, Mastectomy Reconstruction Outcomes Consortium; MRI, magnetic resonance imaging; NACT, neoadjuvant chemotherapy; n/a, not applicable; NSM, nipple-sparing mastectomy; OR, odds ratio; OS, overall survival; PFS, progression-free survival; PR, progesterone receptor; PRO, patient-reported outcomes; pts; patients; QoL, quality of life; RCT, randomized controlled trial; RFS, recurrence-free survival; RT, radiotherapy; STROBE, strengthening the reporting of observational studies in epidemiology (www.strobe-statement.org); SLNB, sentinel lymph node biopsy; SSM, skin-sparing mastectomy; STROCSS, Strengthening the Reporting of Cohort Studies in Surgery statement [342]; SVF, stromal vascular fraction

References

1. Saiga, M.; Nakagiri, R.; Mukai, Y.; Matsumoto, H.; Kimata, Y. Trends and issues in clinical research on satisfaction and quality of life after mastectomy and breast reconstruction: a 5-year scoping review. *Int. J. Clin. Oncol.* **2023**, *28*, 847-859, https://dx.doi.org/10.1007/s10147-023-02347-5
2. Sousa, H.; Castro, S.; Abreu, J.; Pereira, M. G. A systematic review of factors affecting quality of life after postmastectomy breast reconstruction in women with breast cancer. *Psychooncology*. **2019**, *28*, 2107-2118, https://dx.doi.org/10.1002/pon.5206
3. Winters, Z. E.; Benson, J. R.; Pusic, A. L. A systematic review of the clinical evidence to guide treatment recommendations in breast reconstruction based on patient-reported outcome measures and health-related quality of life. *Ann. Surg.* **2010**, *252*, 929-942, https://dx.doi.org/10.1097/SLA.0b013e3181e623db
4. Pusic, A. L.; Klassen, A. F.; Snell, L.; Cano, S. J.; McCarthy, C.; Scott, A.; Cemal, Y.; Rubin, L. R.; Cordeiro, P. G. Measuring and managing patient expectations for breast reconstruction: Impact on quality of life and patient satisfaction. *Expert Rev. Pharmacoecon. Outcomes Res.* **2012**, *12*, 149-158, https://dx.doi.org/10.1586/erp.11.105
5. Kronowitz, S. J. A systematic review of the clinical evidence to guide treatment recommendations in breast reconstruction based on patient-reported outcome measures and health-related quality of life: Winters ZE, Benson JR, Pusic AL (Univ of Bristol, UK; Univ of Cambridge, UK; Memorial Sloan-Kettering Cancer Ctr, NY) Ann Surg 252:929-942, 2010. *Breast Dis.* **2011**, *22(4)*, 420-422, https://dx.doi.org/10.1016/j.breastdis.2011.10.042
6. Wu, Z. Y.; Kim, H. J.; Lee, J.; Chung, I. Y.; Kim, J.; Lee, S. B.; Son, B. H.; Han, J.; Han, H. H.; Eom, J. S.; et al. Oncologic safety of nipple-sparing mastectomy in patients with breast cancer and tumor-to-nipple distance <= 1 cm: A matched cohort study. *Ann. Surg. Oncol.* **2021c**, *28*, 4284-4291, https://dx.doi.org/10.1245/s10434-020-09427-0
7. Zhu, M.; Mao, J.; Fang, J.; Chen, D. Safety of atypical ductal hyperplasia at the nipple margin in nipple-sparing mastectomy. *J. Breast Cancer.* **2024**, *16*, 16, https://dx.doi.org/10.4048/jbc.2024.0077
8. Parvez, E.; Martel, K.; Morency, D.; Dumitra, S.; Meguerditchian, A. N.; Dionisopoulos, T.; Meterissian, S.; Basik, M.; Boileau, J. F. Surgical and oncologic outcomes of nipple-sparing mastectomy for a cohort of breast cancer patients, including cases with high-risk features. *Clin. Breast Cancer.* **2020**, *20*, 353-358, https://dx.doi.org/10.1016/j.clbc.2020.03.001
9. Hung, Y. C.; McCarthy, J. T.; Park, B. C.; Chaker, S. C.; Saad, M.; Braun, S. A.; Perdikis, G.; Higdon, K. Comparison of complication rates between subpectoral vs prepectoral techniques in prosthetic breast reconstruction. *Aesthet. Surg. J.* **2023**, *43*, 1285-1292, https://dx.doi.org/10.1093/asj/sjad145
10. Woo, K. J.; Park, J. W.; Mun, G. H.; Pyon, J. K.; Jeon, B. J.; Bang, S. I. Does the use of acellular dermal matrix increase postoperative complications of the first-stage reconstruction of immediate expander-implant breast reconstruction: A matched cohort study. *Ann. Plast. Surg.* **2017**, *79*, 341-345, https://dx.doi.org/10.1097/SAP.0000000000001067
11. Wells, M. W.; Chang, I. A.; Gatherwright, J. R.; Festekjian, J. H.; Delong, M. R. Postsurgical outcomes with meshes for two-stage prosthetic breast reconstruction in

20,817 patients. *Plast. Reconstr. Surg. Glob. Open.* **2022**, *10*, e4699, https://dx.doi.org/10.1097/GOX.0000000000004699
12. Wu, Z. Y.; Kim, H. J.; Lee, J.; Chung, I. Y.; Kim, J. S.; Lee, S. B.; Son, B. H.; Eom, J. S.; Kim, S. B.; Gong, G. Y.; et al. Recurrence outcomes after nipple-sparing mastectomy and immediate breast reconstruction in patients with pure ductal carcinoma in situ. *Ann. Surg. Oncol.* **2020**, *27*, 1627-1635, https://dx.doi.org/10.1245/s10434-019-08184-z
13. Seth, A. K.; Hirsch, E. M.; Fine, N. A.; Kim, J. Y. S. Utility of acellular dermis-assisted breast reconstruction in the setting of radiation: A comparative analysis. *Plast. Reconstr. Surg.* **2012**, *130*, 750-758, https://dx.doi.org/10.1097/PRS.0b013e318262f009
14. Agarwal, J. P.; Mendenhall, S. D.; Anderson, L. A.; Ying, J.; Boucher, K. M.; Liu, T.; Neumayer, L. A. The breast reconstruction evaluation of acellular dermal matrix as a sling trial (BREASTrial): Design and methods of a prospective randomized trial. *Plast. Reconstr. Surg.* **2015**, *135*, 20e-28e, https://dx.doi.org/10.1097/PRS.0000000000000809
15. Pu, Y.; Mao, T. C.; Zhang, Y. M.; Wang, S. L.; Fan, D. L. The role of postmastectomy radiation therapy in patients with immediate prosthetic breast reconstruction: A meta-analysis. *Medicine.* **2018**, *97*, e9548, https://dx.doi.org/10.1097/MD.0000000000009548
16. Christopher, A. N.; Morris, M. P.; Broach, R. B.; Serletti, J. M. A comparative analysis of immediate and delayed-immediate breast reconstruction after postmastectomy radiation therapy. *J. Reconstr. Microsurg.* **2022**, *38*, 499-505, https://dx.doi.org/10.1055/s-0041-1740123
17. Prantl, L.; Moellhoff, N.; von Fritschen, U.; Giunta, R. E.; Germann, G.; Kehrer, A.; Lonic, D.; Zeman, F.; Broer, P. N.; Heidekrueger, P. I. Immediate versus secondary DIEP flap breast reconstruction: A multicenter outcome study. *Arch. Gynecol. Obstet.* **2020**, *302*, 1451-1459, https://dx.doi.org/10.1007/s00404-020-05779-w
18. Liew, B.; Southall, C.; Kanapathy, M.; Nikkhah, D. Does post-mastectomy radiation therapy worsen outcomes in immediate autologous breast flap reconstruction? A systematic review and meta-analysis. *J. Plast. Reconstr. Aesthet. Surg.* **2021**, *74*, 3260-3280, https://dx.doi.org/10.1016/j.bjps.2021.08.005
19. Beugels, J.; Bod, L.; van Kuijk, S. M. J.; Qiu, S. S.; Tuinder, S. M. H.; Heuts, E. M.; Piatkowski, A.; van der Hulst, R. Complications following immediate compared to delayed deep inferior epigastric artery perforator flap breast reconstructions. *Breast Cancer Res. Treat.* **2018**, *169*, 349-357, https://dx.doi.org/10.1007/s10549-018-4695-0
20. Zhong, T.; Spithoff, K.; Kellett, S.; Boyd, K.; Brackstone, M.; Hanrahan, R.; Whelan, T.; and the Breast Reconstruction Expert Panel. Breast cancer reconstruction surgery (immediate and delayed) across Ontario: Patient indications and appropriate surgical options. Toronto (ON): Cancer Care Ontario; 2016 Jan 5 [warning on implant-associated anaplastic large cell lymphoma (BIA-ALCL) added 2019 Nov 26 and interim revision 2021 Nov]. Program in Evidence-Based Care Guideline No.: 17-10. Available from: https://www.cancercareontario.ca/en/guidelines-advice/ until Version 2 is posted; thereafter contact the Program in Evidence-Based Care by email at ccopgi@mcmaster.ca.
21. Fletcher, G.; Zhong, T.; Frank, S.; Hanrahan, R.; Vesprini, D.; Stevens, C.; Wright, F.; Stotland, P.; Miragias, V.; Vito, A.; et al. Postmastectomy breast reconstruction in patients with non-metastatic breast cancer. PROSPERO 2023 CRD42023409083. Updated 2025 Feb 18. Available from: https://www.crd.york.ac.uk/prospero/display_record.php?ID=CRD42023409083.

22. Zhong, T.; Fletcher, G. G.; Brackstone, M.; Frank, S.; Hanrahan, R.; Miragias, V.; Stevens, C.; Vesprini, D.; Vito, A.; Wright, F. C.; et al. Postmastectomy breast reconstruction in patients with non-metastatic breast cancer. Toronto (ON): Ontario Health (Cancer Care Ontario); 2025 March 19. Program in Evidence-Based Care Guideline No.: 17-10 v2. Available from: https://www.cancercareontario.ca/en/guidelines-advice/types-of-cancer/31721.
23. Page, M. J.; McKenzie, J. E.; Bossuyt, P. M.; Boutron, I.; Hoffmann, T. C.; Mulrow, C. D.; Shamseer, L.; Tetzlaff, J. M.; Akl, E. A.; Brennan, S. E.; et al. The PRISMA 2020 statement: An updated guideline for reporting systematic reviews. *BMJ*. **2021**, *372*, n71. doi: 10.1136/bmj.n1171. See also: http://www.prisma-statement.org/, 10.1136/bmj.n71
24. Liberati, A.; Altman, D. G.; Tetzlaff, J.; Mulrow, C.; Gøtzsche, P. C.; Ioannidis, J. P. A.; Clarke, M.; Devereaux, P. J.; Kleijnen, J.; Moher, D. The PRISMA statement for reporting systematic reviews and meta-analyses of studies that evaluate health care interventions: Explanation and elaboration. *J. Clin. Epidemiol.* **2009**, *62*, e1-e34, 10.1016/j.jclinepi.2009.06.006
25. Hidalgo, B.; Goodman, M. Multivariate or multivariable regression? *Am. J. Public Health*. **2013**, *103*, 39-40, 10.2105/ajph.2012.300897
26. Ebrahimi Kalan, M.; Jebai, R.; Zarafshan, E.; Bursac, Z. Distinction between two statistical terms: Multivariable and multivariate logistic regression. *Nicotine Tob. Res.* **2020**, *23*, 1446-1447, 10.1093/ntr/ntaa055
27. Katz, M. H. Multivariable analysis: A primer for readers of medical research. *Ann. Intern. Med.* **2003**, *138*, 644-650, 10.7326/0003-4819-138-8-200304150-00012
28. Chowdhury, M. Z. I.; Turin, T. C. Variable selection strategies and its importance in clinical prediction modelling. *Fam. Med. Community Health*. **2020**, *8*, e000262, 10.1136/fmch-2019-000262
29. Bursac, Z.; Gauss, C. H.; Williams, D. K.; Hosmer, D. W. Purposeful selection of variables in logistic regression. *Source Code Biol. Med.* **2008**, *3*, 17, 10.1186/1751-0473-3-17
30. Mickey, R. M.; Greenland, S. The impact of confounder selection criteria on effect estimation. *Am. J. Epidemiol.* **1989**, *129*, 125-137, 10.1093/oxfordjournals.aje.a115101
31. Riley, R. D.; Snell, K. I.; Ensor, J.; Burke, D. L.; Harrell, F. E., Jr.; Moons, K. G.; Collins, G. S. Minimum sample size for developing a multivariable prediction model: PART II - binary and time-to-event outcomes. *Stat. Med.* **2019**, *38*, 1276-1296, 10.1002/sim.7992
32. Riley, R. D.; Snell, K. I. E.; Ensor, J.; Burke, D. L.; Harrell, F. E., Jr.; Moons, K. G. M.; Collins, G. S. Minimum sample size for developing a multivariable prediction model: Part I - Continuous outcomes. *Stat. Med.* **2019**, *38*, 1262-1275, 10.1002/sim.7993
33. VanVoorhis, C. R. W.; Morgan, B. L. Understanding power and rules of thumb for determining sample sizes. 2007; 2007.
34. Ogundimu, E. O.; Altman, D. G.; Collins, G. S. Adequate sample size for developing prediction models is not simply related to events per variable. *J. Clin. Epidemiol.* **2016**, *76*, 175-182, 10.1016/j.jclinepi.2016.02.031
35. van Smeden, M.; Moons, K. G.; de Groot, J. A.; Collins, G. S.; Altman, D. G.; Eijkemans, M. J.; Reitsma, J. B. Sample size for binary logistic prediction models: Beyond events per variable criteria. *Stat. Methods Med. Res.* **2019**, *28*, 2455-2474, 10.1177/0962280218784726
36. van Smeden, M.; de Groot, J. A.; Moons, K. G.; Collins, G. S.; Altman, D. G.; Eijkemans, M. J.; Reitsma, J. B. No rationale for 1 variable per 10 events criterion for binary logistic regression analysis. *BMC Med. Res. Methodol.* **2016**, *16*, 163, 10.1186/s12874-016-0267-3

37. Vittinghoff, E.; McCulloch, C. E. Relaxing the rule of ten events per variable in logistic and Cox regression. *Am. J. Epidemiol.* **2007**, *165*, 710-718, 10.1093/aje/kwk052
38. Shea, B. J.; Reeves, B. C.; Wells, G.; Thuku, M.; Hamel, C.; Moran, J.; Moher, D.; Tugwell, P.; Welch, V.; Kristjansson, E.; et al. AMSTAR 2: A critical appraisal tool for systematic reviews that include randomised or non-randomised studies of healthcare interventions, or both. *BMJ.* **2017**, *358*, j4008, 10.1136/bmj.j4008
39. Higgins, J.; Savović, J.; Page, M.; Elbers, R.; Sterne, J. Chapter 8: Assessing risk of bias in a randomized trial. In: Higgins JPT, Thomas J, Chandler J, Cumpston M, Li T, Page MJ, Welch VA (editors). Cochrane handbook for systematic reviews of interventions version 6.1 (updated September 2020). Cochrane, 2020. Available from: https:// training.cochrane.org/handbook. 2020.
40. Higgins, J.; Thomas, J.; Chandler, J.; Cumpston, M.; Li, T.; Page, M.; Welch, V. Cochrane handbook for systematic reviews of interventions version 6.1 [Internet]. London (UK): Cochrane; 2020 Sept [cited 2021 Aug 13]. Available from: https://training.cochrane.org/handbook. [Note: version 6.3, 2022 was subsequently released].
41. Higgins, J.; Sterne, J.; Savović, J.; Page, M.; Hróbjartsson, A.; Boutron, I.; Reeves, B.; Eldridge, S. A revised tool for assessing risk of bias in randomized trials. In: Chandler J, McKenzie J, Boutron I, Welch V (editors). Cochrane Methods. Cochrane Database of Systematic Reviews 2016, Issue 10 (Suppl 1). https:// doi.org/10.1002/14651858.CD201601.;
42. Sterne, J.; Higgins, J.; Elers, R.; Reeves, B.; and the development group for ROBINS-I. Risk of bias in non-randomized studies of interventions (ROBINS-I): Detailed guidance [Internet]. Updated 2016 Oct 12. [cited 2021 Jul 16]. Available from: http://www.riskofbias.info
43. Sterne, J. A.; Hernán, M. A.; Reeves, B. C.; Savović, J.; Berkman, N. D.; Viswanathan, M.; Henry, D.; Altman, D. G.; Ansari, M. T.; Boutron, I.; et al. ROBINS-I: A tool for assessing risk of bias in non-randomised studies of interventions. *BMJ.* **2016**, *355*, i4919 (4917 pp), 10.1136/bmj.i4919
44. Sterne, J. A. C.; Savović, J.; Page, M. J.; Elbers, R. G.; Blencowe, N. S.; Boutron, I.; Cates, C. J.; Cheng, H.-Y.; Corbett, M. S.; Eldridge, S. M.; et al. RoB 2: A revised tool for assessing risk of bias in randomised trials. *BMJ.* **2019**, *366*, l4898, 10.1136/ bmj.l4898
45. Ontario Health (Cancer Care Ontario); Program in Evidence-Based Care, McMaster University. Program in Evidence-Based Care handbook, June 2020. Hamilton: Program in Evidence-Based Care. 2022 June (accessed 2024 Dec 2). Available from: https://www.cancercareontario.ca/sites/ccocancercare/files/assets/CCOPEBCHandbo ok.pdf.
46. Program in Evidence-Based Care, McMaster University. PEBC toolkit: CCO/PEBC methods handbook. Hamilton: Program in Evidence-Based Care. Accessed 2024 Dec 2. Available from: https://pebctoolkit.mcmaster.ca/doku.php?id=projectdev:pebc_methods_handbook&.
47. Brouwers, M. C.; Kho, M. E.; Browman, G. P.; Burgers, J. S.; Cluzeau, F.; Feder, G.; Fervers, B.; Graham, I. D.; Grimshaw, J.; Hanna, S. E.; et al. AGREE II: Advancing guideline development, reporting and evaluation in health care. *CMAJ.* **2010**, *182*, E839-842, 10.1503/cmaj.090449
48. The Cochrane Collaboration. Review Manager (RevMan) [Computer program on internet]. Version 5.4. London (UK): Cochrane; 2020 [cited 2021 May 6]. Available from: https://training.cochrane.org/online-learning/core-software-cochrane-reviews/revman/revman-5-download.

49. McKenzie, D. P.; Thomas, C. Relative risks and odds ratios: Simple rules on when and how to use them. *Eur. J. Clin. Invest.* **2020**, *50*, e13249 (13246 pp), https://doi.org/10.1111/eci.13249
50. Zhang, J.; Yu, K. F. What's the relative risk? A method of correcting the odds ratio in cohort studies of common outcomes. *JAMA.* **1998**, *280*, 1690-1691, 10.1001/jama.280.19.1690
51. Doi, S. A.; Furuya-Kanamori, L.; Xu, C.; Lin, L.; Chivese, T.; Thalib, L. Controversy and debate: Questionable utility of the relative risk in clinical research: Paper 1: A call for change to practice. *J. Clin. Epidemiol.* **2022**, *142*, 271-279,
52. Ranganathan, P.; Aggarwal, R.; Pramesh, C. S. Common pitfalls in statistical analysis: Odds versus risk. *Perspect. Clin. Res.* **2015**, *6*, 222-224, 10.4103/2229-3485.167092
53. Gentilucci, M.; Mazzocchi, M.; Alfano, C. Effects of prophylactic lipofilling after radiotherapy compared to non-fat injected breasts: A randomized, objective study. *Aesthet. Surg. J.* **2020**, *40*, NP597-NP607, https://dx.doi.org/10.1093/asj/sjaa182
54. Wederfoort, J. L. M.; Schop, S.; van der Broeck, L. C. A.; Hommes, J. E.; van Kuijk, S. M. J.; Timmermans, F.; Smit, J. M.; Heuts, E. M.; de Wit, T.; van der Hulst, R.; et al. Superior sensibility after full breast reconstruction with autologous fat transfer. *Plast. Reconstr. Surg.* **2024**, *153*, 316-323, 10.1097/prs.0000000000010619
55. Piatkowski, A. A.; Wederfoort, J. L. M.; Hommes, J. E.; Schop, S. S. J.; Krastev, T. K.; van Kuijk, S. M. J.; van der Hulst, R. Effect of total breast reconstruction with autologous fat transfer using an expansion device vs implants on quality of life among patients with breast cancer: A randomized clinical trial. *JAMA Surg.* **2023**, *158*, 456-464, 10.1001/jamasurg.2022.7625
56. Wederfoort, J. L. M.; Kleeven, A.; Hommes, J. E.; Van Kuijk, S. M. J.; van der Hulst, R.; Piatkowski, A.; investigators, M. D. f. T. B. t. Aesthetic evaluation of breast reconstruction with autologous fat transfer vs. Implants. *Aesthetic Plast. Surg.* **2023**, *47*, 593-604, 10.1007/s00266-022-03076-2
57. Schop, S. S. J.; Hommes, J. E.; Krastev, T. K.; Derks, D.; Larsen, M.; Rakhorst, H.; Schmidbauer, U.; Smit, J. M.; Tan, T.; Wehrens, K.; et al. BREAST trial study protocol: Evaluation of a non-invasive technique for breast reconstruction in a multicentre, randomised controlled trial. *BMJ Open.* **2021**, *11*, e051413, https://dx.doi.org/10.1136/bmjopen-2021-051413
58. Bennett, K. G.; Qi, J.; Kim, H. M.; Hamill, J. B.; Wilkins, E. G.; Mehrara, B. J.; Kozlow, J. H. Association of fat grafting with patient-reported outcomes in postmastectomy breast reconstruction. *JAMA Surg.* **2017**, *152*, 944-950, https://dx.doi.org/10.1001/jamasurg.2017.1716
59. Wilkins, E. G. The Mastectomy Reconstruction Outcomes Consortium (MROC) Study. ClinicalTrials.gov NCT01723423. Last updated 2017 July 13; cited 2024 Jul 12. Available from: https://clinicaltrials.gov/study/NCT01723423.
60. Sinha, I.; Pusic, A. L.; Wilkins, E. G.; Hamill, J. B.; Chen, X.; Kim, H. M.; Guldbrandsen, G.; Chun, Y. S. Late surgical-site infection in immediate implant-based breast reconstruction. *Plast. Reconstr. Surg.* **2017**, *139*, 20-28, https://dx.doi.org/10.1097/PRS.0000000000002839
61. Pusic, A. L.; Matros, E.; Fine, N.; Buchel, E.; Gordillo, G. M.; Hamill, J. B.; Kim, H. M.; Qi, J.; Albornoz, C.; Klassen, A. F.; et al. Patient-reported outcomes 1 year after immediate breast reconstruction: Results of the Mastectomy Reconstruction Outcomes Consortium Study. *J. Clin. Oncol.* **2017**, *35*, 2499-2506, https://dx.doi.org/10.1200/JCO.2016.69.9561
62. Wilkins, E. G.; Cederna, P. S.; Lowery, J. C.; Davis, J. A.; Kim, H. M.; Roth, R. S.; Goldfarb, S.; Izenberg, P. H.; Houin, H. P.; Shaheen, K. W. Prospective analysis of

psychosocial outcomes in breast reconstruction: One-year postoperative results from the Michigan Breast Reconstruction Outcome Study. *Plast. Reconstr. Surg.* **2000**, *106*, 1014-1025; discussion 1026-1017,
63. Yoon, A. P.; Qi, J.; Brown, D. L.; Kim, H. M.; Hamill, J. B.; Erdmann-Sager, J.; Pusic, A. L.; Wilkins, E. G. Outcomes of immediate versus delayed breast reconstruction: Results of a multicenter prospective study. *Breast.* **2018**, *37*, 72-79, https://dx.doi.org/10.1016/j.breast.2017.10.009
64. Voineskos, S. H.; Klassen, A. F.; Cano, S. J.; Pusic, A. L.; Gibbons, C. J. Giving meaning to differences in BREAST-Q scores: Minimal important difference for breast reconstruction patients. *Plast. Reconstr. Surg.* **2020**, *145*, 11e-20e, https://dx.doi.org/10.1097/PRS.0000000000006317
65. Kulkarni, A. R.; Pusic, A. L.; Hamill, J. B.; Kim, H. M.; Qi, J.; Wilkins, E. G.; Roth, R. S. Factors associated with acute postoperative pain following breast reconstruction. *JPRAS Open.* **2017**, *11*, 1-13, https://dx.doi.org/10.1016/j.jpra.2016.08.005
66. Wilkins, E. G.; Hamill, J. B.; Kim, H. M.; Kim, J. Y.; Greco, R. J.; Qi, J.; Pusic, A. L. Complications in postmastectomy breast reconstruction: One-year outcomes of the Mastectomy Reconstruction Outcomes Consortium (MROC) Study. *Ann. Surg.* **2018**, *267*, 164-170, https://dx.doi.org/10.1097/SLA.0000000000002033
67. Billig, J.; Jagsi, R.; Qi, J.; Hamill, J. B.; Kim, H. M.; Pusic, A. L.; Buchel, E.; Wilkins, E. G.; Momoh, A. O. Should immediate autologous breast reconstruction be considered in women who require postmastectomy radiation therapy? A prospective analysis of outcomes. *Plast. Reconstr. Surg.* **2017**, *139*, 1279-1288, https://dx.doi.org/10.1097/PRS.0000000000003331
68. Yoon, A. P.; Qi, J.; Kim, H. M.; Hamill, J. B.; Jagsi, R.; Pusic, A. L.; Wilkins, E. G.; Kozlow, J. H. Patient-reported outcomes after irradiation of tissue expander versus permanent implant in breast reconstruction: A multicenter prospective study. *Plast. Reconstr. Surg.* **2020**, *145*, 917e-926e, https://dx.doi.org/10.1097/PRS.0000000000006724
69. Sorkin, M.; Qi, J.; Kim, H. M.; Hamill, J. B.; Kozlow, J. H.; Pusic, A. L.; Wilkins, E. G. Acellular dermal matrix in immediate expander/implant breast reconstruction: A multicenter assessment of risks and benefits. *Plast. Reconstr. Surg.* **2017**, *140*, 1091-1100, https://dx.doi.org/10.1097/PRS.0000000000003842
70. Ganesh Kumar, N.; Berlin, N. L.; Kim, H. M.; Hamill, J. B.; Kozlow, J. H.; Wilkins, E. G. Development of an evidence-based approach to the use of acellular dermal matrix in immediate expander-implant-based breast reconstruction. *J. Plast. Reconstr. Aesthet. Surg.* **2021**, *74*, 30-40, https://dx.doi.org/10.1016/j.bjps.2020.10.005
71. Panayi, A. C.; Agha, R. A.; Sieber, B. A.; Orgill, D. P. Impact of obesity on outcomes in breast reconstruction: A systematic review and meta-analysis. *J. Reconstr. Microsurg.* **2018**, *34*, 363-375, https://dx.doi.org/10.1055/s-0038-1627449
72. Tan, M. Y. L.; Onggo, J.; Serag, S.; Phan, K.; Dusseldorp, J. R. Deep inferior epigastric perforator (DIEP) flap safety profile in slim versus non-slim BMI patients: A systematic review and meta-analysis. *J. Plast. Reconstr. Aesthet. Surg.* **2022**, *75*, 2180-2189, https://dx.doi.org/10.1016/j.bjps.2022.04.046
73. ElAbd, R.; Prabhu, N.; Alibrahim, A.; Burke, E.; Williams, J.; Samargandi, O. Autologous versus alloplastic reconstruction for patients with obesity: A systematic review and meta-analysis. *Aesthetic Plast. Surg.* **2022**, *46*, 597-609, https://dx.doi.org/10.1007/s00266-021-02664-y
74. Liu, Q.; Aggarwal, A.; Wu, M.; Darwish, O. A.; Baldino, K.; Haug, V.; Agha, R. A.; Orgill, D. P.; Panayi, A. C. Impact of diabetes on outcomes in breast reconstruction: A

systematic review and meta-analysis. *J. Plast. Reconstr. Aesthet. Surg.* **2022**, *75*, 1793-1804, https://dx.doi.org/10.1016/j.bjps.2022.02.053
75. Mortada, H.; Alwadai, A.; Bamakhrama, B.; Alsinan, T.; Hanawi, M. D.; Alfaryan, S. M.; Obeid, F. M.; Arab, K. The impact of diabetes mellitus on breast reconstruction outcomes and complications: A systematic literature review and meta-analysis. *Aesthetic Plast. Surg.* **2023**, *23*, 23, https://dx.doi.org/10.1007/s00266-023-03258-6
76. Theocharidis, V.; Katsaros, I.; Sgouromallis, E.; Serifis, N.; Boikou, V.; Tasigiorgos, S.; Kokosis, G.; Economopoulos, K. P. Current evidence on the role of smoking in plastic surgery elective procedures: A systematic review and meta-analysis. *J. Plast. Reconstr. Aesthet. Surg.* **2018**, *71*, 624-636, https://dx.doi.org/10.1016/j.bjps.2018.01.011
77. Mrad, M. A.; Al Qurashi, A. A.; Shah Mardan, Q. N. M.; Alqarni, M. D.; Alhenaki, G. A.; Alghamdi, M. S.; Fathi, A. B.; Alobaidi, H. A.; Alnamlah, A. A.; Aljehani, S. K.; et al. Predictors of complications after breast reconstruction surgery: A systematic review and meta-analysis. *Plast. Reconstr. Surg. Glob. Open.* **2022**, *10*, e4693. https://dx.doi.org/4610.1097/GOX.0000000000004693, https://dx.doi.org/10.1097/GOX.0000000000004693
78. Chung, J. H.; Sohn, S. M.; Jung, S. P.; Park, S. H.; Yoon, E. S. Effects of pre-existing abdominal scar on postoperative complications after autologous breast reconstruction using abdominal flaps: A systematic review and meta-analysis. *J. Plast. Reconstr. Aesthet. Surg.* **2021**, *74*, 277-289, https://dx.doi.org/10.1016/j.bjps.2020.11.020
79. Bond, E. S.; Soteropulos, C. E.; Yang, Q.; Poore, S. O. The impact of prior abdominal surgery on complications of abdominally based autologous breast reconstruction: A systematic review and meta-analysis. *J. Reconstr. Microsurg.* **2021**, *37*, 566-579, https://dx.doi.org/10.1055/s-0041-1723816
80. Chicco, M.; Ahmadi, A. R.; Cheng, H. T. Systematic review and meta-analysis of complications following mastectomy and prosthetic reconstruction in patients with and without prior breast augmentation. *Aesthet. Surg. J.* **2021**, *41*, NP763-NP770, https://dx.doi.org/10.1093/asj/sjab028
81. Varghese, J.; Gohari, S. S.; Rizki, H.; Faheem, M.; Langridge, B.; Kummel, S.; Johnson, L.; Schmid, P. A systematic review and meta-analysis on the effect of neoadjuvant chemotherapy on complications following immediate breast reconstruction. *Breast.* **2021**, *55*, 55-62, https://dx.doi.org/10.1016/j.breast.2020.11.023
82. Spera, L. J.; Cook, J. A.; Dolejs, S.; Fisher, C.; Lester, M. E.; Hassanein, A. H. Perioperative use of antiestrogen therapies in breast reconstruction: A systematic review and treatment recommendations. *Ann. Plast. Surg.* **2020**, *85*, 448-455, https://dx.doi.org/10.1097/SAP.0000000000002394
83. Hong, W. J.; Zhang, G. Y.; Chen, C. L.; Li, F. W.; Wang, H. B. The effect of previous irradiation for patients with prosthetic breast reconstruction: A meta-analysis. *Aesthet. Surg. J.* **2021**, *41*, NP748-NP757, https://dx.doi.org/10.1093/asj/sjaa372
84. Zugasti, A.; Hontanilla, B. The impact of adjuvant radiotherapy on immediate implant-based breast reconstruction surgical and satisfaction outcomes: A systematic review and meta-analysis. *Plast. Reconstr. Surg. Glob. Open.* **2021**, *9*, e3910, https://dx.doi.org/10.1097/GOX.0000000000003910
85. Magill, L. J.; Robertson, F. P.; Jell, G.; Mosahebi, A.; Keshtgar, M. Determining the outcomes of post-mastectomy radiation therapy delivered to the definitive implant in patients undergoing one- and two-stage implant-based breast reconstruction: A systematic review and meta-analysis. *J. Plast. Reconstr. Aesthet. Surg.* **2017**, *70*, 1329-1335, https://dx.doi.org/10.1016/j.bjps.2017.05.057
86. Kim, M.; Ali, B.; Zhang, K.; Vingan, P.; Boe, L.; Ly, C. L.; Allen, R. J., Jr.; Stern, C. S.; Matros, E.; Cordeiro, P. G.; et al. Age impacts clinical and patient reported outcomes

following post mastectomy breast reconstruction. *Plast. Reconstr. Surg.* **2024**, *21*, 21, https://dx.doi.org/10.1097/PRS.0000000000011554
87. Honig, S. E.; Habarth-Morales, T. E.; Davis, H. D.; Niu, E. F.; Amro, C.; Broach, R. B.; Serletti, J. M.; Azoury, S. C. Increased patient age as a risk factor following free flap reconstruction after breast cancer: A single institutional review of 2,598 cases. *J. Reconstr. Microsurg.* **2024**, *12*, 12, https://dx.doi.org/10.1055/s-0044-1787728
88. Santosa, K. B.; Qi, J.; Kim, H. M.; Hamill, J. B.; Pusic, A. L.; Wilkins, E. G. Effect of patient age on outcomes in breast reconstruction: Results from a multicenter prospective study. *J. Am. Coll. Surg.* **2016**, *223*, 745-754, https://dx.doi.org/10.1016/j.jamcollsurg.2016.09.003
89. Chang, E. I.; Vaca, L.; DaLio, A. L.; Festekjian, J. H.; Crisera, C. A. Assessment of advanced age as a risk factor in microvascular breast reconstruction. *Ann. Plast. Surg.* **2011**, *67*, 255-259, https://dx.doi.org/10.1097/SAP.0b013e3181f9b20c
90. Cuccolo, N. G.; Sparenberg, S.; Ibrahim, A. M. S.; Crystal, D. T.; Blankensteijn, L. L.; Lin, S. J. Does age or frailty have more predictive effect on outcomes following pedicled flap reconstruction? An analysis of 44,986 cases. *J. Plast. Surg. Hand Surg.* **2020**, *54*, 67-76, https://dx.doi.org/10.1080/2000656X.2019.1688166
91. Butz, D. R.; Lapin, B.; Yao, K.; Wang, E.; Song, D. H.; Johnson, D.; Sisco, M. Advanced age is a predictor of 30-day complications after autologous but not implant-based postmastectomy breast reconstruction. *Plast. Reconstr. Surg.* **2015**, *135*, 253e-261e, https://dx.doi.org/10.1097/PRS.0000000000000988
92. Jeevan, R.; Cromwell, D. A.; Browne, J. P.; Caddy, C. M.; Pereira, J.; Sheppard, C.; Greenaway, K.; van der Meulen, J. H. Findings of a national comparative audit of mastectomy and breast reconstruction surgery in England. *J. Plast. Reconstr. Aesthet. Surg.* **2014**, *67*, 1333-1344, https://dx.doi.org/10.1016/j.bjps.2014.04.022
93. Jeevan, R.; Cromwell, D.; Browne, J.; van der Meulen, J.; Pereira, J.; Caddy, C.; Sheppard, C.; Greenaway, K.; Napper, R.; Dean, S. National Mastectomy and Breast Reconstruction Audit 2011. A national audit of provision and outcomes of mastectomy and breast reconstruction surgery for women in England. Fourth Annual Report 2011. Leeds: The NHS Information Centre; 2011. Cited 2024 Jul 4. Available from: https://associationofbreastsurgery.org.uk/media/1085/nmbra-annual-report-2011.pdf and https://files.digital.nhs.uk/publicationimport/pub02xxx/pub02731/clin-audi-supp-prog-mast-brea-reco-2011-rep1.pdf.
94. Knoedler, S.; Kauke-Navarro, M.; Knoedler, L.; Friedrich, S.; Ayyala, H. S.; Haug, V.; Didzun, O.; Hundeshagen, G.; Bigdeli, A.; Kneser, U.; et al. The significance of timing in breast reconstruction after mastectomy: An ACS-NSQIP analysis. *J. Plast. Reconstr. Aesthet. Surg.* **2024**, *89*, 40-50, https://dx.doi.org/10.1016/j.bjps.2023.11.049
95. Kroll, S. S.; Coffey, J. A., Jr.; Winn, R. J.; Schusterman, M. A. A comparison of factors affecting aesthetic outcomes of TRAM flap breast reconstructions. *Plast. Reconstr. Surg.* **1995**, *96*, 860-864,
96. Fosnot, J.; Fischer, J. P.; Smartt, J. M., Jr.; Low, D. W.; Kovach, S. J., 3rd; Wu, L. C.; Serletti, J. M. Does previous chest wall irradiation increase vascular complications in free autologous breast reconstruction? *Plast. Reconstr. Surg.* **2011**, *127*, 496-504, https://dx.doi.org/10.1097/PRS.0b013e3181fed560
97. Joosen, M. E. M.; Schop, S. J.; Reinhoudt, L. L.; van Kuijk, S. M. J.; Beugels, J.; de Bruine, A. P.; Goudkade, D.; Heuts, E. M.; van der Hulst, R.; de Grzymala, A. A. P. The difference in local, regional and distant breast cancer recurrence between the immediate and delayed DIEP flap procedure; a retrospective cohort study. *Breast Cancer Res. Treat.* **2021**, *188*, 389-398, https://dx.doi.org/10.1007/s10549-021-06199-3

98. Shammas, R. L.; Gordee, A.; Lee, H. J.; Sergesketter, A. R.; Scales, C. D.; Hollenbeck, S. T.; Phillips, B. T. Complications, costs, and healthcare resource utilization after staged, delayed, and immediate free-flap breast reconstruction: A longitudinal, claims-based analysis. *Ann. Surg. Oncol.* **2023**, *30*, 2534-2549, 10.1245/s10434-022-12896-0
99. Huang, H.; Chadab, T. M.; Wang, M. L.; Norman, S.; Cohen, L. E.; Otterburn, D. M. A comparison between immediate and babysitter deep inferior epigastric perforator flap breast reconstruction in postoperative outcomes. *Ann. Plast. Surg.* **2022**, *88*, S179-S183, https://dx.doi.org/10.1097/SAP.0000000000003078
100. Marquez, J. L.; Sudduth, J. D.; Kuo, K.; Patel, A. A.; Eddington, D.; Agarwal, J. P.; Kwok, A. C. A comparison of postoperative outcomes between immediate, delayed immediate, and delayed autologous free flap breast reconstruction: Analysis of 2010-2020 NSQIP Data. *J. Reconstr. Microsurg.* **2023**, *39*, 664-670, https://dx.doi.org/10.1055/a-2056-0909
101. Kalmar, C. L.; Montorfano, L.; Thayer, W. P.; Kassis, S.; Higdon, K. K.; Perdikis, G. Timing of autologous tissue breast reconstruction does not affect free flap failure. *Ann. Plast. Surg.* **2024**, *92*, 663-666, https://dx.doi.org/10.1097/SAP.0000000000003900
102. Ulrikh, D. G.; Krivorotko, P. V.; Bryantseva, Z. V.; Pesotskiy, R. S.; Bondarchuk, Y. I.; Amirov, N. S.; Enaldieva, D. A.; Tabagua, T. T.; Akulova, I. A.; Levchenko, V. E.; et al. Reconstructive plastic surgery in combined treatment of breast cancer: Predictive risk factors of complications and reconstruction failure. [Russian]. *P.A. Herzen J. Oncol.* **2024**, *13*, 13-19, https://dx.doi.org/10.17116/onkolog20241303113
103. Hassan, A. M.; Ray, N.; Govande, J. G.; Paidisetty, P.; Largo, R. D.; Chu, C. K.; Mericli, A. F.; Schaverien, M. V.; Clemens, M. W.; Hanasono, M. M.; et al. Long-term surgical and patient-reported outcomes comparing skin-preserving, staged versus delayed microvascular breast reconstruction. *Ann. Surg. Oncol.* **2023**, *30*, 5711-5722, https://dx.doi.org/10.1245/s10434-023-13470-y
104. Gabriel, A.; Sigalove, S.; Storm-Dickerson, T. L.; Sigalove, N. M.; Pope, N.; Rice, J.; Maxwell, G. P. Dual-plane versus prepectoral breast reconstruction in high-body mass index patients. *Plast. Reconstr. Surg.* **2020**, *145*, 1357-1365, https://dx.doi.org/10.1097/PRS.0000000000006840
105. Warren Peled, A.; Foster, R. D.; Garwood, E. R.; Moore, D. H.; Ewing, C. A.; Alvarado, M.; Hwang, E. S.; Esserman, L. J. The effects of acellular dermal matrix in expander-implant breast reconstruction after total skin-sparing mastectomy: Results of a prospective practice improvement study. *Plast. Reconstr. Surg.* **2012b**, *129*, 901e-908e, https://dx.doi.org/10.1097/PRS.0b013e31824ec447
106. Wang, F.; Warren Peled, A.; Garwood, E.; Fiscalini, A. S.; Sbitany, H.; Foster, R. D.; Alvarado, M.; Ewing, C.; Hwang, E. S.; Esserman, L. J. Total skin-sparing mastectomy and immediate breast reconstruction: An evolution of technique and assessment of outcomes. *Ann. Surg. Oncol.* **2014**, *21*, 3223-3230, https://dx.doi.org/10.1245/s10434-014-3915-z
107. Sbitany, H.; Piper, M.; Lentz, R. Prepectoral breast reconstruction: A safe alternative to submuscular prosthetic reconstruction following nipple-sparing mastectomy. *Plast. Reconstr. Surg.* **2017**, *140*, 432-443, https://dx.doi.org/10.1097/PRS.0000000000003627
108. Bettinger, L. N.; Waters, L. M.; Reese, S. W.; Kutner, S. E.; Jacobs, D. I. Comparative study of prepectoral and subpectoral expander-based breast reconstruction and Clavien IIIb score outcomes. *Plast. Reconstr. Surg. Glob. Open.* **2017**, *5*, e1433, https://dx.doi.org/10.1097/GOX.0000000000001433
109. Koesters, E. C.; Chang, D. W. Radiation and free flaps: What is the optimal timing? *Gland Surg.* **2023**, *12*, 1122-1130, https://dx.doi.org/10.21037/gs-23-154

110. Gerber, B.; Krause, A.; Reimer, T.; Muller, H.; Kuchenmeister, I.; Makovitzky, J.; Kundt, G.; Friese, K. Skin-sparing mastectomy with conservation of the nipple-areola complex and autologous reconstruction is an oncologically safe procedure. *Ann. Surg.* **2003**, *238*, 120-127,
111. Gerber, B.; Krause, A.; Dieterich, M.; Kundt, G.; Reimer, T. The oncological safety of skin sparing mastectomy with conservation of the nipple-areola complex and autologous reconstruction: An extended follow-up study. *Ann. Surg.* **2009**, *249*, 461-468, https://dx.doi.org/10.1097/SLA.0b013e31819a044f
112. Jeon, Y. S.; Kang, S. H.; Bae, Y. K.; Lee, S. J. The oncologic safety of skin sparing mastectomy with or without conservation of the nipple-areolar complex: 5 years follow up results. [Korean]. *J. Breast Cancer.* **2010**, *13(1)*, 65-73, https://dx.doi.org/10.4048/jbc.2010.13.1.65
113. Kim, H. J.; Park, E. H.; Lim, W. S.; Seo, J. Y.; Koh, B. S.; Lee, T. J.; Eom, J. S.; Lee, S. W.; Son, B. H.; Lee, J. W.; et al. Nipple areola skin-sparing mastectomy with immediate transverse rectus abdominis musculocutaneous flap reconstruction is an oncologically safe procedure: A single center study. *Ann. Surg.* **2010**, *251*, 493-498, https://dx.doi.org/10.1097/SLA.0b013e3181c5dc4e
114. Wang, M.; Huang, J.; Chagpar, A. B. Is nipple sparing mastectomy associated with increased complications, readmission and length of stay compared to skin sparing mastectomy? *Am. J. Surg.* **2020**, *219*, 1030-1035, https://dx.doi.org/10.1016/j.amjsurg.2019.09.011
115. Kelly, B. N.; Faulkner, H. R.; Smith, B. L.; Korotkin, J. E.; Lanahan, C. R.; Brown, C.; Gadd, M. A.; Specht, M. C.; Hughes, K. S.; Oseni, T. S.; et al. Nipple-sparing mastectomy versus skin-sparing mastectomy: Does saving the nipple impact short- and long-term patient satisfaction? *Ann. Surg. Oncol.* **2022**, *29*, 1033-1040, https://dx.doi.org/10.1245/s10434-021-10767-8
116. Racz, J. M.; Harless, C. A.; Hoskin, T. L.; Day, C. N.; Nguyen, M. T.; Harris, A. M.; Boughey, J. C.; Hieken, T. J.; Degnim, A. C. Sexual well-being after nipple-sparing mastectomy: Does preservation of the nipple matter? *Ann. Surg. Oncol.* **2022**, *06*, 06, https://dx.doi.org/10.1245/s10434-022-11578-1
117. Ogiya, A.; Nagura, N.; Shimo, A.; Nogi, H.; Narui, K.; Seki, H.; Mori, H.; Sasada, S.; Ishitobi, M.; Kondo, N.; et al. Long-term outcomes of breast cancer patients with local recurrence after mastectomy undergoing immediate breast reconstruction: A retrospective multi-institutional study of 4153 cases. *Ann. Surg. Oncol.* **2023**, *30*, 6532-6540, https://dx.doi.org/10.1245/s10434-023-13832-6
118. Cho, J. H.; Park, J. M.; Park, H. S.; Kim, H. J.; Shin, D. M.; Kim, J. Y.; Park, S.; Kim, S. I.; Park, B. W. Oncologic outcomes in nipple-sparing mastectomy with immediate reconstruction and total mastectomy with immediate reconstruction in women with breast cancer: A machine-learning analysis. *Ann. Surg. Oncol.* **2023**, *30*, 7281-7290, https://dx.doi.org/10.1245/s10434-023-13963-w
119. Sasada, S.; Nagura, N.; Shimo, A.; Ogiya, A.; Saiga, M.; Seki, H.; Mori, H.; Kondo, N.; Ishitobi, M.; Narui, K.; et al. Impact of radiation therapy for breast cancer with involved surgical margin after immediate breast reconstruction: A multi-institutional observational study. *Eur. J. Surg. Oncol.* **2024**, *50*, 108360, https://dx.doi.org/10.1016/j.ejso.2024.108360
120. Ryu, J. M.; Nam, S. J.; Kim, S. W.; Lee, S. K.; Bae, S. Y.; Yi, H. W.; Park, S.; Paik, H. J.; Lee, J. E. Feasibility of nipple-sparing mastectomy with immediate breast reconstruction in breast cancer patients with tumor-nipple distance less than 2.0 cm. *World J. Surg.* **2016**, *40*, 2028-2035, https://dx.doi.org/10.1007/s00268-016-3487-0

121. Alsharif, E.; Ryu, J. M.; Choi, H. J.; Nam, S. J.; Kim, S. W.; Yu, J.; Chae, B. J.; Lee, S. K.; Lee, J. E. Oncologic outcomes of nipple-sparing mastectomy with immediate breast reconstruction in patients with tumor-nipple distance less than 2.0 cm. *J. Breast Cancer*. **2019**, *22*, 613-623, https://dx.doi.org/10.4048/jbc.2019.22.e48
122. Balci, F. L.; Kara, H.; Dulgeroglu, O.; Uras, C. Oncologic safety of nipple-sparing mastectomy in patients with short tumor-nipple distance. *Breast J.* **2019**, *25*, 612-618, https://dx.doi.org/10.1111/tbj.13289
123. Fregatti, P.; Gipponi, M.; Zoppoli, G.; Lambertini, M.; Blondeaux, E.; Belgioia, L.; Derosa, R.; Murelli, F.; Depaoli, F.; Ceppi, M.; et al. Tumor-to-nipple distance should not preclude nipple-sparing mastectomy in breast cancer patients. Personal experience and literature review. *Anticancer Res.* **2020**, *40*, 3543-3550, https://dx.doi.org/10.21873/anticanres.14343
124. Kim, S.; Lee, S.; Bae, Y.; Lee, S. Nipple-sparing mastectomy for breast cancer close to the nipple: A single institution's 11-year experience. *Breast Cancer*. **2020**, *27*, 999-1006, https://dx.doi.org/10.1007/s12282-020-01104-0
125. Beller, F. K.; Schnepper, E. [Conservative primary operation of carcinoma of the breast: Subcutaneous mastectomy, lymphadenectomy and radiotherapy (author's transl)] konservative primäroperation des mammakarzinoms: Subkutane mastektomie, lymphadenektomie und bestrahlung. *Dtsch. Med. Wochenschr.* **1981**, *106*, 329-334,
126. Psaila, A.; Pozzi, M.; Barone Adesi, L.; Varanese, A.; Costantini, M.; Gullo, P.; Panimolle, M.; Pugliese, P.; Botti, C.; Di Filippo, F.; et al. Nipple sparing mastectomy with immediate breast reconstruction: A short term analysis of our experience. *J. Exp. Clin. Cancer Res.* **2006**, *25*, 309-312,
127. Benediktsson, K. P.; Perbeck, L. Survival in breast cancer after nipple-sparing subcutaneous mastectomy and immediate reconstruction with implants: A prospective trial with 13 years median follow-up in 216 patients. *Eur. J. Surg. Oncol.* **2008**, *34*, 143-148,
128. Boneti, C.; Yuen, J.; Santiago, C.; Diaz, Z.; Robertson, Y.; Korourian, S.; Westbrook, K. C.; Henry-Tillman, R. S.; Klimberg, V. S. Oncologic safety of nipple skin-sparing or total skin-sparing mastectomies with immediate reconstruction. *J. Am. Coll. Surg.* **2011**, *212*, 686-693; discussion 693-685, https://dx.doi.org/10.1016/j.jamcollsurg.2010.12.039
129. Fortunato, L.; Loreti, A.; Andrich, R.; Costarelli, L.; Amini, M.; Farina, M.; Santini, E.; Vitelli, C. E. When mastectomy is needed: Is the nipple-sparing procedure a new standard with very few contraindications? *J. Surg. Oncol.* **2013**, *108*, 207-212, https://dx.doi.org/10.1002/jso.23390
130. Sakurai, T.; Zhang, N.; Suzuma, T.; Umemura, T.; Yoshimura, G.; Sakurai, T.; Yang, Q. Long-term follow-up of nipple-sparing mastectomy without radiotherapy: A single center study at a Japanese institution. *Med. Oncol.* **2013**, *30*, 481, https://dx.doi.org/10.1007/s12032-013-0481-3
131. Stanec, Z.; Zic, R.; Budi, S.; Stanec, S.; Milanovic, R.; Vlajcic, Z.; Roje, Z.; Rudman, F.; Martic, K.; Held, R.; et al. Skin and nipple-areola complex sparing mastectomy in breast cancer patients: 15-year experience. *Ann. Plast. Surg.* **2014**, *73*, 485-491, https://dx.doi.org/10.1097/SAP.0b013e31827a30e6
132. Rossi, C.; Mingozzi, M.; Curcio, A.; Buggi, F.; Folli, S. Nipple areola complex sparing mastectomy. *Gland Surg.* **2015**, *4*, 528-540, https://dx.doi.org/10.3978/j.issn.2227-684X.2015.04.12
133. Santoro, S.; Loreti, A.; Cavaliere, F.; Costarelli, L.; La Pinta, M.; Manna, E.; Mauri, M.; Scavina, P.; Santini, E.; De Paula, U.; et al. Neoadjuvant chemotherapy is not a contraindication for nipple sparing mastectomy. *Breast*. **2015**, *24*, 661-666, https://dx.doi.org/10.1016/j.breast.2015.08.001

134. Seki, T.; Jinno, H.; Okabayashi, K.; Murata, T.; Matsumoto, A.; Takahashi, M.; Hayashida, T.; Kitagawa, Y. Comparison of oncological safety between nipple sparing mastectomy and total mastectomy using propensity score matching. *Ann. R. Coll. Surg. Engl.* **2015**, *97*, 291-297, https://dx.doi.org/10.1308/003588415X14181254788881
135. Fujimoto, H.; Ishikawa, T.; Satake, T.; Ko, S.; Shimizu, D.; Narui, K.; Yamada, A.; Sasaki, T.; Nagashima, T.; Endo, I.; et al. Donor site selection and clinical outcomes of nipple-areola skin-sparing mastectomy with immediate autologous free flap reconstruction: A single-institution experience. *Eur. J. Surg. Oncol.* **2016**, *42*, 369-375, https://dx.doi.org/10.1016/j.ejso.2015.12.002
136. Moo, T. A.; Pinchinat, T.; Mays, S.; Landers, A.; Christos, P.; Alabdulkareem, H.; Tousimis, E.; Swistel, A.; Simmons, R. Oncologic outcomes after nipple-sparing mastectomy. *Ann. Surg. Oncol.* **2016**, *23*, 3221-3225, https://dx.doi.org/10.1245/s10434-016-5366-1
137. Shimo, A.; Tsugawa, K.; Tsuchiya, S.; Yoshie, R.; Tsuchiya, K.; Uejima, T.; Kojima, Y.; Shimo, A.; Hayami, R.; Nishikawa, T.; et al. Oncologic outcomes and technical considerations of nipple-sparing mastectomies in breast cancer: Experience of 425 cases from a single institution. *Breast Cancer.* **2016**, *23*, 851-860, https://dx.doi.org/10.1007/s12282-015-0651-6
138. Tang, R.; Coopey, S. B.; Merrill, A. L.; Rai, U.; Specht, M. C.; Gadd, M. A.; Colwell, A. S.; Austen, W. G., Jr.; Brachtel, E. F.; Smith, B. L. Positive nipple margins in nipple-sparing mastectomies: Rates, management, and oncologic safety. *J. Am. Coll. Surg.* **2016**, *222*, 1149-1155, https://dx.doi.org/10.1016/j.jamcollsurg.2016.02.016
139. Smith, B. L.; Tang, R.; Rai, U.; Plichta, J. K.; Colwell, A. S.; Gadd, M. A.; Specht, M. C.; Austen, W. G., Jr.; Coopey, S. B. Oncologic safety of nipple-sparing mastectomy in women with breast cancer. *J. Am. Coll. Surg.* **2017**, *225*, 361-365, https://dx.doi.org/10.1016/j.jamcollsurg.2017.06.013
140. Coopey, S. B.; Tang, R.; Lei, L.; Freer, P. E.; Kansal, K.; Colwell, A. S.; Gadd, M. A.; Specht, M. C.; Austen, W. G., Jr.; Smith, B. L. Increasing eligibility for nipple-sparing mastectomy. *Ann. Surg. Oncol.* **2013**, *20*, 3218-3222, https://dx.doi.org/10.1245/s10434-013-3152-x
141. Agresti, R.; Sandri, M.; Gennaro, M.; Bianchi, G.; Maugeri, I.; Rampa, M.; Capri, G.; Carcangiu, M. L.; Trecate, G.; Riggio, E.; et al. Evaluation of local oncologic safety in nipple-areola complex-sparing mastectomy after primary chemotherapy: A propensity score-matched study. *Clin. Breast Cancer.* **2017**, *17*, 219-231, https://dx.doi.org/10.1016/j.clbc.2016.12.003
142. Cont, N. T.; Maggiorotto, F.; Martincich, L.; Rivolin, A.; Kubatzki, F.; Sgandurra, P.; Marocco, F.; Magistris, A.; Gatti, M.; Balmativola, D.; et al. Primary tumor location predicts the site of local relapse after nipple-areola complex (NAC) sparing mastectomy. *Breast Cancer Res. Treat.* **2017**, *165*, 85-95, https://dx.doi.org/10.1007/s10549-017-4312-7
143. Huang, J.; Mo, Q.; Zhuang, Y.; Qin, Q.; Huang, Z.; Mo, J.; Tan, Q.; Lian, B.; Cao, Y.; Qin, S.; et al. Oncological safety of nipple-sparing mastectomy in young patients with breast cancer compared with conventional mastectomy. *Oncol. Lett.* **2018**, *15*, 4813-4820, https://dx.doi.org/10.3892/ol.2018.7913
144. Dornellas de Barros, A. C. S.; Carvalho, H. A.; Andrade, F. E. M.; Nimir, C.; Sampaio, M. M. C.; Makdissi, F. B.; Mano, M. S. Mammary adenectomy followed by immediate reconstruction for treatment of patients with early-infiltrating breast carcinoma: A cohort study. *Sao Paulo Med. J.* **2019**, *137*, 336-342, https://dx.doi.org/10.1590/1516-3180.2018.0356220719

145. Ng, Y. Y.; Tan, V. K.; Pek, W. S.; Chang, J. H.; Sim, Y.; Ong, K. W.; Yong, W. S.; Madhukumar, P.; Wong, C. Y.; Ong, Y. S.; et al. Surgical and oncological safety of nipple-sparing mastectomy in an Asian population. *Breast Cancer*. **2019**, *26*, 165-171, https://dx.doi.org/10.1007/s12282-018-0908-y
146. Valero, M. G.; Muhsen, S.; Moo, T. A.; Zabor, E. C.; Stempel, M.; Pusic, A.; Gemignani, M. L.; Morrow, M.; Sacchini, V. S. Increase in utilization of nipple-sparing mastectomy for breast cancer: Indications, complications, and oncologic outcomes. *Ann. Surg. Oncol*. **2020**, *27*, 344-351, 10.1245/s10434-019-07948-x
147. Vladimir, S.; Milan, R.; Zoran, R.; Ferenc, V.; Dejan, L.; Mladen, D. Early postoperative complications and local relapses after nipple sparing mastectomy and immediate breast reconstruction using silicone implants. *Arch. Oncol*. **2019**, *25(1)*, 9-12, https://dx.doi.org/10.2298/AOO180701008S
148. Li, X.; Wang, Y. Skin- and nipple-areola-sparing mastectomy with immediate breast reconstruction using transverse rectus abdominis myocutaneous flap and silicone implants in breast carcinoma patients. *Oncol. Res. Treat*. **2020**, *43*, 354-361, https://dx.doi.org/10.1159/000506841
149. Metere, A.; Fabiani, E.; Lonardo, M. T.; Giannotti, D.; Pace, D.; Giacomelli, L. Nipple-sparing mastectomy long-term outcomes: Early and late complications. *Medicina*. **2020**, *56*, 08, https://dx.doi.org/10.3390/medicina56040166
150. Scardina, L.; A, D. I. L.; Sanchez, A. M.; D'Archi, S.; Biondi, E.; Franco, A.; Mason, E. J.; Magno, S.; Terribile, D.; Barone-Adesi, L.; et al. Nipple sparing mastectomy with prepectoral immediate prosthetic reconstruction without acellular dermal matrices: A single center experience. *Minerva Surg*. **2021**, *76*, 498-505, https://dx.doi.org/10.23736/S2724-5691.21.08998-X
151. Webster, A. J.; Shanno, J. N.; Santa Cruz, H. S.; Kelly, B. N.; Garstka, M.; Henriquez, A.; Specht, M. C.; Gadd, M. A.; Verdial, F. C.; Nguyen, A.; et al. Oncologic safety of nipple-sparing mastectomy for breast cancer in BRCA gene mutation carriers: Outcomes at 70 months median follow-up. *Ann. Surg. Oncol*. **2023**, *30*, 3215-3222, 10.1245/s10434-022-13006-w
152. Colwell, A. S.; Gadd, M.; Smith, B. L.; Austen, W. G., Jr. An inferolateral approach to nipple-sparing mastectomy: Optimizing mastectomy and reconstruction. *Ann. Plast. Surg*. **2010**, *65*, 140-143, https://dx.doi.org/10.1097/SAP.0b013e3181c1fe77
153. Zarba Meli, E.; De Santis, A.; Cortese, G.; Manna, E.; Mastropietro, T.; La Pinta, M.; Loreti, A.; Arelli, F.; Scavina, P.; Minelli, M.; et al. Nipple-sparing mastectomy after neoadjuvant chemotherapy: Definitive results with a long-term follow-up evaluation. *Ann. Surg. Oncol*. **2023**, *30*, 2163-2172, 10.1245/s10434-022-13035-5
154. Wu, Z. Y.; Kim, H. J.; Lee, J. W.; Chung, I. Y.; Kim, J. S.; Lee, S. B.; Son, B. H.; Eom, J. S.; Kim, S. B.; Gong, G. Y.; et al. Breast cancer recurrence in the nipple-areola complex after nipple-sparing mastectomy with immediate breast reconstruction for invasive breast cancer. *JAMA Surg*. **2019**, *154*, 1030-1037, https://dx.doi.org/10.1001/jamasurg.2019.2959
155. Wu, Z. Y.; Han, H. H.; Kim, H. J.; Lee, J.; Chung, I. Y.; Kim, J.; Lee, S.; Han, J.; Eom, J. S.; Kim, S. B.; et al. Locoregional recurrence following nipple-sparing mastectomy with immediate breast reconstruction: Patterns and prognostic significance. *Eur. J. Surg. Oncol*. **2021a**, *47*, 1309-1315, https://dx.doi.org/10.1016/j.ejso.2021.01.006
156. Wu, Z. Y.; Han, H. H.; Kim, H. J.; Lee, J.; Chung, I. Y.; Kim, J.; Lee, S.; Eom, J. S.; Kim, S. B.; Gong, G.; et al. Data on distant metastasis and survival after locoregional recurrence following nipple-sparing mastectomy and immediate breast reconstruction. *Data Brief*. **2021b**, *35*, 106837, https://dx.doi.org/10.1016/j.dib.2021.106837

157. Wu, Z. Y.; Han, H. H.; Han, J.; Kim, H. J.; Lee, J.; Chung, I. Y.; Kim, J.; Lee, S.; Eom, J. S.; Kim, S. B.; et al. Impact of local breast cancer recurrence on reconstructed breast in nipple-sparing mastectomy with immediate reconstruction. *J. Plast. Reconstr. Aesthet. Surg.* **2022b**, *75*, 2535-2541, https://dx.doi.org/10.1016/j.bjps.2021.05.030
158. Wu, Z. Y.; Kim, H. J.; Lee, J. W.; Chung, I. Y.; Kim, J. S.; Lee, S. B.; Son, B. H.; Eom, J. S.; Kim, S. B.; Gong, G. Y.; et al. Oncologic outcomes of nipple-sparing mastectomy and immediate reconstruction after neoadjuvant chemotherapy for breast cancer. *Ann. Surg.* **2021d**, *274*, e1196-e1201, https://dx.doi.org/10.1097/SLA.0000000000003798
159. Wu, Z. Y.; Han, H. H.; Kim, H. J.; Chung, I. Y.; Kim, J.; Lee, S. B.; Son, B. H.; Eom, J. S.; Kim, S. B.; Ahn, J. H.; et al. A propensity score-matched analysis of long-term oncologic outcomes after nipple-sparing versus conventional mastectomy for locally advanced breast cancer. *Ann. Surg.* **2022a**, *276*, 386-390, https://dx.doi.org/10.1097/SLA.0000000000004416
160. Petit, J. Y.; Veronesi, U.; Orecchia, R.; Rey, P.; Martella, S.; Didier, F.; Viale, G.; Veronesi, P.; Luini, A.; Galimberti, V.; et al. Nipple sparing mastectomy with nipple areola intraoperative radiotherapy: One thousand and one cases of a five years experience at the European Institute of Oncology of Milan (EIO). *Breast Cancer Res. Treat.* **2009**, *117*, 333-338, https://dx.doi.org/10.1007/s10549-008-0304-y
161. Lohsiriwat, V.; Martella, S.; Rietjens, M.; Botteri, E.; Rotmensz, N.; Mastropasqua, M. G.; Garusi, C.; De Lorenzi, F.; Manconi, A.; Sommario, M.; et al. Paget's disease as a local recurrence after nipple-sparing mastectomy: Clinical presentation, treatment, outcome, and risk factor analysis. *Ann. Surg. Oncol.* **2012**, *19*, 1850-1855, https://dx.doi.org/10.1245/s10434-012-2226-5
162. Lohsiriwat, V.; Rotmensz, N.; Botteri, E.; Intra, M.; Veronesi, P.; Martella, S.; Garusi, C.; De Lorenzi, F.; Manconi, A.; Lomeo, G.; et al. Do clinicopathological features of the cancer patient relate with nipple areolar complex necrosis in nipple-sparing mastectomy? *Ann. Surg. Oncol.* **2013**, *20*, 990-996, https://dx.doi.org/10.1245/s10434-12-2677-8
163. Galimberti, V.; Morigi, C.; Bagnardi, V.; Corso, G.; Vicini, E.; Fontana, S. K. R.; Naninato, P.; Ratini, S.; Magnoni, F.; Toesca, A.; et al. Oncological outcomes of nipple-sparing mastectomy: A single-center experience of 1989 patients. *Ann. Surg. Oncol.* **2018**, *25*, 3849-3857, https://dx.doi.org/10.1245/s10434-018-6759-0
164. Vicini, E.; De Lorenzi, F.; Invento, A.; Corso, G.; Radice, D.; Bozzo, S.; Kahler Ribeiro Fontana, S.; Caldarella, P.; Veronesi, P.; Galimberti, V. Is nipple-sparing mastectomy indicated after previous breast surgery? A series of 387 institutional cases. *Plast. Reconstr. Surg.* **2021**, *148*, 21-30, https://dx.doi.org/10.1097/PRS.0000000000008097
165. Chirappapha, P.; Petit, J. Y.; Rietjens, M.; De Lorenzi, F.; Garusi, C.; Martella, S.; Barbieri, B.; Gottardi, A.; Andrea, M.; Giuseppe, L.; et al. Nipple sparing mastectomy: Does breast morphological factor related to necrotic complications? *Plast. Reconstr. Surg. Glob. Open.* **2014**, *2*, e99, https://dx.doi.org/10.1097/GOX.0000000000000038
166. Warren Peled, A.; Foster, R. D.; Stover, A. C.; Itakura, K.; Ewing, C. A.; Alvarado, M.; Hwang, E. S.; Esserman, L. J. Outcomes after total skin-sparing mastectomy and immediate reconstruction in 657 breasts. *Ann. Surg. Oncol.* **2012a**, *19*, 3402-3409, https://dx.doi.org/10.1245/s10434-012-2362-y
167. Amara, D.; Warren Peled, A.; Wang, F.; Ewing, C. A.; Alvarado, M.; Esserman, L. J. Tumor involvement of the nipple in total skin-sparing mastectomy: Strategies for management. *Ann. Surg. Oncol.* **2015**, *22*, 3803-3808, https://dx.doi.org/10.1245/s10434-015-4646-5
168. Warren Peled, A.; Wang, F.; Foster, R. D.; Alvarado, M.; Ewing, C. A.; Sbitany, H.; Esserman, L. J. Expanding the indications for total skin-sparing mastectomy: Is it safe

for patients with locally advanced disease? *Ann. Surg. Oncol.* **2016**, *23*, 87-91, https://dx.doi.org/10.1245/s10434-015-4734-6
169. Holland, M.; Wallace, A.; Viner, J.; Sbitany, H.; Piper, M. Safety of incision placement with nipple-sparing mastectomy and immediate prepectoral breast reconstruction. *Plast. Reconstr. Surg. Glob. Open.* **2023**, *11*, e4736, https://dx.doi.org/10.1097/GOX.0000000000004736
170. Frey, J. D.; Salibian, A. A.; Lee, J.; Harris, K.; Axelrod, D. M.; Guth, A. A.; Shapiro, R. L.; Schnabel, F. R.; Karp, N. S.; Choi, M. Oncologic trends, outcomes, and risk factors for locoregional recurrence: An analysis of tumor-to-nipple distance and critical factors in therapeutic nipple-sparing mastectomy. *Plast. Reconstr. Surg.* **2019**, *143*, 1575-1585, https://dx.doi.org/10.1097/PRS.0000000000005600
171. Salibian, A. A.; Bekisz, J. M.; Frey, J. D.; Thanik, V. D.; Levine, J. P.; Karp, N. S.; Choi, M. Comparing incision choices in immediate microvascular breast reconstruction after nipple-sparing mastectomy: Unique considerations to optimize outcomes. *Plast. Reconstr. Surg.* **2021**, *148*, 1173-1185, https://dx.doi.org/10.1097/PRS.0000000000008282
172. Boyd, C. J.; Salibian, A. A.; Bekisz, J. M.; Axelrod, D. M.; Guth, A. A.; Shapiro, R. L.; Schnabel, F. R.; Karp, N. S.; Choi, M. Long-term cancer recurrence rates following nipple-sparing mastectomy: A 10-year follow-up study. *Plast. Reconstr. Surg.* **2022**, *150*, 13S-19S, https://dx.doi.org/10.1097/PRS.0000000000009495
173. Radovanovic, Z.; Radovanovic, D.; Golubovic, A.; Ivkovic-Kapicl, T.; Bokorov, B.; Mandic, A. Early complications after nipple-sparing mastectomy and immediate breast reconstruction with silicone prosthesis: Results of 214 procedures. *Scand. J. Surg.* **2010**, *99*, 115-118,
174. Folli, S.; Curcio, A.; Buggi, F.; Mingozzi, M.; Lelli, D.; Barbieri, C.; Asioli, S.; Nava, M. B.; Falcini, F. Improved sub-areolar breast tissue removal in nipple-sparing mastectomy using hydrodissection. *Breast.* **2012**, *21*, 190-193, https://dx.doi.org/10.1016/j.breast.2011.09.010
175. Lee, K. T.; Pyon, J. K.; Bang, S. I.; Lee, J. E.; Nam, S. J.; Mun, G. H. Does the reconstruction method influence development of mastectomy flap complications in nipple-sparing mastectomy? *J. Plast. Reconstr. Aesthet. Surg.* **2013**, *66*, 1543-1550, https://dx.doi.org/10.1016/j.bjps.2013.06.032
176. Huston, T. L.; Small, K.; Swistel, A. J.; Dent, B. L.; Talmor, M. Nipple-sparing mastectomy via an inframammary fold incision for patients with scarring from prior lumpectomy. *Ann. Plast. Surg.* **2015**, *74*, 652-657, https://dx.doi.org/10.1097/SAP.0000000000000004
177. Ahn, S. J.; Woo, T. Y.; Lee, D. W.; Lew, D. H.; Song, S. Y. Nipple-areolar complex ischemia and necrosis in nipple-sparing mastectomy. *Eur. J. Surg. Oncol.* **2018**, *44*, 1170-1176, https://dx.doi.org/10.1016/j.ejso.2018.05.006
178. Pek, W. S.; Tan, B. K.; Ru Ng, Y. Y.; Kiak Mien Tan, V.; Rasheed, M. Z.; Kiat Tee Tan, B.; Ong, K. W.; Ong, Y. S. Immediate breast reconstruction following nipple-sparing mastectomy in an Asian population: Aesthetic outcomes and mitigating nipple-areolar complex necrosis. *Arch. Plast. Surg.* **2018**, *45*, 229-238, https://dx.doi.org/10.5999/aps.2017.01067
179. Radovanovic, Z.; Ranisavljevic, M.; Radovanovic, D.; Vicko, F.; Ivkovic-Kapicl, T.; Solajic, N. Nipple-sparing mastectomy with primary implant reconstruction: Surgical and oncological outcome of 435 breast cancer patients. *Breast Care.* **2018**, *13*, 373-378, https://dx.doi.org/10.1159/000489317
180. Pallara, T.; Cagli, B.; Fortunato, L.; Altomare, V.; Loreti, A.; Grasso, A.; Manna, E.; Persichetti, P. Direct-to-implant and 2-stage breast reconstruction after nipple sparing

mastectomy: Results of a retrospective comparison. *Ann. Plast. Surg.* **2019**, *83*, 392-395, https://dx.doi.org/10.1097/SAP.0000000000001893
181. Park, S.; Yoon, C.; Bae, S. J.; Cha, C.; Kim, D.; Lee, J.; Ahn, S. G.; Roh, T. S.; Kim, Y. S.; Jeong, J. Comparison of complications according to incision types in nipple-sparing mastectomy and immediate reconstruction. *Breast.* **2020**, *53*, 85-91, https://dx.doi.org/10.1016/j.breast.2020.06.009
182. Seki, H.; Sakurai, T.; Maeda, Y.; Oki, N.; Aoyama, M.; Yamaguchi, R.; Shimizu, K. Utility of the periareolar incision technique for breast reconstructive surgery in patients with breast cancer. *Surg. Today.* **2020**, *50*, 1008-1015, https://dx.doi.org/10.1007/s00595-20-01975-y
183. Najmiddinov, B.; Park, J. K.; Yoon, K. H.; Myung, Y.; Koh, H. W.; Lee, O. H.; Hoon, J. J.; Shin, H. C.; Kim, E. K.; Heo, C. Y. Conventional versus modified nipple sparing mastectomy in immediate breast reconstruction: Complications, aesthetic, and patient-reported outcomes. *Front. Surg.* **2022**, *9*, 1001019, https://dx.doi.org/10.3389/fsurg.2022.1001019
184. Lai, H. W.; Lee, Y. Y.; Chen, S. T.; Liao, C. Y.; Tsai, T. L.; Chen, D. R.; Lai, Y. C.; Kao, W. P.; Wu, W. P. Nipple-areolar complex (NAC) or skin flap ischemia necrosis post nipple-sparing mastectomy (NSM)-analysis of clinicopathologic factors and breast magnetic resonance imaging (MRI) features. *World J. Surg. Oncol.* **2023**, *21*, 23, https://dx.doi.org/10.1186/s12957-023-02898-x
185. Cadili, L.; Pao, J. S.; McKevitt, E.; Dingee, C.; Bazzarelli, A.; Warburton, R. Nipple margin assessment at the time of nipple-sparing mastectomy. *Can. J. Surg.* **2023**, *66*, E298-E303, https://dx.doi.org/10.1503/cjs.001922
186. Cavalcante, F. P.; Lima, T. O.; Alcantara, R.; Cardoso, A.; Ulisses, F.; Novita, G.; Zerwes, F.; Millen, E. Inframammary versus periareolar incision: A comparison of early complications in nipple-sparing mastectomy. *Plast. Reconstr. Surg. Glob. Open.* **2023**, *11*, e5367, https://dx.doi.org/10.1097/GOX.0000000000005367
187. Moo, T. A.; Nelson, J. A.; Sevilimedu, V.; Charyn, J.; Le, T. V.; Allen, R. J.; Mehrara, B. J.; Barrio, A. V.; Capko, D. M.; Pilewskie, M.; et al. Strategies to avoid mastectomy skin-flap necrosis during nipple-sparing mastectomy. *Br. J. Surg.* **2023**, *110*, 831-838, https://dx.doi.org/10.1093/bjs/znad107
188. Serio, F.; Manna, E.; La Pinta, M.; Arienzo, F.; Costarelli, L.; Zarba Meli, E.; Loreti, A.; Mastropietro, T.; Broglia, L.; Ascarelli, A.; et al. Intraoperative examination of retro-areolar margin is not routinely necessary during nipple-sparing mastectomy for cancer. *Ann. Surg. Oncol.* **2023**, *30*, 6488-6496, https://dx.doi.org/10.1245/s10434-023-13726-7
189. Black, G. G.; Chen, Y.; Qin, N.; Wang, M. L.; Huang, H.; Otterburn, D. M. An evolving landscape: Return of breast sensation after mastectomy varies by anatomic region and reconstructive method. *Ann. Plast. Surg.* **2024**, *92*, S91-S95, https://dx.doi.org/10.1097/SAP.0000000000003857
190. Golijanin, D.; Radovanovic, Z.; Radovanovic, D.; Dermanovic, A.; Starcevic, S.; Dermanovic, M. Molecular subtype and risk of local recurrence after nipple-sparing mastectomy for breast cancer. *Oncol. Lett.* **2024**, *28*, 389, https://dx.doi.org/10.3892/ol.2024.14522
191. Lin, A. M.; Lorenzi, R.; Van Der Hulst, J. E.; Liao, E. C.; Austen, W. G., Jr.; Webster, A.; Smith, B. L.; Colwell, A. S. A decade of nipple-sparing mastectomy: Lessons learned in 3035 immediate implant-based breast reconstructions. *Plast. Reconstr. Surg.* **2024**, *153*, 277-287, https://dx.doi.org/10.1097/PRS.0000000000010616
192. Liston, J. M.; Hollenbeck, S. T. Discussion: A decade of nipple-sparing mastectomy: Lessons learned in 3035 immediate implant-based breast reconstructions. *Plast.*

Reconstr. Surg. **2024**, *153(2)*, 288-289, https://dx.doi.org/10.1097/PRS.0000000000010715
193. Shanno, J. N.; Daly, A. E.; Anderman, K. J.; Santa Cruz, H. S.; Webster, A. J.; Pride, R. M.; Specht, M. C.; Gadd, M. A.; Oseni, T. O.; Verdial, F. C.; et al. Positive nipple margins in nipple-sparing mastectomy: Management of nipples containing cancer or atypia. *Ann. Surg. Oncol.* **2024**, *31*, 5148-5156, https://dx.doi.org/10.1245/s10434-024-15362-1
194. Nashimoto, M.; Asano, Y.; Matsui, H.; Machida, Y.; Hoshi, K.; Kurosumi, M.; Fukuma, E. Comparison of locoregional recurrence risk among nipple-sparing mastectomy, skin-sparing mastectomy, and simple mastectomy in patients with ductal carcinoma in situ: A single-center study. *Breast Cancer.* **2024**, *17*, 17, https://dx.doi.org/10.1007/s12282-24-01613-2
195. Sagir, M.; Guven, E.; Saylik, O.; Dulgeroglu, O.; Uras, C. A new convenient incision model of the nipple-sparing mastectomy: Lateralized parabolic multiplanar incision. *Aesthetic Plast. Surg.* **2024**, *20*, 20, https://dx.doi.org/10.1007/s00266-024-04115-w
196. Spoor, J.; Heeling, E.; Collewijn, R. C.; van der Ploeg, I. M. C.; Hoornweg, M. J.; Russell, N.; van den Berg, J. G.; Vrancken Peeters, M.; van Duijnhoven, F. H. Intraoperative frozen section of subareolar tissue in nipple-sparing mastectomy: Towards a less is more approach. *Eur. J. Surg. Oncol.* **2024**, *50*, 108320, https://dx.doi.org/10.1016/j.ejso.2024.108320
197. Braun, S. E.; Sinik, L. M.; Meyer, A. M.; Larson, K. E.; Butterworth, J. A. Predicting complications in breast reconstruction: Development and prospective validation of a machine learning model. *Ann. Plast. Surg.* **2023**, *91*, 282-286, https://dx.doi.org/10.1097/SAP.0000000000003621
198. Ponzone, R.; Maggiorotto, F.; Carabalona, S.; Rivolin, A.; Pisacane, A.; Kubatzki, F.; Renditore, S.; Carlucci, S.; Sgandurra, P.; Marocco, F.; et al. MRI and intraoperative pathology to predict nipple-areola complex (NAC) involvement in patients undergoing NAC-sparing mastectomy. *Eur. J. Cancer.* **2015**, *51*, 1882-1889, https://dx.doi.org/10.1016/j.ejca.2015.07.001
199. Sbitany, H.; Wang, F.; Peled, A. W.; Alvarado, M.; Ewing, C. A.; Esserman, L. J.; Foster, R. D. Tissue expander reconstruction after total skin-sparing mastectomy: Defining the effects of coverage technique on nipple/areola preservation. *Ann. Plast. Surg.* **2016**, *77*, 17-24, https://dx.doi.org/10.1097/SAP.0000000000000292
200. Robertson, S. A.; Jeevaratnam, J. A.; Agrawal, A.; Cutress, R. I. Mastectomy skin flap necrosis: Challenges and solutions. *Breast Cancer (Dove Med Press)).* **2017**, *9*, 141-152, https://dx.doi.org/10.2147/BCTT.S81712
201. Huang, H.; Wang, M. L.; Ellison, A.; Otterburn, D. M. Comparing autologous to device-based breast reconstruction: A pilot study of return in breast sensation. *Ann. Plast. Surg.* **2022**, *88*, S184-S189, https://dx.doi.org/10.1097/SAP.0000000000003073
202. Rusby, J. E.; Kirstein, L. J.; Brachtel, E. F.; Michaelson, J. S.; Koerner, F. C.; Smith, B. L. Nipple-sparing mastectomy: Lessons from ex vivo procedures. *Breast J.* **2008**, *14*, 464-470, https://dx.doi.org/10.1111/j.1524-4741.2008.00623.x
203. Suarez-Zamora, D. A.; Mustafa, R. A.; Estrada-Orozco, K.; Rodriguez-Urrego, P. A.; Torres-Franco, F.; Barreto-Hauzeur, L.; Mora-Ochoa, H.; Di Tanna, G. L.; Yepes-Nunez, J. J. Intraoperative sub-areolar frozen section analysis for detecting nipple involvement in candidates for nipple-sparing mastectomy. *Cochrane Database Syst. Rev.* **2021**, *2021(4) (no pagination)*, https://dx.doi.org/10.1002/14651858.CD014702
204. Hogan, K. O.; Lai, S. M.; Wagner, J. L.; Fan, F. The utility of intraoperative retroareolar margin frozen section assessment and the management of atypical epithelial proliferative lesions at the retroareolar margin in nipple-sparing mastectomies. *Ann.*

Diagn. Pathol. **2021**, *51*, 151697, https://dx.doi.org/10.1016/j.anndiagpath.2020.151697
205. Heinzen, R. N.; de Barros, A.; Carvalho, F. M.; Aguiar, F. N.; Nimir, C.; Jacomo, A. L. Nipple-sparing mastectomy for early breast cancer: The importance of intraoperative evaluation of retroareolar margins and intra-nipple duct removal. *Gland Surg.* **2020**, *9*, 637-646, https://dx.doi.org/10.21037/gs-20-405
206. D'Alonzo, M.; Pecchio, S.; Campisi, P.; De Rosa, G.; Bounous, V. E.; Villasco, A.; Balocco, P.; Biglia, N. Nipple-sparing mastectomy: Reliability of sub-areolar sampling and frozen section in predicting occult nipple involvement in breast cancer patients. *Eur. J. Surg. Oncol.* **2018**, *44*, 1736-1742, https://dx.doi.org/10.1016/j.ejso.2018.07.059
207. Suarez-Zamora, D. A.; Barrera-Herrera, L. E.; Palau-Lazaro, M. A.; Torres-Franco, F.; Orozco-Plazas, A.; Barreto-Hauzeur, L.; Rodriguez-Urrego, P. A. Accuracy and interobserver agreement of retroareolar frozen sections in nipple-sparing mastectomies. *Ann. Diagn. Pathol.* **2017**, *29*, 46-51, https://dx.doi.org/10.1016/j.anndiagpath.2017.05.001
208. Dent, B. L.; Chao, J. W.; Eden, D. J.; Stone, B. V.; Swistel, A.; Talmor, M. Nipple resection and reconstruction after attempted nipple-sparing mastectomy. *Ann. Plast. Surg.* **2017**, *78*, 28-34,
209. Alperovich, M.; Choi, M.; Karp, N. S.; Singh, B.; Ayo, D.; Frey, J. D.; Roses, D. F.; Schnabel, F. R.; Axelrod, D. M.; Shapiro, R. L.; et al. Nipple-sparing mastectomy and sub-areolar biopsy: To freeze or not to freeze? Evaluating the role of sub-areolar intraoperative frozen section. *Breast J.* **2016**, *22*, 18-23, https://dx.doi.org/10.1111/tbj.12517
210. Duarte, G. M.; Tomazini, M. V.; Oliveira, A.; Moreira, L.; Tocchet, F.; Worschech, A.; Torresan, R. Z. Accuracy of frozen section, imprint cytology, and permanent histology of sub-nipple tissue for predicting occult nipple involvement in patients with breast carcinoma. *Breast Cancer Res. Treat.* **2015**, *153(3)*, 557-563, https://dx.doi.org/10.1007/s10549-015-3568-z
211. Kneubil, M. C.; Lohsiriwat, V.; Curigliano, G.; Brollo, J.; Botteri, E.; Rotmensz, N.; Martella, S.; Mastropasqua, M. G.; Iera, M.; Coelho, M. B.; et al. Risk of locoregional recurrence in patients with false-negative frozen section or close margins of retroareolar specimen in nipple-sparing mastectomy. *Ann. Surg. Oncol.* **2012**, *19*, 4117-4123, https://dx.doi.org/10.1245/s10434-012-2514-0
212. Luo, D.; Ha, J.; Latham, B.; Ingram, D.; Connell, T.; Hastrich, D.; Yeow, W. C.; Willsher, P.; Luo, J. The accuracy of intraoperative subareolar frozen section in nipple-sparing mastectomies. *Ochsner J.* **2010**, *10*, 188-192,
213. Chan, S. E.; Liao, C. Y.; Wang, T. Y.; Chen, S. T.; Chen, D. R.; Lin, Y. J.; Chen, C. J.; Wu, H. K.; Chen, S. L.; Kuo, S. J.; et al. The diagnostic utility of preoperative breast magnetic resonance imaging (MRI) and/or intraoperative sub-nipple biopsy in nipple-sparing mastectomy. *Eur. J. Surg. Oncol.* **2017**, *43*, 76-84, https://dx.doi.org/10.1016/j.ejso.2016.08.005
214. Coopey, S.; Keleher, A.; Daniele, K.; Peled, A. W.; Gomez, J.; Julian, T.; Moreira, A. A. Careful where you cut: Strategies for successful nerve-preserving mastectomy. *Plast. Reconstr. Surg. Glob. Open.* **2024**, *12*, e5817, https://dx.doi.org/10.1097/GOX.0000000000005817
215. Franceschini, G.; Scardina, L.; Di Leone, A.; Terribile, D. A.; Sanchez, A. M.; Magno, S.; D'Archi, S.; Franco, A.; Mason, E. J.; Carnassale, B.; et al. Immediate prosthetic breast reconstruction after nipple-sparing mastectomy: Traditional subpectoral technique versus direct-to-implant prepectoral reconstruction without acellular dermal matrix. *J. Pers. Med.* **2021**, *11*, 22, https://dx.doi.org/10.3390/jpm11020153

216. Scardina, L.; Di Leone, A.; Biondi, E.; Carnassale, B.; Sanchez, A. M.; D'Archi, S.; Franco, A.; Moschella, F.; Magno, S.; Terribile, D.; et al. Prepectoral vs. submuscular immediate breast reconstruction in patients undergoing mastectomy after neoadjuvant chemotherapy: Our early experience. *J. Pers. Med.* **2022**, *12*, 19, https://dx.doi.org/10.3390/jpm12091533
217. Darrach, H.; Kraenzlin, F. S.; Khavanin, N.; He, W.; Lee, E.; Sacks, J. M. Pectoral placement of tissue expanders affects inpatient opioid use. *Breast J.* **2021**, *27*, 126-133, https://dx.doi.org/10.1111/tbj.14149
218. Kraenzlin, F.; Darrach, H.; Khavanin, N.; Kokosis, G.; Aliu, O.; Broderick, K.; Rosson, G. D.; Manahan, M. A.; Sacks, J. M. Tissue expander-based breast reconstruction in the prepectoral versus subpectoral plane: An analysis of short-term outcomes. *Ann. Plast. Surg.* **2021**, *86*, 19-23, https://dx.doi.org/10.1097/SAP.0000000000002415
219. Sinnott, C. J.; Persing, S. M.; Pronovost, M.; Hodyl, C.; McConnell, D.; Ott Young, A. Impact of postmastectomy radiation therapy in prepectoral versus subpectoral implant-based breast reconstruction. *Ann. Surg. Oncol.* **2018**, *25*, 2899-2908, https://dx.doi.org/10.1245/s10434-018-6602-7
220. Copeland-Halperin, L. R.; Yemc, L.; Emery, E.; Collins, D.; Liu, C.; Mesbahi, A. N.; Venturi, M. L. Evaluating postoperative narcotic use in prepectoral versus dual-plane breast reconstruction following mastectomy. *Plast. Reconstr. Surg. Glob. Open.* **2019**, *7*, e2082, https://dx.doi.org/10.1097/GOX.0000000000002082
221. Avila, A.; Bartholomew, A. J.; Sosin, M.; Deldar, R.; Griffith, K. F.; Willey, S. C.; Song, D. H.; Fan, K. L.; Tousimis, E. A. Acute postoperative complications in prepectoral versus subpectoral reconstruction following nipple-sparing mastectomy. *Plast. Reconstr. Surg.* **2020**, *146*, 715e-720e, https://dx.doi.org/10.1097/PRS.0000000000007326
222. Banuelos, J.; Abu-Ghname, A.; Vyas, K.; Sharaf, B.; Nguyen, M. T.; Harless, C.; Manrique, O. J.; Martinez-Jorge, J.; Tran, N. V. Should obesity be considered a contraindication for prepectoral breast reconstruction? *Plast. Reconstr. Surg.* **2020**, *145*, 619-627, https://dx.doi.org/10.1097/PRS.0000000000006540
223. Kim, J. H.; Hong, S. E. A comparative analysis between subpectoral versus prepectoral single stage direct-to-implant breast reconstruction. *Medicina.* **2020**, *56*, 13, https://dx.doi.org/10.3390/medicina56100537
224. Nealon, K. P.; Weitzman, R. E.; Sobti, N.; Gadd, M.; Specht, M.; Jimenez, R. B.; Ehrlichman, R.; Faulkner, H. R.; Austen, W. G., Jr.; Liao, E. C. Prepectoral direct-to-implant breast reconstruction: Safety outcome endpoints and delineation of risk factors. *Plast. Reconstr. Surg.* **2020**, *145*, 898e-908e, https://dx.doi.org/10.1097/PRS.0000000000006721
225. Belmonte, B. M.; Campbell, C. A. Safety profile and predictors of aesthetic outcomes after prepectoral breast reconstruction with meshed acellular dermal matrix. *Ann. Plast. Surg.* **2021**, *86*, S585-S592, https://dx.doi.org/10.1097/SAP.0000000000002764
226. Bozzuto, L. M.; Bartholomew, A. J.; Tung, S.; Sosin, M.; Tambar, S.; Cox, S.; Perez-Alvarez, I. M.; King, C. A.; Chan, M. C.; Pittman, T. A.; et al. Decreased postoperative pain and opioid use following prepectoral versus subpectoral breast reconstruction after mastectomy: A retrospective cohort study: Pain after pre- versus subpectoral reconstruction. *J. Plast. Reconstr. Aesthet. Surg.* **2021**, *74*, 1763-1769, https://dx.doi.org/10.1016/j.bjps.2020.12.009
227. Haddock, N. T.; Kadakia, Y.; Liu, Y.; Teotia, S. S. Prepectoral versus subpectoral tissue expander breast reconstruction: A historically controlled, propensity score-matched comparison of perioperative outcomes. *Plast. Reconstr. Surg.* **2021**, *148*, 1-9, https://dx.doi.org/10.1097/PRS.0000000000008013

228. Plachinski, S. J.; Boehm, L. M.; Adamson, K. A.; LoGiudice, J. A.; Doren, E. L. Comparative analysis of prepectoral versus subpectoral implant-based breast reconstruction. *Plast. Reconstr. Surg. Glob. Open*. **2021**, *9*, e3709, https://dx.doi.org/10.1097/GOX.0000000000003709
229. Ribuffo, D.; Berna, G.; De Vita, R.; Di Benedetto, G.; Cigna, E.; Greco, M.; Valdatta, L.; Onesti, M. G.; Lo Torto, F.; Marcasciano, M.; et al. Dual-plane retro-pectoral versus pre-pectoral DTI breast reconstruction: An Italian multicenter experience. *Aesthetic Plast. Surg*. **2021**, *45*, 51-60, https://dx.doi.org/10.1007/s00266-020-01892-y
230. Walker, N. J.; Park, J. G.; Maus, J. C.; Motamedi, V.; Rebowe, R. E.; Runyan, C. M.; Tucker, S. L. Prepectoral versus subpectoral breast reconstruction in high-body mass index patients. *Ann. Plast. Surg*. **2021**, *87*, 136-143, https://dx.doi.org/10.1097/SAP.0000000000002682
231. Holland, M.; Su, P.; Piper, M.; Withers, J.; Harbell, M. W.; Bokoch, M. P.; Sbitany, H. Prepectoral breast reconstruction reduces opioid consumption and pain after mastectomy: A head-to-head comparison with submuscular reconstruction. *Ann. Plast. Surg*. **2022**, *89*, 492-499, https://dx.doi.org/10.1097/SAP.0000000000003271
232. Houvenaeghel, G.; Bannier, M.; Bouteille, C.; Tallet, C.; Sabiani, L.; Charavil, A.; Bertrand, A.; Van Troy, A.; Buttarelli, M.; Teyssandier, C.; et al. Postoperative outcomes of pre-pectoral versus sub-pectoral implant immediate breast reconstruction. *Cancers (Basel)*. **2024**, *16*, 12, https://dx.doi.org/10.3390/cancers16061129
233. Asaad, M.; Hassan, A. M.; Morris, N.; Kumar, S.; Liu, J.; Butler, C. E.; Selber, J. C. Impact of obesity on outcomes of prepectoral vs subpectoral implant-based breast reconstruction. *Aesthet. Surg. J*. **2023a**, *43*, NP774-NP786, https://dx.doi.org/10.1093/asj/sjad175
234. Asaad, M.; Yu, J. Z.; Tran, J. P.; Liu, J.; O'Grady, B.; Clemens, M. W.; Largo, R. D.; Mericli, A. F.; Schaverien, M.; Shuck, J.; et al. Surgical and patient-reported outcomes of 694 two-stage prepectoral versus subpectoral breast reconstructions. *Plast. Reconstr. Surg*. **2023b**, *152*, 43S-54S, https://dx.doi.org/10.1097/PRS.0000000000010380
235. Hassan, A. M.; Asaad, M.; Morris, N.; Kumar, S.; Liu, J.; Mitchell, M. P.; Shuck, J. W.; Clemens, M. W.; Butler, C. E.; Selber, J. C. Subpectoral implant placement is not protective against postmastectomy radiotherapy-related complications compared to prepectoral placement. *Plast. Reconstr. Surg*. **2024**, *153*, 24-33, https://dx.doi.org/10.1097/PRS.0000000000010489
236. ElSherif, A.; Bernard, S.; Djohan, R.; Atallah, A.; Tu, C.; Valente, S. A. Nipple necrosis rate with submuscular versus prepectoral implant-based reconstruction in nipple sparing mastectomy: Does it differ? *Am. J. Surg*. **2024**, *230*, 57-62, https://dx.doi.org/10.1016/j.amjsurg.2023.11.039
237. Min, K.; Min, J. C.; Han, H. H.; Kim, E. K.; Eom, J. S. Comparing outcomes of prepectoral, partial muscle-splitting subpectoral, and dual-plane subpectoral direct-to-implant reconstruction: Implant upward migration and the pectoralis muscle. *Gland Surg*. **2024**, *13*, 852-863, https://dx.doi.org/10.21037/gs-24-45
238. Gabriel, A.; Sigalove, S.; Sigalove, N. M.; Storm-Dickerson, T. L.; Rice, J.; Pope, N.; Maxwell, G. P. Prepectoral revision breast reconstruction for treatment of implant-associated animation deformity: A review of 102 reconstructions. *Aesthet. Surg. J*. **2018**, *38*, 519-526, https://dx.doi.org/10.1093/asj/sjx261
239. Sigalove, S.; Maxwell, G. P.; Gabriel, A. Outcomes utilizing Inspira implants in revisionary reconstructive surgery. *Plast. Reconstr. Surg*. **2019**, *144*, 66S-72S, https://dx.doi.org/10.1097/PRS.0000000000005952

240. Jones, G. E.; King, V. A.; Yoo, A. Prepectoral site conversion for animation deformity. *Plast. Reconstr. Surg. Glob. Open*. **2019**, *7*, e2301, https://dx.doi.org/10.1097/GOX.0000000000002301
241. Holland, M. C.; Lentz, R.; Sbitany, H. Surgical correction of breast animation deformity with implant pocket conversion to a prepectoral plane. *Plast. Reconstr. Surg.* **2020**, *145*, 632-642, https://dx.doi.org/10.1097/PRS.0000000000006590
242. Salgarello, M.; Barone Adesi, L.; Macri, G.; Visconti, G. When to consider prepectoral implant conversion after subpectoral implant breast reconstruction and how to plan it. *Aesthet. Surg. J.* **2023**, *43*, NP1071-NP1077, https://dx.doi.org/10.1093/asj/sjad290
243. Talwar, A. A.; Lanni, M. A.; Ryan, I. A.; Kodali, P.; Bernstein, E.; McAuliffe, P. B.; Broach, R. B.; Serletti, J. M.; Butler, P. D.; Fosnot, J. Prepectoral versus submuscular implant-based breast reconstruction: A matched-pair comparison of outcomes. *Plast. Reconstr. Surg.* **2024**, *153*, 281e-290e, https://dx.doi.org/10.1097/PRS.0000000000010618
244. Chen, Y.; Wang, M. L.; Black, G. G.; Bernstein, J. L.; Chinta, M.; Otterburn, D. M. Timeline and incidence of postoperative complications in prepectoral, dual-plane, and total submuscular alloplastic reconstruction with and without biosynthetic scaffold usage. *Ann. Plast. Surg.* **2023b**, *90*, S466-S471, https://dx.doi.org/10.1097/SAP.0000000000003482
245. Houvenaeghel, G.; Cohen, M.; Sabiani, L.; Van Troy, A.; Quilichini, O.; Charavil, A.; Buttarelli, M.; Rua, S.; Tallet, A.; de Nonneville, A.; et al. Mastectomy and immediate breast reconstruction with pre-pectoral or sub-pectoral implant: Assessing clinical practice, post-surgical outcomes, patient's satisfaction and cost. *J. Surg. Res. (Houst.).* **2022**, *5*, 500-510, https://dx.doi.org/10.26502/jsr.10020250
246. Parikh, R. P.; Brown, G. M.; Sharma, K.; Yan, Y.; Myckatyn, T. M. Immediate implant-based breast reconstruction with acellular dermal matrix: A comparison of sterile and aseptic alloderm in 2039 consecutive cases. *Plast. Reconstr. Surg.* **2018**, *142*, 1401-1409, https://dx.doi.org/10.1097/PRS.0000000000004968
247. Dieterich, M.; Faridi, A. Biological matrices and synthetic meshes used in implant-based breast reconstruction - a review of products available in Germany. *Geburtshilfe Frauenheilkd.* **2013**, *73*, 1100-1106,
248. Zenn, M.; Venturi, M.; Pittman, T.; Spear, S.; Gurtner, G.; Robb, G.; Mesbahi, A.; Dayan, J. Optimizing outcomes of postmastectomy breast reconstruction with acellular dermal matrix: A review of recent clinical data. *Eplasty [Electronic Resource].* **2017**, *17*, e18,
249. Allergan Aesthetics: An AbbVie Company. AlloDermTM regenerative tissue matrix. Product portfolio brochure. Allergan Aesthetics. 2022 Dec 22, cited 2024 Jun 10. Available from: https://hcp.alloderm.com/resources#DownloadMaterials; https://hcp.alloderm.com/-/media/project/alloderm2022/downloadpdf/AlloDermPortfolioBrochure.pdf.
250. Allergan Aesthetics: An AbbVie Company. AlloDerm Select™. AlloDerm Select Restore™ regenerative tissue matrix. Instructions for use. Markham, ON: Allergan Aesthetics. 2024 Feb 29. Modified 2024 Apr 3, cited 2024 Jun 10. Available from: https://www.allerganaesthetics.ca/en/our-products/medical-devices.
251. Powers, J. M.; Reuter Munoz, K. D.; Parkerson, J.; Nigro, L. C.; Blanchet, N. P. From salvage to prevention: A single-surgeon experience with acellular dermal matrix and infection in prepectoral breast reconstruction. *Plast. Reconstr. Surg.* **2021**, *148*, 1201-1208, https://dx.doi.org/10.1097/PRS.0000000000008519
252. Wood, K. L.; Margulies, I. G.; Shay, P. L.; Ashikari, A. Y.; Salzberg, C. A. Complications after perforated versus nonperforated acellular dermal matrix use in direct-to-implant

breast reconstruction: A propensity score analysis. *Plast. Reconstr. Surg. Glob. Open.* **2020**, *8*, e2690, https://dx.doi.org/10.1097/GOX.0000000000002690
253. Luo, J.; Willis, R. N., Jr.; Ohlsen, S. M.; Piccinin, M.; Moores, N.; Kwok, A. C.; Agarwal, J. P. Meshed acellular dermal matrix for two-staged prepectoral breast reconstruction: An institutional experience. *Arch. Plast. Surg.* **2022**, *49*, 166-173, https://dx.doi.org/10.1055/s-0042-1744408
254. Zammit, D.; Kanevsky, J.; Meng, F.-Y.; Dionisopoulos, T. Meshed acellular dermal matrix: Technique and application in implant based breast reconstruction. *Plast. Aesthet. Res.* **2016**, *3*, 254-256, 10.20517/2347-9264.2015.128
255. Sweitzer, K.; Carruthers, K. H.; Blume, L.; Tiwari, Pankaj; Kocak, Ergun. The biomechanical properties of meshed versus perforated acellular dermal matrices (ADMs). *Plastic and Reconstructive Surgery – Global Open.* **2021**, *9*, e3454, 10.1097/gox.0000000000003454
256. Scheflan, M.; Allweis, T. M.; Ben Yehuda, D.; Maisel Lotan, A. Meshed acellular dermal matrix in immediate prepectoral implant-based breast reconstruction. *Plast. Reconstr. Surg. Glob. Open.* **2020**, *8*, e3265, https://dx.doi.org/10.1097/GOX.0000000000003265
257. Gui, G.; Gui, M.; Gui, A.; Tasoulis, M. K. Physical characteristics of surgimend meshed biological adm in immediate prepectoral implant breast reconstruction. *Plast. Reconstr. Surg. Glob. Open.* **2022**, *10*, e4369, https://dx.doi.org/10.1097/GOX.0000000000004369
258. Hill, E. J. R.; Buck, D. W., 2nd. The "butterfly" wrap: A simplified technique for consistent prosthesis coverage in prepectoral breast reconstruction. *Plast. Reconstr. Surg. Glob. Open.* **2018**, *6*, e2007, https://dx.doi.org/10.1097/GOX.0000000000002007
259. Liliav, B.; Patel, P.; Jacobson, A. K. Prepectoral breast reconstruction: A technical algorithm. *Plast. Reconstr. Surg. Glob. Open.* **2019**, *7*, e2107, https://dx.doi.org/10.1097/GOX.0000000000002107
260. Sigalove, S. Options in acellular dermal matrix-device assembly. *Plast. Reconstr. Surg.* **2017**, *140*, 39S-42S, https://dx.doi.org/10.1097/PRS.0000000000004049
261. Lee, Y. J.; Kanchwala, S. K.; Cho, H.; Jolly, J. C.; Jablonka, E.; Tanis, M.; Kamien, R. D.; Yang, S. Natural shaping of acellular dermal matrices for implant-based breast reconstruction via expansile kirigami. *Adv. Mater.* **2023**, *35*, e2208088, https://dx.doi.org/10.1002/adma.202208088
262. Jordan, S. W.; Khavanin, N.; Fine, N. A.; Kim, J. Y. S. An algorithmic approach for selective acellular dermal matrix use in immediate two-stage breast reconstruction: Indications and outcomes. *Plast. Reconstr. Surg.* **2014**, *134*, 178-188, https://dx.doi.org/10.1097/PRS.0000000000000366
263. Lee, K. T.; Lee, H.; Jeon, B. J.; Mun, G. H.; Bang, S. I.; Pyon, J. K. Impact of overweight/obesity on the development of hematoma following tissue expander-based breast reconstruction. *J. Plast. Reconstr. Aesthet. Surg.* **2020**, *19*, 19, https://dx.doi.org/10.1016/j.bjps.2020.08.105
264. Lee, K. T.; Eom, Y.; Mun, G. H.; Bang, S. I.; Jeon, B. J.; Pyon, J. K. Efficacy of partial-versus full-sling acellular dermal matrix use in implant-based breast reconstruction: A head-to-head comparison. *Aesthetic Plast. Surg.* **2018**, *42*, 422-433, https://dx.doi.org/10.1007/s00266-018-1084-3
265. Lee, K. T.; Hong, S. H.; Jeon, B. J.; Pyon, J. K.; Mun, G. H.; Bang, S. I. Predictors for prolonged drainage following tissue expander-based breast reconstruction. *Plast. Reconstr. Surg.* **2019**, *144*, 9e-17e, https://dx.doi.org/10.1097/PRS.0000000000005697
266. Pires, G.; Marquez, J. L.; Memmott, S.; Sudduth, J. D.; Moss, W.; Eddington, D.; Hobson, G.; Tuncer, F.; Agarwal, J. P.; Kwok, A. C. Early complications after prepectoral tissue expander placement in breast reconstruction with and without acellular dermal matrix.

Plast. Reconstr. Surg. **2024**, *153*, 1221-1229, https://dx.doi.org/10.1097/PRS.0000000000010801
267. Pannucci, C. J.; Antony, A. K.; Wilkins, E. G. The impact of acellular dermal matrix on tissue expander/implant loss in breast reconstruction: An analysis of the tracking outcomes and operations in plastic surgery database. *Plast. Reconstr. Surg.* **2013**, *132*, 1-10, https://dx.doi.org/10.1097/PRS.0b013e318290f917
268. Kilmer, L. H.; Challa, S.; Stranix, J. T.; Campbell, C. A. Case-matched comparison of implant-based breast reconstruction with and without acellular dermal matrix. *Plast. Reconstr. Surg. Glob. Open.* **2024**, *12*, e5660, https://dx.doi.org/10.1097/GOX.0000000000005660
269. Plotsker, E. L.; Graziano, F. D.; Rubenstein, R. N.; Haglich, K.; Allen, R. J., Jr.; Coriddi, M. R.; Dayan, J. H.; Poulton, R.; McKernan, C.; Mehrara, B. J.; et al. Early complications in prepectoral breast reconstructions with and without acellular dermal matrix: A preliminary analysis of outcomes. *Plast. Reconstr. Surg.* **2024**, *153*, 786-793, 10.1097/prs.0000000000010712
270. Davila, A. A.; Seth, A. K.; Wang, E.; Hanwright, P.; Bilimoria, K.; Fine, N.; Kim, J. Y. Human acellular dermis versus submuscular tissue expander breast reconstruction: A multivariate analysis of short-term complications. *Arch. Plast. Surg.* **2013**, *40*, 19-27, https://dx.doi.org/10.5999/aps.2013.40.1.19
271. Winocour, S.; Martinez-Jorge, J.; Habermann, E.; Thomsen, K.; Lemaine, V. Early surgical site infection following tissue expander breast reconstruction with or without acellular dermal matrix: National benchmarking using National Surgical Quality Improvement Program. *Arch. Plast. Surg.* **2015**, *42*, 194-200, https://dx.doi.org/10.5999/aps.2015.42.2.194
272. Luo, J.; Moss, W. D.; Pires, G. R.; Rhemtulla, I. A.; Rosales, M.; Stoddard, G. J.; Agarwal, J. P.; Kwok, A. C. A nationwide analysis evaluating the safety of using acellular dermal matrix with tissue expander-based breast reconstruction. *Arch. Plast. Surg.* **2022**, *49*, 716-723, https://dx.doi.org/10.1055/s-0042-1758638
273. Graziano, F. D.; Plotsker, E. L.; Rubenstein, R. N.; Haglich, K.; Stern, C. S.; Matros, E.; Nelson, J. A. National trends in acellular dermal matrix utilization in immediate breast reconstruction. *Plast. Reconstr. Surg.* **2024**, *153*, 25e-36e, https://dx.doi.org/10.1097/PRS.0000000000010575
274. Nahabedian, M. Y. AlloDerm performance in the setting of prosthetic breast surgery, infection, and irradiation. *Plast. Reconstr. Surg.* **2009**, *124*, 1743-1753, https://dx.doi.org/10.1097/PRS.0b013e3181bf8087
275. Sbitany, H.; Sandeen, S. N.; Amalfi, A. N.; Davenport, M. S.; Langstein, H. N. Acellular dermis-assisted prosthetic breast reconstruction versus complete submuscular coverage: A head-to-head comparison of outcomes. *Plast. Reconstr. Surg.* **2009**, *124*, 1735-1740, https://dx.doi.org/10.1097/PRS.0b013e3181bf803d
276. Chun, Y. S.; Verma, K.; Rosen, H.; Lipsitz, S.; Morris, D.; Kenney, P.; Eriksson, E. Implant-based breast reconstruction using acellular dermal matrix and the risk of postoperative complications. *Plast. Reconstr. Surg.* **2010**, *125*, 429-436, https://dx.doi.org/10.1097/PRS.0b013e3181c82d90
277. Ganske, I.; Verma, K.; Rosen, H.; Eriksson, E.; Chun, Y. S. Minimizing complications with the use of acellular dermal matrix for immediate implant-based breast reconstruction. *Ann. Plast. Surg.* **2013**, *71*, 464-470, https://dx.doi.org/10.1097/SAP.0b013e3182a7cc9b
278. Liu, A. S.; Kao, H. K.; Reish, R. G.; Hergrueter, C. A.; May, J. W., Jr.; Guo, L. Postoperative complications in prosthesis-based breast reconstruction using acellular

dermal matrix. *Plast. Reconstr.Surg.* **2011**, *127*, 1755-1762, https://dx.doi.org/10.1097/PRS.0b013e31820cf233
279. Vardanian, A. J.; Clayton, J. L.; Roostaeian, J.; Shirvanian, V.; Da Lio, A.; Lipa, J. E.; Crisera, C.; Festekjian, J. H. Comparison of implant-based immediate breast reconstruction with and without acellular dermal matrix. *Plast. Reconstr. Surg.* **2011**, *128*, 403e-410e, https://dx.doi.org/10.1097/PRS.0b013e31822b6637
280. McCarthy, C. M.; Lee, C. N.; Halvorson, E. G.; Riedel, E.; Pusic, A. L.; Mehrara, B. J.; Disa, J. J. The use of acellular dermal matrices in two-stage expander/implant reconstruction: A multicenter, blinded, randomized controlled trial. *Plast. Reconstr. Surg.* **2012**, *130*, 57S-66S, https://dx.doi.org/10.1097/PRS.0b013e31825f05b4
281. Parks, J. W.; Hammond, S. E.; Walsh, W. A.; Adams, R. L.; Chandler, R. G.; Luce, E. A. Human acellular dermis versus no acellular dermis in tissue expansion breast reconstruction. *Plast. Reconstr. Surg.* **2012**, *130*, 739-746, https://dx.doi.org/10.1097/PRS.0b013e318262f06e
282. Weichman, K. E.; Wilson, S. C.; Weinstein, A. L.; Hazen, A.; Levine, J. P.; Choi, M.; Karp, N. S. The use of acellular dermal matrix in immediate two-stage tissue expander breast reconstruction. *Plast. Reconstr. Surg.* **2012**, *129,* 1049-1058, https://dx.doi.org/10.1097/PRS.0b013e31824a2acb
283. Weichman, K. E.; Wilson, S. C.; Saadeh, P. B.; Hazen, A.; Levine, J. P.; Choi, M.; Karp, N. S. Sterile "ready-to-use" AlloDerm decreases postoperative infectious complications in patients undergoing immediate implant-based breast reconstruction with acellular dermal matrix. *Plast. Reconstr. Surg.* **2013**, *132*, 725-736, https://dx.doi.org/10.1097/PRS.0b013e31829fe35b
284. Arnaout, A.; Zhang, J.; Frank, S.; Momtazi, M.; Cordeiro, E.; Roberts, A.; Ghumman, A.; Fergusson, D.; Stober, C.; Pond, G.; et al. A randomized controlled trial comparing Alloderm-RTU with DermACELL in immediate subpectoral implant-based breast reconstruction. *Curr. Oncol.* **2021**, *28,* 184-195, https://dx.doi.org/10.3390/curroncol28010020
285. Stein, M. J.; Arnaout, A.; Lichtenstein, J. B.; Frank, S. G.; Cordeiro, E.; Roberts, A.; Ghaedi, B.; Zhang, J. A comparison of patient-reported outcomes between Alloderm and Dermacell in immediate alloplastic breast reconstruction: A randomized control trial. *J. Plast. Reconstr. Aesthet. Surg.* **2021**, *74,* 41-47, https://dx.doi.org/10.1016/j.bjps.2020.08.018
286. Chu, J. J.; Nelson, J. A.; Kokosis, G.; Haglich, K.; McKernan, C. D.; Rubenstein, R.; Vingan, P. S.; Allen, R. J., Jr.; Coriddi, M. R.; Dayan, J. H.; et al. A cohort analysis of early outcomes after Alloderm, FlexHD, and Surgimend use in two-stage prepectoral breast reconstruction. *Aesthet. Surg. J.* **2023**, *43*, 1491-1498, https://dx.doi.org/10.1093/asj/sjad246
287. Berger, L. E.; Spoer, D. L.; Huffman, S. S.; Haffner, Z. K.; Tom, L. K.; Parkih, R. P.; Song, D. H.; Fan, K. L. Acellular dermal matrix-assisted, prosthesis-based breast reconstruction: A comparison of SurgiMend PRS, AlloDerm, and DermACELL. *Ann. Plast. Surg.* **2024**, *93*, 34-42, https://dx.doi.org/10.1097/SAP.0000000000003990
288. Johnson, A. C.; Colakoglu, S.; Siddikoglu, D.; Li, A.; Kaoutzanis, C.; Cohen, J. B.; Chong, T. W.; Mathes, D. W. Impact of dermal matrix brand in implant-based breast reconstruction outcomes. *Plast. Reconstr. Surg.* **2022**, *150,* 17-25, https://dx.doi.org/10.1097/PRS.0000000000009178
289. Zenn, M. R.; Salzberg, C. A. A direct comparison of Alloderm-Ready to Use (RTU) and DermACELL in immediate breast implant reconstruction. *Eplasty [Electronic Resource].* **2016**, *16*, e23,

290. Mendenhall, S. D.; Anderson, L. A.; Ying, J.; Boucher, K. M.; Liu, T.; Neumayer, L. A.; Agarwal, J. P. The BREASTrial: Stage I. Outcomes from the time of tissue expander and acellular dermal matrix placement to definitive reconstruction. *Plast. Reconstr. Surg.* **2015**, *135*, 29e-42e, https://dx.doi.org/10.1097/PRS.0000000000000758
291. Mendenhall, S. D.; Anderson, L. A.; Ying, J.; Boucher, K. M.; Neumayer, L. A.; Agarwal, J. P. The BREASTrial Stage II: ADM breast reconstruction outcomes from definitive reconstruction to 3 months postoperative. *Plast. Reconstr. Surg. Glob. Open.* **2017**, *5*, e1209, https://dx.doi.org/10.1097/GOX.0000000000001209
292. Mendenhall, S. D.; Moss, W. D.; Graham, E. M.; Carter, G.; Agarwal, J. P. The BREASTrial Stage III: Acellular dermal matrix breast reconstruction outcomes from 3 months to 2 years postoperatively. *Plast. Reconstr. Surg.* **2023**, *151*, 17-24, https://dx.doi.org/10.1097/PRS.0000000000009768
293. Palaia, D. A.; Arthur, K. S.; Cahan, A. C.; Rosenberg, M. H. Incidence of seromas and infections using fenestrated versus nonfenestrated acellular dermal matrix in breast reconstructions. *Plast. Reconstr. Surg. Glob. Open.* **2015**, *3*, e569, https://dx.doi.org/10.1097/GOX.0000000000000559
294. Seth, A. K.; Persing, S.; Connor, C. M.; Davila, A.; Hirsch, E.; Fine, N. A.; Kim, J. Y. A comparative analysis of cryopreserved versus prehydrated human acellular dermal matrices in tissue expander breast reconstruction. *Ann. Plast. Surg.* **2013**, *70*, 632-635, https://dx.doi.org/10.1097/SAP.0b013e318250f0b4
295. Liu, D. Z.; Mathes, D. W.; Neligan, P. C.; Said, H. K.; Louie, O. Comparison of outcomes using AlloDerm versus FlexHD for implant-based breast reconstruction. *Ann. Plast. Surg.* **2014**, *72*, 503-507, https://dx.doi.org/10.1097/SAP.0b013e318268a87c
296. Ranganathan, K.; Santosa, K. B.; Lyons, D. A.; Mand, S.; Xin, M.; Kidwell, K.; Brown, D. L.; Wilkins, E. G.; Momoh, A. O. Use of acellular dermal matrix in postmastectomy breast reconstruction: Are all acellular dermal matrices created equal? *Plast. Reconstr. Surg.* **2015**, *136*, 647-653, https://dx.doi.org/10.1097/PRS.0000000000001569
297. Sobti, N.; Liao, E. C. Surgeon-controlled study and meta-analysis comparing FlexHD and AlloDerm in immediate breast reconstruction outcomes. *Plast. Reconstr. Surg.* **2016**, *138*, 959-967, https://dx.doi.org/10.1097/PRS.0000000000002616
298. Broyles, J. M.; Liao, E. C.; Kim, J.; Heistein, J.; Sisco, M.; Karp, N.; Lau, F. H.; Chun, Y. S. Acellular dermal matrix-associated complications in implant-based breast reconstruction: A multicenter, prospective, randomized controlled clinical trial comparing two human tissues. *Plast. Reconstr. Surg.* **2021**, *148*, 493-500, https://dx.doi.org/10.1097/PRS.0000000000008194
299. Keifer, O. P., Jr.; Page, E. K.; Hart, A.; Rudderman, R.; Carlson, G. W.; Losken, A. A complication analysis of 2 acellular dermal matrices in prosthetic-based breast reconstruction. *Plast. Reconstr. Surg. Glob. Open.* **2016**, *4*, e800, https://dx.doi.org/10.1097/GOX.0000000000000790
300. Hadad, I.; Liu, A. S.; Guo, L. A new approach to minimize acellular dermal matrix use in prosthesis-based breast reconstruction. *Plast. Reconstr. Surg. Glob. Open.* **2015**, *3*, e472, https://dx.doi.org/10.1097/GOX.0000000000000433
301. Frey, J. D.; Alperovich, M.; Weichman, K. E.; Wilson, S. C.; Hazen, A.; Saadeh, P. B.; Levine, J. P.; Choi, M.; Karp, N. S. Breast reconstruction using contour fenestrated Alloderm: Does improvement in design translate to improved outcomes? *Plast. Reconstr. Surg. Glob. Open.* **2015**, *3*, e505, https://dx.doi.org/10.1097/GOX.0000000000000482
302. Yuen, J. C.; Yue, C. J.; Erickson, S. W.; Cooper, S.; Boneti, C.; Henry-Tillman, R.; Klimberg, S. Comparison between freeze-dried and ready-to-use Alloderm in alloplastic breast reconstruction. *Plast. Reconstr. Surg. Glob. Open.* **2014**, *2*, e119, https://dx.doi.org/10.1097/GOX.0000000000000061

303. Widmyer, A. S.; Mirhaidari, S. J.; Wagner, D. S. Implant-based breast reconstruction outcomes comparing freeze-dried aseptic Alloderm and sterile ready-to-use Alloderm. *Plast. Reconstr. Surg. Glob. Open.* **2019**, *7*, e2530, https://dx.doi.org/10.1097/GOX.0000000000002530
304. Hanson, S. E.; Meaike, J. D.; Selber, J. C.; Liu, J.; Li, L.; Hassid, V. J.; Baumann, D. P.; Butler, C. E.; Garvey, P. B. Aseptic freeze-dried versus sterile wet-packaged human cadaveric acellular dermal matrix in immediate tissue expander breast reconstruction: A propensity score analysis. *Plast. Reconstr. Surg.* **2018**, *141*, 624e-632e, https://dx.doi.org/10.1097/PRS.0000000000004323
305. Han, W. Y.; Han, S. J.; Eom, J. S.; Kim, E. K.; Han, H. H. A comparative study of wraparound versus anterior coverage placement of acellular dermal matrix in prepectoral breast reconstruction. *Plast. Reconstr. Surg.* **2023a**, *152*, 716-724, https://dx.doi.org/10.1097/PRS.0000000000010347
306. Han, W. Y.; Han, S. J.; Kim, E. K.; Han, H. H.; Eom, J. S. A comparison of clinical outcomes of acellular dermal matrix with and without radiation sterilization process in immediate prepectoral direct-to-implant breast reconstruction. *J. Plast. Reconstr. Aesthet. Surg.* **2023b**, *87*, 461-466, https://dx.doi.org/10.1016/j.bjps.2023.10.087
307. Sigalove, S.; O'Rorke, E.; Maxwell, G. P.; Gabriel, A. Evaluation of the safety of a GalaFLEX-AlloDerm construct in prepectoral breast reconstruction. *Plast. Reconstr. Surg.* **2022**, *150*, 75S-81S, https://dx.doi.org/10.1097/PRS.0000000000009520
308. Levy, A. S.; Bernstein, J. L.; Xia, J. J.; Otterburn, D. M. Poly-4-hydroxybutyric acid mesh compares favorably with acellular dermal matrix in tissue expander-based breast reconstruction. *Ann. Plast. Surg.* **2020**, *85*, S2-S7, https://dx.doi.org/10.1097/SAP.0000000000002339
309. Wederfoort, J. L. M.; van Santbrink, E.; Hommes, J. E.; Heuts, E. M.; Van Kuijk, S. M. J.; van der Hulst, R.; Piatkowski, A. Donor site satisfaction following autologous fat transfer for total breast reconstruction. *Aesthet. Surg. J.* **2023a**, *43*, 40-48, https://dx.doi.org/10.1093/asj/sjac125
310. Petit, J. Y.; Botteri, E.; Lohsiriwat, V.; Rietjens, M.; De Lorenzi, F.; Garusi, C.; Rossetto, F.; Martella, S.; Manconi, A.; Bertolini, F.; et al. Locoregional recurrence risk after lipofilling in breast cancer patients. *Ann. Oncol.* **2012**, *23*, 582-588, https://dx.doi.org/10.1093/annonc/mdr158
311. Gale, K. L.; Rakha, E. A.; Ball, G.; Tan, V. K.; McCulley, S. J.; Macmillan, R. D. A case-controlled study of the oncologic safety of fat grafting. *Plast. Reconstr. Surg.* **2015**, *135*, 1263-1275, https://dx.doi.org/10.1097/PRS.0000000000001151
312. Seth, A. K.; Hirsch, E. M.; Kim, J. Y. S.; Fine, N. A. Long-term outcomes following fat grafting in prosthetic breast reconstruction: A comparative analysis. *Plast. Reconstr. Surg.* **2012**, *130*, 984-990, https://dx.doi.org/10.1097/PRS.0b013e318267d34d
313. Kim, H. Y.; Jung, B. K.; Lew, D. H.; Lee, D. W. Autologous fat graft in the reconstructed breast: Fat absorption rate and safety based on sonographic identification. *Arch. Plast. Surg.* **2014**, *41*, 740-747, https://dx.doi.org/10.5999/aps.2014.41.6.740
314. Masia, J.; Bordoni, D.; Pons, G.; Liuzza, C.; Castagnetti, F.; Falco, G. Oncological safety of breast cancer patients undergoing free-flap reconstruction and lipofilling. *Eur. J. Surg. Oncol.* **2015**, *41*, 612-616, https://dx.doi.org/10.1016/j.ejso.2015.02.008
315. Fertsch, S.; Hagouan, M.; Munder, B.; Schulz, T.; Abu-Ghazaleh, A.; Schaberick, J.; Stambera, P.; Aldeeri, M.; Andree, C.; Thamm, O. C. Increased risk of recurrence associated with certain risk factors in breast cancer patients after DIEP-flap reconstruction and lipofilling-a matched cohort study with 200 patients. *Gland Surg.* **2017**, *6*, 315-323, https://dx.doi.org/10.21037/gs.2017.03.11

316. Myckatyn, T. M.; Wagner, I. J.; Mehrara, B. J.; Crosby, M. A.; Park, J. E.; Qaqish, B. F.; Moore, D. T.; Busch, E. L.; Silva, A. K.; Kaur, S.; et al. Cancer risk after fat transfer: A multicenter case-cohort study. *Plast. Reconstr. Surg.* **2017**, *139(1)*, 11-18, https://dx.doi.org/10.1097/PRS.0000000000002838
317. Silva-Vergara, C.; Fontdevila, J.; Weshahy, O.; Yuste, M.; Descarrega, J.; Grande, L. Breast cancer recurrence is not increased with lipofilling reconstruction: A case-controlled study. *Ann. Plast. Surg.* **2017**, *79*, 243-248, https://dx.doi.org/10.1097/SAP.0000000000001106
318. Silva-Vergara, C.; Fontdevila, J.; Descarrega, J.; Burdio, F.; Yoon, T. S.; Grande, L. Oncological outcomes of lipofilling breast reconstruction: 195 consecutive cases and literature review. *J. Plast. Reconstr. Aesthet. Surg.* **2016**, *69*, 475-481, https://dx.doi.org/10.1016/j.bjps.2015.12.029
319. Calabrese, C.; Kothari, A.; Badylak, S.; Di Taranto, G.; Marcasciano, M.; Sordi, S.; Barellini, L.; Lo Torto, F.; Tarallo, M.; Gaggelli, I.; et al. Oncological safety of stromal vascular fraction enriched fat grafting in two-stage breast reconstruction after nipple sparing mastectomy: Long-term results of a prospective study. *Eur. Rev. Med. Pharmacol. Sci.* **2018**, *22*, 4768-4777, https://dx.doi.org/10.26355/eurrev_201808_15610
320. Perez-Cano, R.; Vranckx, J. J.; Lasso, J. M.; Calabrese, C.; Merck, B.; Milstein, A. M.; Sassoon, E.; Delay, E.; Weiler-Mithoff, E. M. Prospective trial of adipose-derived regenerative cell (ADRC)-enriched fat grafting for partial mastectomy defects: The RESTORE-2 trial. *Eur. J. Surg. Oncol.* **2012**, *38*, 382-389, https://dx.doi.org/10.1016/j.ejso.2012.02.178
321. Calabrese, S.; Zingaretti, N.; De Francesco, F.; Riccio, M.; De Biasio, F.; Massarut, S.; Almesberger, D.; Parodi, P. C. Long-term impact of lipofilling in hybrid breast reconstruction: Retrospective analysis of two cohorts. *Eur. J. Plast. Surg.* **2020**, *43(3)*, 257-268, https://dx.doi.org/10.1007/s00238-019-01577-z
322. Krastev, T.; van Turnhout, A.; Vriens, E.; Smits, L.; van der Hulst, R. Long-term follow-up of autologous fat transfer vs conventional breast reconstruction and association with cancer relapse in patients with breast cancer. *JAMA Surg.* **2019**, *154*, 56-63, https://dx.doi.org/10.1001/jamasurg.2018.3744
323. Cason, R. W.; Shammas, R. L.; Broadwater, G.; Glener, A. D.; Sergesketter, A. R.; Vernon, R.; Le, E.; Wickenheisser, V. A.; Marks, C. E.; Orr, J.; et al. The influence of fat grafting on breast imaging after postmastectomy reconstruction: A matched cohort analysis. *Plast. Reconstr. Surg.* **2020**, *146*, 1227-1236, https://dx.doi.org/10.1097/PRS.0000000000007327
324. Vyas, K. S.; DeCoster, R. C.; Burns, J. C.; Rodgers, L. T.; Shrout, M. A.; Mercer, J. P.; Coquillard, C.; Dugan, A. J.; Baratta, M. D.; Rinker, B. D.; et al. Autologous fat grafting does not increase risk of oncologic recurrence in the reconstructed breast. *Ann. Plast. Surg.* **2020**, *84*, S405-S410, https://dx.doi.org/10.1097/SAP.0000000000002285
325. Casarrubios, J. M.; Frances, M.; Fuertes, V.; Singer, M.; Navarro, C.; Garcia-Duque, O.; Fernandez-Palacios, J. Oncological outcomes of lipofilling in breast reconstruction: A matched cohort study with 250 patients. *Gland Surg.* **2021**, *10*, 914-923, https://dx.doi.org/10.21037/gs-20-775
326. Klinger, M.; Losurdo, A.; Lisa, A. V. E.; Morenghi, E.; Vinci, V.; Corsi, F.; Albasini, S.; Leonardi, M. C.; Jereczek-Fossa, B. A.; Veronesi, P.; et al. Safety of autologous fat grafting in breast cancer: A multicenter Italian study among 17 senonetwork breast units autologous fat grafting safety: A multicenter Italian retrospective study. *Breast Cancer Res. Treat.* **2022**, *191*, 355-363, https://dx.doi.org/10.1007/s10549-021-06444-9

327. Palve, J.; Luukkaala, T.; Kaariainen, M. Comparison of different techniques in latissimus dorsi breast reconstruction: Plain, immediately lipofilled, and implant enhanced. *Ann. Plast. Surg.* **2022**, *88*, 20-24, https://dx.doi.org/10.1097/SAP.0000000000002798
328. Sorotos, M.; Paolini, G.; D'Orsi, G.; Firmani, G.; Timmermans, F. W.; Santanelli di Pompeo, F. Oncologic outcome of 1000 postmastectomy breast reconstructions with fat transfer: A single-center, matched case-control study. *Plast. Reconstr. Surg.* **2022**, *150*, 4S-12S, https://dx.doi.org/10.1097/PRS.0000000000009494
329. Lee, K. T.; Kim, J. H.; Jeon, B. J.; Pyon, J. K.; Mun, G. H.; Lee, S. K.; Yu, J.; Kim, S. W.; Lee, J. E.; Ryu, J. M.; et al. Association of fat graft with breast cancer recurrence in implant-based reconstruction: Does the timing matter? *Ann. Surg. Oncol.* **2023**, *30*, 1087-1097, https://dx.doi.org/10.1245/s10434-022-12389-0
330. Strong, A. L.; Syrjamaki, J. D.; Kamdar, N.; Wilkins, E. G.; Sears, E. D. Oncological safety of autologous fat grafting for breast reconstruction. *Ann. Plast. Surg.* **2024**, *92*, 21-27, https://dx.doi.org/10.1097/SAP.0000000000003772
331. Escandon, J. M.; Langstein, H. N.; Christiano, J. G.; Aristizabal, A.; Gooch, J. C.; Weiss, A.; Manrique, O. J. Latissimus dorsi flap with immediate fat transfer (LIFT) for autologous breast reconstruction: Single institution experience. *Am. J. Surg.* **2024**, *228*, 185-191, https://dx.doi.org/10.1016/j.amjsurg.2023.09.023
332. Navarro, A. S.; Omalek, D.; Chaltiel, L.; Vaysse, C.; Meresse, T.; Gangloff, D.; Jouve, E.; Selmes, G. Oncologic safety of autologous fat grafting in primary breast reconstruction after mastectomy for cancer. *Eur. J. Surg. Oncol.* **2024**, *50*, 107998, https://dx.doi.org/10.1016/j.ejso.2024.107998
333. National Institute for Health and Care Excellence (NICE). Early and locally advanced breast cancer: diagnosis and management. NICE guideline [NG101] Published: 18 July 2018 Last updated: 16 January 2024.
334. Berrino, P.; Berrino, V. Postmastectomy total breast reconstruction by serial lipografting. In: Di Giuseppe A, Bassetto F, Nahia F (ed), Fat transfer in plastic surgery: Techniques, technology and safety, Chapter 25. Switzerland: Springer NatureSwitzerland AG, pp 355-363. https://doi.org/10.1007/978-3-031-10881-5_25.
335. Homsy, P.; Hockerstedt, A.; Hukkinen, K.; Kauhanen, S. Total breast reconstruction with lipofilling after traditional mastectomy without the use of tissue expanders. *Plast. Reconstr. Surg.* **2023**, *152*, 483-491, 10.1097/PRS.0000000000010252
336. Piffer, A.; Aubry, G.; Cannistra, C.; Popescu, N.; Nikpayam, M.; Koskas, M.; Uzan, C.; Bichet, J. C.; Canlorbe, G. Breast reconstruction by exclusive lipofilling after total mastectomy for breast cancer: Description of the technique and evaluation of quality of life. *J. Pers. Med.* **2022**, *12*, 25, https://dx.doi.org/10.3390/jpm12020153
337. Di Giuseppe A; Bassetto F; Nahia F (eds). Fat transfer in plastic surgery: Techniques, technology and safety. Switzerland: Springer NatureSwitzerland AG. https://doi.org/10.1007/978-3-031-10881-5.
338. Skillman, J.; McManus, P.; Bhaskar, P.; Hamilton, S.; Roy, P. G.; O'Donoghue, J. M. UK guidelines for lipomodelling of the breast on behalf of Plastic, Reconstructive and Aesthetic Surgery and Association of Breast Surgery Expert Advisory Group. *J. Plast. Reconstr. Aesthet. Surg.* **2022**, *75*, 511-518, https://dx.doi.org/10.1016/j.bjps.2021.09.033
339. Lemaine, V.; Hoskin, T. L.; Farley, D. R.; Grant, C. S.; Boughey, J. C.; Torstenson, T. A.; Jacobson, S. R.; Jakub, J. W.; Degnim, A. C. Introducing the SKIN score: A validated scoring system to assess severity of mastectomy skin flap necrosis. *Ann. Surg. Oncol.* **2015**, *22*, 2925-2932, https://dx.doi.org/10.1245/s10434-015-4409-3
340. Coleman, S. R. Structural fat grafting: More than a permanent filler. *Plast. Reconstr. Surg.* **2006**, *118*, 108S-120S,

341. Coleman, S. R.; Saboeiro, A. P. Fat grafting to the breast revisited: Safety and efficacy. *Plast. Reconstr. Surg.* **2007**, *119*, 775-785; discussion 786-777,
342. Agha, R. A.; Borrelli, M. R.; Vella-Baldacchino, M.; Thavayogan, R.; Orgill, D. P. The STROCSS statement: Strengthening the Reporting of Cohort Studies in Surgery. *Int. J. Surg.* **2017**, *46*, 198-202, 10.1016/j.ijsu.2017.08.586
